# Euphane and Tirucallane Triterpenes with Trypanocidal Activity from *Euphorbia desmondii*

**DOI:** 10.1021/acs.jnatprod.4c00730

**Published:** 2024-09-14

**Authors:** Muhammad Bello Saidu, Gordana Krstić, Anita Barta, Attila Hunyadi, Róbert Berkecz, Umar Shehu Gallah, Kaushavi Cholke, Jürg Gertsch, Dóra Rédei, Judit Hohmann

**Affiliations:** † Department of Pharmacognosy, University of Szeged, Eötvös u. 6, 6720 Szeged, Hungary; ‡ University of Belgrade, Faculty of Chemistry, Studentski trg 12-16, 11158 Belgrade, Serbia; § Institute of Pharmaceutical Analysis, University of Szeged, Somogyi u. 4, 6720 Szeged, Hungary; ∥ Bioresource Department, National Research Institute for Chemical Technology (NARICT), Zaria, 1052, Nigeria; ⊥ Institute of Biochemistry and Molecular Medicine, University of Bern, Bühlstrasse 28, 3012 Bern, Switzerland; # HUN-REN-USZ Biologically Active Natural Products Research Group, University of Szeged, Eötvös u. 6, H-6720 Szeged, Hungary

## Abstract

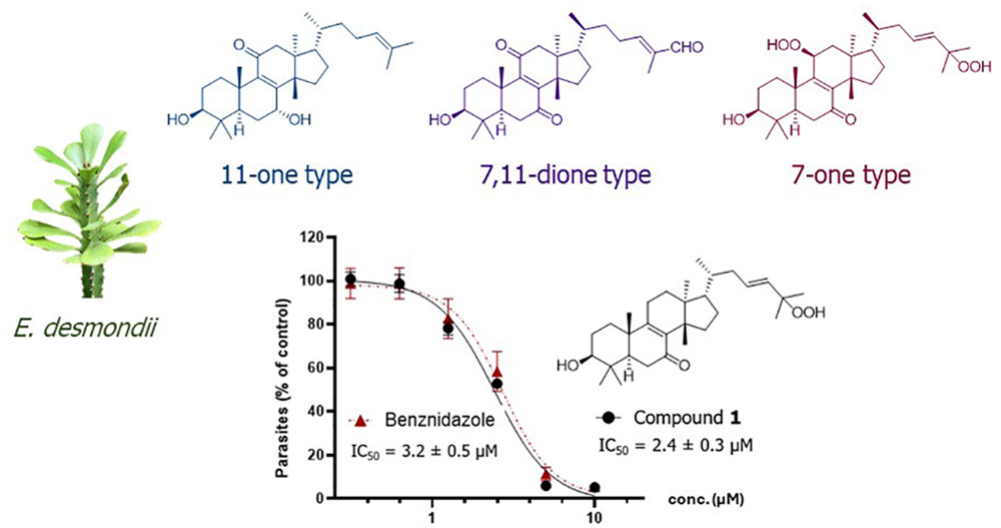

The phytochemical investigation of *Euphorbia desmondii* resulted in the isolation of 15 previously undescribed triterpenoids (desmondiins A, C-P) and 8 already described compounds. The structures of the isolated compounds were determined by extensive spectroscopic analyses. The compounds were identified as tirucallane and euphane triterpenes based on 7-keto-8-ene, 11-keto-8-ene, or 7,11-diketo-8-ene skeletons. Additionally, the selective trypanocidal activities of these compounds against *Trypanosoma cruzi* were evaluated. Desmondiins A, C, D, F, H, and M exhibited IC_50_ values in the range of 3–5 μM, and selectivity indices between 5–9, against *T. cruzi* epimastigotes over the host cell (RAW264.7 macrophages). Furthermore, desmondiin A efficiently inhibited amastigote replication in host cells (IC_50_ = 2.5 ± 0.3 μM), which was comparable to that of the positive control, benznidazole (3.6 ± 0.4 μM). Overall, the isolated euphane and tirucallane triterpenoids could act as antichagasic lead scaffolds.

*Trypanosoma cruzi*, a parasitic protozoan, is the causative agent of Chagas disease (American trypanosomiasis). It is believed that 6–7 million people worldwide, primarily in Latin America, suffer from this disease, which accounts for 12,000 fatalities annually. Further, 10% of patients with persistent infections experience neurological or digestive disorders or both, and up to one-third of these patients experience cardiac changes that may require special care.^1,2^ The dermatologic symptoms of acute infections include conjunctivitis, widespread morbilliform eruption, and localized swelling at the inoculation site (chagoma).^3^ Chagas disease can be transmitted by the triatomine bug (vector-borne) as well as orally (food-borne). Furthermore, it can spread during pregnancy or childbirth, through blood or blood products, during organ transplants, and through laboratory accidents.^4^

The proliferative forms of *T. cruzi* are known as epimastigotes. In the gut of kissing bugs (*Triatoma* spp.), epimastigotes transform into metacyclic trypomastigotes, which are capable of infecting mammals. The disease is primarily transmitted through bites by an infected *T. cruzi* bug. The infected *T. cruzi* bug, which feeds on the blood of mammals (including humans), contaminates human skin with metacyclic trypomastigotes, causing an infection. Thereafter, the parasites exploit a process that is facilitated by self-inflicted scratching and use the proteolytic enzymes present in the saliva of the infected bug to penetrate the bloodstream through the skin.^5,6^

Even though Chagas’ disease was discovered over a century ago, only two nitro derivatives (nifurtimox and benznidazole) have been identified for its treatments. However, both substances are significantly limited by their severe side effects, need a prolonged treatment duration, show selective drug sensitivities toward various *T. cruzi* strains, and are ineffective at the chronic stage of the disease.^7^ Therefore, it is crucial to discover new, less toxic, and more effective therapeutic options for treating the disease.^8,9^

Over 600 plant species belonging to different families have been evaluated for their trypanocidal activities. Screening of extracts and compounds from species of the families Asteraceae, Poaceae, Piperaceae, Euphorbiaceae, Polygalaceae, and Solanaceae have yielded promising results.^10,11^ Following the extant reports on the trypanocidal effects of chemically different triterpenoids,^10^*Euphorbia desmondii* Keay & Milne-Redh (Euphorbiaceae), which is native to West and Central African countries (Ghana, Benin Republic, Nigeria, Niger, Cameroun, and Chad), was investigated in this study.^12,13^ This plant is a shrub or tree that typically grows to a height of 5.2 m. It is called a “male gu” to distinguish it from *E. kamerunica*, which is called “female gu”. Economically, its products are used to produce gums and resins.^14^ Previous phytochemical investigations provided the identification of ingenol in *E. desmondii*.^15,16^ The present study focuses on the isolation of triterpenoids from *E. desmondii* Keay & Milne-Redh (Euphorbiaceae) and evaluation of the trypanocidal activities of the isolated compounds.

## Results and Discussion

### Isolation and Structure Determination of the Compounds

The CHCl_3_ fraction of the MeOH extract prepared from the aerial parts of *E. desmondii* was separated by multistep chromatography to afford 23 pure compounds (**1**–**23**).

Desmondiin A (**1**) was isolated as a white, amorphous powder. Its molecular formula was determined as C_30_H_48_O_4_ by HR-ESI-MS analysis (*m*/*z* 473.3637 [M + H]^+^, calcd. for C_30_H_49_O_4_^+^: 473.3625). The ^1^H and ^13^C NMR JMOD spectra of **1** revealed the presence of one secondary and seven tertiary methyl groups, eight methylenes, and six methines of which one was oxygenated (Tables 1 and 2). The ^13^C NMR JMOD and HSQC spectra indicated eight quaternary carbons (δ_C_ 198.2, 165.2, 139.1, 82.4, 47.9, 44.9, 39.5, and 38.8). Further, the presence of two olefinic groups in the molecule was evident from the proton (δ_H_ 5.54 d and 5.69 m) and carbon chemical shifts (δ_C_ 130.6, 134.9, 139.1, and 165.2), and one keto group was revealed by the carbon signal at δ_C_ 198.2. The structure of **1** was further analyzed by ^1^H–^1^H COSY spectrum, which enabled the elucidation of four structural fragments: C-1–C-3, C-5–C-6, C-11–C-12, and C-15–C-16–C-17–C-20(C-21)–C-22–C-23–C-24 based on the sequences of the correlated protons.
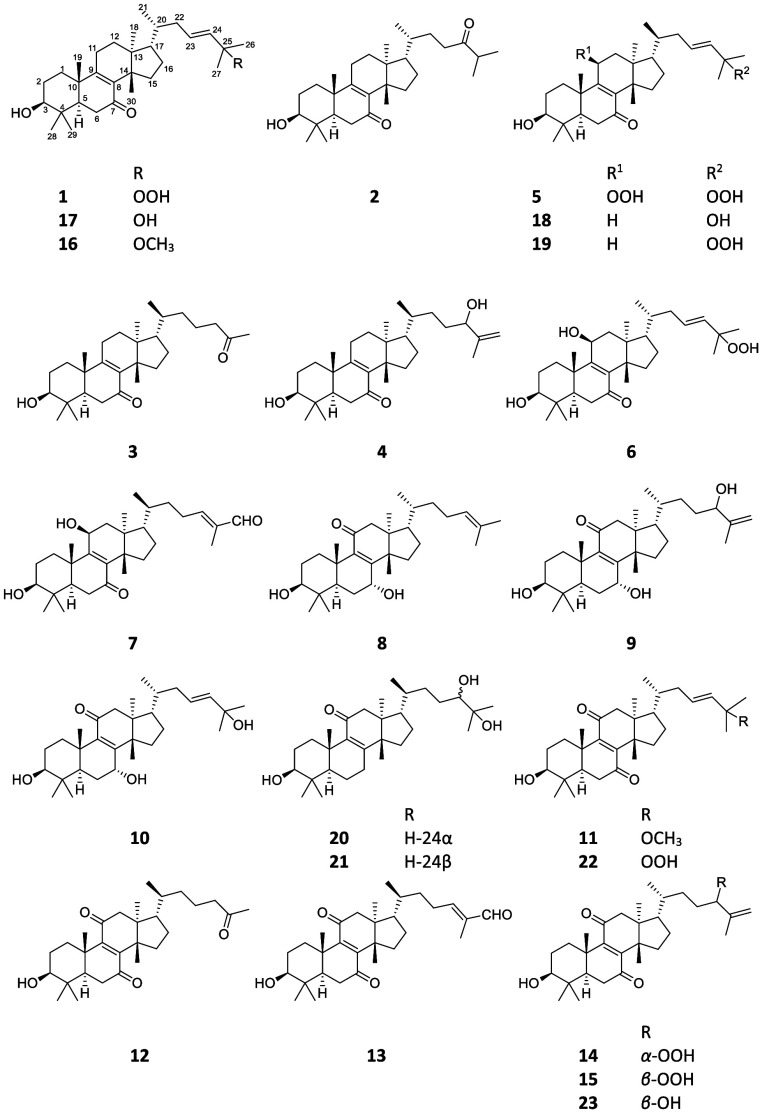


**Table 1:** ^1^H NMR (500 MHz) Data for Compounds **1**–**7** in CDCl_3_

	***δ***_**H**_**,mult. (*****J*****in Hz)**						
**No.**	**1**	**2**	**3**	**4**	**5**	**6**	**7**
**1**	1.44, m	1.43, m	1.43, m	1.43, m	1.54, m	1.56, m	1.78, m
	1.87, dt (13.2, 3.6)	1.86, m	1.86, dt (13.0, 3.4)	1.86, dt (13.0, 3.4)	2.32, m	2.45, m	2.42, m
**2**	1.75, m	1.77, m	1.76, m,	1.75, m	1.78, m	1.77, m (2H)	1.74, m
	1.68, m	1.67, m	1.68, m	1.67, m	1.72, m		
**3**	3.30, m	3.29, dd (11.6, 4.4)	3.30, dd (11.2, 4.3)	3.30, dd (11.5, 4.3)	3.31, dd (11.0, 4.7)	3.32, dd (10.1, 5.8)	3.32, m
**5**	1.68, m	1.68, dd (13.5, 4.7)	1.67, dd (13.6, 4.7)	1.67, dd (13.5, 4.6)	1.65, dd (14.1, 5.2)	1.65, dd (12.5, 5.8)	1.65, dd (12.3, 6.0)
**6**	2.47, m	2.44, dd (18.6, 4.7)	2.42, m	2.40, dd (18.7, 4.6)	2.40, m	2.45, m (2H)	2.45, m (2H)
**6**	2.33, m	2.36, dd (18.6, 13.5)	2.32, m	2.33, dd (18.7, 13.5)	2.46, m		
**11**	2.41, dt (20.1, 9.0)	2.41, m	2.40, m	2.37, m	5.04, t (7.7)	4.70, t (8.2)	4.70, t (7.8)
	2.24, m	2.24, dd (20.7, 7.2)	2.22, ddd (19.4, 6.7, 1.5)	2.22, dd (20.3, 6.6)			
**12**	1.78, m (2H)	1.82, m	1.76, m	1.77, m	2.16, dd (13.4, 7.3)	1.79, m	1.81, m
					2.34, m	2.39, m	2.36, m
**15**	1.54, m	1.51, m	1.56, m	1.52, m	1.44, m	1.44, m	1.44, m
	2.15, m	2.13, tt (10.8, 2.4)	2.13, m	2.12, ddd (12.2, 9.8, 1.7)	2.08, m	2.10, ddd (12.2, 9.6, 2.0)	2.12, m
**16**	1.38, m	1.37, m	1.35, m	1.35, m	1.33, m	1.32, m	1.34, m
	1.95, m	1.94, m	1.95, m	2.00, m	1.92, m	1.92, m	1.95, m
**17**	1.55, m	1.49, m	1.46, m	1.46, m	1.67, m	1.60, m	1.51, m
**18**	0.76, s	0.75, s	0.73, s	0.72, s	0.73, s	0.74, s	0.73, s
**19**	1.06, s	1.05, s	1.06, s	1.05, s	1.17, s	1.26, s	1.26, s
**20**	1.57, m	1.50, m	1.42, m	1.42, m	1.59, m	1.55, s	1.46, m
**21**	0.86, d (6.2)	0.86, d (6.4)	0.94, d (6.3)	0.93, d (6.1)	0.91, d (6.4)	0.86, d (6.4)	0.92, d (6.2)
**22**	1.88, m	1.94, m	1.05, m	0.96, m	2.06, m	1.89, m	1.20, m
	2.38, m	2.14, m	2.32, m	1.42, m	2.31, m	2.39, m	1.85, m
**23**	5.69, m	2.43, m	1.47, m	1.62, m	5.63, ddd (15.5, 8.3, 4.9)	5.65, ddd (15.8, 7.7, 6.3)	2.28 m
			1.66, m	1.42, m			2.54 m
**24**	5.54, d (15.6)		2.39, m	4.02, t (6.6)	5.59, d (15.5)	5.55, d (15.8)	6.48, t (7.2)
**25**		2.61, sept (6.8)					
**26**	1.34, s	1.10, d (6.8)	2.13, s	4.84, br s, 4.93, br s	1.30, s	1.33, s	9.40, s
**27**	1.34, s	1.10, d (6.8)	–	1.72, s	1.38, s	1.34, s	1.76, s
**28**	1.00, s	0.99, s	1.00, s	0.96, s	1.00, s	1.00, s	1.00, s
**29**	0.89, s	0.88, s	0.89, s	0.88, s	0.92, s	0.91, s	0.92, s
**30**	0.98, s	0.98, s	0.97, s	0.99, s	1.12, s	1.14, s	1.16, s

**Table 2:** ^13^C NMR (125 MHz) Data for Compounds **1**–**7** in CDCl_3_

	***δ***_**C**_**,type**						
**No.**	**1**	**2**	**3**	**4**	**5**	**6**	**7**
**1**	34.8, CH_2_	34.8, CH_2_	34.8, CH_2_	34.8, CH_2_	34.1, CH_2_	33.8, CH_2_	33.8, CH_2_
**2**	27.6, CH_2_	27.7, CH_2_	27.6, CH_2_	27.6, CH_2_	27.5, CH_2_	27.6, CH_2_	27.5, CH_2_
**3**	78.3, CH	78.3, CH	78.3, CH	78.2, CH	78.4, CH	78.4, CH	78.4, CH
**4**	38.8, C	39.0, C	39.0, C	39.0, C	39.2, C	39.2, C	39.2, C
**5**	48.5, CH	48.5, CH	48.5, CH	48.4, CH	49.4, CH	49.4, CH	49.4, CH
**6**	36.0, CH_2_	35.8, CH_2_	35.9, CH_2_	35.9, CH_2_	36.0, CH_2_	36.0, CH_2_	36.0, CH_2_
**7**	198.2, C	198.3, C	198.3, C	198.4, C	199.7, C	200.2, C	200.1, C
**8**	139.1, C	139.1, C	139.1, C	139.1, C	143.5, C	140.5, C	140.5, C
**9**	165.2, C	165.4, C	165.4, C	165.5, C	157.3, C	161.3, C	161.1, C
**10**	39.5, C	39.5, C	39.5, C	39.4, C	38.4, C	39.7, C	39.7, C
**11**	23.8, CH_2_	23.9, CH_2_	23.8, CH_2_	23.8, CH_2_	81.1, CH	68.2, CH	68.2, CH
**12**	30.3, CH_2_	30.2, CH_2_	30.1, CH_2_	30.0, CH_2_	38.2, CH_2_	43.0, CH_2_	43.1, CH_2_
**13**	44.9, C	44.9, C	44.8, C	44.8, C	46.4, C	46.4, C	46.3, C
**14**	47.9, C	47.9, C	47.8, C	47.8, C	48.0, C	48.1, C	48.2, C
**15**	31.6, CH_2_	31.6, CH_2_	31.6, CH_2_	31.6, CH_2_	32.0, CH_2_	31.9, CH_2_	31.9, CH_2_
**16**	28.5, CH_2_	28.8, CH_2_	28.8, CH_2_	28.7, CH_2_	27.6, CH_2_	27.7, CH_2_	28.0, CH_2_
**17**	48.4, CH	48.6, CH	48.9, CH	48.8, CH	48.3, CH	48.6, CH	48.9, CH
**18**	16.1, CH_3_	15.9, CH_3_	15.7, CH_3_	15.7, CH_3_	17.1, CH_3_	16.6, CH_3_	16.5, CH_3_
**19**	18.8, CH_3_	18.8, CH_3_	18.8, CH_3_	18.8, CH_3_	19.2, CH_3_	19.9, CH_3_	19.9, CH_3_
**20**	36.0, CH	35.7, CH	36.4, CH	36.4, CH	35.5, CH	35.9, CH	35.8, CH
**21**	19.3, CH_3_	19.0, CH_3_	18.9, CH_3_	19.0, CH_3_	19.6, CH_3_	19.3, CH_3_	18.8, CH_3_
**22**	38.8, CH_2_	30.1, CH_2_	36.0, CH_2_	31.8, CH_2_	38.1, CH_2_	38.6, CH_2_	34.6, CH_2_
**23**	130.6, CH	37.4, CH_2_	20.9, CH_2_	32.1, CH_2_	129.2, CH	130.1, CH	26.2, CH_2_
**24**	134.9, CH	215.3, CH_2_	44.4, CH_2_	76.5, CH	135.8, CH	135.1, CH	155.0, CH
**25**	82.4, C	41.1, CH	209.2, C	147.6, C	82.5, C	82.4, C	139.9, C
**26**	24.6, CH_3_	18.8, CH_3_	30.0, CH_3_	111.6, CH_2_	24.9, CH_3_	24.5, CH_3_	195.4, CH
**27**	24.6, CH_3_	18.8, CH_3_	–	17.3, CH_3_	23.8, CH_3_	24.5, CH_3_	9.4, CH_3_
**28**	27.5, CH_3_	27.3, CH_3_	27.5, CH_3_	24.5, CH_3_	27.8, CH_3_	27.7, CH_3_	27.7, CH_3_
**29**	15.2, CH_3_	15.2, CH_3_	15.2, CH_3_	15.2, CH_3_	15.4, CH_3_	15.3, CH_3_	15.3, CH_3_
**30**	24.6, CH_3_	24.3, CH_3_	24.5, CH_3_	27.4, CH_3_	25.3, CH_3_	25.9, CH_3_	25.9, CH_3_

**Figure 1:**
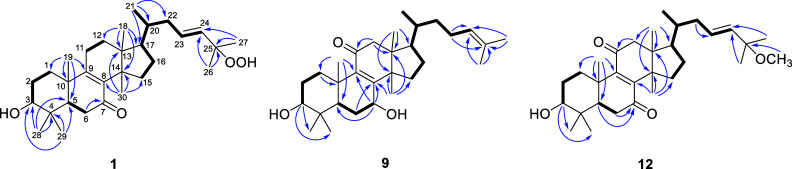
Key ^1^H–^1^H COSY (−) and HMBC correlations (H → C) of compounds **1**, **9**, and **12**.

These structural units, along with the quaternary carbons, were connected by long-range heteronuclear correlations extracted from an HMBC spectrum. The most informative HMBC correlations were those of the methyl groups as follows: H_3_-18 to C-12, C-13, C-14, and C-17; H_3_-19 to C-1, C-5, C-10, and C-9; H_3_-21 to C-17, C-20, and C-22; H_3_-26 and H_3_-27 to C-24 and C-25; H_3_-28 and H_3_-29 to C-3, C-4, and C-5; and H_3_-30 to C-8, C-13, C-14, and C-15 (Figure 1). These correlations allowed the establishment of the planar structure as a tetracyclic euphane- or tirucallane-type triterpene. The position of the keto group at C-7 was confirmed by the HMBC cross peaks of H_2_-6 (δ_H_ 2.33, 2.47) with C-7 (δ_C_ 198.2). A tetrasubstituted olefin group was placed at position C-8–C-9, as indicated by the correlations of H_3_-19 with C-9 and H_3_-30 with C-8; the disubstituted olefin was positioned at C-23–C-24, as revealed by the correlations of H_3_-26 and H_3_-27 with C-24. The 26- and 27-methyl groups displayed correlations with the quaternary carbon at δ_C_ 82.4 (C-25); this high chemical shift value combined with the molecular formula confirmed the presence of a hydroperoxy group (OOH) at C-25.^17^

The relative configuration of compound **1** was determined using the NOESY experiment. The Overhauser effect observed between H-3 and H-5, as well as H_3_-18 and H-11α indicated the α orientation of these groups and hydrogens (Figure 3). Conversely, the NOESY correlations between H-11β and H_3_-19 and H_3_-30 and H-17 revealed the stereochemistry of rings A–D bearing an α-oriented 18-methyl group and β-oriented 19- and 30-methyl groups. To determine the stereochemistry of C-20, two arguments were considered: the NOESY correlation of H_3_-21 with H-16α (δ_H_ 1.38 m) exhibiting an α-oriented 21-methyl group and the ^1^H NMR chemical shift of H_3_-21 at δ_H_ 0.86, which is characteristic to the euphane triterpenes (instead of H-21 at δ_H_ 0.94, which is characteristic to tirucallane triterpenes).^18−21^ Therefore, compound **1** was confirmed to be (23*E*)-25-hydroperoxyeupha-8,23-diene-3β-ol-7-one, which was named desmondiin A. Compound **1** is a stereoisomer of (23*E*)-25-hydroperoxytirucalla-8,23-dien-3β-ol-7-one (**19**) isolated from *Euphorbia micractina*.^17^

Desmondiin C (**2**) was isolated as a white, amorphous powder. Its molecular formula was C_30_H_48_O_3_, as determined by HR-ESI-MS analysis (*m*/*z* 457.3688 [M + H]^+^, calcd. for C_30_H_49_O_3_^+^: 457.3676). The 1D- and 2D-NMR spectra of **2** revealed that it shares the same structural series as **1**. Further, the ^1^H and ^13^C JMOD NMR spectra of **2** indicated two keto groups (δ_C_ = 198.3 and 215.3) (Tables 1 and 2). The C-23–C-24 olefin bond and hydroxyl group at C-25 were absent, and a 24-keto group and an isopropyl group [C-25(C-26)–C-27] were detected. The position of the keto group at C-24 was supported by the HMBC correlations of H-23 (δ_H_ 2.43 m) with C-24 (δ_C_ 215.3), and the isopropyl group connected to the keto group was shown by the HMBC cross peaks of H_3_-26 and H_3_-27 (both at δ_H_ 1.10 d) with C-24. Thus, it was confirmed that desmondiin C (**2**) was eupha-8-ene-3β-ol-7,24-dione.

**Figure 2:**
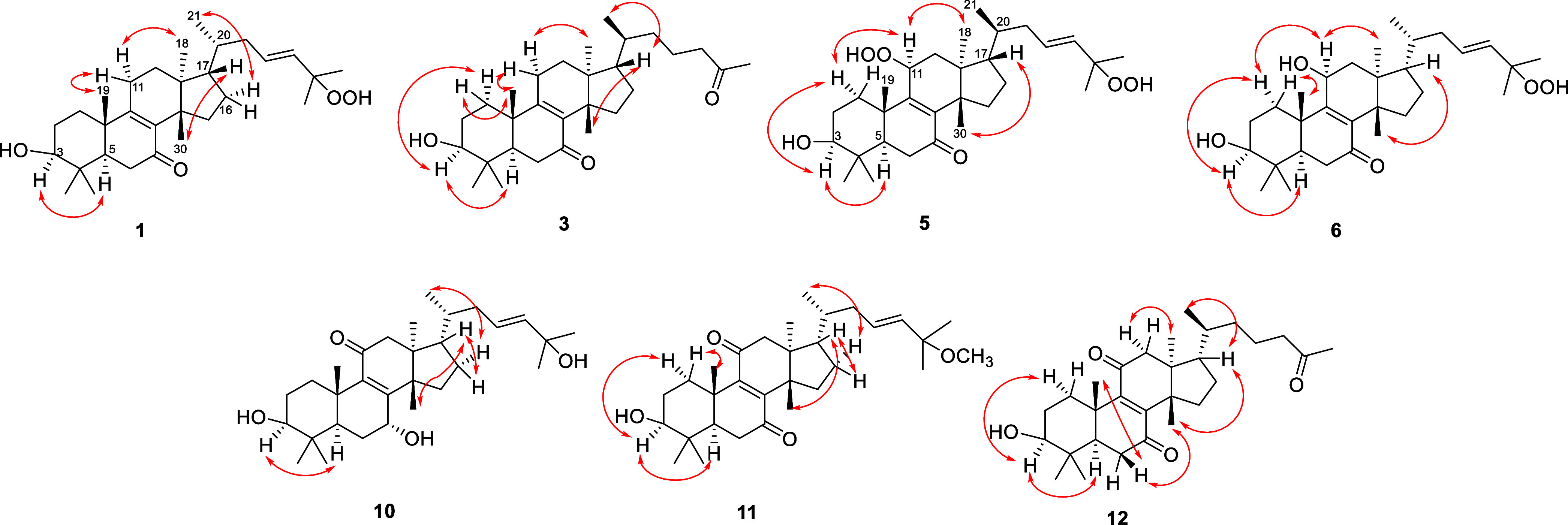
Key NOESY correlations of compounds **1**, **3**, **5**, **6**, **10**–**12**.

Desmondiin D (**3**) was isolated as a white, amorphous powder. Its molecular formula was C_29_H_46_O_3_, as confirmed by HR-ESI-MS analysis (*m*/*z* 443.3527 [M + H]^+^, calcd. for C_29_H_47_O_3_^+^: 443.3520). The ^1^H and ^13^C JMOD NMR spectra of **3** indicated that it exhibited the same 3-hydroxy-7-keto-8-ene-substituted skeleton as **1** and **2**, but in **3**, a seven-carbon-containing moiety at C-17 was elucidated. This side chain comprised two methyls, three methylenes, one methine, and one keto group (Tables 1 and 2). The keto group was positioned at C-25 because of its HMBC correlation with the terminal 26-methyl group (δ_H_ = 2.13 s). ^1^H–^1^H COSY correlation between H_2_-24 and H_2_-23, and HMBC cross-peaks between H_3_-21 and C-22, and H_3_-26 and C-25 provided evidence to the structure of the C_7_ side chain. Further, diagnostic NOESY correlation between H-11α/H_3_-18, H-11β/H_3_-19, H_3_-19/H-1β, H-1α/H-3α, H-3α/H-5α, H_3_-30/H-17, H-17/H_3_-21 (Figure 2) indicated the stereostructure of desmondiin D (**3**).

Desmondiin E (**4**) was isolated as a white, amorphous powder. HR-ESI-MS data provided its molecular formula C_30_H_48_O_3_ (*m*/*z* 457.3685 [M + H]^+^, calcd. for C_30_H_49_O_3_^+^: 457.3676). The 1D and 2D NMR spectral data of **4** displayed the same tirucallan-8-en-7-one structure as that of **3** (Tables 1 and 2), the differences being in the C-24–C-27 part of the molecule. This part comprised a quaternary carbon (δ_C_ 147.6), a methine (δ_H_ 4.02 t; δ_C_ 76.5), a methylidene (δ_H_ 4.84 br s, 4.93 br s; δ_C_ 111.6), and one methyl group (δ_H_ 1.72 s; δ_C_ 17.3). HMBC correlations of this methyl group with C-25, C-26, and C-24), in addition to the correlations of the methine proton (δ_H_ 4.02) with C-25 and C-22 confirmed the presence of the 24-hydroxy-25-ene-substituted side chain and allowed the structure of desmondiin E, as depicted in the structural formula **4**. This compounds was synthetised by reduction of (24*R*)-24-hydroperoxytirucalla-8,25-dien-3-ol-7-one by Xu et al.^17^ The stereoisomer of **4**, the euphane sooneuphanone was isolated from *Euphorbia soongarica*.^22^

The molecular formula of desmondiin F (**5**) was C_30_H_48_O_6_, as determined by the HR-APCI-MS analysis (*m*/*z* 505.3519 [M + H]^+^, calcd. for C_30_H_49_O_6_^+^: 505.3524). The evaluation of the 1D- and 2D-NMR spectra of **5** revealed that it was similar to the known compound, (23*E*)-25-hydroperoxytirucalla-8,23-dien-3β-ol-7-one (**19**),^17^ also isolated in this study. However, the resonances attributed to the 11-methylene of **19** were replaced by that of a methine group (δ_H_ 5.04 t; δ_C_ 81.1) in **5** (Tables 1 and 2). The position of this oxygenated methine at C-11 was supported by the HMBC correlations between the proton signal at δ_H_ 5.04 (H-11) with the carbon signals at δ_C_ 157.3 (C-9), 143.5 (C-8), and 38.2 (C-12) in the HMBC spectrum. The deshielded ^13^C NMR signal of C-11 (δ_C_ 81.1) indicated the presence of a hydroxyperoxy group in this position,^23^ corresponding to the molecular formula. The NOESY experiment of **5** confirmed the tirucallane skeleton and α-position of H-11 by demonstrating the NOE correlation between H-11α and H_3_-18 (Figure 2). The β-oriented 11-hydroxyperoxy group was consistent with the coupling constant of H-11, i.e., 7.7 Hz.^24^ Therefore, the structure of desmondiin F (**5**) was elucidated as (23*E*)-11,25-dihydroperoxytirucalla-8,23-dien-3β-ol-7-one.

Desmondiin G (**6**) had the molecular formula C_30_H_48_O_5_ based on the HR-APCI-MS analysis (*m*/*z* 489.3568 [M + H]^+^, calcd. for C_30_H_49_O_5_^+^: 489.3575; *m*/*z* 455.3512 [M – OOH]^+^, calcd. for C_30_H_47_O_3_^+^: 455.3520). The 1D- and 2D-NMR spectra of **6** revealed that this compound was an euphane triterpene substituted with one keto and two hydroxy groups, and contained a tetrasubstituted olefin and the same side chain at C-17, as in compound **1** (Tables 1 and 2). The position of the keto group (δ_C_ 200.2) at C-7 was determined by its HMBC correlation with those of H-5 and H-6. The presence of the 11β-hydroxy group was confirmed by the HMBC cross peaks of H_2_-12 with C-11 and the NOESY correlation of H-11 with H-1α (δ_H_ 1.56) and H_3_-18 (Figure 2). The presence of a C-8–C-9 double bond was confirmed from the long-range correlations of H_3_-19 and H_3_-30 with carbon signals at δ_C_ 140.5 (C-8) and 161.3 (C-9), respectively. The 23-ene-25-hydroperoxy structure of **7** corresponded to the MS data as well as the ^1^H and ^13^C chemical shift assignments of **1**. Thus, desmondiin G was confirmed as (23*E*)-25-hydroperoxyeupha-8,23-diene-3β,11β-diol-7-one (**6**).

Desmondiin H (**7**) exhibited a quasi-molecular ion at *m*/*z* 471.3465 [M + H]^+^ (calcd. for C_30_H_47_O_4_ 471.3469) in the positive HR-APCI-MS spectrum, indicating that its molecular formula was C_30_H_46_O_4_. The NMR spectroscopic features of **7** were similar to those of **6**, except for the aliphatic chain at C-17. In this part of compound **7**, a trisubstituted olefin group (δ_H_ 6.48 t; δ_C_ 155.0, 139.9) and an aldehyde group (δ_H_ 9.40 s; δ_C_ 195.4) were detected (Tables 1 and 2). The methyl group at δ_H_ 1.76 s (H_3_-27) exhibited HMBC correlation with the olefinic and aldehyde carbons, indicating that it exhibited a 24-en-26-al structure. The key NOESY correlations were the same as for compound **5** indicating that desmondiin H (**7**) comprised a tirucallane skeleton.

Desmondiin I (**8**) had a molecular formula C_30_H_48_O_3_, as confirmed the HR-ESI-MS analysis (*m*/*z* 457.3687 [M + H]^+^, calcd. for C_30_H_49_O_3_^+^: 457.3676). The ^1^H and ^13^C NMR spectra displayed signals that were attributable to an euphane triterpene containing two hydroxylated methines (δ_H_ 3.31 dd, 4.27 dd; δ_C_ 78.8, 65.6), one trisubstituted (δ_H_ 5.08 t; 131.4) and one tetrasubstituted olefin group (δ_C_ 141.6, 157.3), and one keto group (δ_C_ 200.4) (Tables 3 and 4). The HMBC spectrum facilitated the determination of the positions of these functionalities (Figure 1): the hydroxy groups were positioned at C-3 and C-7 with respect to the correlations of H-3 with C-28 and C-29 and H-7 with C-5, C-8, and C-9. The position of the keto group at C-11 was elucidated based on its HMBC cross peaks with H_2_-12.

**Table 3:** ^1^H NMR (500 MHz) Data for Compounds **8**–**10** in CDCl_3_

	***δ***_**H**_**,mult. (*****J*****in Hz)**		
**No.**	**8**	**9**	**10**
**1**	1.01, m	1.01, m	1.00, m
	2.56, dt (13.4, 3.4)	2.57, dt (13.3, 3.2)	2.58, m
**2**	1.70, m	1.71, m	1.70, m
		1.67, m	
**3**	3.31, dd (11.3, 5.2)	3.32, dd (10.4, 3.4)	3.32, dd (11.2, 5.0)
**5**	1.42, m	1.43, m	1.40, m
**6**	1.83, m	1.83, d (14.1)	1.82, d (14.0)
		1.53, m	1.69, m
**7**	4.27, dd (3.6, 1.7)	4.27, br s	4.27, br s
**12**	2.60, d (18.9)	2.62, m	2.60, d (18.8)
	2.44, d (18.9)	2.42, m	2.42, d (18.8)
**15**	2.36, m	2.36, m	2.37, m
	1.39, m	1.40, m	1.41, m
**16**	1.42, m	1.42, m	1.43, m
	2.01, m	2.00, m	2.00, m
**17**	1.66, m	1.68, m	1.69, m
**18**	0.92, s	0.92, s	0.95, s
**19**	1.17, s	1.17, s	1.17, m
**20**	1.46, m	1.47, m	1.53, m
**21**	0.82, d (6.4)	0.89, d (6.3)	0.86, d (6.8)
**22**	1.06, m	1.07, m	1.71, m
	1.49, m	1.68, m	2.25, dt (13.3, 3.9)
**23**	1.86, m	1.43, m	5.60, d (15.6)
	2.09, m	1.62, m	
**24**	5.08, t (7.3)	4.02, t (6.3)	5.55, dd (16.1, 6.6)
**26**	1.68, s	4.85, s	1.30, s
		4.94, s	
**27**	1.60, s	1.72, s	1.30, s
**28**	1.04, s	1.05, s	1.05, s
**29**	0.85, s	0.85, s	0.85, s
**30**	1.01, s	1.01, s	1.02, s

**Table 4:** ^13^C NMR (125 MHz) Data for Compounds **8**–**10** in CDCl_3_

	***δ***_**C**_**,type**		
**No.**	**8**	**9**	**10**
**1**	33.9, CH_2_	34.0, CH_2_	33.9, CH_2_
**2**	28.0, CH_2_	28.0, CH_2_	28.0, CH_2_
**3**	78.8, CH	78.8, CH	78.8, CH
**4**	38.5, C	38.6, C	38.6, C
**5**	45.5, CH	45.6, CH	45.6, CH
**6**	28.6, CH_2_	28.6, CH_2_	28.6, CH_2_
**7**	65.6, CH	65.6, CH	65.6, CH
**8**	157.3, C	157.2, C	157.2, C
**9**	141.6, C	141.6, C	141.6, C
**10**	38.6, C	38.6, C	38.6, C
**11**	200.4, C	200.3, C	200.2, C
**12**	51.8, CH_2_	51.7, CH_2_	51.6, CH_2_
**13**	44.3, C	44.3, C	44.4, C
**14**	51.1, C	51.1, C	51.1, C
**15**	29.7, CH_2_	29.7, CH_2_	29.7, CH_2_
**16**	27.8, CH_2_	27.8, CH_2_	27.6, CH_2_
**17**	50.8, CH	50.8, CH	50.4, CH
**18**	18.3, CH_3_	18.5, CH_3_	18.5, CH_3_
**19**	18.1, CH_3_	18.4, CH_3_	18.1, CH_3_
**20**	36.0, CH	36.1, CH	36.3, CH
**21**	18.6, CH_3_	18.8, CH_3_	18.9, CH_3_
**22**	35.3, CH_2_	30.9, CH_2_	38.0, CH_2_
**23**	25.3, CH_2_	32.1, CH_2_	125.1, CH
**24**	124.8, CH	76.1, C	139.9, CH
**25**	131.4, C	147.9, C	70.9, C
**26**	25.8, CH_3_	111.3, CH_2_	30.1, CH_3_
**27**	17.8, CH_3_	17.7, CH_3_	30.1, CH_3_
**28**	28.3, CH_3_	28.3, CH_3_	28.3, CH_3_
**29**	16.2, CH_3_	16.3, CH_3_	16.2, CH_3_
**30**	26.0, CH_3_	26.1, CH_3_	26.0, CH_3_

The 8,9-olefin group was confirmed by the HMBC correlations of H_3_-30 and H_2_-6 with C-8 and H_3_-19 with C-9. The 24,25 double bond was proved by the correlations of H_3_-27, H_3_-26, and H-23 with C-24 and C-25. The NOESY experiment allowed the stereochemical assignment of **8** considering the H-3/H-7, H-7/H_3_-30, H-20/H-12β, H-16α/H_3_-21, H-16β/H-17, and H_3_-30/H-17 cross-peaks Thus, the structure of desmondiin I corresponded to eupha-8,24-dien-3β,7α-diol-11-one (**8**).

Desmondiin J (**9**) was isolated as a white amorphous powder with a molecular formula C_30_H_48_O_4_, as established from the HR-APCI-MS analysis (*m*/*z* 473.3620 [M + H]^+^, calcd. for C_30_H_49_O_4_^+^: 473.3625). This compound displayed structural patterns similar to those of **8**, only differentiated by the side chain at C-17 which was similar to that of compound **4** (Tables 3 and 4). The key NOESY correlation of H_3_-30 with H-7, and H-3 with H-5 confirmed the presence of 3β- and 7α-hydroxy groups.

HR-APCI-MS spectrum of desmondiin K (**10**) displayed a protonated molecular ion peak at *m*/*z* 473.3620 [M + H]^+^ (calcd. for C_30_H_49_O_4_^+^: 473.3625), corresponding to a molecular formula C_30_H_46_O_4_. A comparison of the 1D- and 2D-NMR data of **10** with those of **8** indicated that these compounds only differed in positions C-23–C-27 (Tables 3 and 4). This structural part comprised a disubstituted olefin group [δ_H_ 5.55 dd (*J* = 15.8, 6.6 Hz), 5.60 d (*J* = 15.8 Hz), δ_C_ 125.1, 139.9.], one oxygen-substituted quaternary carbon (δ_C_ 70.9), and two methyl groups (δ_H_ 2 × 1.30 s, δ_C_ 2 × 30.1). HMBC correlations of H-23, H_3_-26, and H_3_-27 with C-25 and those of H-24 with C-26 and C-27 demonstrated the presence of a double bond at C-23–C-24 and a hydroxyl group at C-25. The stereochemical assignment of desmondiin K (**10**) was made based on the NOESY correlations as depicted on Figure 2.

The molecular formula of desmondiin L (**11**) was C_31_H_48_O_4_, as provided by the HR-APCI-MS analysis (*m*/*z* 485.3618 [M + H]^+^, calcd. for C_31_H_49_O_4_^+^: 485.3625; *m*/*z* 471.3462 [M + H – CH_2_]^+^, calcd. for C_30_H_47_O_4_^+^: 471.3470); *m*/*z* 453.3359 [M + H – CH_3_OH]^+^, calcd. for C_30_H_45_O_3_^+^: 453.3363). The ^1^H NMR and ^13^C NMR spectra revealed that **11** was a triterpene comprising two keto groups, two olefin bonds, one methoxy, and one hydroxy group, as indicated by the carbon at δ_C_ 78.1 (C-3) (Tables 5 and 6). The keto groups were positioned at C-7 and C-11 based on the HMBC connectivities of H-5 and H_2_-6 to C-7 and H_2_-12 to C-11 (Figure 1). The positions of the olefin groups at C-8–C-9 and C-23–C-24 were confirmed by the HMBC correlations H_3_-30/C-8, H_3_-19/C-9, and H_2_-22/C-23, H_3_-26, and H_3_-27/C-24, respectively. The 25-methoxy substitution was evident from the long-range correlation of the OCH_3_ protons (δ_H_ 3.15) with C-25 (δ_C_ 74.9). The NOESY correlations shown in Figure 2 led to the conclusion that desmondiin L is eupha-8,23-dien-3β-hydroxy-25-methoxy-8,11-dione (**11**).

**Table 5:** ^1^H NMR (500 MHz) Data for Compounds **11**–**15** in CDCl_3_

	***δ***_**H**_**,mult. (*****J*****in Hz)**			
**No.**	**11**	**12**	**13**	**14 + 15**
**1**	1.10, m	1.11, m	1.05, m	1.11, m
	2.55, m	2.52, m	2.45, m	2.52, m
**2**	1.67, m	1.73, m	1.71, m	1.74, m
	1.76, m	1.74, m		
**3**	3.30, dd (10.3, 5.5)	3.30, m	3.28, m	3.30, dd (10.4, 5.5)
**5**	1.64, m	1.64, dd (13.5, 4.9)	1.65, m	
**6**	2.44, m	2.48, m	2.49, m	1.65, m
	2.55, m	2.52, m	2.54, m	2.48, m
**12**	2.48, d (18.7)	2.43, d (19.0)	2.44, d (18.7)	2.44, m
	2.67, d (18.7)	2.65, d (19.0)	2.68, d (18.7)	2.64, m
**15**	1.66, m	1.66, m	1.65, m	1.65, m
	2.15, t (11.2)	2.13, m	2.16, m	2.14, m
**16**	1.41, m	1.38, m	1.40, m	1.40, m
	2.02, m	2.02, m	2.04, m	2.02, m
**17**	1.68, m	1.65, m	1.67, m	1.65, m
**18**	0.95, s	0.92, s	0.93, s	0.92, s
**19**	1.31, s	1.31, s	1.31, s	1.30, s
**20**	1.53, m	1.46, m	1.55, m	1.46, m
**21**	0.87, d, 6.5	0.92, d (6.8)	0.94, d (6.4)	0.87, d (6.4)
**22**	1.73, m	0.99, m	1.24, m	1.10, m
	2.27, dt, (13.7, 3.3)	1.44, m	1.65, m	1.50, m
**23**	5.48, m	1.46, m	2.29, m	1.37, m
		1.65, m	2.42, m	1.65, m
**24**	5.40, d (15.8)	2.40, t (7.0)	6.45, t (7.3)	4.27, t (6.5)
**25**	1.25, s			
**26**	1.25, s	2.13, s	9.40, s	5.02, br s
				5.04, m
**27**			1.75, s	1.74, s
**28**	1.03, s	1.03, s	1.03, s	1.03, s
**29**	0.90, s	0.91, s	0.90, s	0.90, s
**30**	1.09, s	1.08, s	1.09, s	1.08, s
**25-OCH**_**3**_	3.15, s			

**Table 6:** ^13^C NMR (125 MHz) Data for Compounds **11**–**15** in CDCl_3_

	***δ***_**C**_**,type**			
**No.**	**11**	**12**	**13**	**14, 15**
**1**	34.1, CH_2_	34.1, CH_2_	34.1, CH_2_	34.1, CH_2_
**2**	27.6, CH_2_	27.6, CH_2_	27.6, CH_2_	27.6, CH_2_
**3**	78.1, CH	78.2, CH	78.1, CH	78.1, CH
**4**	38.7, C	38.8, C	38.7, C	38.7, C
**5**	48.7, CH	48.7, CH	48.6, CH	48.6, CH
**6**	35.9, CH_2_	36.0, CH_2_	35.9, CH_2_	35.9, CH_2_
**7**	200.0, C	199.9, C	200.0, C	200.0, C
**8**	149.9, C	150.0, C	149.9, C	149.9, C
**9**	154.9, C	154.9, C	154.9, C	155.0, C
**10**	38.2, C	38.3, C	38.2, C	38.2, C
**11**	201.8, C	202.0, C	201.7, C	202.0, C
**12**	51.5, CH_2_	51.6, CH_2_	51.6, CH_2_	51.5/51.6, CH_2_
**13**	45.3, C	45.3, C	45.2, C	45.3, C
**14**	48.0, C	48.1, C	48.1, C	48.1, C
**15**	31.9, CH_2_	32.0, CH_2_	31.9, C	31.9, CH_2_
**16**	28.1, CH_2_	28.3, CH_2_	28.3, CH_2_	27.9, CH_2_
**17**	49.2, CH	49.5, CH	48.6, CH	48.6, CH
**18**	18.8, CH_3_	18.7, CH_3_	18.8, CH_3_	18.6, CH_3_
**19**	17.9, CH_3_	17.9, CH_3_	17.9, CH_3_	17.9, CH_3_
**20**	36.2, CH	36.1, CH	35.9, CH	35.8, CH
**21**	18.9, CH_3_	18.5, CH_3_	18.5, CH_3_	18.8, CH_3_
**22**	38.2, CH_2_	34.6, CH_2_	34.1, CH_2_	30.7/31.0, CH_2_
**23**	128.2, CH	21.0, CH_2_	26.4, CH_2_	27.6, CH_2_
**24**	137.3, CH	44.0, CH_2_	154.4, CH	89.5/90.1, CH
**25**	74.9, C	208.8, C	139.6, C	144.0/143.7, C
**26**	26.0, CH_3_	30.0, CH_3_	195.3, CH	114.3/114.8, CH_2_
**27**	26.0, CH_3_		9.4, CH_3_	17.2/17.4, CH_3_
**28**	27.7, CH_3_	27.8, CH_3_	27.7, CH_3_	27.7, CH_3_
**29**	15.2, CH_3_	15.2, CH_3_	15.3, CH_3_	15.2, CH_3_
**30**	24.1, CH_3_	24.2, CH_3_	24.1, CH_3_	24.2, CH_3_
**25–OCH**_**3**_	50.4			

HR-ESI-MS analysis of desmondiin M (**12**) displayed an [M + H]^+^ ion at *m*/*z* 457.3325, corresponding to a molecular formula C_29_H_44_O_4_ (calcd. for C_29_H_44_O_4_^+^: 457.3312), indicating that it was a nor-triterpene. The ^1^H and ^13^C JMOD NMR spectra of **12** were similar to those of **11**, except for the side chain connected at C-17 (Tables 5 and 6). The C-23–C-24 double bond in **12** was saturated, and an acetyl group (δ_C_ 30.0, 208.8, δ_H_ 2.13 s) was detected at C-24. This was supported by the HMBC correlations of H_2_-24 (δ_H_ 2.40 t) and H_3_-26 (δ_H_ 2.13 s) with C-25 (δ_C_ 208.8). Further, HMBC correlations between H_2_-24 and C-22, C-23 and between H_3_-21 and C-22 justified the shortened (C_7_) side chain at C-17. NOESY correlations (Figure 2) proved the tirucallane skeleton by the cross-peaks of H-17 with H_3_-21 and H_3_-30, and H-20 with H_3_-18 thus, the structure 3β-hydroxy-27-nor-tirucalla-8-en-7,11,25-trione (**12**) was elucidated for desmondiin M (Figure 1).

Desmondiin N (**13**), a white amorphous powder, had a molecular formula C_30_H_44_O_4_ as confirmed by HR-ESI-MS analysis (*m*/*z* 469.3308 [M + H]^+^, calcd for C_30_H_45_O_4_^+^: 469.3312). The NMR data of **13** (Tables 5 and 6) indicated that it was very similar to those of **11** and **12**, except for the side chain at C-17, which was elucidated as the same as that of desmondiin G (**7**). Accordingly, the structure of desmondiin N was determined as **13**.

The isomeric compounds desmondiin O and P (**14**, **15**) were isolated as a mixture. Their molecular formulas are C_30_H_46_O_5_ each, as calculated using the HR-ESI-MS peak at *m*/*z* 487.3430 [M + H]^+^ (calcd. for C_30_H_47_O_5_^+^: 487.3418). A comparison of the ^1^H and ^13^C JMOD NMR spectra of **14** and **15** (Tables 5 and 6) revealed that they are different in the C_8_ aliphatic chain, as indicated by the duplicated carbon signals assigned to this part (δ_C-22_ 30.7/31.0, δ_C-24_ 89.5/90.1, δ_C-25_ 143.7/144.0, δ_C-26_ 114.3/114.8, and δ_C-27_ 17.2/17.4). Compounds **14** and **15** are structurally similar to euphorol A isolated from *Euphorbia resinifera* latex, although the ^1^H (δ_H24_ 4.27 t) and ^13^C NMR signals (δ_C-24_ 89.5/90.1) of 24-methine group of **14** and **15** were downfield-shifted compared to those of euphorol A (δ_H-24_ 4.01 t; δ_C-24_ 76.5).^23^ Such deshielding of the NMR signals indicated the presence of a hydroperoxy group instead of a hydroxy group.^17^ Based on the above data, desmondiins O and P were identified as 24α (**14**) and 24β (**15**) stereoisomers of eupha-8,25-dien-3β,24-dihydroxy-8,11-dione.

Compounds **16** (named desmondiin B),^25^**17**,^21^**18**,^17,18^**19**,^17^**20** (neritriterpenol J),^23^**21** (neritriterpenol K),^23^**22**,^17,26^ and **23**(24) were previously reported from *E. sikkimensis*, *E. kansui*, *E. micractina*, *Monadenium lugardae*, *E. neriifolia*, *E. humifusa*, and *E. resinifera*.

### Determination of the Trypanocidal Activity

To biologically characterize the isolated triterpenoids for their trypanocidal activities, their cytotoxicities in RAW264.7 macrophages (mammalian host cells) and *T. cruzi* epimastigotes were analyzed. As presented in Table 7, eight of the compounds displayed relatively potent and selective trypanocidal activities, with good selectivity indices (>5).

**Table 7:** Compounds Showing Trypanocidal Effects on *Trypanosoma cruzi* with IC_50_ Values <5 μM and a Selectivity Index of 5

**Compound**	**RAW 264.7 IC**_**50**_**(μM)**	***T. cruzi*****epimastigote** WT-Y IC_**50**_**(μM)**	**Selectivity Index**
**1**	38.1	4.5	8.5
**2**	31.5	5	6.3
**3**	31	4	7.8
**5**	18.4	3.4	5.4
**7**	23.5	3.7	6.4
**8**	28.4	4.9	5.8
**12**	24.4	3.7	6.6
**20** + **21**	26.7	3.1	8.6

**Figure 3:**
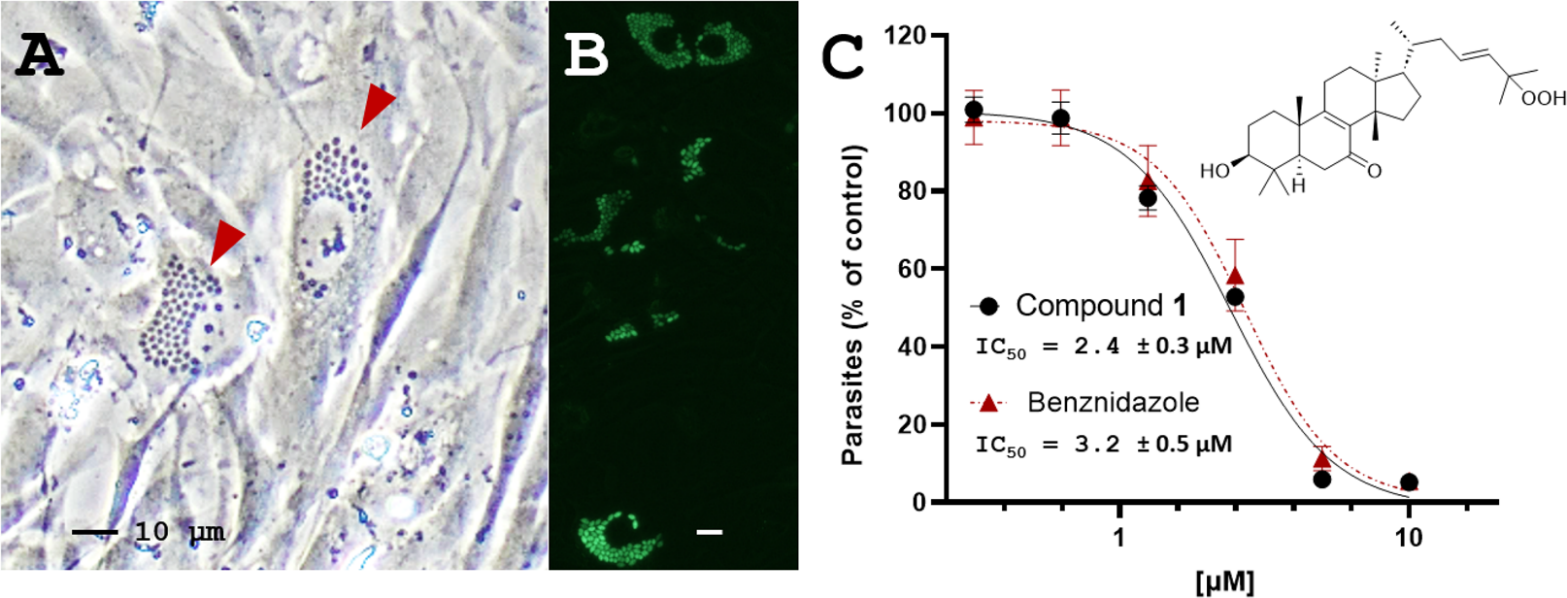
Inhibition activity of **1** against amastigote replication in the host cells. Compound **1** efficiently inhibited amastigote replication, thereby limiting the transmission of the parasite from infected cells, as observed with benznidazole. (A) Representative brightfield microscopic image of CCL39 fibroblasts infected with *Trypanosoma cruzi* with amastigotes (arrow). (B) Infected cells exhibiting the GFPY fluorescence signal of the analyzed *T. cruzi* strain. (C) Inhibition curves of compound **1** and the positive control, benznidazole, of the GFPY parasite release from the infected RAW264.7 cells were measured by fluorescence-activated cell sorting (FACS) after 6 days. The data show the mean values ± SD of three independent experiments (each performed in triplicates).

Interestingly, some compounds (**5**, **8**, and **12**) at the lower micromolar concentrations increased the proliferation of the epimastigotes by 15–30%; however, they became trypanocidal (inhibiting the epimastigotes) at higher concentrations (Supporting Information Figure S130). To determine the efficiencies of these compounds in a cellular infection system in which the amastigote stage was targeted (Figures 3A–B), desmondiin A (**1**) was analyzed for its effects against amastigotes, which displayed the best potency and selectivity toward the epimastigotes. As shown in Figure 3C, desmondiin A (**1**) efficiently and potently inhibited parasite replication in the host cells, as confirmed by the concentration-dependent reduction of parasite release after 6 days (see Experimental section), yielding an IC_50_ value of 2.4 ± 0.3 μM. Thus, desmondiin A (**1**) was as potent as the positive control, benznidazole, in the infection assay (IC_50_ = 3.2 ± 0.5 μM, with a robust 10-fold selective toxicity over the host cells). The structure–activity relationships of the desmondiins appeared complex and related to the absolute configuration of the side chain of the tetracyclic triterpenoid scaffold, combined with the functional group substitutions. Although studies have demonstrated the trypanocidal effects of triterpenoids on *T. cruzi*,^27^ their mode of action remains elusive. Desmondiin A (**1**) is currently among the most potent antichagasic triterpene when compared to previous reports on different triterpenoids,^28,29^ including ursolic acid and betulinic acid.^30−32^ Subsequent studies will focus on elucidating the action mechanism of these compounds, as well as the utilization of natural scaffolds to generate more potent antichagasic triterpenoids.

## Experimental Section

### General Experimental Procedures

The optical rotations were measured by using a Jasco P-2000 polarimeter (Jasco International Co. Ltd., Hachioji, Tokyo, Japan). NMR spectra were recorded in CDCl_3_ using a Bruker Avance DRX 500 spectrometer at 500 MHz (^1^H) and 125 MHz (^13^C). Signals of residue nondeuterated solvents were used as references. The HR-ESI-MS spectra were recorded on a Thermo Scientific Q-Exactive Plus Orbitrap mass spectrometer equipped with an ESI- or APCI-ion source in positive ionization mode. The MassLynx software was used for data acquisition and processing. To separate the extract and fractions, VLC was performed using silica gel (15 μm, Merck); LiChroprep RP-18 (40–63 μm, Merck) stationary phase was used for RP-VLC; OCC was performed with polyamide (MP Biomedicals). Further, flash chromatography (FC) was performed using a CombiFlash Rf+ Lumen instrument with integrated ultraviolet (UV), UV–visible (UV–Vis), and evaporative light scattering detection using an RP column (RediSep C_18_ Bulk 950, Teledyne Isco, Lincoln, NE, USA). PTLC was performed on silica gel 60 F_254_ plates (Merck). HPLC was performed using WUFENG, WATERS, and Agilent HPLC instruments equipped with LiChrospher Si 60 (4 mm × 250 mm, 5 μm) and Luna (R) Silica (2) 100 (250 mm × 21.2 mm, 5 μm), as well as RP Kinetex C_18_ 100A (4.6 mm × 150 mm, 5 μm) and Agilent ZORBAX ODS C_18_ 100A (9.4 mm × 250 mm, 5 μm) columns. TLC plates were detected under UV light at 254 nm and sprayed with concentrated sulfuric acid followed by heating for 5 min. Solvents employed for the extraction, OCC, VLC, FC, and PTLC were of analytical grade (VWR Ltd., Hungary).

### Plant Material

The aerial parts of *Euphorbia desmondii* Keay and Milne-Redh (Euphorbiaceae) were collected in June 2020 from the outskirts of Zaria City (Kaduna State, Nigeria). They were identified by Umar Shehu Gallah (National Research Institute for Chemical Technology, NARICT, Zaria, Nigeria). A voucher specimen was deposited in NARICT (number: Narict/Biores/322) and in the Herbarium of the Department of Pharmacognosy, University of Szeged (Szeged, Hungary; No. 898).

### Extraction and Isolation

The experiment aimed to isolate triterpenes, so we designed all separation steps adequate to triterpenes, and the progress of the compound isolation was monitored using TLC with conc. sulfuric acid detection. The air-dried powdered plant material (1950 g) was extracted with MeOH (63 L) via percolation at room temperature. Thereafter, the MeOH extract was concentrated in vacuo to yield a greenish brown oily material (207.4 g). This extract was dissolved in MeOH–H_2_O (1:1) and subjected to solvent–solvent partitioning using CHCl_3_ (4 × 1000 mL). The organic phase (109.8 g) was subjected to OCC on polyamide (320 g) using MeOH–H_2_O (1:4, 2:3, 3:2, 4:1, and 5:0) mixtures as eluents to yield 20% (5.5 g), 40% (11.4 g), 60% (22.4 g), 80% (14.2 g), and 100% (31.2 g) MeOH fractions, respectively. The 60% MeOH fraction displayed a unique TLC profile and was selected for further chromatographic separations. VLC was performed on the 60% MeOH fraction using silica gel and a gradient system comprising cyclohexane–EtOAc–EtOH (100:0:0, 90:10:0, 80:20:0, 70:30:0, 75:25:1, 75:25:2, 75:25:4, 75:25:6, 75:25:8, 75:25:10, 75:25:12, 75:25:15, and 75:25:20). The obtained fractions were analyzed by TLC, and those with similar profiles were combined, yielding combined fractions A–H. Fraction C (1.14 g) was separated by VLC on reversed-phase Si gel using MeOH–H_2_O mixtures (50:50, 60:40, 65:35, 70:30, 75:35, 80:20, 85:15, and 90:10) as eluents, thus affording fractions C/I–C/VII. Next, fraction C/VI was subjected to NP-VLC separation using a gradient system comprising petroleum ether–EtOAc as the mobile phase to afford subfractions C/IV/1–4. Furthermore, C/IV/3 was further separated by RP-HPLC using an isocratic system comprising MeCN–H_2_O (58:42) as the eluent to yield fractions C/IV/3/A–C. Additionally, the NP-HPLC purification of C/IV/3/C via isocratic elution with cyclohexane–EtOAc (65:35) resulted in the isolation of **12** (4.9 mg) and fraction C/IV/3/C/III; they were separated by PTLC on silica gel using CHCl_3_–acetone (9:1) as the developing system to isolate **2** (2.9 mg). Similarly, subfraction C/V was subjected to NP-VLC separation using a gradient system comprising petroleum ether–EtOAc as the eluent to yield fractions C/V/1–3. The RP-HPLC separation of C/V/3 using a gradient system comprising MeCN–H_2_O (from 85:15 to 100:0) yielded subfractions C/V/3/A–C. The two-step purification of C/V/3/B by NP-HPLC (gradient system: cyclohexane/EtOAc, from 70:30 to 65:35) and NP-PLC (isocratic eluent: CHCl_3_–acetone, 9:1) facilitated the isolation of **8** (2.0 mg). The NP-HPLC purification of fraction C/V/3/C using an isocratic system comprising cyclohexane/EtOAc (65:35) facilitated the isolation of **16** (4.3 mg), **17** (11.6 mg), and **18** (7.3 mg).

Fraction D (3.21 g) was separated by FC using MeOH–H_2_O mixtures (from 50:50 to 100:0) as eluents to afford subfractions D/I–XIV. The four-step purification of fraction D/VII by NP-VLC (gradient elution with petroleum ether–EtOAc mixtures), RP-HPLC [gradient system of MeCN–H_2_O (91:9–100:0)], NP-HPLC (gradient system: cyclohexane–EtOAc, from 55:45 to 52:48), and NP-PTLC (isocratic elution: CHCl_3_–acetone, 9:1) resulted in the isolation of **12** (4.5 mg). Conversely, the chromatographic separation of D/VIII by NP-VLC using a gradient system comprising petroleum ether–EtOAc as the eluent afforded fractions D/VIII/1–6. The RP-HPLC separation of D/VIII/2 using a gradient system comprising MeCN–H_2_O (96:4–100:0) afforded fractions D/VIII/2/A–D. The two-step purification of D/VIII/2/B by NP-HPLC (gradient system: cyclohexane–EtOAc, 60:40–55:45) and RP-HPLC (gradient system: MeOH–H_2_O, 89:11–91:9) resulted in the isolation of **22** (8.4 mg) and the mixture of **14** and **15** (2.3 mg). The two-step purification of D/VIII/2/C/III by NP-HPLC (gradient system: cyclohexane–EtOAc, 50:50–45:55) and NP-PLC (developing system: CHCl_3_–acetone, 87:13) resulted in the isolation of **3** (2.3 mg) and **4** (5.2 mg). The NP-HPLC separation of fraction D/VIII/2/C yielded D/VIII/2/C/I–III; the three fractions were purified by NP-PTLC using a mobile phase comprising CHCl_3_–acetone (87:13). Employing this approach, D/VIII/2/C/I, D/VIII/2/C/II, and D/VIII/2/C/III facilitated the isolation of **13** (2.3 mg), **19** (4.3 mg), and **1** (11.3 mg), respectively. Furthermore, fraction D/VIII/3 was subjected to RP-HPLC using an isocratic mobile system comprising MeCN–H_2_O (50:50), affording D/VIII/3/A–C. The NP-HPLC purification of D/VIII/3/A using a gradient system comprising cyclohexane–EtOAc (50:50–47:53) resulted in the isolation of **5** (2.5 mg). The further purification of D/VIII/3/C by NP-PTLC using a CHCl_3_–acetone (86:14) developing system resulted in the isolation of compound **23** (6.3 mg).

Additionally, fraction E (3.28 g) was subjected to FC using MeOH/H_2_O mixtures (from 55:45 to 100:0) as eluents. This separation afforded subfractions E/I–E/VI. The NP-VLC separation of E/IV using a gradient system comprising CHCl_3_–acetone (from 98:2 to 80:20) afforded E/IV/1–3. Thereafter, fraction E/IV/2 was subjected to RP-HPLC via gradient elution using MeCN–H_2_O mixtures (41:59–100:0) to yield E/IV/2/A–P. The two-step purification of E/IV/2/H by NP-HPLC (gradient system: cyclohexane–EtOAc, 50:50–45:55) and RP-HPLC (gradient system: MeOH–H_2_O, 88:12–92:8) resulted in the isolation of mixtures of **20** and **21** (2.2 mg). Similarly, the two-step purification of E/IV/2/P by NP-HPLC (gradient system: cyclohexane–EtOAc, 50:50–40:60) and RP-HPLC (gradient system: MeOH–H_2_O, 88:12–92:8) resulted in the isolation of **9** (2.3 mg) and **7** (1.9 mg). Further, the purification of fraction E/IV/3 by RP-HPLC (gradient system: MeCN–H_2_O, 71:29–78:22) and NP-HPLC (gradient system: cyclohexane–EtOAc, 50:50–30:70) resulted in the isolation of **6** (3.9 mg) and **10** (4.9 mg).

#### Desmondiin A (**1**)

White amorphous powder; [α]_D_^26^ – 7.9 (*c* 0.1, CHCl_3_); UV (MeOH) λ_max_ (log ε) = 257 (3.85) nm; ^1^H and ^13^C NMR data, see Tables 1 and 2; HR-ESI-MS + *m*/*z* 473.3637 [M + H]^+^ (calcd. for C_30_H_49_O_4_^+^: 473.3625).

#### Desmondiin C (**2**)

White amorphous powder; [α]_D_^27^ + 11.6 (*c* 0.1, CHCl_3_); UV (MeOH) λ_max_ (log ε) = 256 (3.80) nm; ^1^H and ^13^C NMR data, see Tables 1 and 2; HR-ESI-MS + *m*/*z* 457.3688 [M + H]^+^ (calcd. for C_30_H_49_O_3_^+^: 457.3676).

#### Desmondiin D (**3**)

White amorphous powder; [α]_D_^25^ – 6.3 (*c* 0.1, CHCl_3_); UV (MeOH) λ_max_ (log ε) = 258 (3.81) nm; ^1^H and ^13^C NMR data, see Tables 1 and 2; HR-ESI-MS + *m*/*z* 443.3527 [M + H]^+^ (calcd. for C_29_H_47_O_3_^+^: 443.3520).

#### Desmondiin E (**4**)

White amorphous powder; [α]_D_^30^ – 10.7 (*c* 0.1, CHCl_3_); UV (MeOH) λ_max_ (log ε) = 258 (3.80) nm; ^1^H and ^13^C NMR data, see Tables 1 and 2; HR-ESI-MS + *m*/*z* 457.3685 [M + H]^+^ (calcd. for C_30_H_49_O_3_^+^: 457.3676).

#### Desmondiin F (**5**)

White amorphous powder; [α]_D_^26^ + 9.1 (*c* 0.1, CHCl_3_); UV (MeOH) λ_max_ (log ε) = 257 (3.79) nm; HR-APCI-MS + *m*/*z* 505.3519 [M + H]^+^ (calcd. for C_30_H_49_O_6_^+^: 505.3524).

#### Desmondiin G (**6**)

White amorphous powder; [α]_D_^25^ + 17.5 (*c* 0.1, CHCl_3_); UV (MeOH) λ_max_ (log ε) = 258 (3.88) nm; ^1^H and ^13^C NMR data, see Tables 1 and 2; HR-APCI-MS + *m*/*z* 489.3568 [M + H]^+^ (calcd. for C_30_H_49_O_5_^+^: 489.3575) and 455.3512 [M – OOH]^+^ (calcd. for C_30_H_47_O_3_^+^: 455.3520).

#### Desmondiin H (**7**)

White amorphous powder; [α]_D_^25^ + 9.8 (*c* 0.05, CHCl_3_); UV (MeOH) λ_max_ (log ε) = 256 (3.82) nm; ^1^H and ^13^C NMR data, see Tables 1 and 2; HR-APCI-MS + *m*/*z* 471.3465 [M + H]^+^ (calcd. for C_30_H_47_O_4_: 471.3469).

#### Desmondiin I (**8**)

White amorphous powder; [α]_D_^25^ – 11.5 (*c* 0.1, CHCl_3_); UV (MeOH) λ_max_ (log ε) = 254 (3.80) nm; ^1^H and ^13^C NMR data, see Tables 3 and 4; HR-ESI-MS + *m*/*z* 457.3687 [M + H]^+^ (calcd. for C_30_H_49_O_3_^+^: 457.3676).

#### Desmondiin J (**9**)

White amorphous powder; [α]_D_^25^ – 8.4 (*c* 0.05, CHCl_3_); UV (MeOH) λ_max_ (log ε) = 254 (3.78) nm; ^1^H and ^13^C NMR data, see Tables 3 and 4; HR-APCI-MS + *m*/*z* 473.3620 [M + H]^+^ (calcd. for C_30_H_49_O_4_^+^ 473.3625).

#### Desmondiin K (**10**)

White amorphous powder; [α]_D_^25^ + 6.3 (*c* 0.1, CHCl_3_); UV (MeOH) λ_max_ (log ε) 253 (3.77) nm; ^1^H and ^13^C NMR data, see Tables 3 and 4; HR-APCI-MS + *m*/*z* 473.3620 [M + H]^+^ (calcd. for C_30_H_49_O_4_^+^: 473.3625) and 455.3513 [M + H – H_2_O]^+^ (calcd. for C_30_H_47_O_3_^+^: 455.3520).

#### Desmondiin L (**11**)

White amorphous powder; [α]_D_^27^ + 29.8 (*c* 0.1, CHCl_3_); UV (MeOH) λ_max_ (log ε) = 270 (3.92) nm; ^1^H and ^13^C NMR data, see Tables 5 and 6; HR-APCI-MS + *m*/*z* 485.3618 [M + H]^+^ (calcd. for C_31_H_49_O_4_^+^: 485.3625), 471.3462 [M + H – CH_2_]^+^ (calcd. for C_30_H_47_O_4_^+^: 471.3470), and 453.3359 [M + H – CH_3_OH]^+^ (calcd. for C_30_H_45_O_3_^+^: 453.3363).

#### Desmondiin M (**12**)

White amorphous powder; [α]_D_^26^ + 26.3 (*c* 0.05, CHCl_3_); UV (MeOH) λ_max_ (log ε) = 272 (3.95) nm; ^1^H and ^13^C NMR data, see Tables 5 and 6; HR-ESI-MS + *m*/*z* 457.3325 (calcd. for C_29_H_44_O_4_^+^: 457.3312).

#### Desmondiin N (**13**)

White amorphous powder; [α]_D_^26^ + 14.2 (*c* 0.1, CHCl_3_); UV (MeOH) λ_max_ (log ε) = 219 (3.90), 269 (3.92) nm; ^1^H and ^13^C NMR data, see Tables 5 and 6; HR-ESI-MS *m*/*z* 469.3308 (calcd. for C_30_H_45_O_4_^+^: 469.3312).

#### Desmondiins O + P (**14** + **15**)

White amorphous solid; UV (MeOH) λ_max_ (log ε) = 271 (3.85) nm; ^1^H and ^13^C NMR data, see Tables 5 and 6; HR-ESI-MS *m*/*z* 487.3430 [M + H]^+^ (calcd. for C_30_H_47_O_5_^+^: 487.3418).

### Biological Assays

Epimastigotes of *T. cruzi* [Y strain (ATCC 50832)] were maintained in weekly passages in a liver infusion tryptose (LIT) medium supplemented with 10% heat-inactivated fetal bovine serum (hiFBS) at 28 °C, as described previously.^33^ The epimastigote cultures in the logarithmic-growth phase (1–5 × 10^7^ parasites/mL) were used for the experiments unless stated otherwise. The green fluorescent protein (GFP)-overexpressing epimastigotes were generated by transfection using pTREX-n-eGFP, a gift from Rick Tarleton (Addgene plasmid # 62544; http://n2t.net/addgene:62544;RRID:Addgene_62544), as described previously. The GFP-expressing epimastigotes were cultured in LIT medium supplemented with 10% hiFBS and 500 μg/mL G418.

According to the Drugs for Neglected Diseases initiative (DNDi), an ideal lead compound must exhibit activity against trypanosomes at concentrations of less than or equal to 10 μM as well as exhibit a 10-fold-greater potency toward *T. cruzi* over mammalian cells (SI).^33^ To assess the selectivities of the isolated compounds, their trypanocidal activities against the epimastigotes were assessed by the Cell Proliferation Kit II (XTT) assay, as previously described.^11^ Briefly, 1.5 × 10^5^ epimastigotes were seeded per well in 96-well plates. The compounds were added at a single concentration, 10 μM (for the initial screening experiments) or six concentrations ranging from 10–0.3 μM for 72 h at 28 °C. In all the experiments, benznidazole was used as the positive control. Following the incubation period, the plates were examined under a microscope for sterility and growth of controls. To the plates, 50 μL of XTT and PMS (Phenazine methosulfate, Sigma-Aldrich, MO, USA) solution (XTT and PMS at 0.5 and 0.025 mg/mL, respectively) was added, and the plates were then incubated at 28 °C for 2.5 h. The parasites were fixed by the addition of 50 μL of MeOH for 15 min before the absorbance was measured at 490 nm on a Tecan plate reader. Results were expressed as percentage cell viability relative to vehicle control or IC_50_ values calculated by GraphPad Prism version 8.0. All assessments were performed in triplicate in at least two independent experiments.

Fluorescence-activated cell sorting (FACS)-based quantitation of released parasites from infected RAW264.7 cells: The host cells were seeded in 24-well plates at densities of 40,000 and 10,000 cells/mL. The cells were allowed to adhere for 24 h, after which they were infected with trypomastigotes that were harvested by the swim-out procedure described above at a multiplicity of 10. On the next day, the noninternalized trypomastigotes were washed off, and fresh RPMI with 2% fetal bovine serum was added to the wells along with the test substance. In all of the experiments, the screening was performed in triplicate, including tests with DMSO and benznidazole controls. The released parasites were fixed using 4% paraformaldehyde in phosphate-buffered saline for 1 h, after which the samples were analyzed by FACS (BD Biosciences), as described previously.^11^

### Microscopy

The host CCL39 cells were seeded in 24-well plates at a density of 20,000 cells/mL. The cells were allowed to adhere for 24 h, after which they were infected with trypomastigotes at a multiplicity of 10. On the next day, the noninternalized trypomastigotes were washed off, and fresh RPMI with 2% hiFBS was added to the wells along with the test substance. On the fourth day, following infection, the cells were fixed with 4% paraformaldehyde for 1 h and scanned using a Nikon Eclipse TS2 microscope. Thereafter, the images were analyzed on ImageJ.

## Data Availability

The NMR data for **1**–**16** have been deposited in the Natural Products Magnetic Resonance Database (NP-MRD; www.np-mrd.org) and can be found at NP0333436 (**1**), NP0333438 (**2**), NP0333439 (**3**), NP0333440 (**4**), NP0333441 (**5**), NP0333442 (**6**), NP0333443 (**7**), NP0333444 (**8**), NP0333445 (**9**), NP0333446 (**10**), NP0333447 (**11**), NP0333448 (**12**), NP0333449 (**13**), NP0333450 (**14**), NP0333451 (**15**), and NP0333451 (**16**).

## Supporting Information Available

UV, HR-ESI-MS, and NMR spectra of the isolated compounds; data of antiproliferative assay on *T. cruzi* epimastigoge (PDF)

## References

[ref1] Coura J. R.; Viñas P. A.; Junqueira A. C. Mem. Inst. Oswaldo Cruz. 2014, 109, 856–62. 10.1590/0074-0276140236. 25410988 PMC4296489

[ref2] https://www.who.int/news-room/fact-sheets/detail/chagas-disease-(american-trypanosomiasis) retrived on 29 05 2024.

[ref3] Hemmige V.; Tanowitz H.; Sethi A. Int. J. Dermatol. 2012, 51, 501–508. 10.1111/j.1365-4632.2011.05380.x. 22515575 PMC3552304

[ref4] Lopez-Albizu C.; Rivero R.; Ballering G.; Freilij H.; Santini M. S.; Bisio M. M. C. Front. Parasitol. 2023, 2, 138375 10.3389/fpara.2023.1138375. PMC11732150 39816836

[ref5] Kemmerling U.; Bosco C.; Galanti N. Biol. Res. 2010, 43, 307–316. 10.4067/S0716-97602010000300007. 21249302

[ref6] Cortes V.; Cruz A.; Onetti S.; Kinzel D.; Garcia J.; Ortiz S.; Lopez A.; Cattan P. E.; Botto-Mahan C.; Solari A. PLoS Negl. Trop. Dis. 2021, 15, e0009729 10.1371/journal.pntd.0009729. 34543275 PMC8452000

[ref7] Meymandi S.; Hernandez S.; Park S.; Sanchez D. R.; Forsyth C. Curr. Treat. Options Infect. Dis. 2018, 10, 373–388. 10.1007/s40506-018-0170-z. 30220883 PMC6132494

[ref8] Lazarin-Bidóia D.; Garcia F. P.; Ueda-Nakamura T.; Silva S. O.; Nakamura C. V. Mem. Inst. Oswaldo Cruz. 2022, 117, e220396 10.1590/0074-02760220396. 35352776 PMC8970591

[ref9] García-Huertas P.; Cardona-Castro N. Biomed. Pharmacother. 2021, 142, 112020 10.1016/j.biopha.2021.112020. 34392087

[ref10] Izumi E.; Ueda-Nakamura T.; Dias Filho B. P.; Veiga Junior V. F.; Nakamura C. V. Nat. Prod. Rep. 2011, 28, 809–823. 10.1039/c0np00069h. 21290079

[ref11] Salm A.; Krishnan S. R.; Collu M.; Danton O.; Hamburger M.; Leonti M.; Almanza G.; Gertsch J. iScience 2021, 24, 102310 10.1016/j.isci.2021.102310. 33870129 PMC8040286

[ref12] Govaerts R.; Frodin D. G.; Radcliffe-Smith A. World Checklist and Bibliography of Euphorbiaceae (and Pandaceae) Vol. 4; Royal Botanic Gardens: Kew, 2000, pp 1–1622.

[ref13] Flora of West Tropical Africa. In Kew Bulletin, Vol 139, 1955. Retrieved from https://powo.science.kew.org/taxon/urn:lsid:ipni.org:names:346272-1#distributions on 4th January 2024.

[ref14] Burkill H. M. The useful plants of West Tropical Africa, Vol 2, 1985. Retrieved from https://plants.jstor.org/search?Date_fld=1968 on 4th January, 2024.

[ref15] Abo K. A. Afr. J. Med. Med. Sci. 1994, 23, 161–163. 7625305

[ref16] Hammadi R.; Kúsz N.; Mwangi P. W.; Kulmány Á.; Zupkó I.; Orvos P.; Tálosi L.; Hohmann J.; Vasas A. Nat. Prod. Commun. 2019, 14, 1–5. 10.1177/1934578X19863509.

[ref17] Xu W.; Zhu C.; Cheng W.; Fan X.; Chen X.; Yang S.; Guo Y.; Ye F.; Shi J. J. Nat. Prod. 2009, 72, 1620–1626. 10.1021/np900305j. 19702283

[ref18] Pettit G. R.; Ye Q.; Herald D. L.; Knight J. C.; Hogan F.; Melody N.; Mukku V. J.; Doubek D. L.; Chapuis J. C. Isolation and structure of cancer cell growth inhibitory tetracyclic triterpenoids from the Zimbabwean *Monadenium lugardae*. J. Nat. Prod. 2016, 79, 1598–1603. 10.1021/acs.jnatprod.6b00107. 27214528

[ref19] Jiang Z. H.; Tanaka T.; Hirata H.; Fukuoka R.; Kouno I. Tetrahedron 1997, 53, 16999–17008. 10.1016/S0040-4020(97)10150-8.

[ref20] Xu J.; Xiao D.; Lin Q. H.; He J. F.; Liu W. Y.; Xie N.; Feng F.; Qu W. J. Nat. Prod. 2016, 79, 1899–1910. 10.1021/acs.jnatprod.5b01137. 27494664

[ref21] Wang L. Y.; Wang N. L.; Yao X. S.; Miyata S.; Kitanaka S. J. Nat. Prod. 2003, 66, 630–633. 10.1021/np0205396. 12762796

[ref22] Gao J.; Aisa H. A. J. Nat. Prod. 2017, 80, 1767–1775. 10.1021/acs.jnatprod.6b01099. 28590124

[ref23] Chang S. S.; Huang H.-T.; Wei W.-C.; Lo I.-W.; Lin Y.-C.; Chao C.-H.; Liao G.-Y.; Shen Y.-C.; Chen J.-J.; Li T.-L.; Lin L.-T.; Tai C.-J.; Kuo Y.-H.; Liaw C.-C. Front. Chem. 2023, 11, 223335 10.3389/fchem.2023.1223335. PMC10326319 37426336

[ref24] Wang S.; Liang H.; Zhao Y.; Wang G.; Yao H.; Kasimu R.; Wu Z.; Li Y.; Huang J.; Wang J. Fitoterapia 2016, 108, 33–40. 10.1016/j.fitote.2015.11.009. 26586618

[ref25] Fang C.-H.; Li Y.-P.; Li Y.; Yue J.-M.; Bao J.; Yu J.-H. Phytochemistry 2023, 211, 113684 10.1016/j.phytochem.2023.113684. 37105350

[ref26] Lu Z. Q.; Chen G. T.; Zhang J. Q.; Huang H. L.; Guan S. H.; Guo D. A. Helv. Chim. Acta 2007, 90, 2245–2250. 10.1002/hlca.200790233.

[ref27] Durão R.; Ramalhete C.; Madureira A. M.; Mendes E.; Duarte N. Pharmaceuticals 2022, 15, 340 10.3390/ph15030340. 35337138 PMC8951850

[ref28] de Araujo J. I. F.; Aires N. L.; Almeida-Neto F. W. Q.; Marinho M. M.; Marinho E. M.; Paula Magalhaes E.; de Menezes R. R. P. P. B.; Sampaio T. L.; Maria Costa Martins A.; Teixeira E. H.; et al. J. Biomol. Struct. Dyn. 2022, 40, 12302–12315. 10.1080/07391102.2021.1970025. 34436980

[ref29] Meira C. S.; Barbosa-Filho J. M.; Lanfredi-Rangel A.; Guimarães E. T.; Moreira D. R.; Soares M. B. Exp. Parasitol. 2016, 166, 108–115. 10.1016/j.exppara.2016.04.007. 27080160

[ref30] Vanrell M. C.; Martinez S. J.; Muñoz L. I.; Salassa B. N.; Gambarte Tudela J.; Romano P. S. Front. Cell. Infect. Microbiol. 2022, 12, 919096 10.3389/fcimb.2022.919096. 36004334 PMC9394444

[ref31] Leite A. C.; Ambrozin A. R.; Fernandes J. B.; Vieira P. C.; da Silva M. F.; de Albuquerque S. Planta Med. 2008, 74, 1795–1799. 10.1055/s-0028-1088323. 18991203

[ref32] Mazoir N.; Benharref A.; Bailén M.; Reina M.; González-Coloma A.; Martínez-Díaz R. A. Z. Naturforsch. C J. Biosci. 2011, 66, 360–366. 10.1515/znc-2011-7-807. 21950160

[ref33] Ioset J. R.; Brun R.; Wenzler T.; Kaiser M.; Yardley V. Drug Screening for Kinetoplastid Diseases: A Training Manual for Screening in Neglected Diseases; DNDi Pan-Asian Screening Network, April 2009.

