# Supplementary material for: Euphane and Tirucallane Triterpenes with Trypanocidal Activity from Euphorbia desmondii
Source: J Nat Prod. 2024 Sep 14;87(9):2281–91. doi: 10.1021/acs.jnatprod.4c00730 (PMC11443485; doi:10.1021/acs.jnatprod.4c00730)
Supplement: Supplementary file 1 — np4c00730_si_001.pdf [file np4c00730_si_001.pdf]

SUPPORTING INFORMATION

New Euphane and Tirucallane Triterpenes from *Euphorbia desmondii* with Trypanocidal Activity

Muhammad Bello Saidu,[1] Gordana Krstić,[1,2] Anita Barta,[1] Attila Hunyadi,[1] Róbert Berkecz,[3] Umar Shehu Gallah,[4] Kaushavi Cholke,[5] Jürg Gertsch,[5] Dóra Rédei,[1*] Judit Hohmann[1,6,]*

[1] Department of Pharmacognosy, University of Szeged, Eötvös u. 6, 6720 Szeged, Hungary

[2] University of Belgrade, Faculty of Chemistry, Studentski trg 12-16, 11158 Belgrade, Serbia

[3] Institute of Pharmaceutical Analysis, University of Szeged, Somogyi u. 4, 6720 Szeged, Hungary

[4] Bioresource Department, National Research Institute for Chemical Technology (NARICT), Zaria, Nigeria

[5] Institute of Biochemistry and Molecular Medicine, University of Bern, Bühlstrasse 28, 3012 Bern, Switzerland

[6] HUN-REN–USZ Biologically Active Natural Products Research Group, University of Szeged, Eötvös u. 6, H-6720 Szeged, Hungary

Content

Figure S1. UV spectrum of compound **1** 6

Figure S2. HRESIMS spectrum of compound **1** 6

Figure S3. ^{1}H NMR spectrum compound **1** (500 MHz, CDCl3) 7

Figure S4. ^{13}C NMR JMOD spectrum compound **1** (125 MHz, CDCl3) 7

Figure S5. HSQC spectrum compound **1** 8

Figure S6. HMBC spectrum compound **1** 8

Figure S7. ^{1}H-^{1}H COSY spectrum compound **1** 9

Figure S8. NOESY spectrum of compound **1** 9

Figure S9. UV spectrum of compound **2** 10

Figure S10. HRESIMS spectrum of compound **2** 10

Figure S11. ^{1}H NMR spectrum compound **2** (500 MHz, $CDCl_3$) 11

Figure S12. ^{13}C NMR JMOD spectrum compound **2** (125 MHz, $CDCl_3$) 11

Figure S13. HSQC spectrum compound **2** 12

Figure S14. HMBC spectrum compound **2** 12

Figure S15. ^{1}H-^{1}H COSY spectrum compound **2** 13

Figure S16. NOESY spectrum of compound **2** 13

Figure S17. UV spectrum of compound **3** 14

Figure S18. ESIMS spectrum of compound **3** 14

Figure S19. ^{1}H NMR spectrum compound **3** (500 MHz, $CDCl_3$) 15

Figure S20. ^{13}C NMR JMOD spectrum compound **3** (125 MHz, $CDCl_3$) 15

Figure S21. HSQC spectrum compound **3** 16

Figure S22. HMBC spectrum compound **3** 16

Figure S23. ^{1}H-^{1}H COSY spectrum compound **3** 17

Figure S24. NOESY spectrum of compound **3** 17

Figure S25. UV spectrum of compound **4** 18

Figure S26. HRESIMS spectrum of **4** 18

Figure S27. ^{1}H NMR spectrum of compound **4** (500 MHz, $CDCl_3$) 19

Figure S28. ^{13}C NMR JMOD spectrum of compound **4** (125 MHz, $CDCl_3$) 19

Figure S29. HSQC spectrum of compound **4** 20

Figure S30. HMBC spectrum of compound **4** 20

Figure S31. ^{1}H-^{1}H COSY spectrum of compound **4** 21

Figure S32. NOESY spectrum of compound **4** 21

Figure S33. UV spectrum of compound **5** 22

Figure S34. HR-APCI-MS spectrum of compound **5** 22

Figure S35. ^{1}H NMR spectrum compound **5** (500 MHz, $CDCl_3$) 23

Figure S36. ^{13}C NMR JMOD spectrum compound **5** (125 MHz, $CDCl_3$) 23
Figure S37. HSQC spectrum compound **5** 24
Figure S38. HMBC spectrum compound **5** 24
Figure S39. ^{1}H-^{1}H COSY spectrum compound **5** 25
Figure S40. NOESY spectrum of compound **5** 25
Figure S41. UV spectrum of compound **6** 26
Figure S42. HR-APCI-MS spectrum of compound **6** 26
Figure S43. ^{1}H NMR spectrum of compound **6** (500 MHz, $CDCl_3$) 27
Figure S44. ^{13}C NMR JMOD spectrum of compound **6** (125 MHz, $CDCl_3$) 27
Figure S45. HSQC spectrum of compound **6** 28
Figure S46. HMBC spectrum of compound **6** 28
Figure S47. ^{1}H-^{1}H COSY spectrum of compound **6** 29
Figure S48. NOESY spectrum of compound **6** 29
Figure S49 UV spectrum of compound **7** 30
Figure S50. HR-APCI-MS spectrum of compound **7** 30
Figure S51. ^{1}H NMR spectrum of compound **7** (500 MHz, $CDCl_3$) 31
Figure S52. ^{13}C NMR JMOD spectrum of compound **7** (125 MHz, $CDCl_3$) 31
Figure S53. HSQC spectrum of compound **7** 32
Figure S54. HMBC spectrum of compound **7** 32
Figure S55. ^{1}H-^{1}H COSY spectrum of compound **7** 33
Figure S56. NOESY spectrum of compound **7** 33
Figure S57. UV spectrum of compound **8** 34
Figure S58. HRESIMS spectrum of compound **8** 34
Figure S59. ^{1}H NMR spectrum of compound **8** (500 MHz, $CDCl_3$) 35
Figure S60. ^{13}C NMR JMOD spectrum of compound **8** (125 MHz, $CDCl_3$) 35
Figure S61. HSQC spectrum of compound **8** 36
Figure S62. HMBC spectrum of compound **9** 36
Figure S63. ^{1}H-^{1}H COSY spectrum of compound **8** 37
Figure S64. NOESY spectrum of compound **8** 37
Figure S65. UV spectrum of compound **9** 38
Figure S66. HRESIMS spectrum of compound **9** 38
Figure S67. ^{1}H NMR spectrum of compound **9** (500 MHz, $CDCl_3$) 39
Figure S68. ^{13}C NMR JMOD spectrum of compound **9** (125 MHz, $CDCl_3$) 39
Figure S69. HSQC spectrum of compound **9** 40
Figure S70. HMBC spectrum of compound **9** 40
Figure S71. ^{1}H-^{1}H COSY spectrum of compound **9** 41

Figure S72. NOESY spectrum of compound **9** 41
Figure S73. UV spectrum of compound **10** 42
Figure S74. HR-APCI-MS spectrum of compound **10** 42
Figure S75. ^{1}H NMR spectrum of compound **10** (500 MHz, $CDCl_3$) 43
Figure S76. ^{13}C NMR JMOD spectrum of compound **10** (125 MHz, $CDCl_3$) 43
Figure S77. HSQC spectrum of compound **10** 44
Figure S78. HMBC spectrum of compound **10** 44
Figure S79. ^{1}H-^{1}H COSY spectrum of compound **10** 45
Figure S80. NOESY spectrum of compound **10** 45
Figure S81. UV spectrum of compound **11** 46
Figure S82. HR-APCI-MS spectrum of compound **11** 46
Figure S83. ^{1}H NMR spectrum of compound **11** (500 MHz, $CDCl_3$) 47
Figure S84. ^{13}C NMR JMOD spectrum of compound **11** (125 MHz, $CDCl_3$) 47
Figure S85. HSQC spectrum of compound **11** 48
Figure S86. HMBC spectrum of compound **11** 48
Figure S87. ^{1}H-^{1}H COSY spectrum of compound **11** 49
Figure S88. NOESY spectrum of compound **11** 49
Figure S89. UV spectrum of compound **12** 50
Figure S90. HRESIMS of compound **12** 50
Figure S91. ^{1}H NMR spectrum of compound **12** (500 MHz, $CDCl_3$) 51
Figure S92. ^{13}C NMR JMOD spectrum of compound **12** (125 MHz, $CDCl_3$) 51
Figure S93. HSQC spectrum of compound **12** 52
Figure S94. HMBC spectrum of compound **12** 52
Figure S95. ^{1}H-^{1}H COSY spectrum of compound **12** 53
Figure S96. NOESY spectrum of compound **12** 53
Figure S97. UV spectrum of compound **13** 54
Figure S98. HRESIMS spectrum of compound **13** 54
Figure S99. ^{1}H NMR spectrum of compound **13** (500 MHz, $CDCl_3$) 55
Figure S100. ^{13}C NMR JMOD spectrum of compound **13** (125 MHz, $CDCl_3$) 55
Figure S101. HSQC spectrum of compound **13** 56
Figure S102. HMBC spectrum of compound **13** 56
Figure S103. ^{1}H-^{1}H COSY spectrum of compound **13** 57
Figure S104. UV spectrum of compound **14 +15** 57
Figure S105. HRESIMS spectrum of compound **14** + **15** 58
Figure S106. ^{1}H NMR spectrum of compound **14** + **15** (500 MHz, $CDCl_3$) 58
Figure S107. ^{13}C NMR JMOD spectrum of compound **14** + **15** (125 MHz, $CDCl_3$) 59

Figure S108. HSQC spectrum of compound **14** + **15** 59
Figure S109. HMBC spectrum of compound **14** + **15** 60
Figure S110. ^{1}H-^{1}H COSY spectrum of compound **14** + **15** 60
Figure S111. NOESY spectrum of compound **14** + **15** 61
Figure S112. UV spectrum of compound **16** 61
Figure S113. ^{1}H NMR spectrum compound **16** (500 MHz, $CDCl_3$) 62
Figure S114. ^{13}C NMR JMOD spectrum compound **16** (125 MHz, $CDCl_3$) 62
Figure S115. ^{1}UV spectrum of compound **17** 63
Figure S116. ^{1}H NMR spectrum of compound **17** (500 MHz, $CDCl_3$) 63
Figure S117. ^{13}C NMR JMOD spectrum of compound **17** (125 MHz, $CDCl_3$) 64
Figure S118. UV spectrum of compound **18** 64
Figure S119. ^{1}H NMR spectrum of compound **18** (500 MHz, $CDCl_3$) 65
Figure S120. ^{13}C NMR JMOD spectrum of compound **18** (125 MHz, $CDCl_3$) 65
Figure S121. ^{1}H NMR spectrum of compound **19** (500 MHz, $CDCl_3$) 66
Figure S122. ^{1}H NMR spectrum of compound **19** (500 MHz, $CDCl_3$) 66
Figure S123. ^{13}C NMR JMOD spectrum of compound **19** (125 MHz, $CDCl_3$) 67
Figure S124. UV spectrum of compound **20** + **21** 67
Figure S125. ^{1}H NMR spectrum of compound **20** + **21** (500 MHz, $CDCl_3$) 68
Figure S126. ^{13}C NMR JMOD spectrum of compound **20** + **21** (125 MHz, $CDCl_3$) 68
Figure S127. ^{1}H NMR spectrum of compound **22** 69
Figure S128. ^{1}H NMR spectrum of compound **22** (500 MHz, $CDCl_3$) 69
Figure S129. ^{13}C NMR JMOD spectrum of compound **22** (125 MHz, $CDCl_3$) 70
Figure S130. Compounds **2**, **5**, **8**, and **12** induced a significant increase in *T. cruzi* epimastigoge proliferation. Experiments show mean values ± SD of three independent experiments each performed in triplicates 70

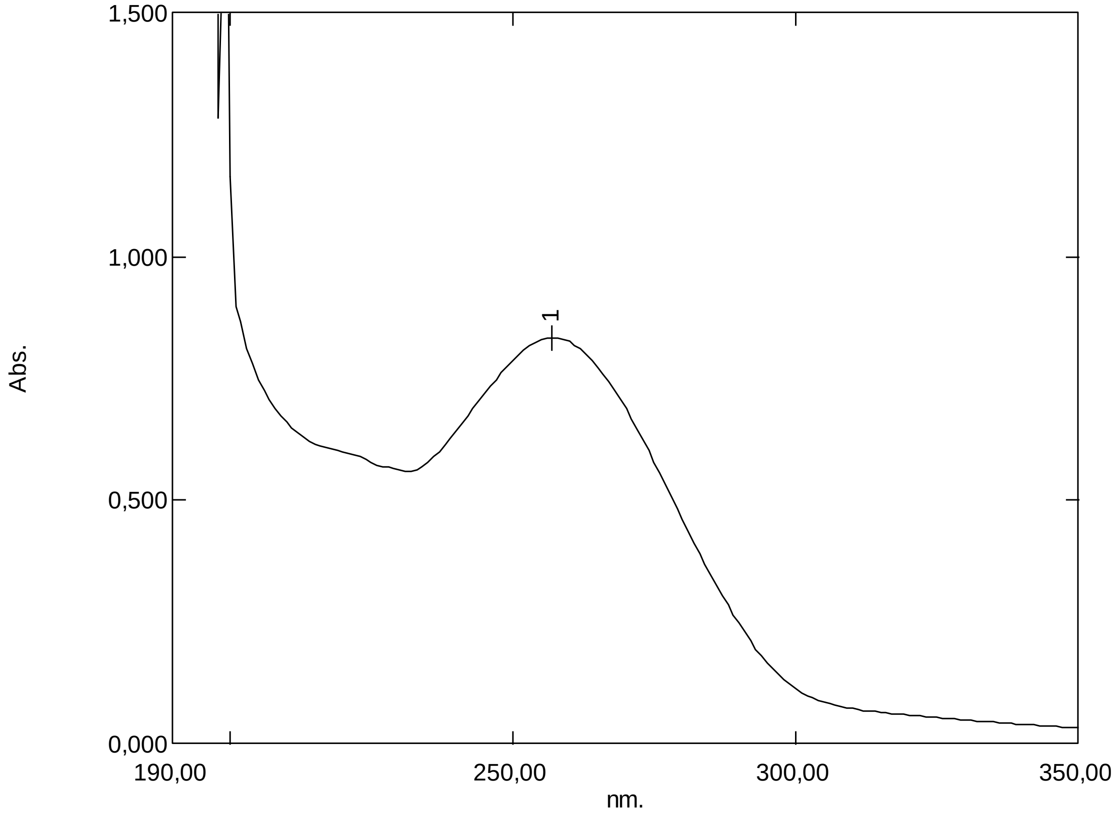

Figure S1. UV spectrum of compound **1**

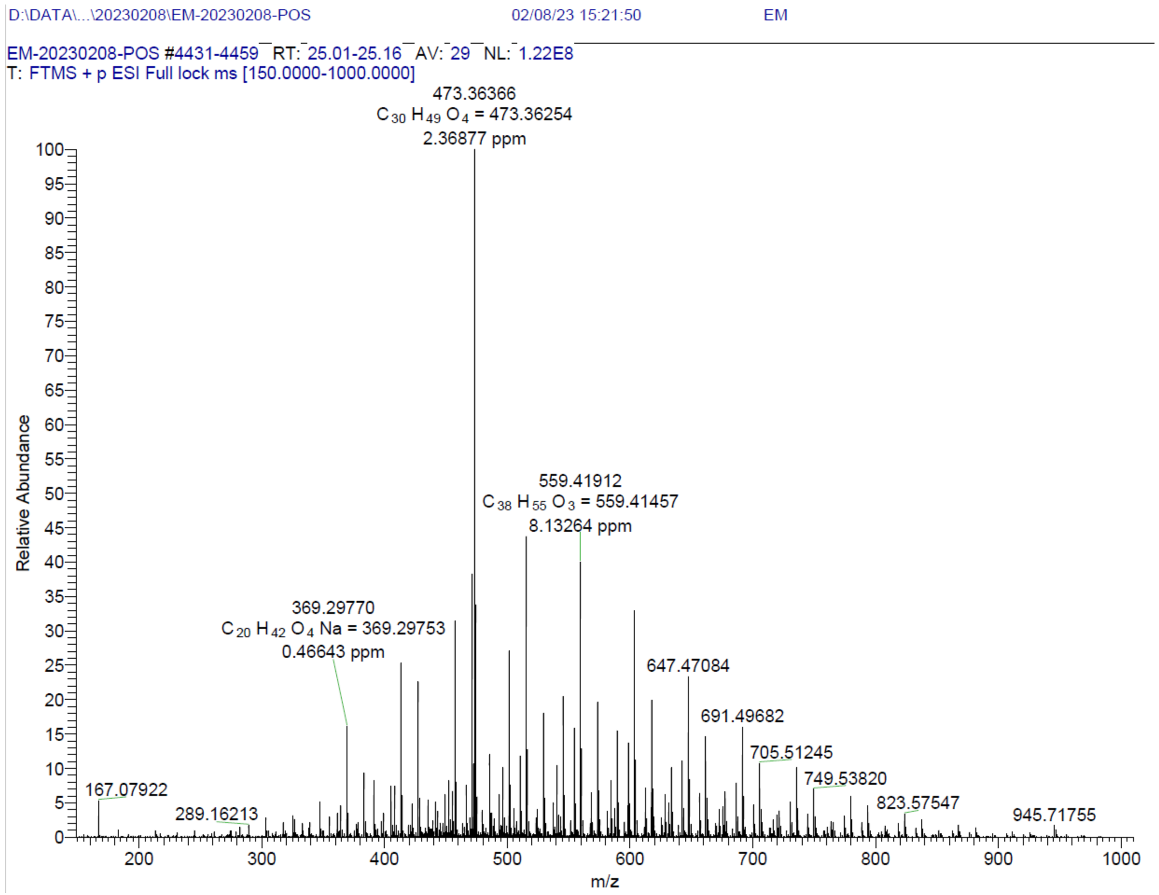

Figure S2. HRESIMS spectrum of compound **1**

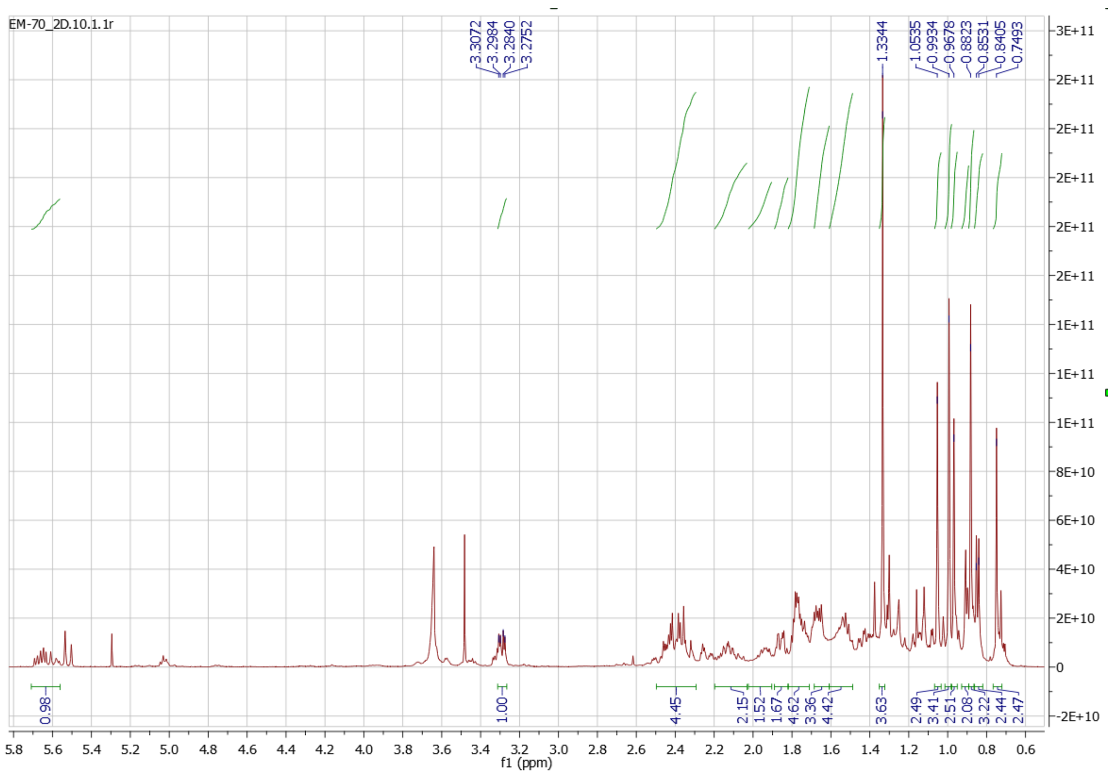

Figure S3. ^{1}H NMR spectrum compound **1** (500 MHz, CDCl3)

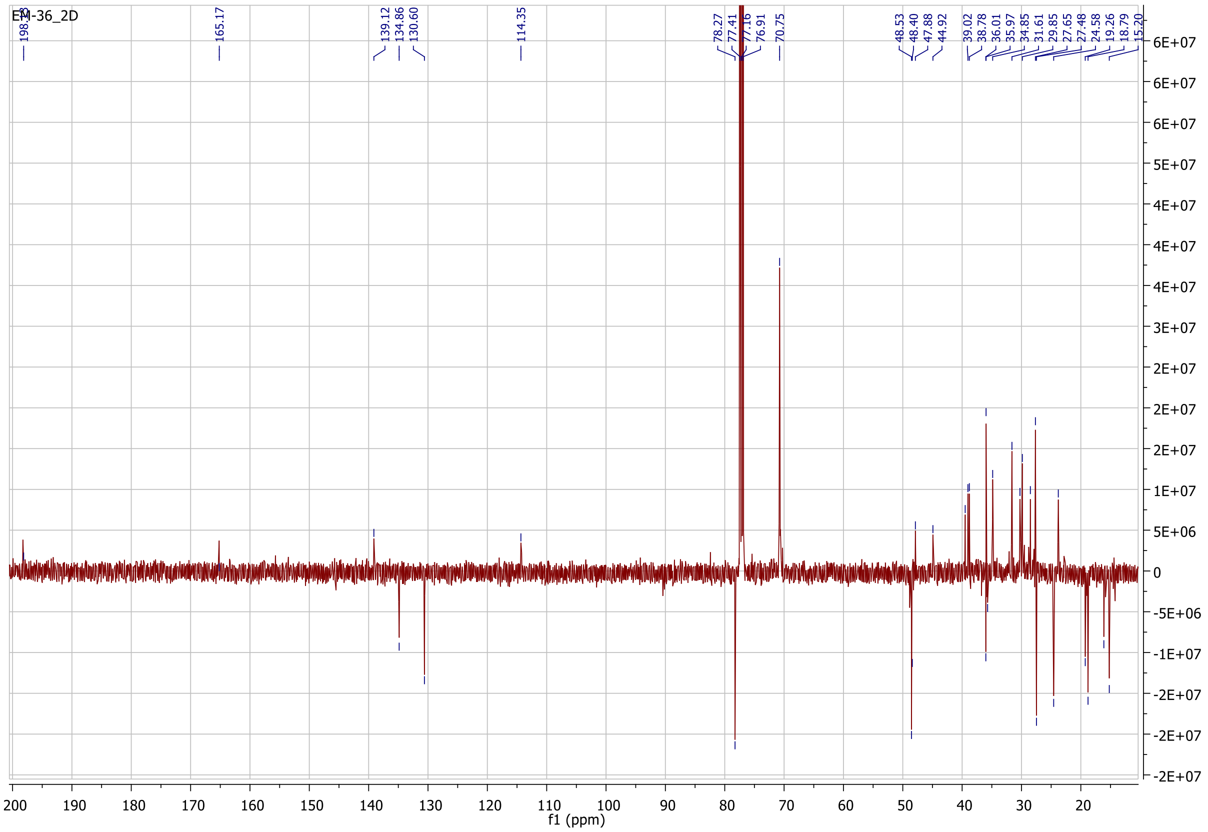

Figure S4. ^{13}C NMR JMOD spectrum compound **1** (125 MHz, CDCl3)

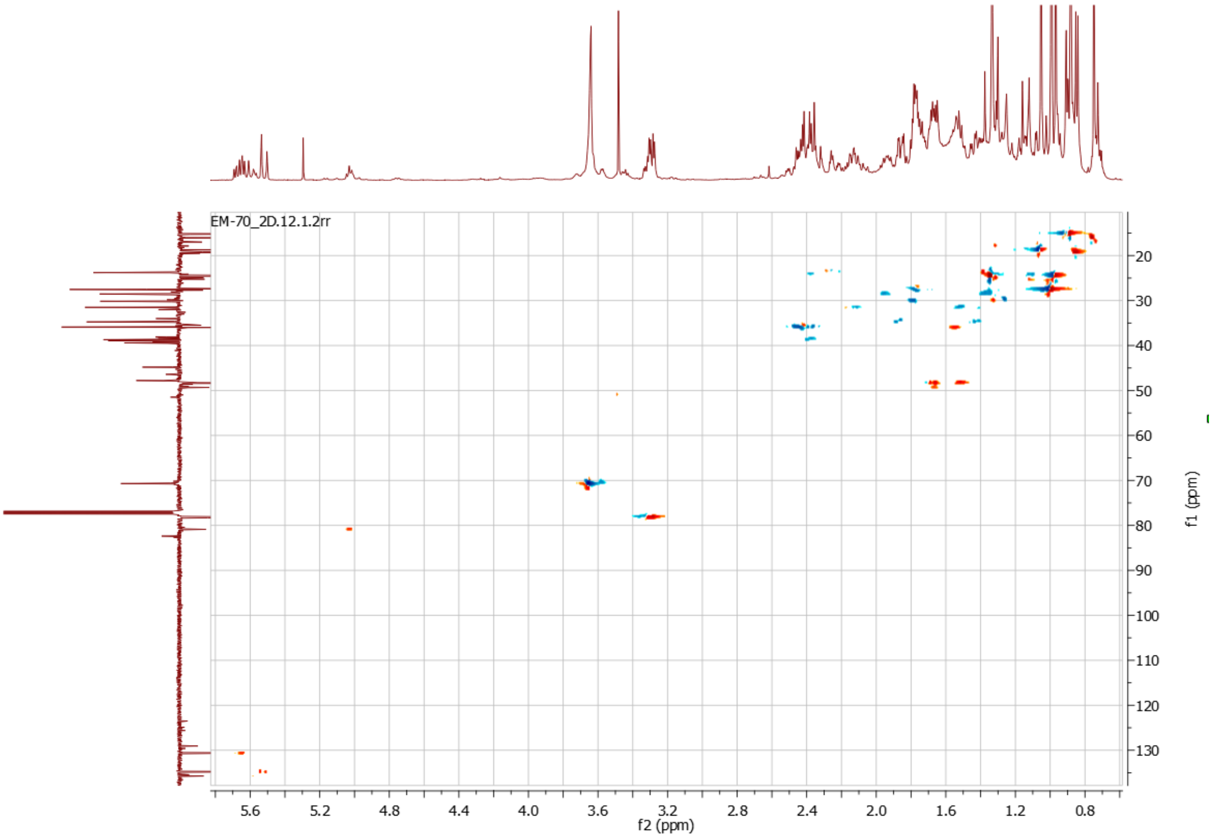

Figure S5. HSQC spectrum compound **1**

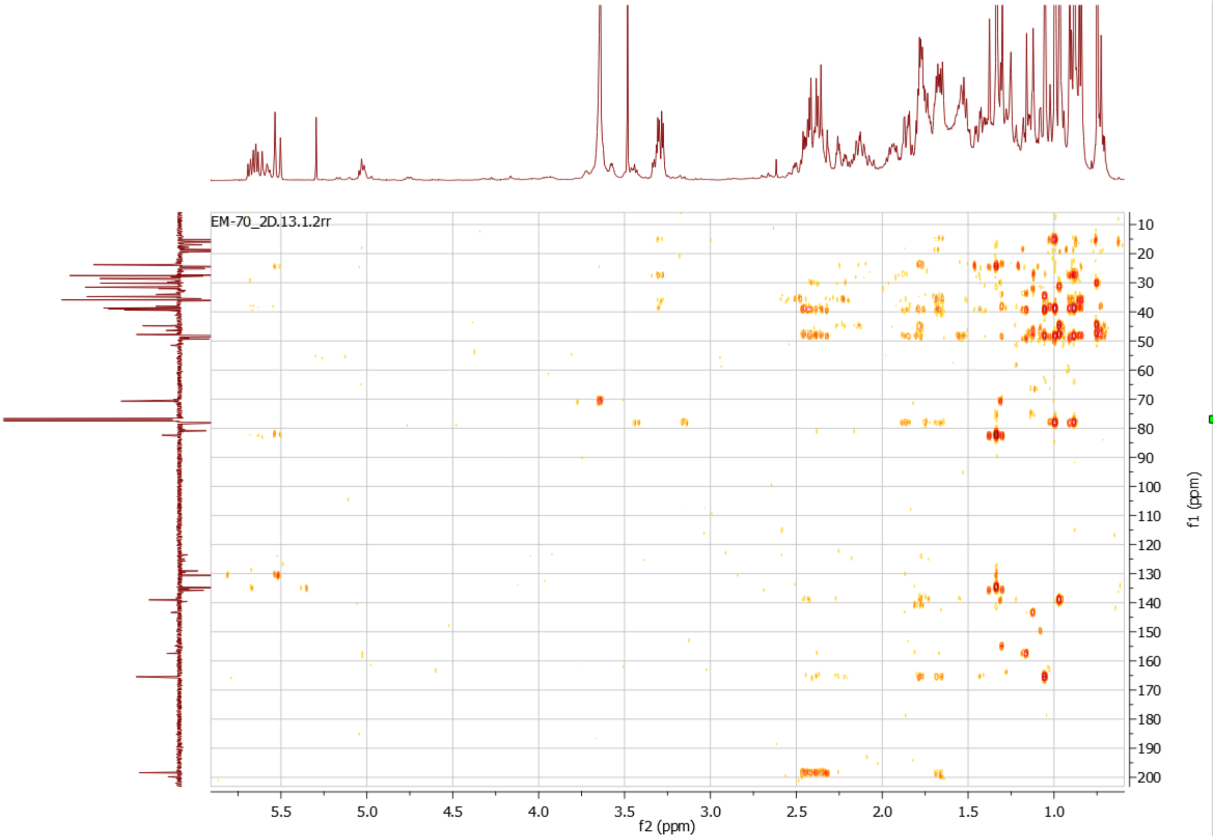

Figure S6. HMBC spectrum compound **1**

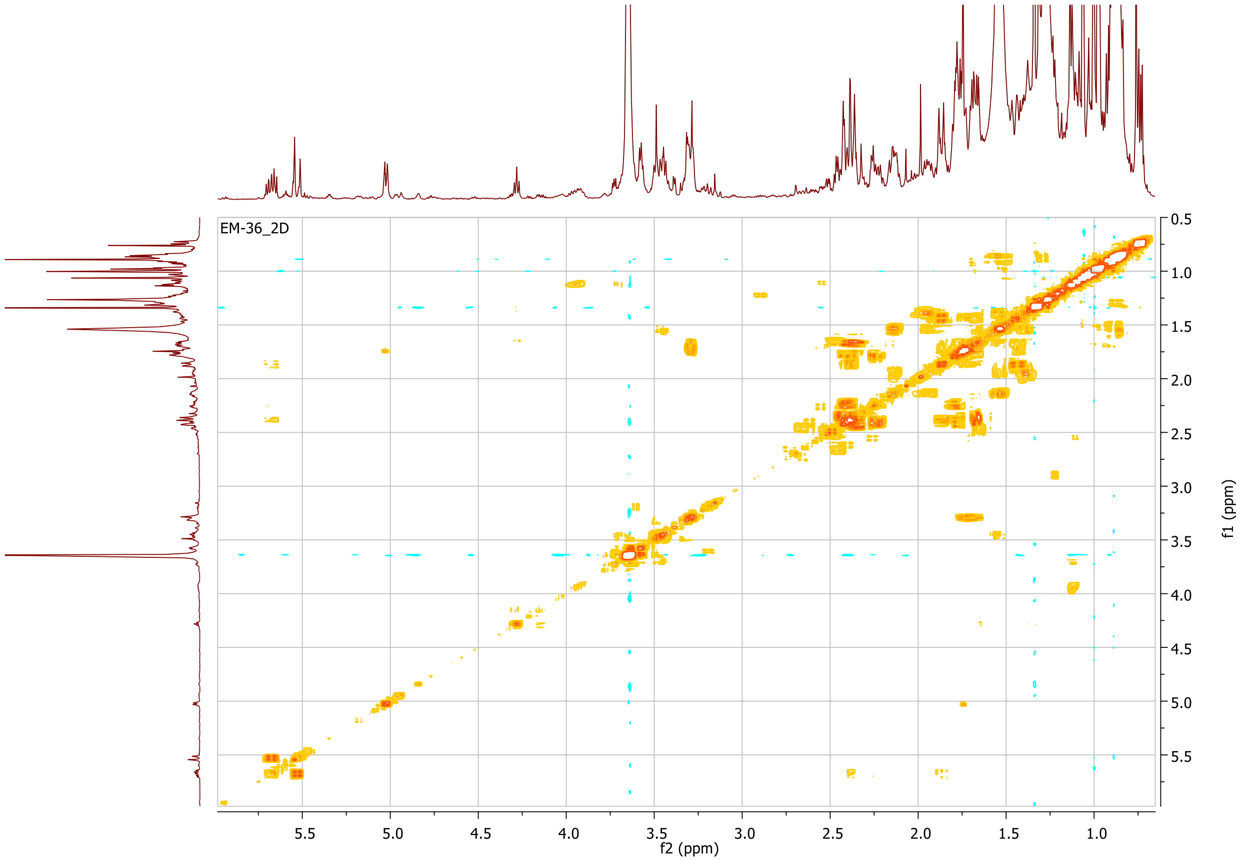

Figure S7. ^{1}H-^{1}H COSY spectrum compound **1**

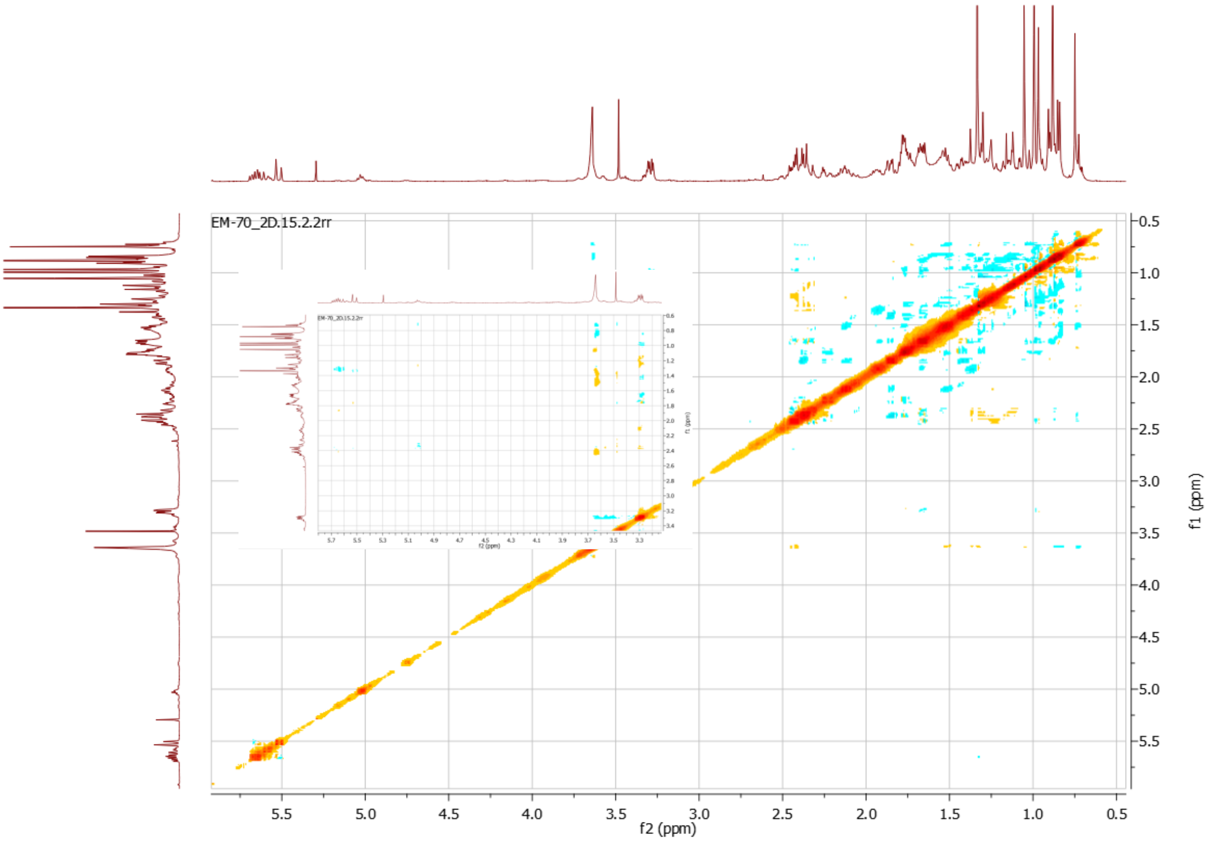

Figure S8. NOESY spectrum of compound **1**

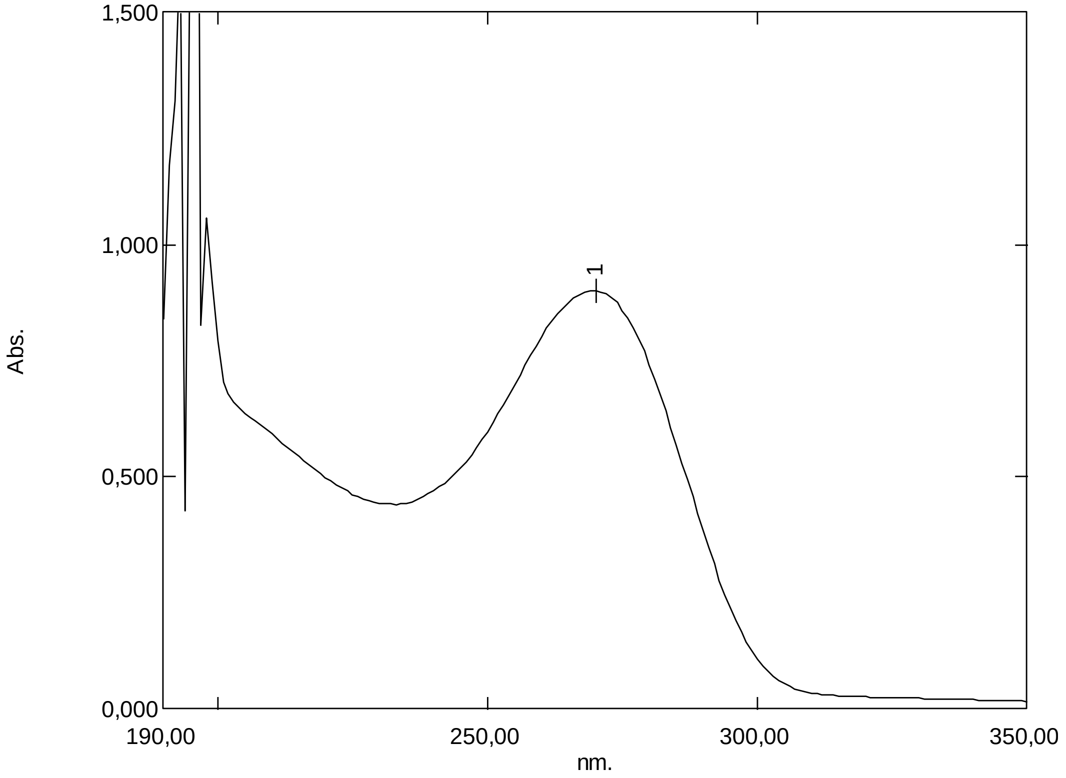

Figure S9. UV spectrum of compound **2**

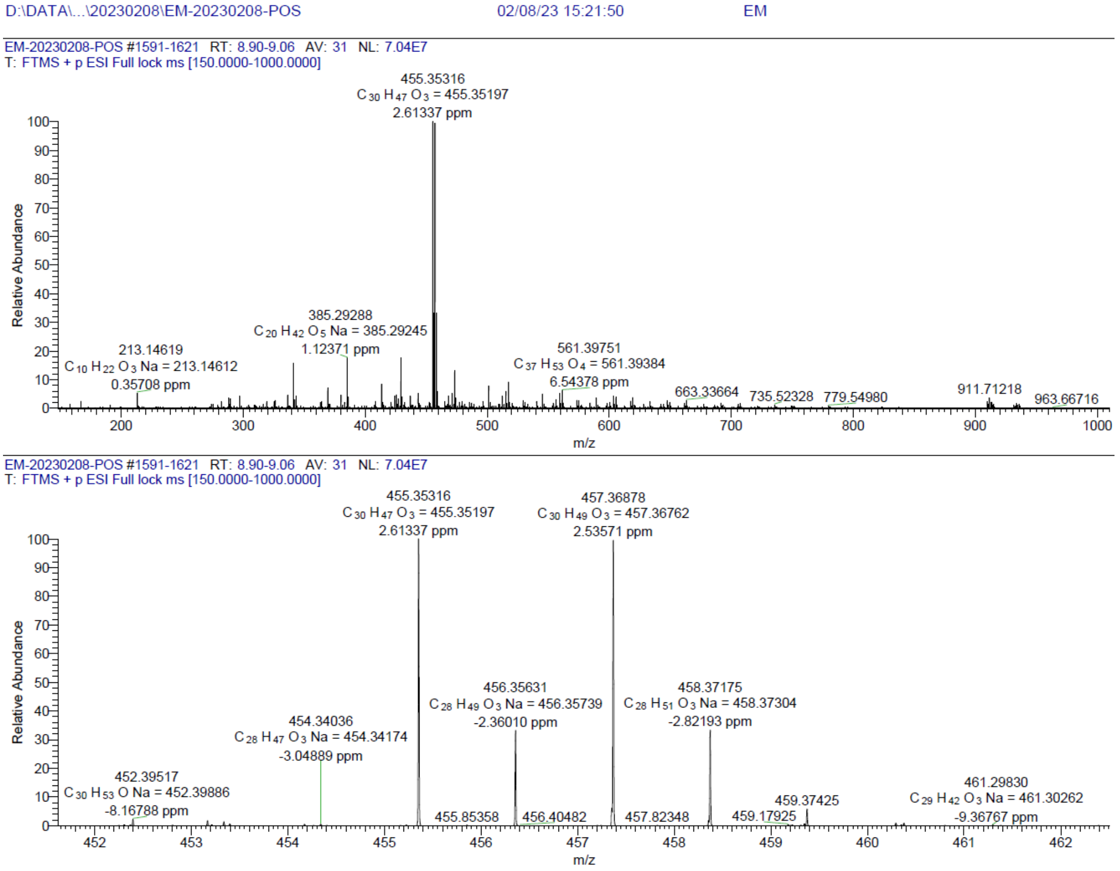

Figure S10. HRESIMS spectrum of compound **2**

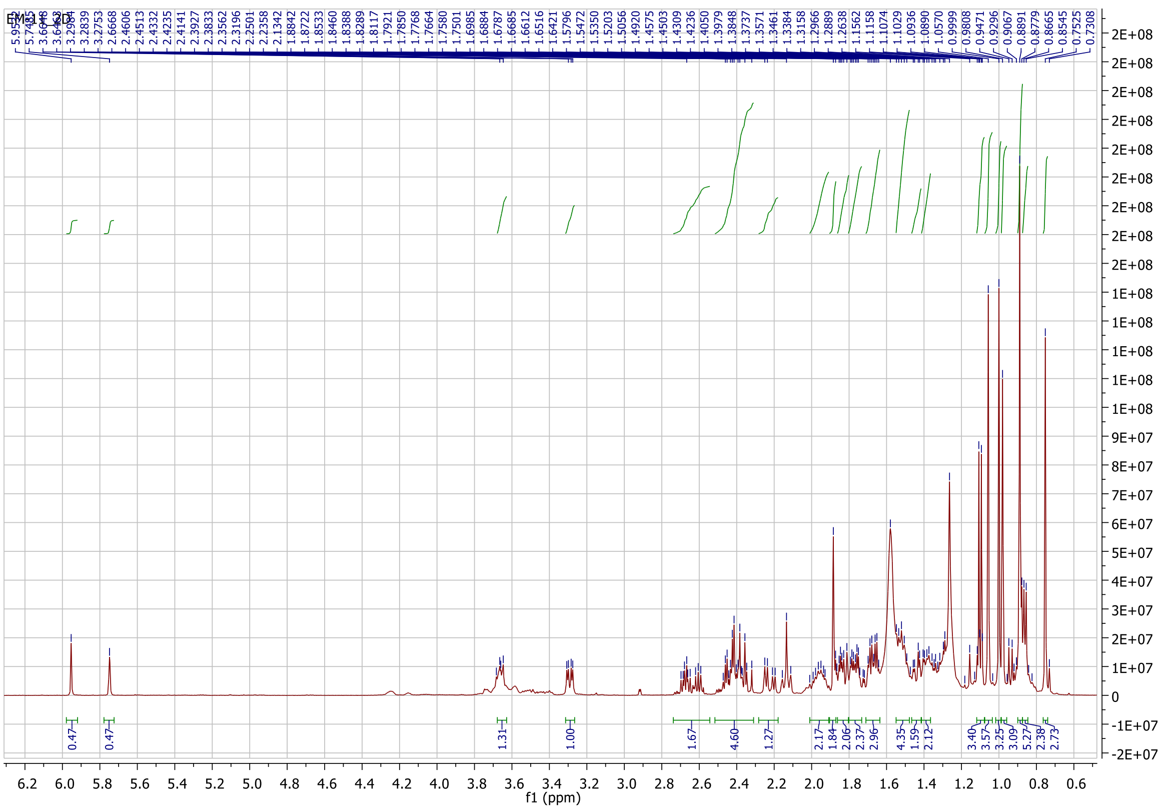

Figure S11. ^{1}H NMR spectrum compound **2** (500 MHz, $CDCl_3$)

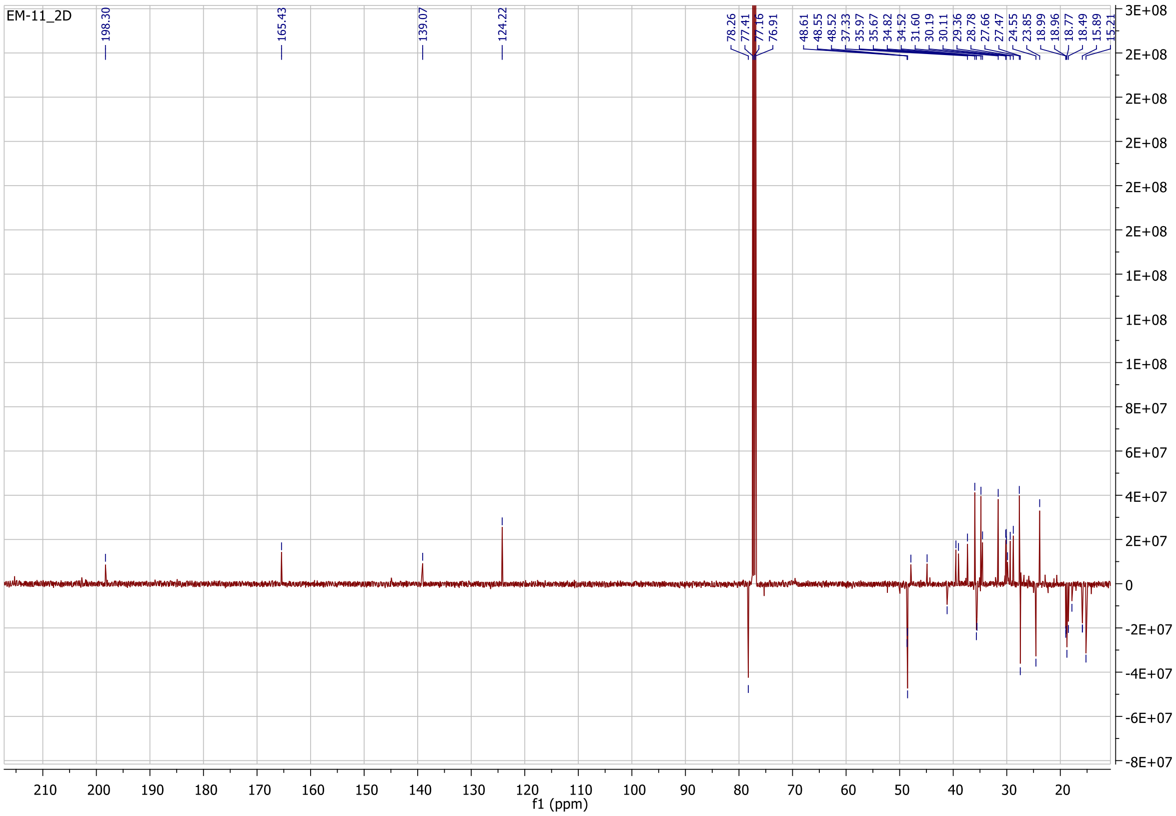

Figure S12. ^{13}C NMR JMOD spectrum compound **2** (125 MHz, $CDCl_3$)

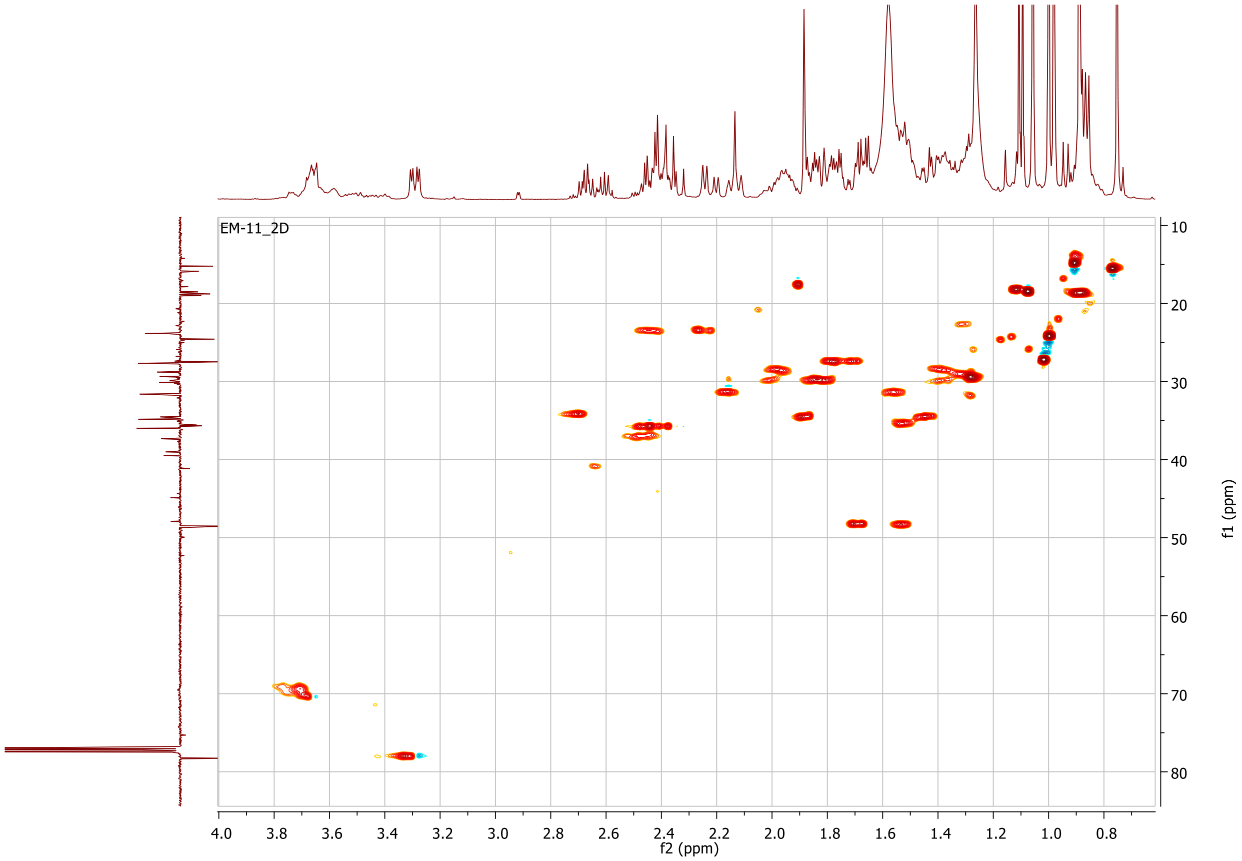

Figure S13. HSQC spectrum compound **2**

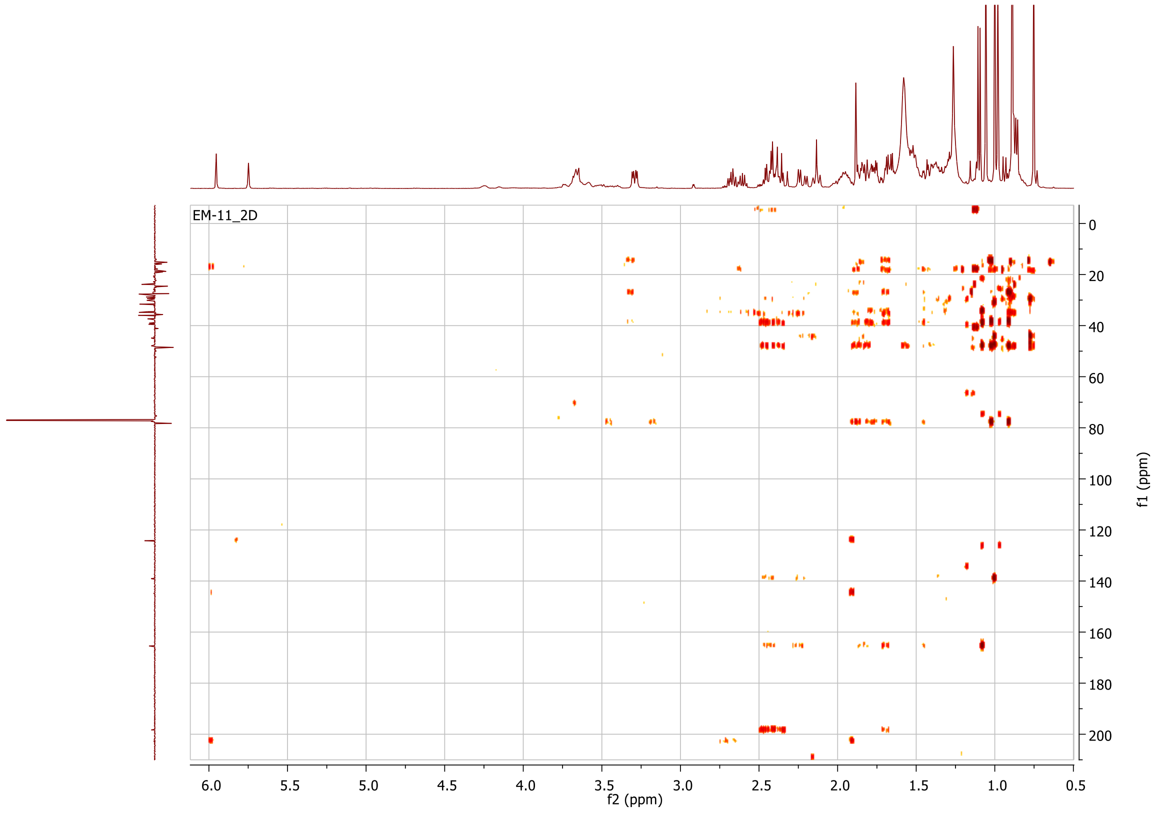

Figure S14. HMBC spectrum compound **2**

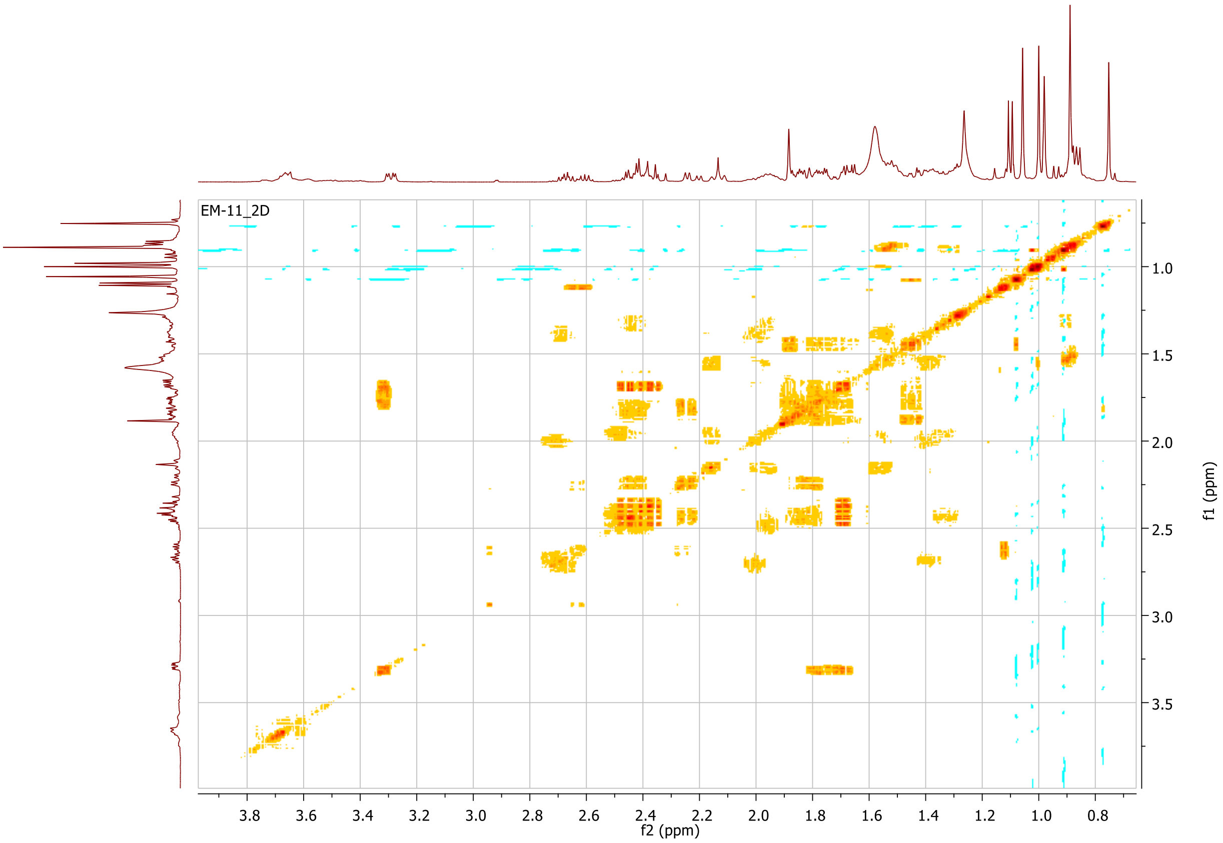

Figure S15. ^{1}H-^{1}H COSY spectrum compound **2**

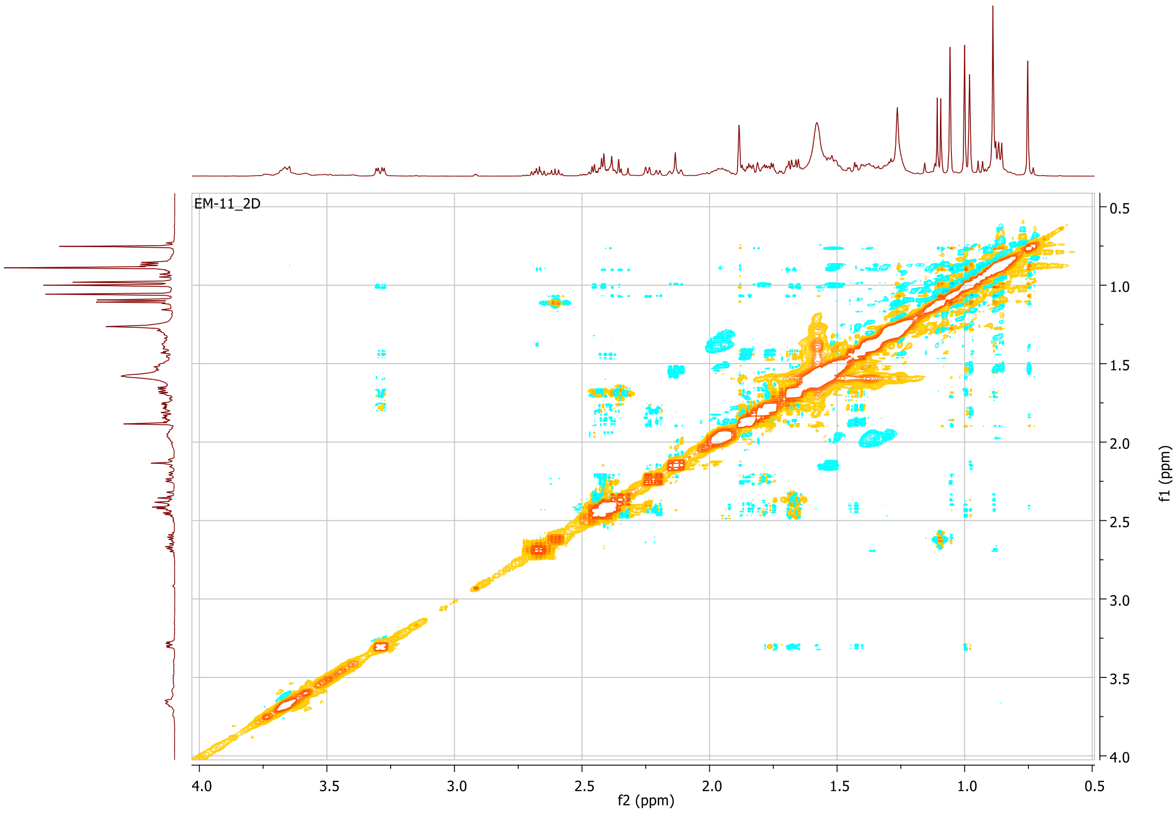

Figure S16. NOESY spectrum of compound **2**

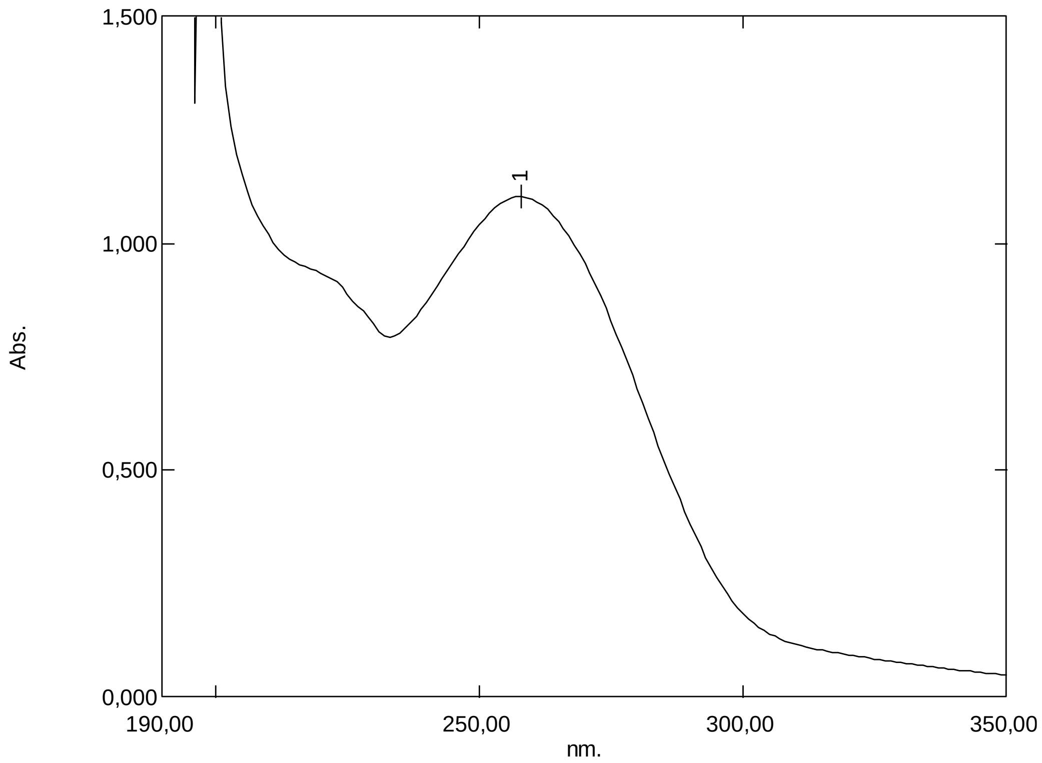

Figure S17. UV spectrum of compound **3**

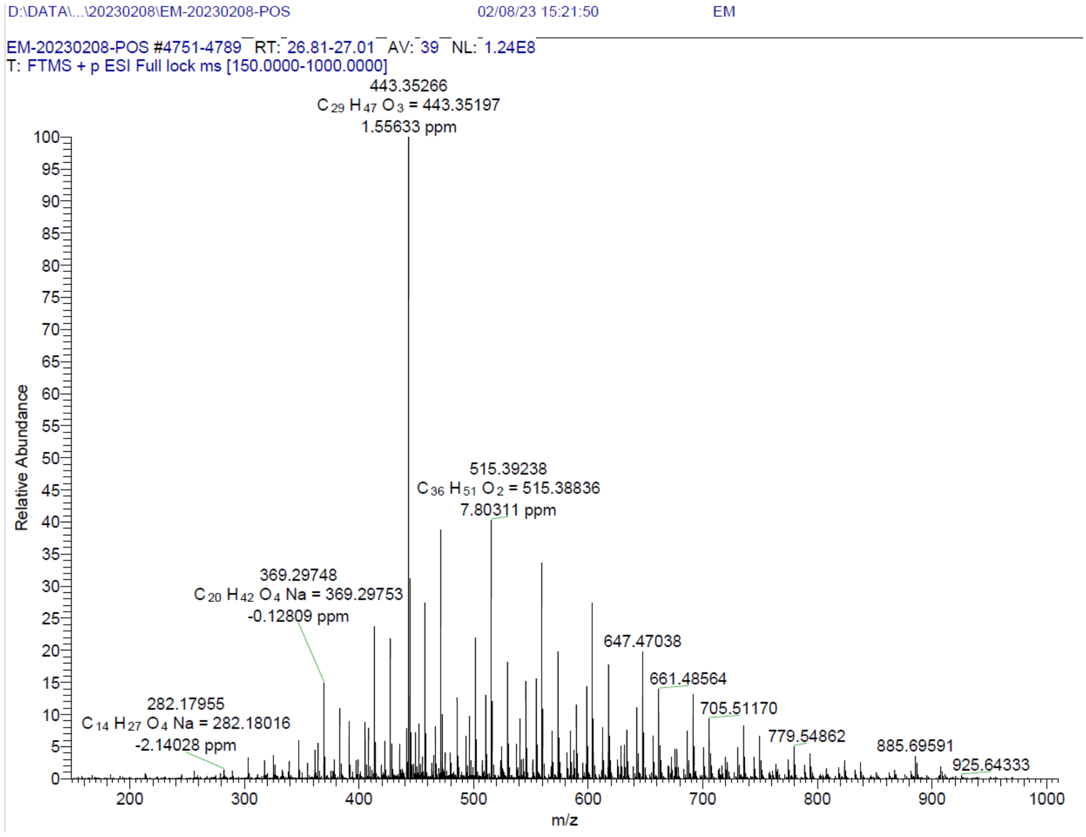

Figure S18. ESIMS spectrum of compound **3**

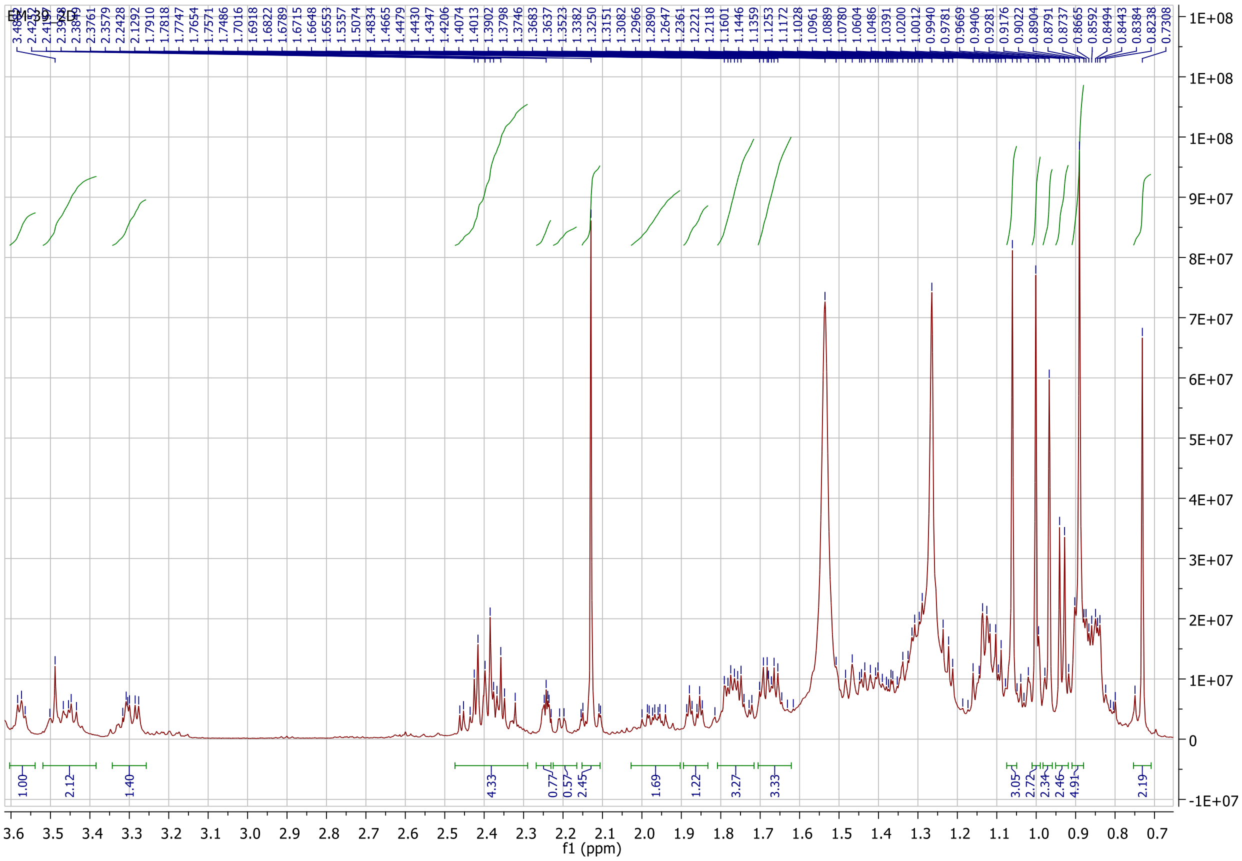

Figure S19. ^{1}H NMR spectrum compound **3** (500 MHz, $CDCl_3$)

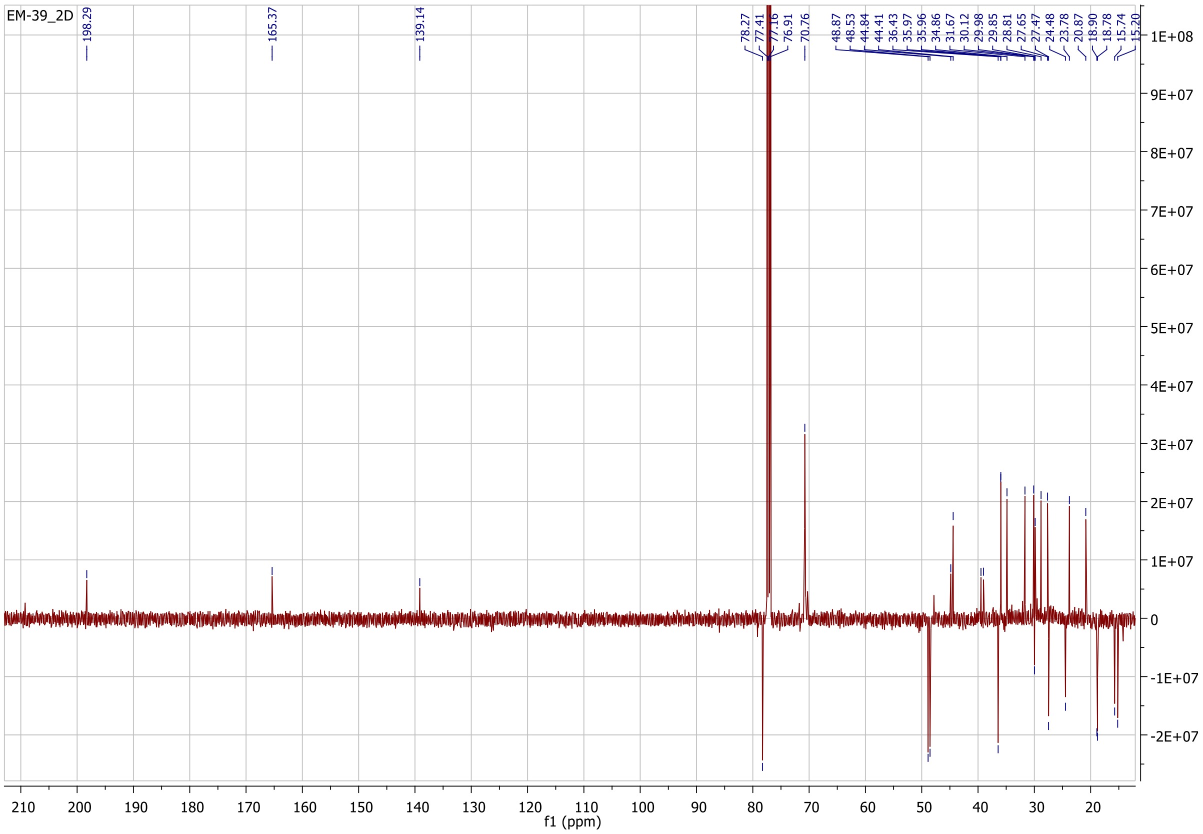

Figure S20. ^{13}C NMR JMOD spectrum compound **3** (125 MHz, $CDCl_3$)

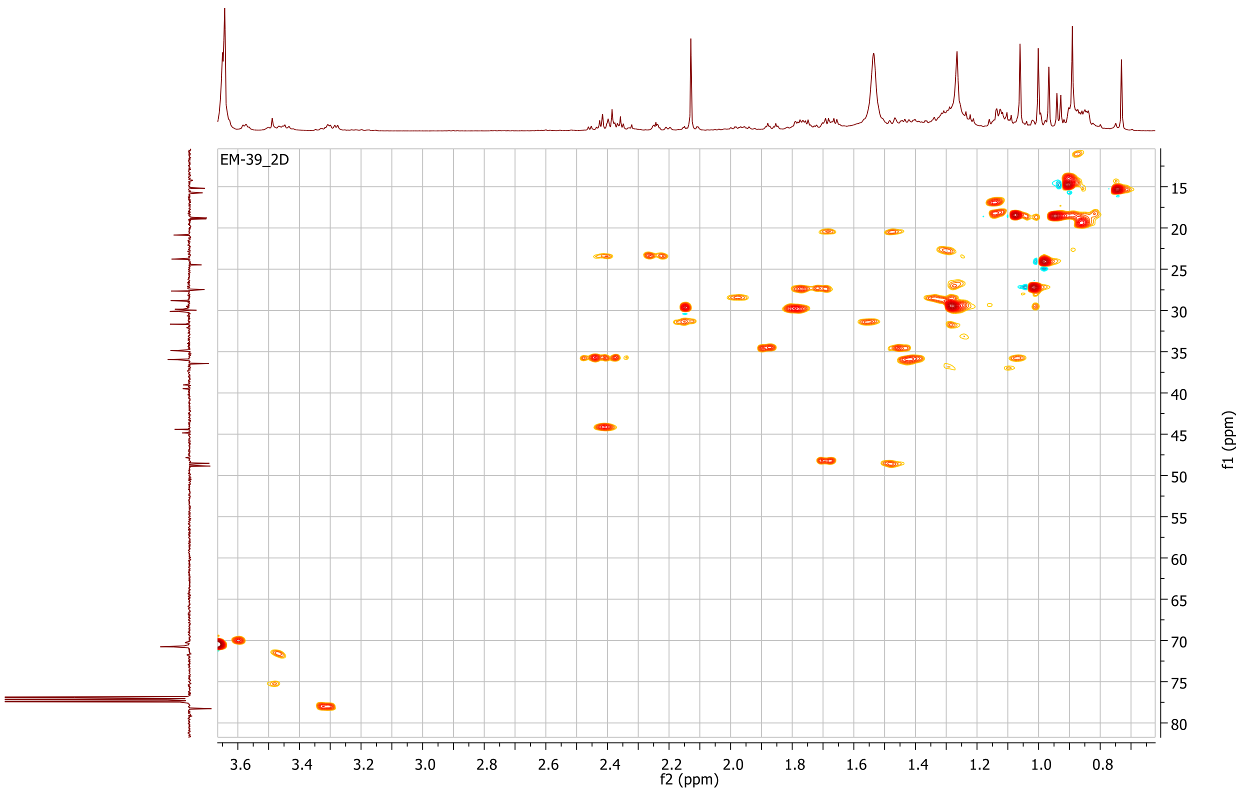

Figure S21. HSQC spectrum compound **3**

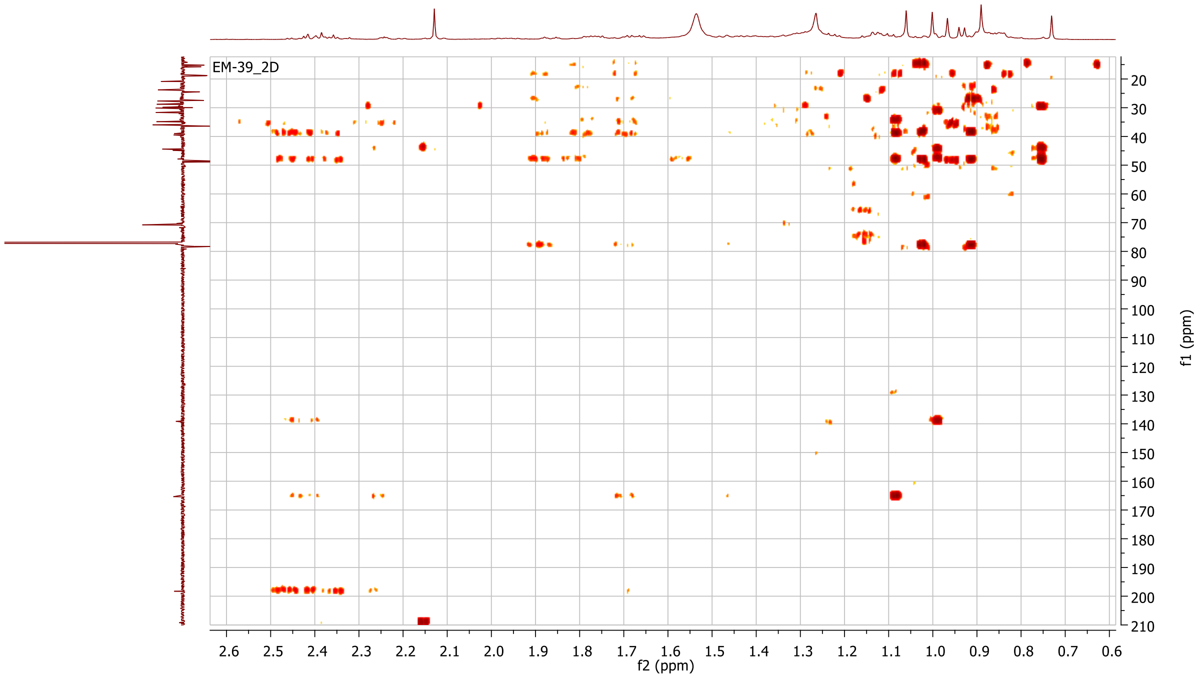

Figure S22. HMBC spectrum compound **3**

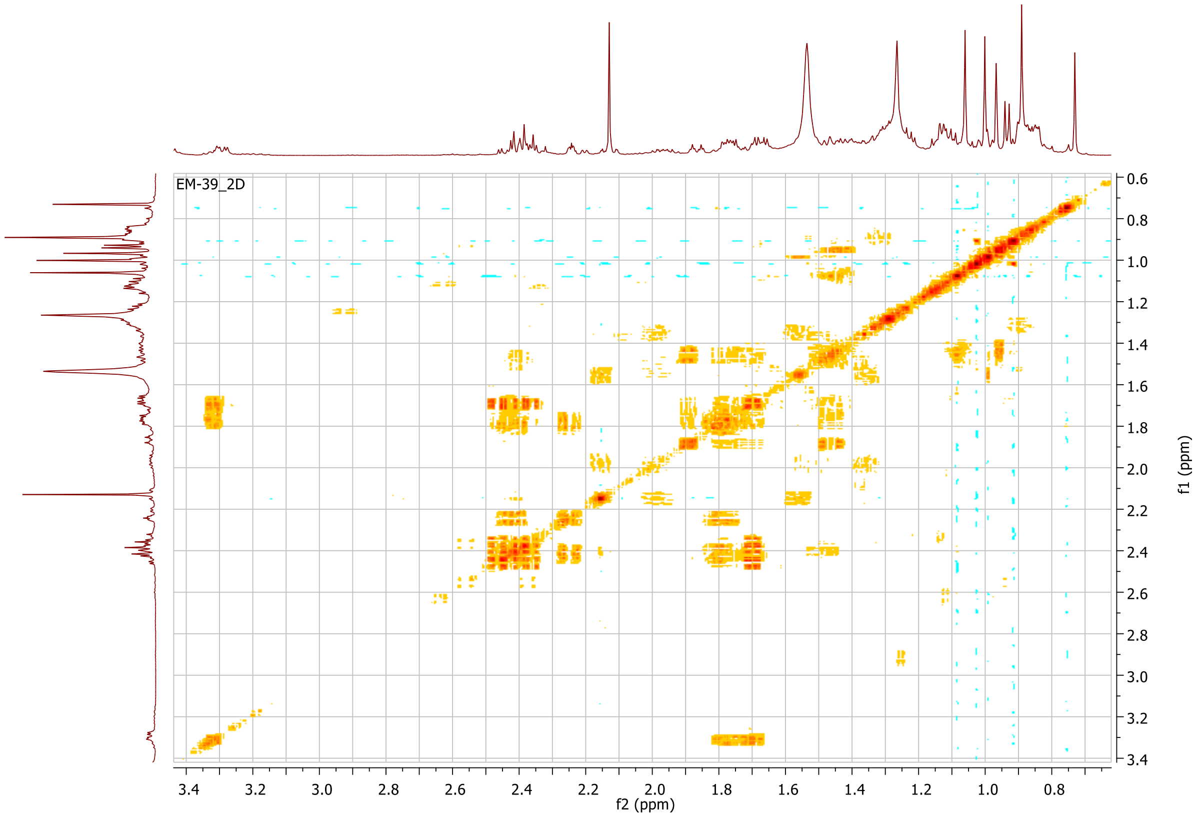

Figure S23. ^{1}H-^{1}H COSY spectrum compound **3**

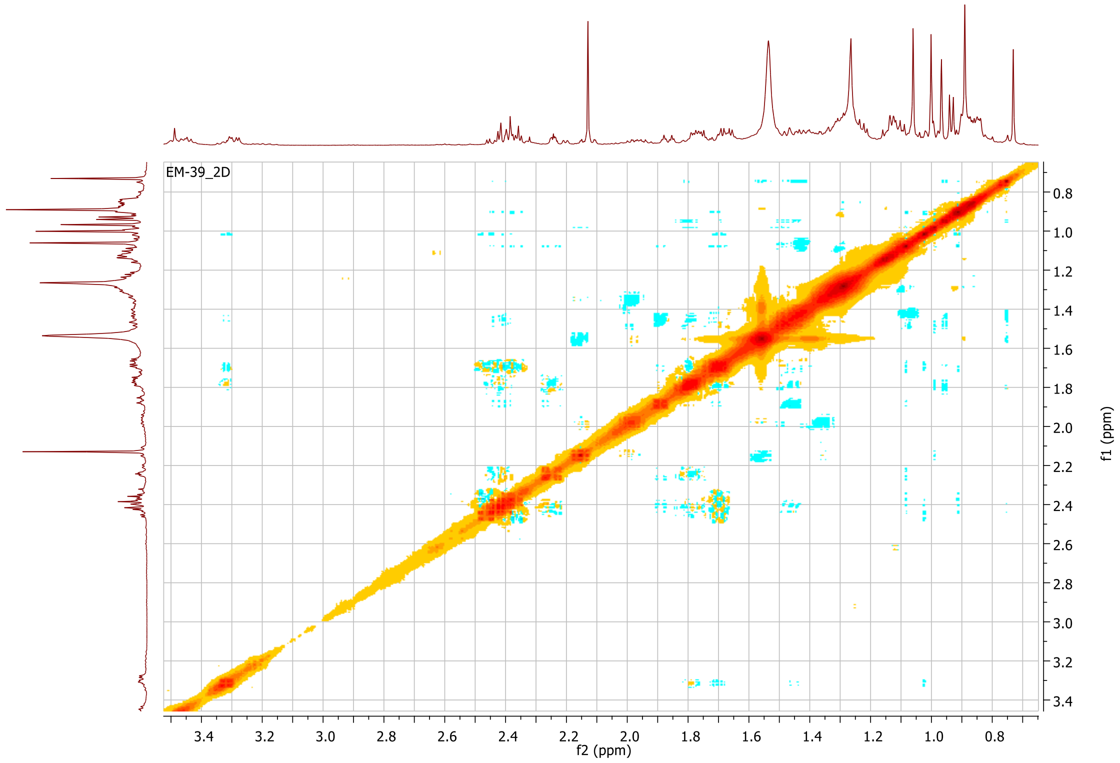

Figure S24. NOESY spectrum of compound **3**

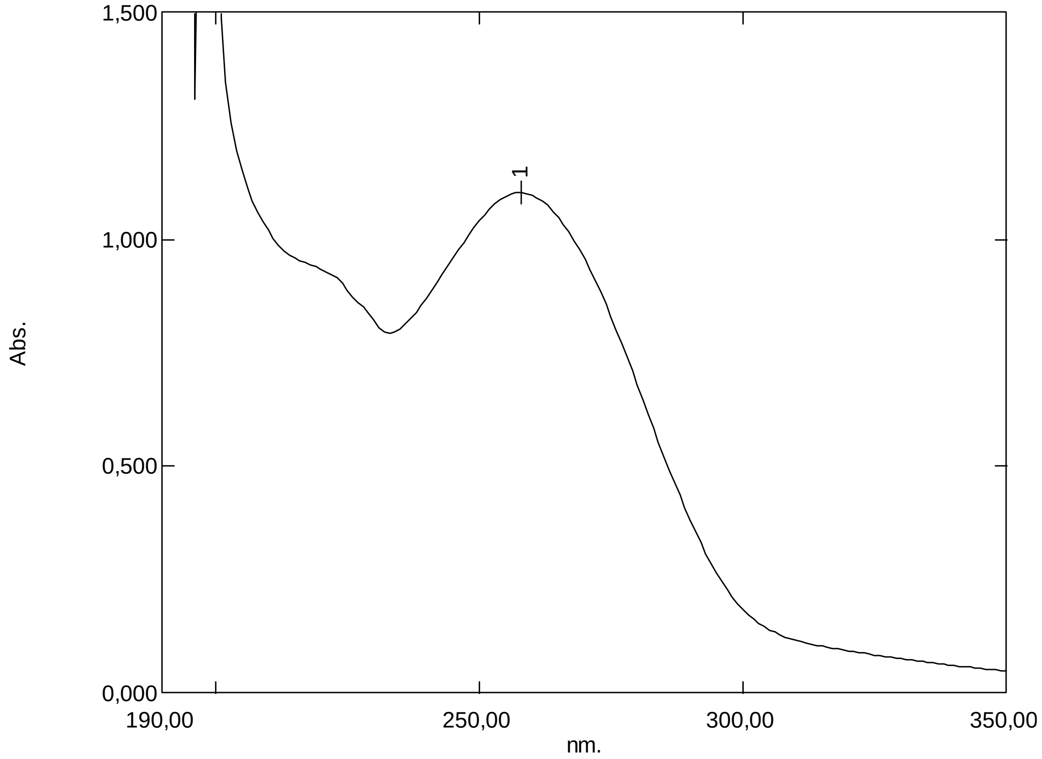

Figure S25. UV spectrum of compound **4**

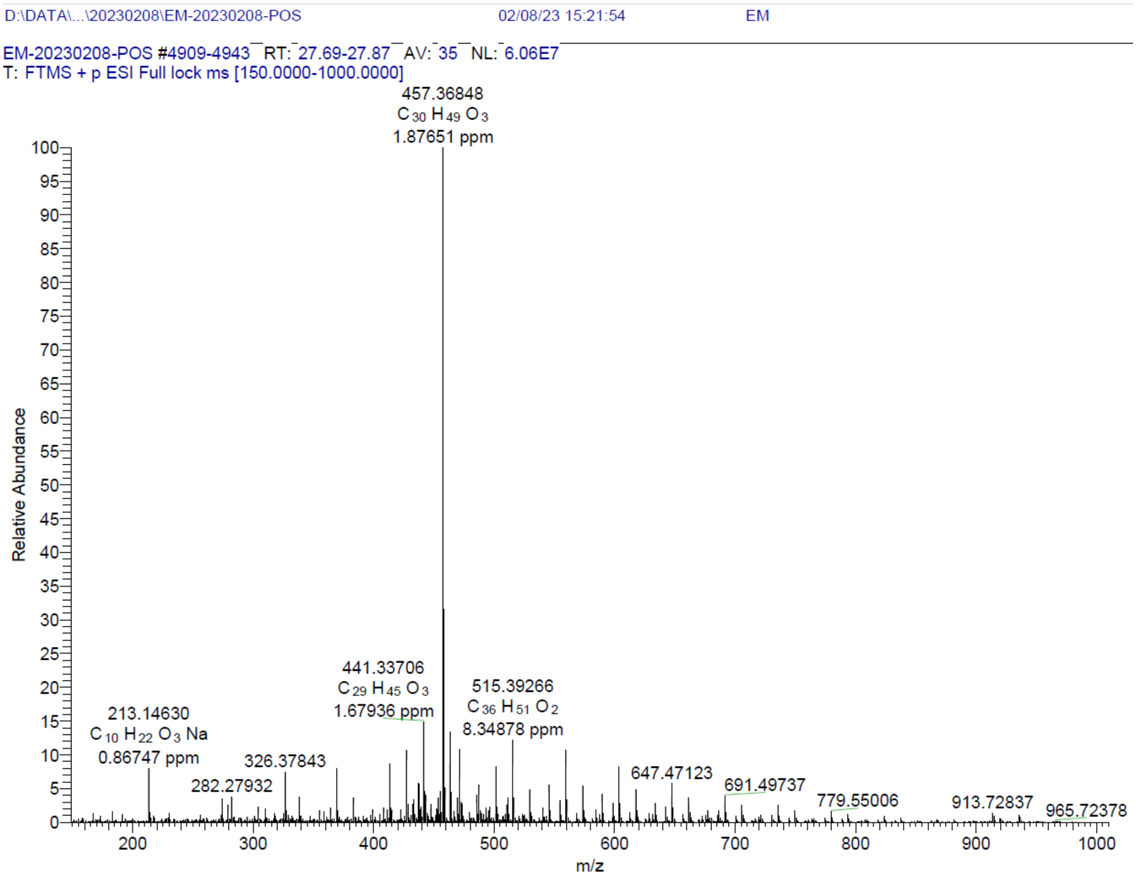

Figure S26. HRESIMS spectrum of **4**

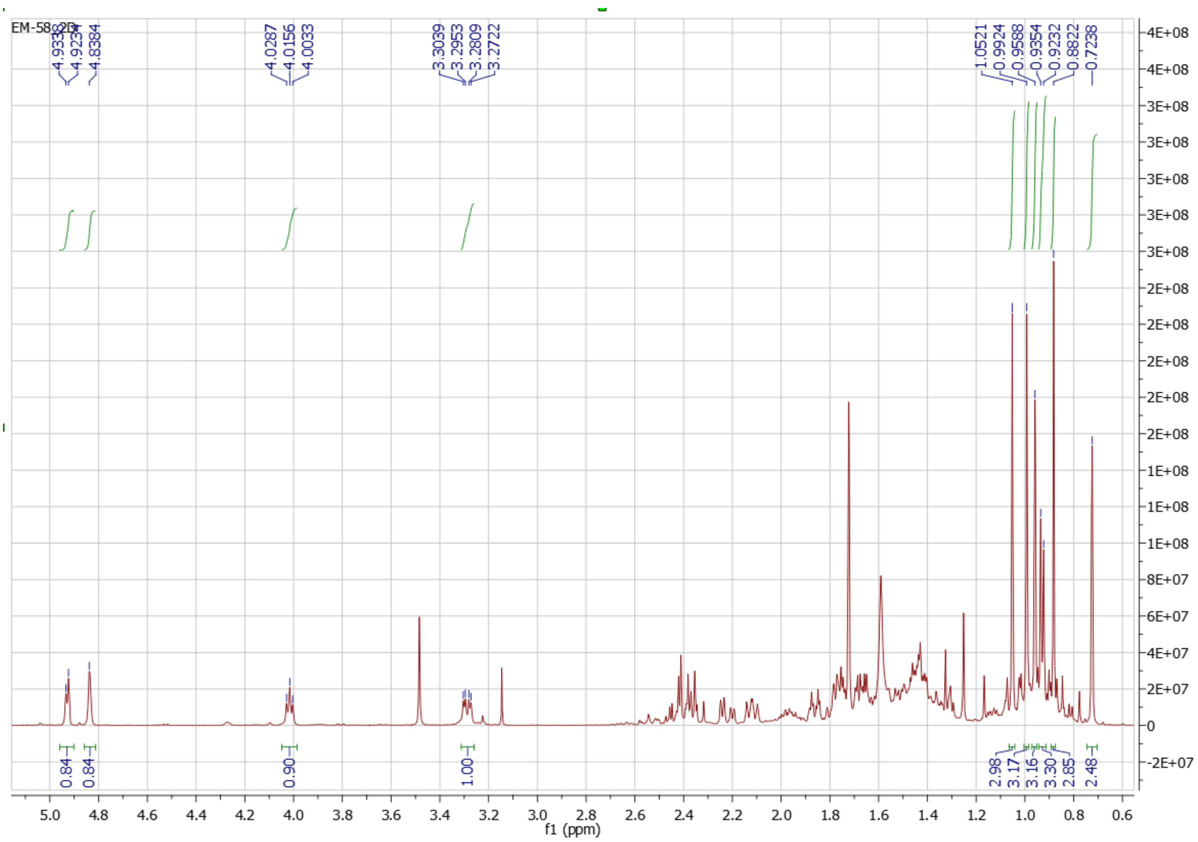

Figure S27. ^{1}H NMR spectrum of compound **4** (500 MHz, $CDCl_3$)

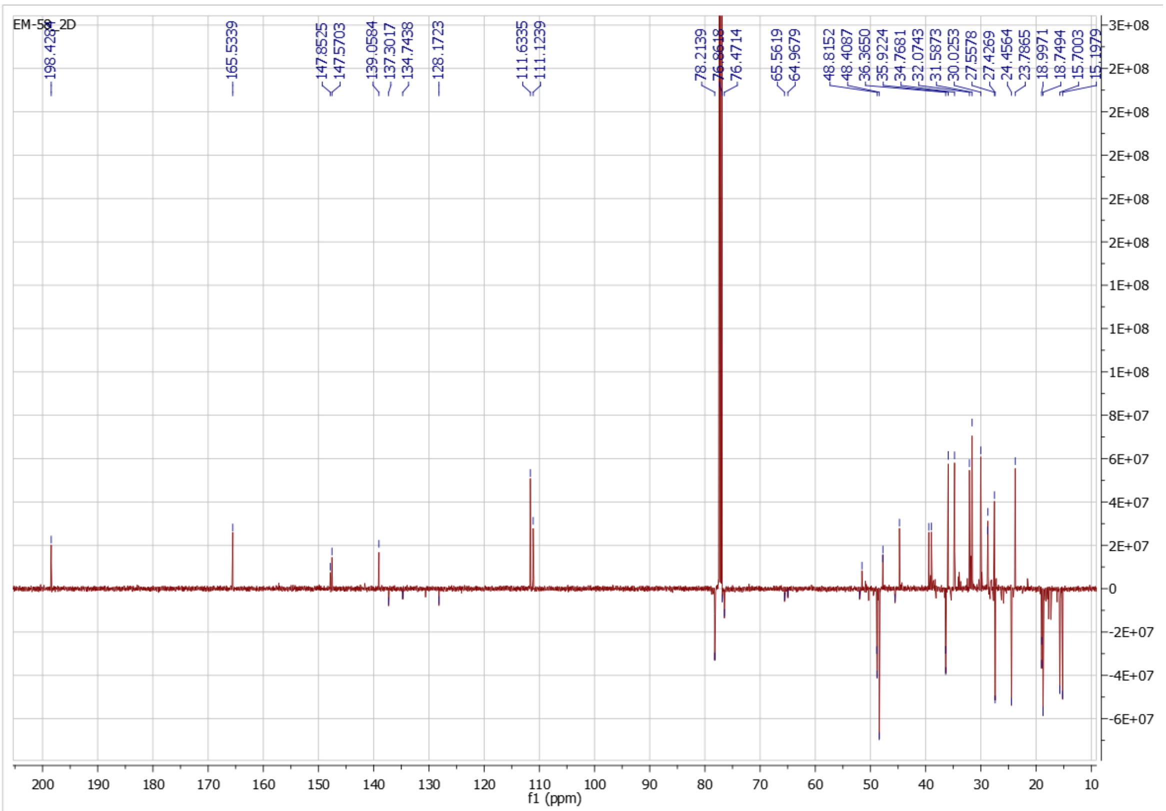

Figure S28. ^{13}C NMR JMOD spectrum of compound **4** (125 MHz, $CDCl_3$)

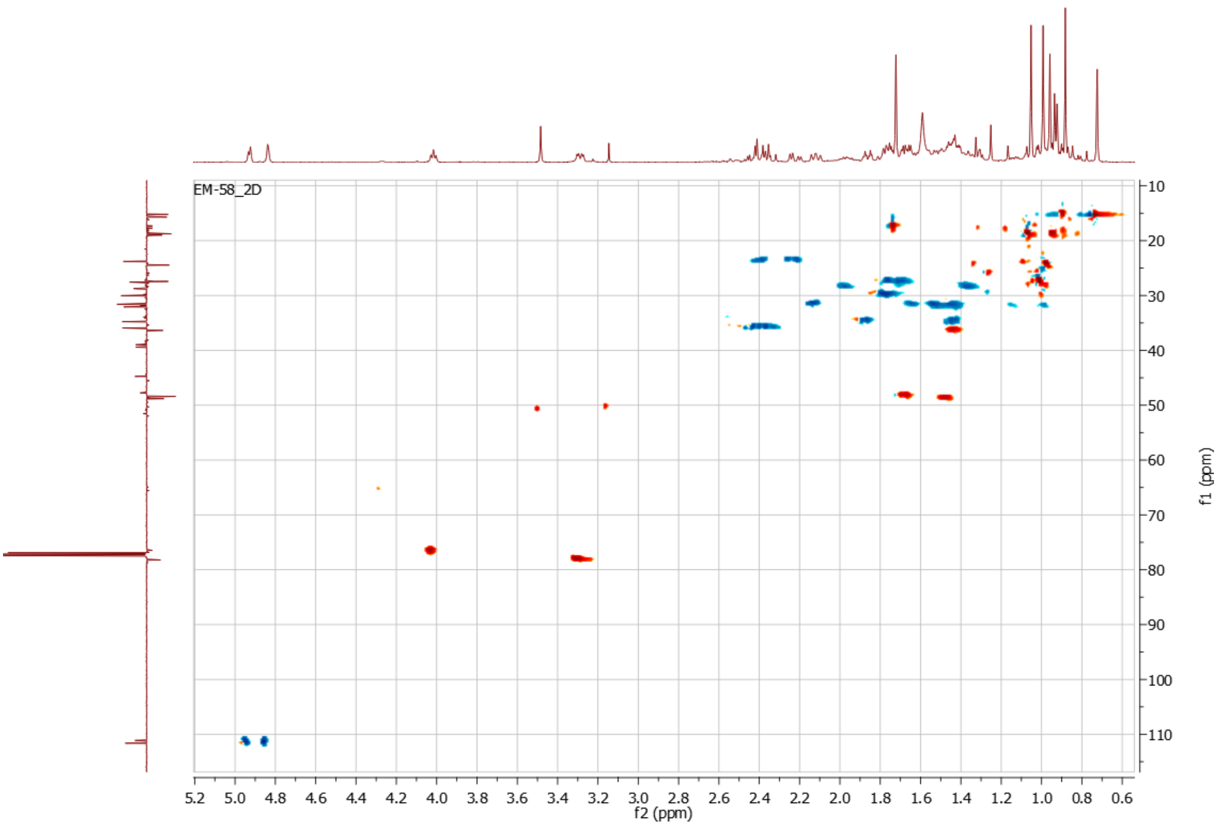

Figure S29. HSQC spectrum of compound **4**

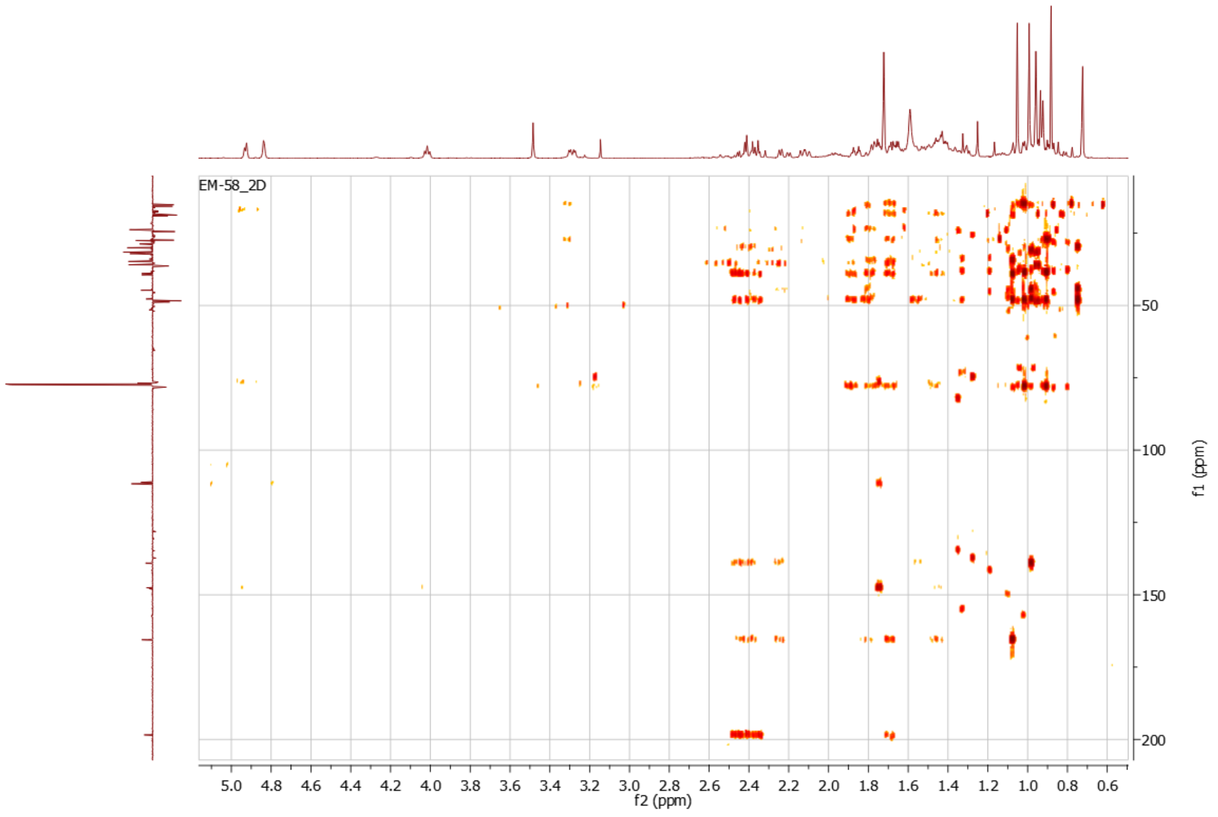

Figure S30. HMBC spectrum of compound **4**

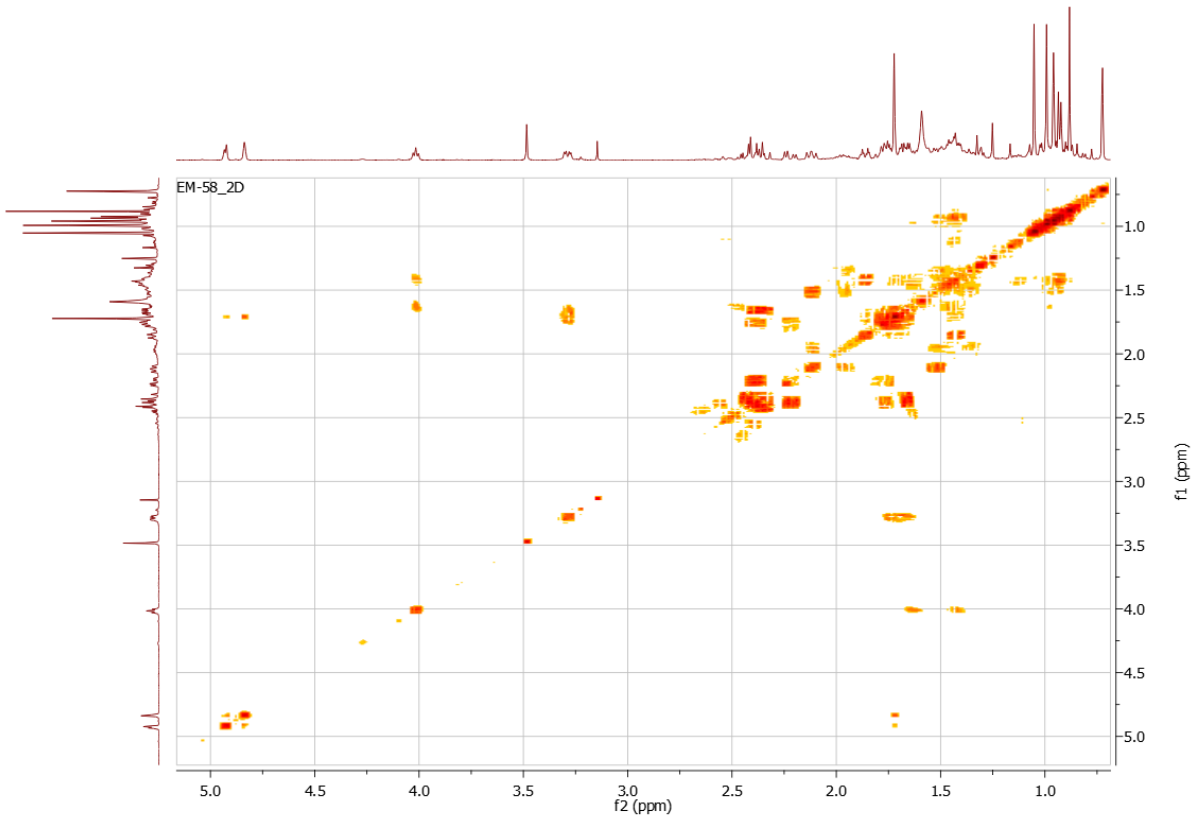

Figure S31. ^{1}H-^{1}H COSY spectrum of compound **4**

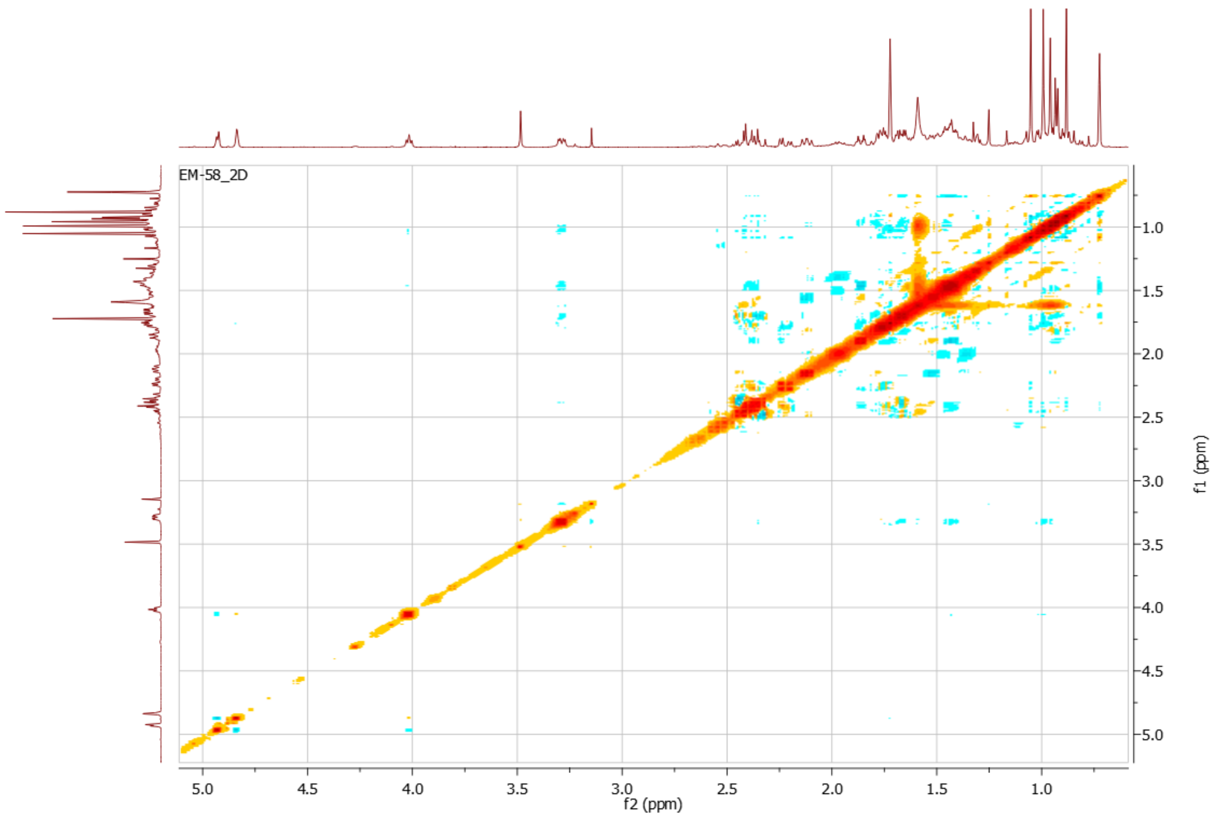

Figure S32. NOESY spectrum of compound **4**

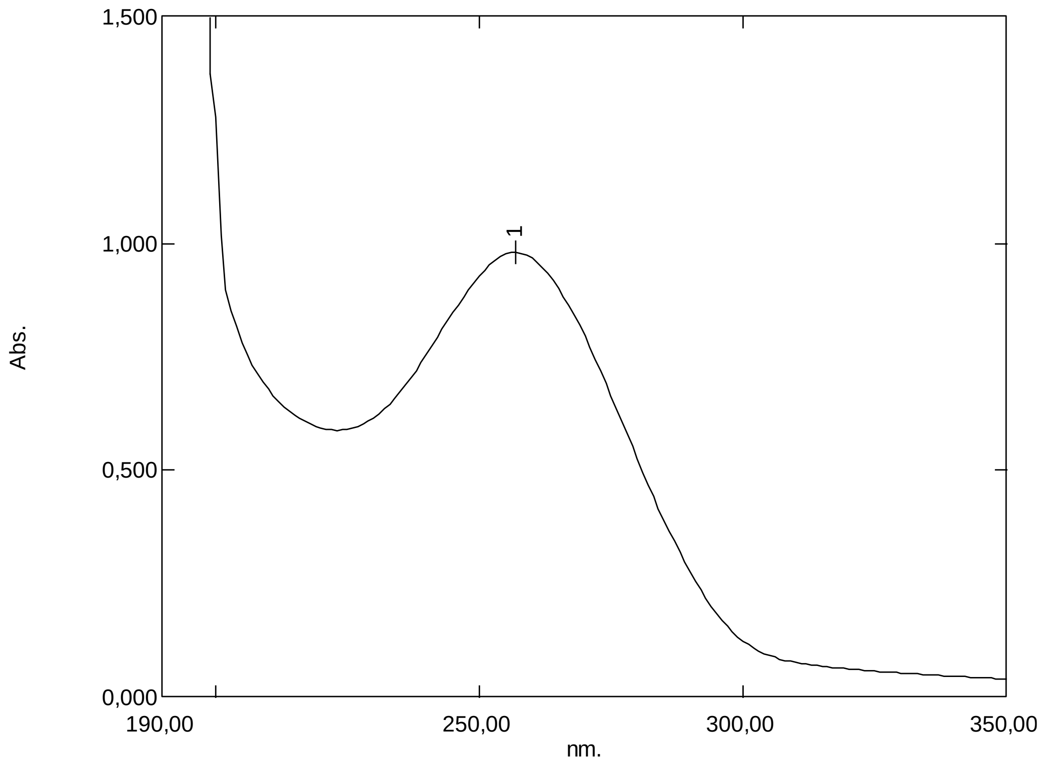

Figure S33. UV spectrum of compound **5**

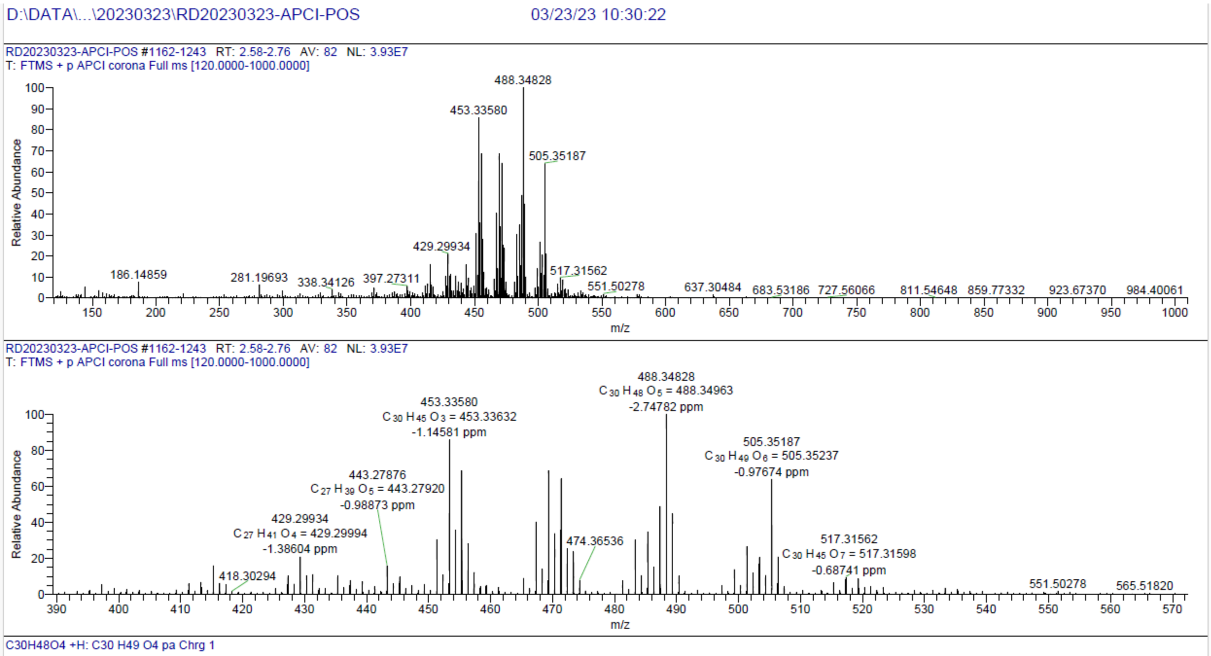

Figure S34. HR-APCI-MS spectrum of compound **5**

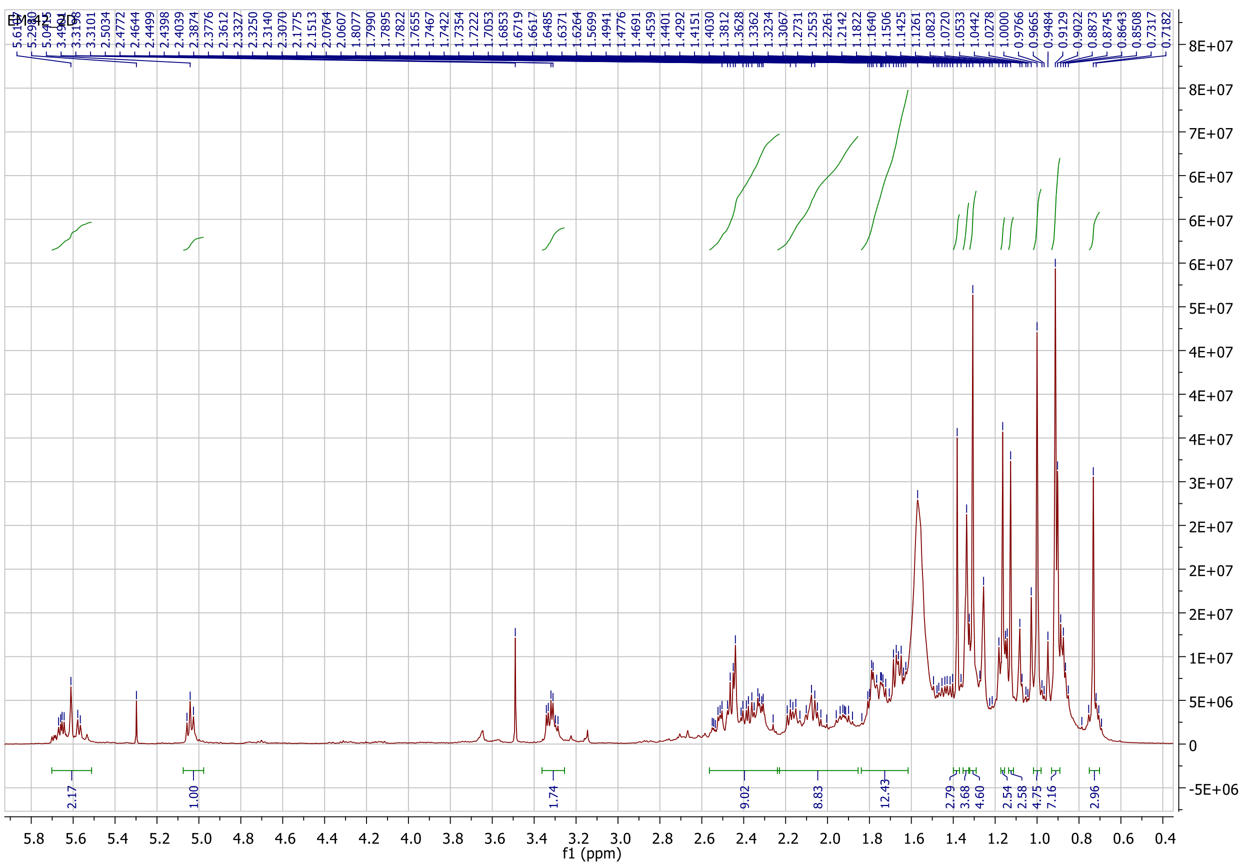

Figure S35. ^{1}H NMR spectrum compound **5** (500 MHz, $CDCl_3$)

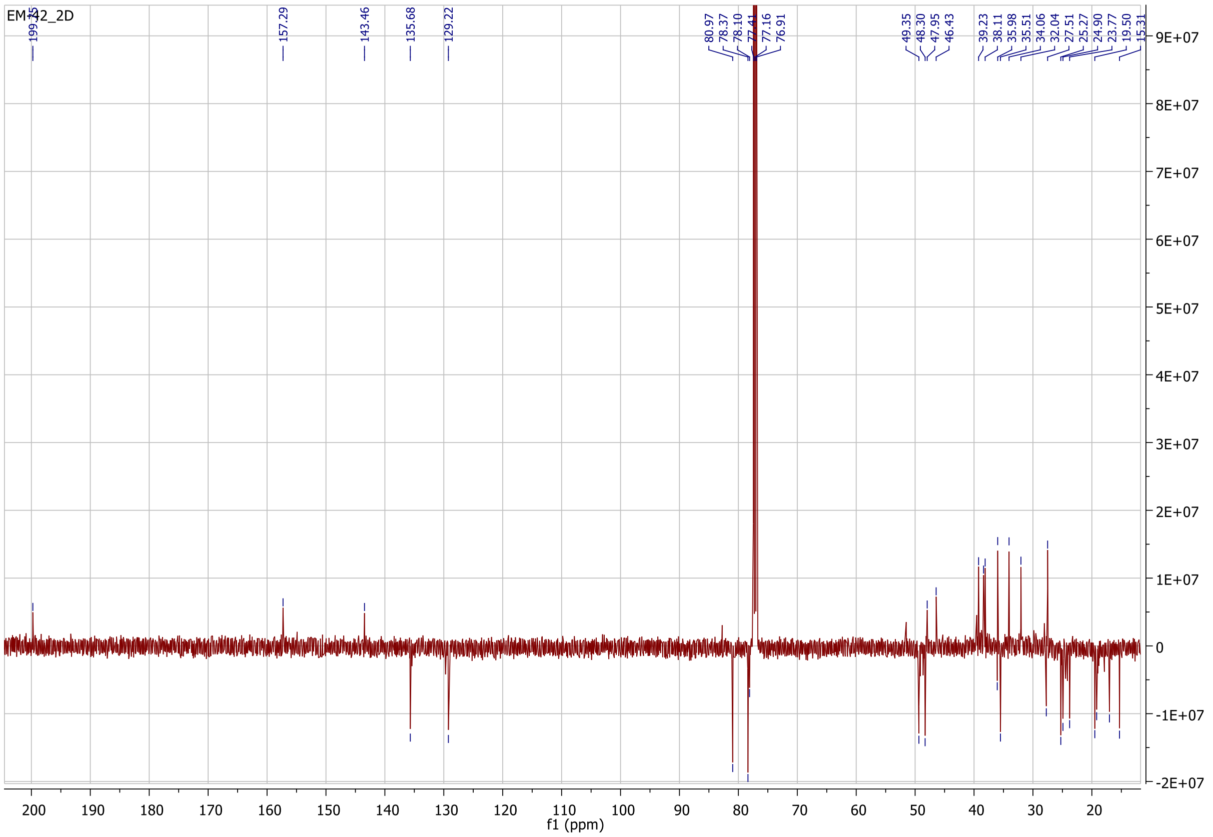

Figure S36. ^{13}C NMR JMOD spectrum compound **5** (125 MHz, $CDCl_3$)

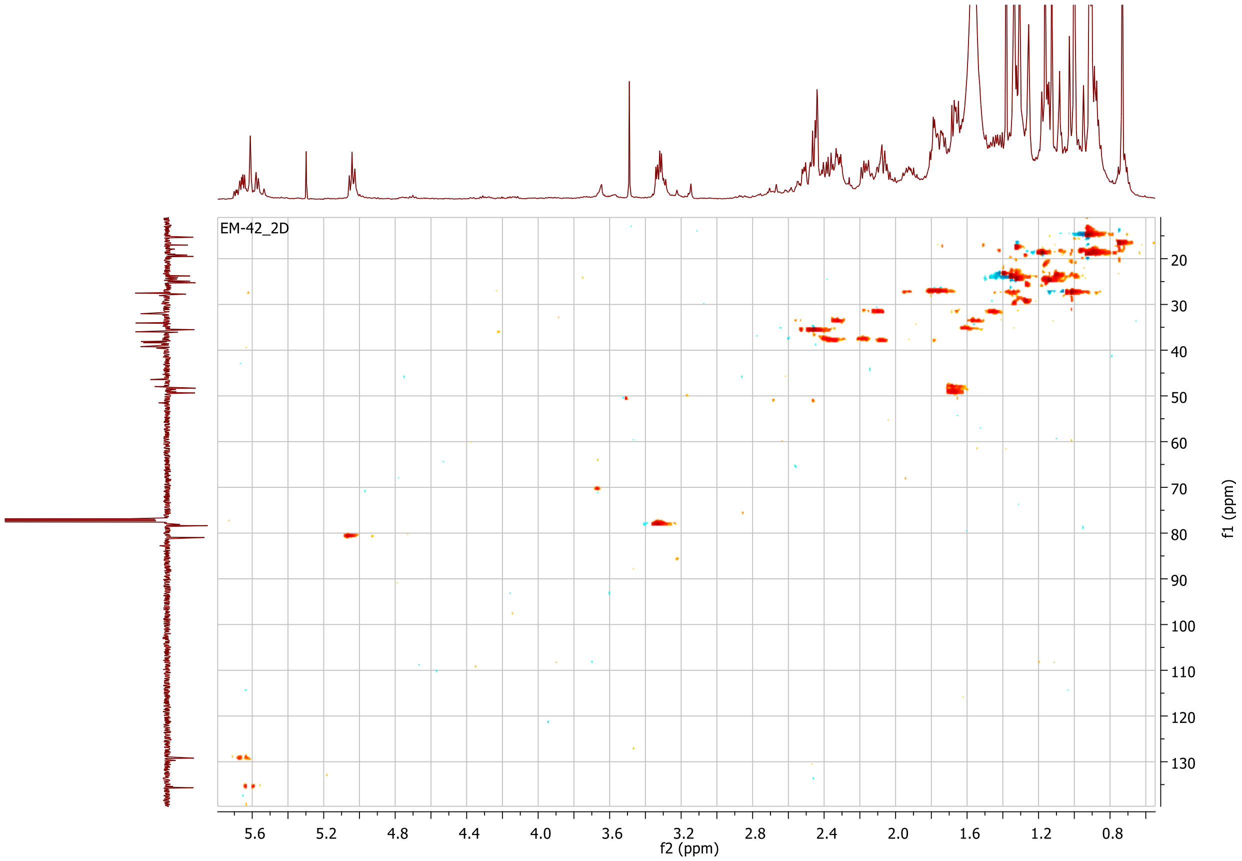

Figure S37. HSQC spectrum compound **5**

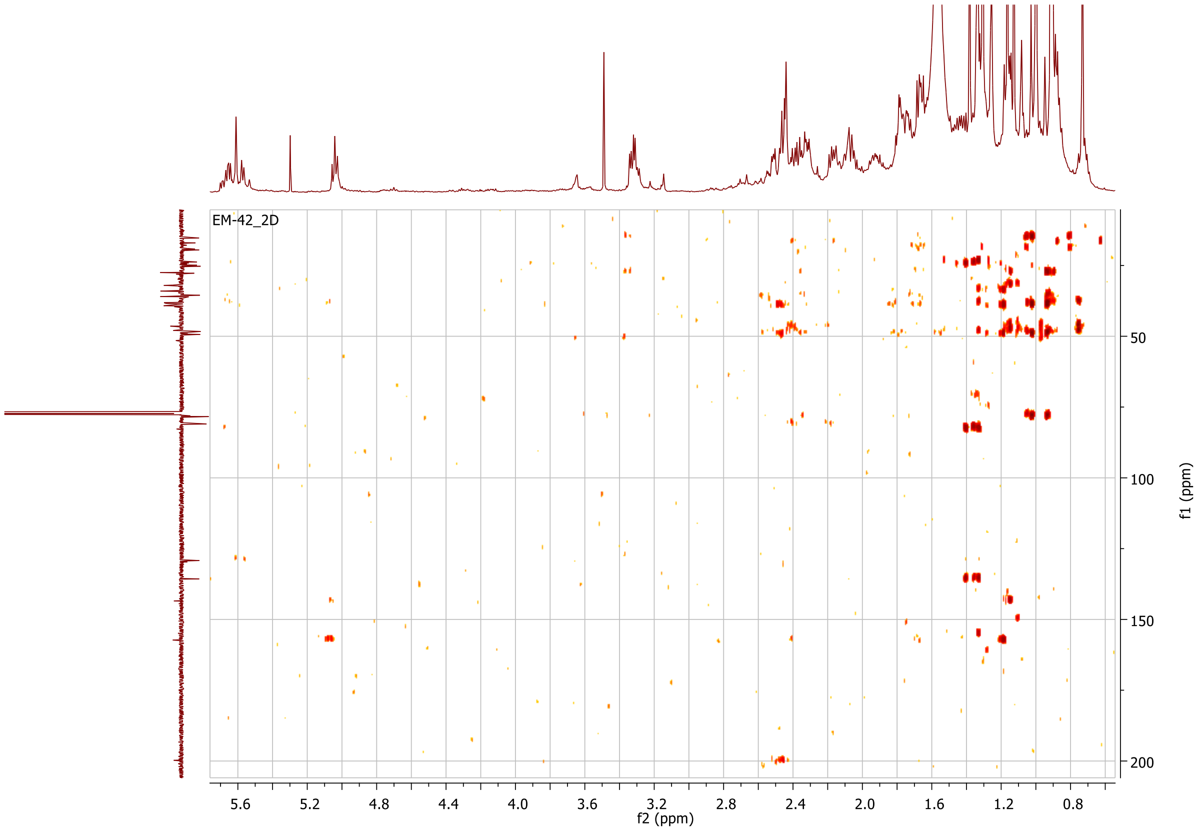

Figure S38. HMBC spectrum compound **5**

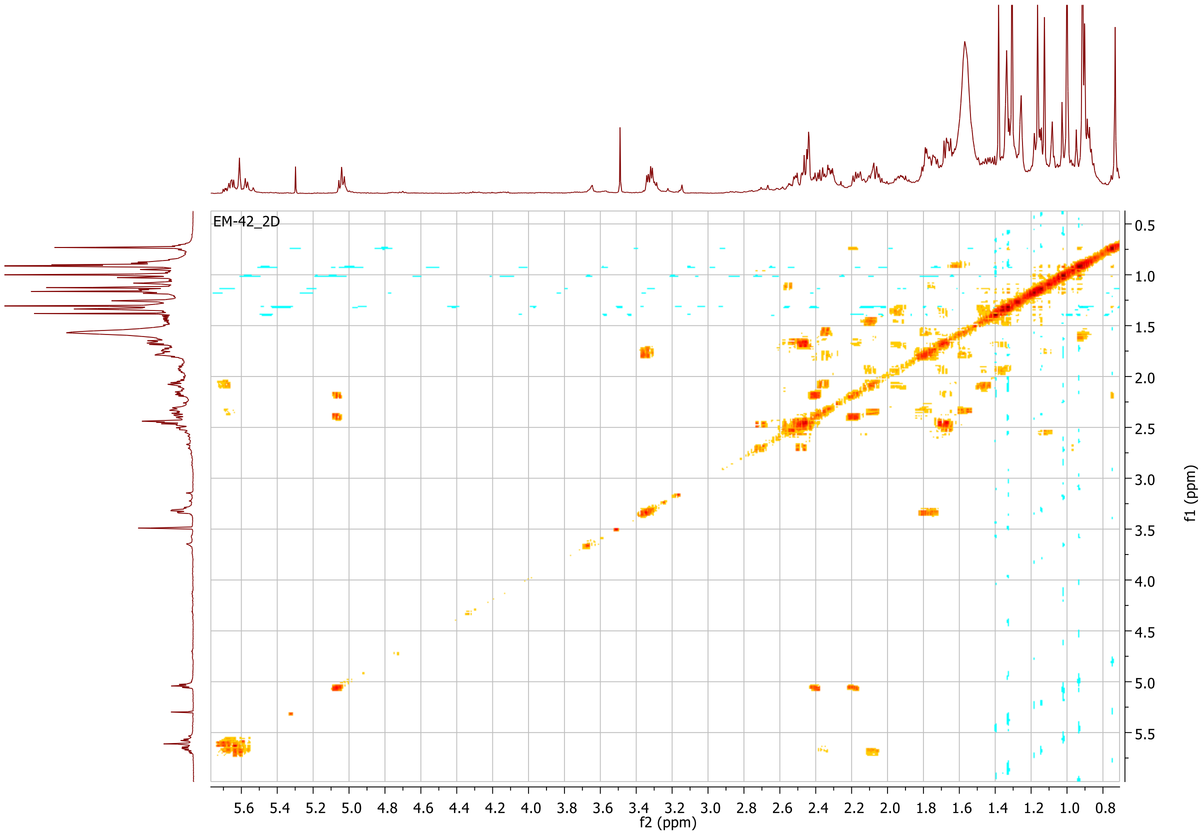

Figure S39. ^{1}H-^{1}H COSY spectrum compound **5**

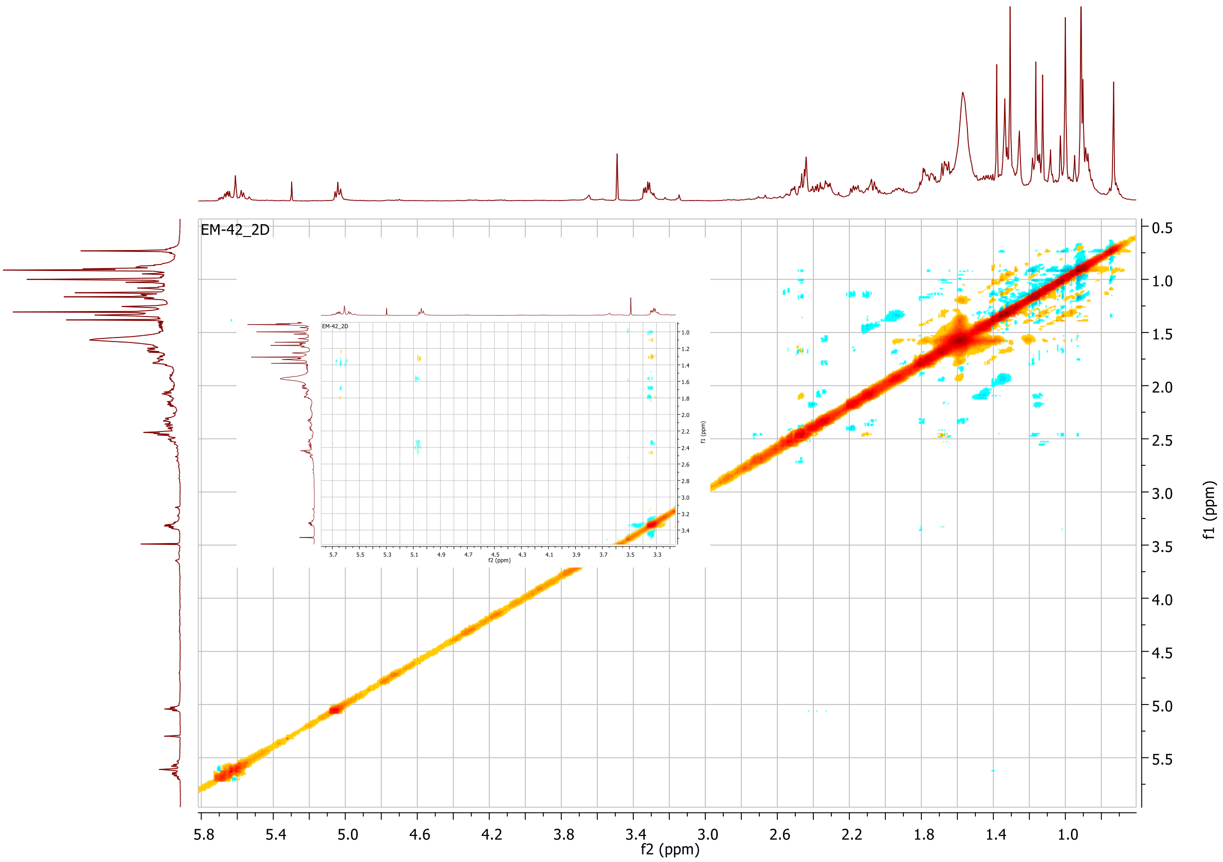

Figure S40. NOESY spectrum of compound **5**

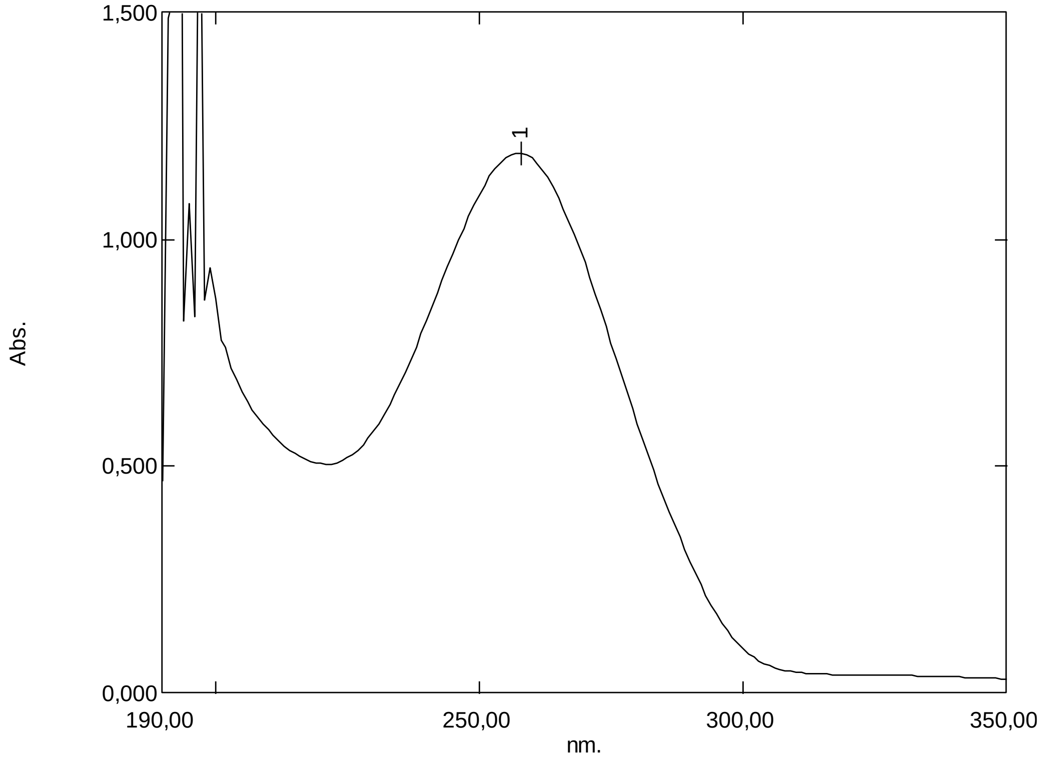

Figure S41. UV spectrum of compound **6**

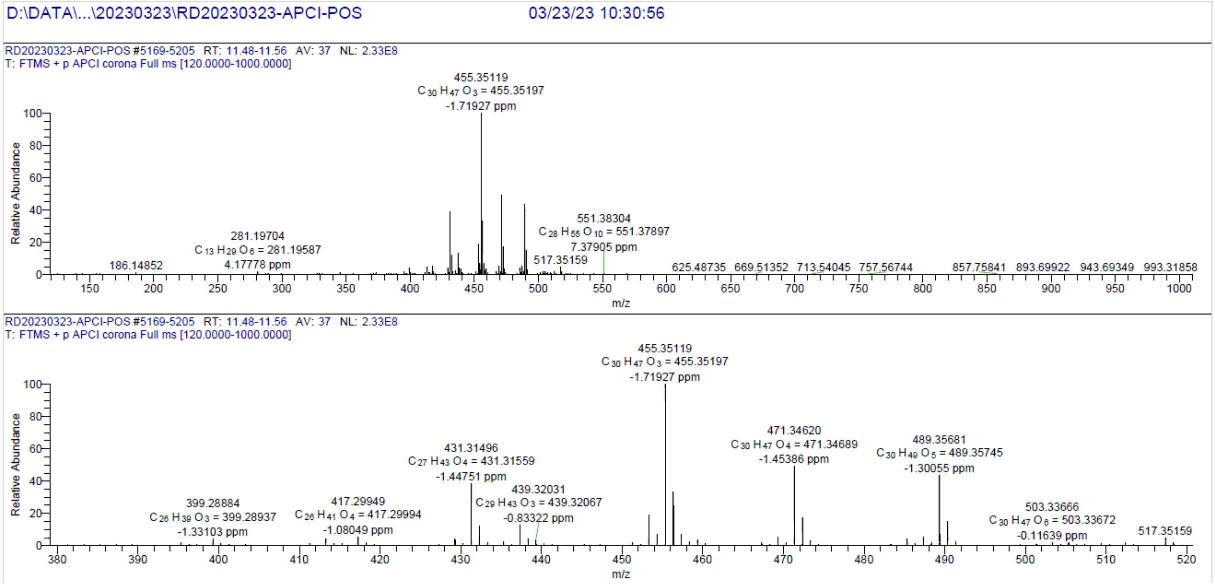

Figure S42. HR-APCI-MS spectrum of compound **6**

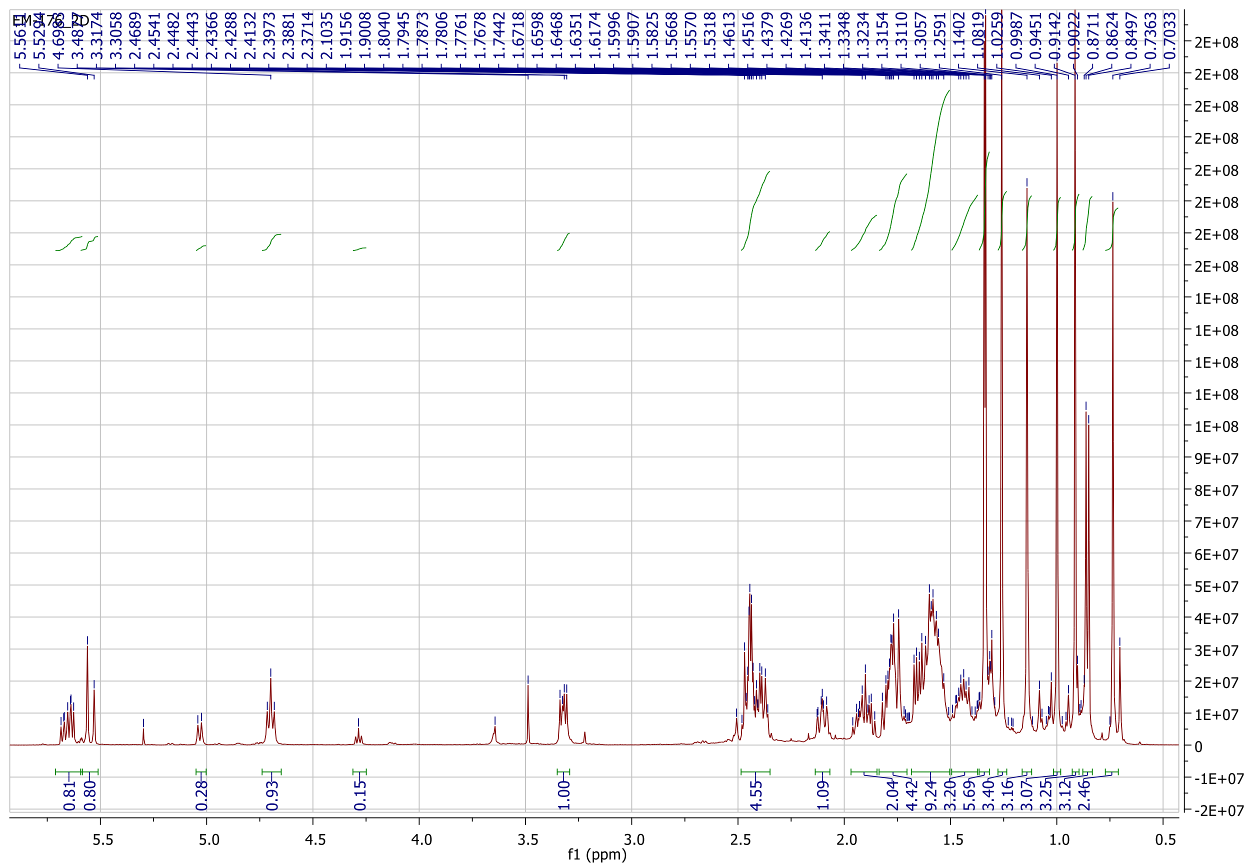

Figure S43. ^{1}H NMR spectrum of compound **6** (500 MHz, $CDCl_3$)

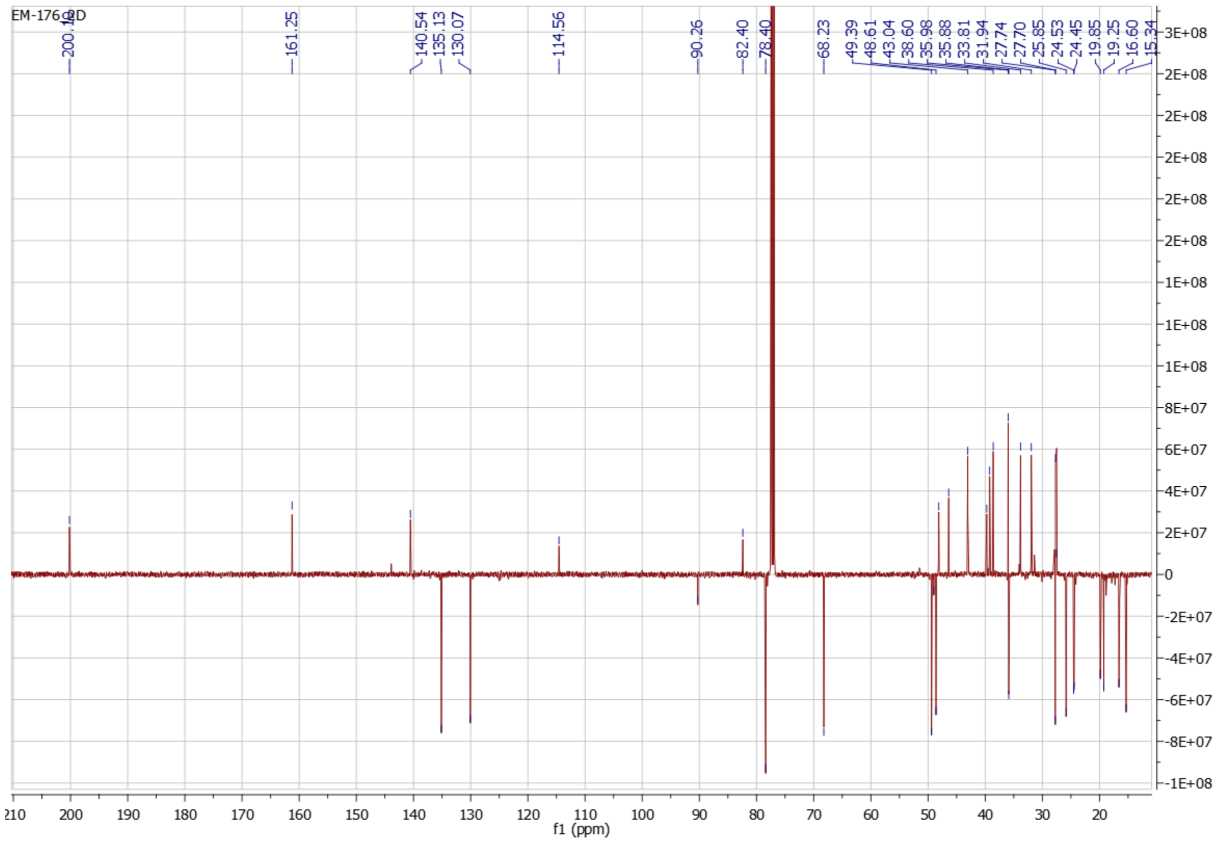

Figure S44. ^{13}C NMR JMOD spectrum of compound **6** (125 MHz, $CDCl_3$)

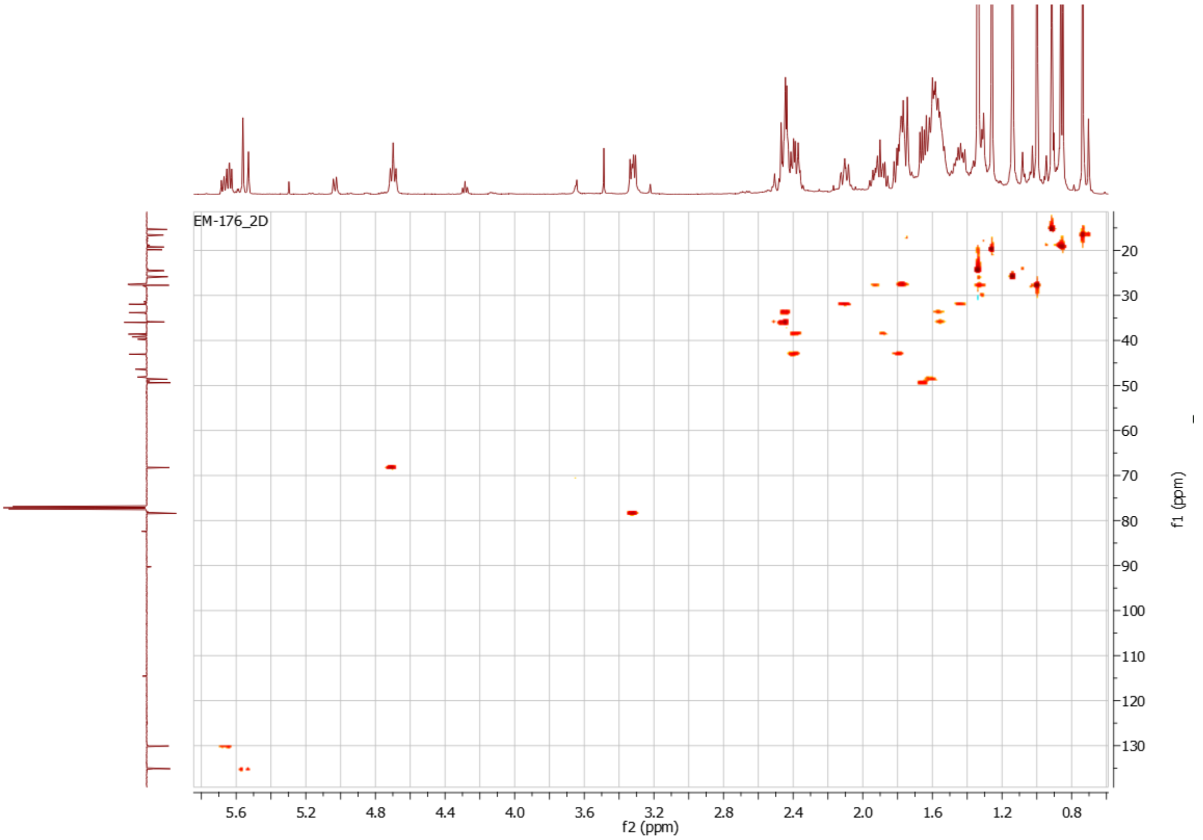

Figure S45. HSQC spectrum of compound **6**

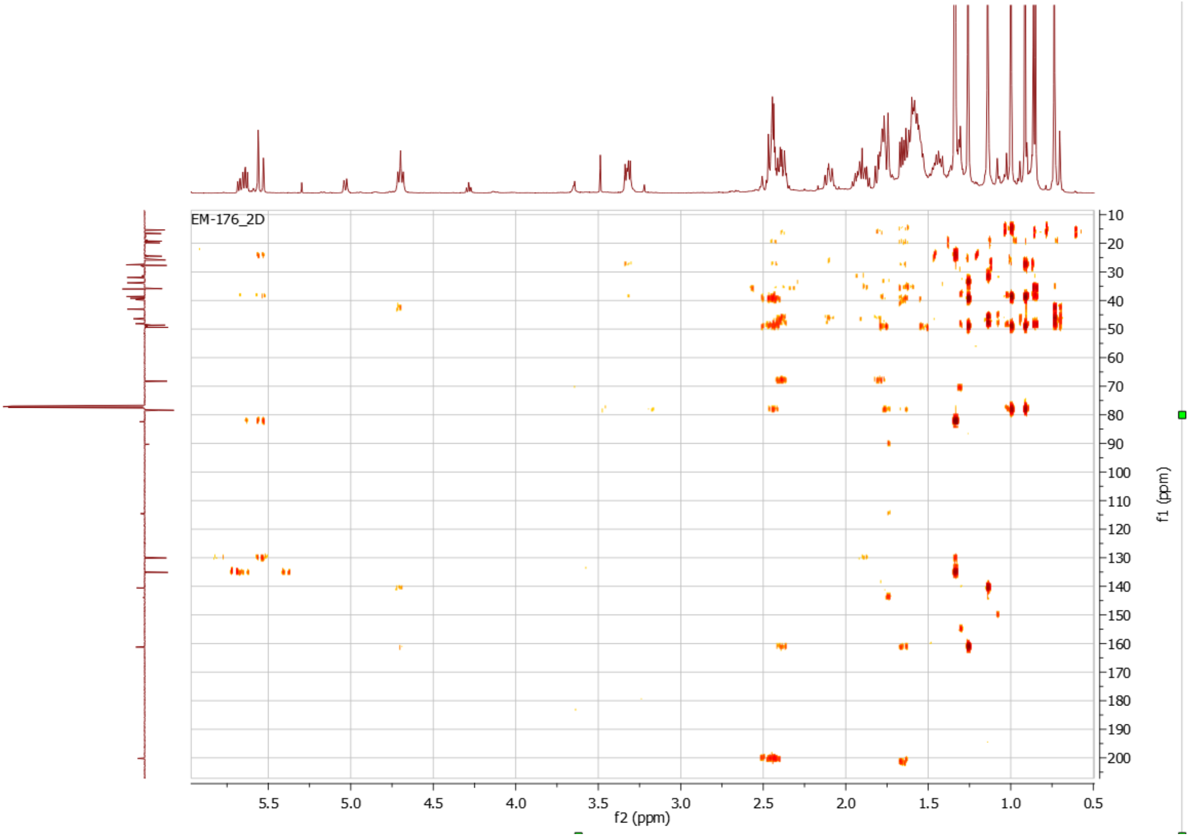

Figure S46. HMBC spectrum of compound **6**

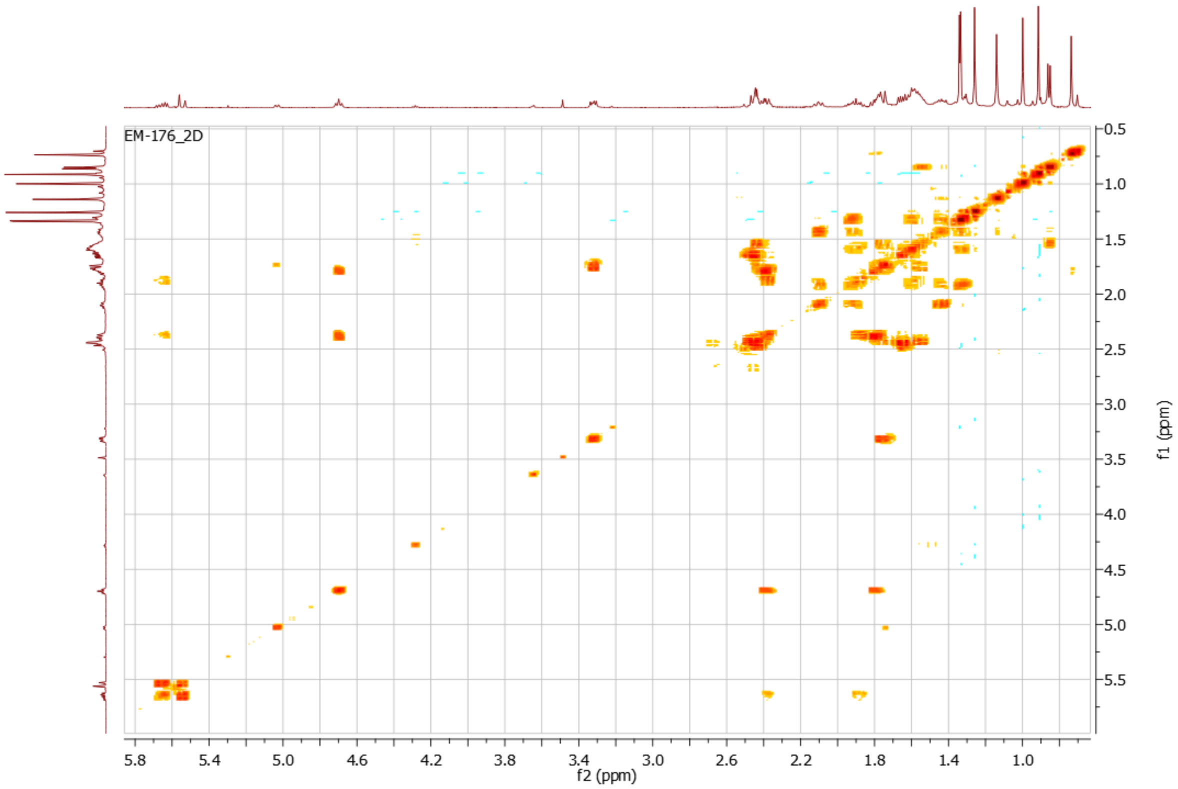

Figure S47. ^{1}H-^{1}H COSY spectrum of compound **6**

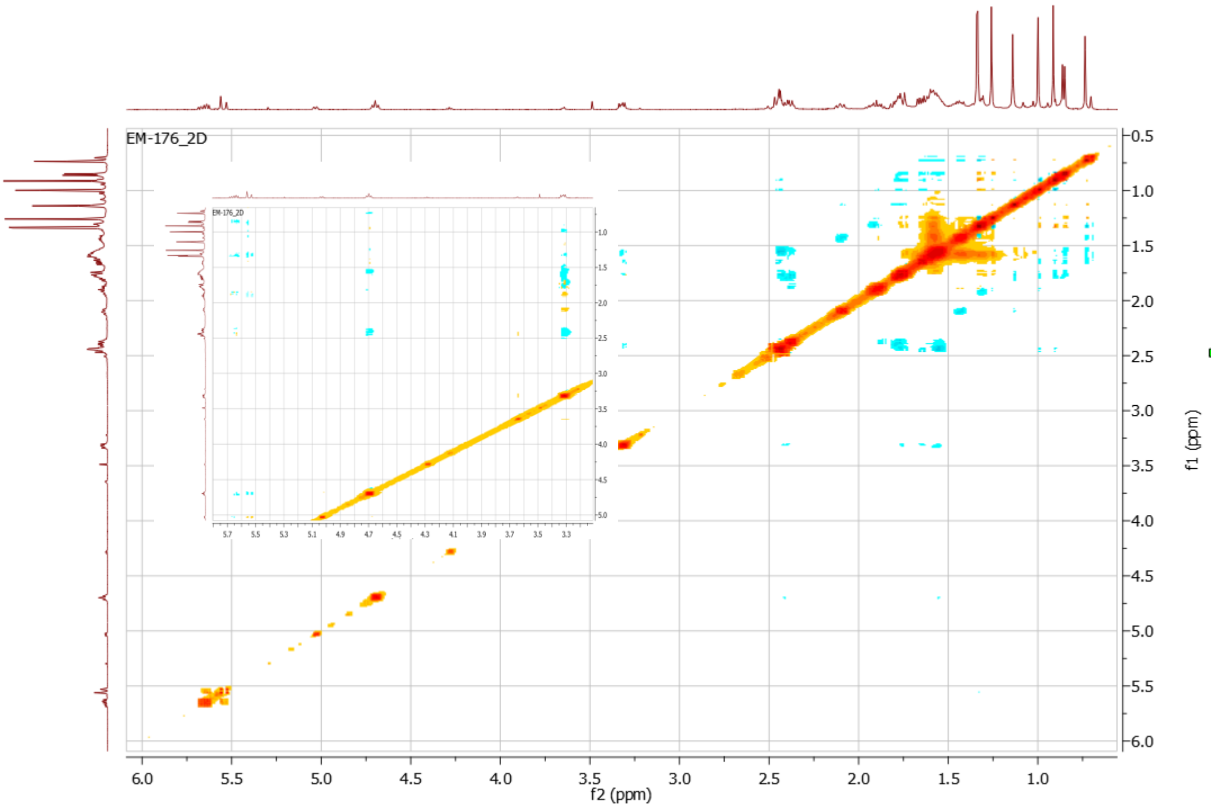

Figure S48. NOESY spectrum of compound **6**

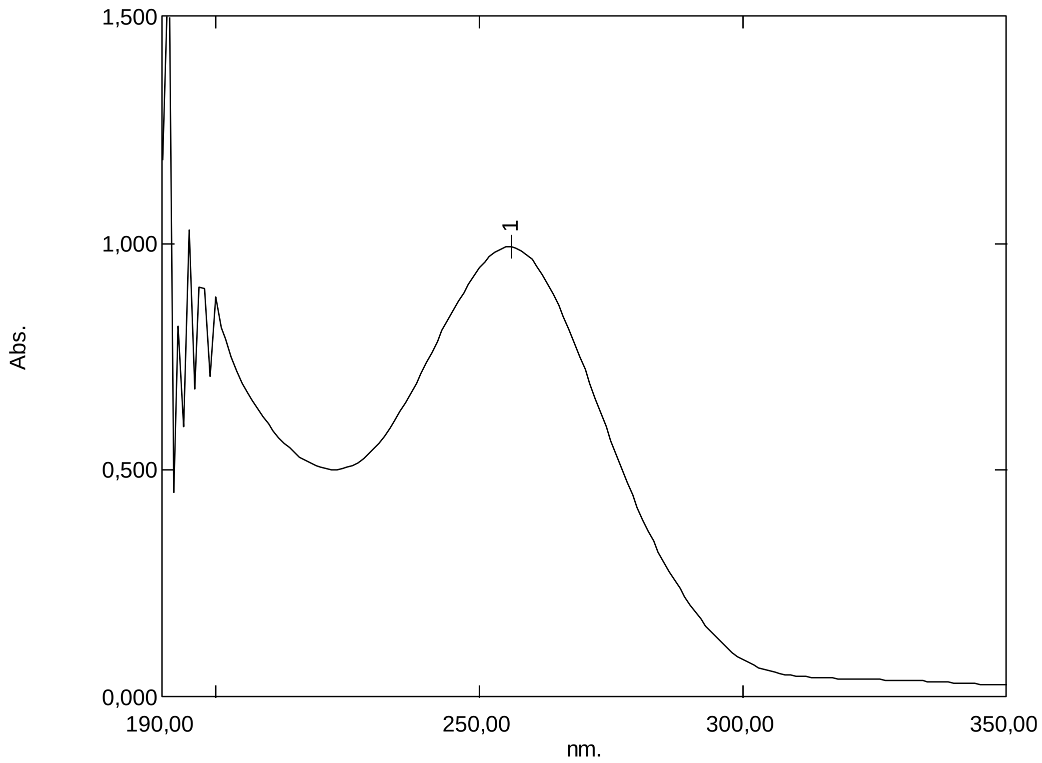

Figure S49 UV spectrum of compound **7**

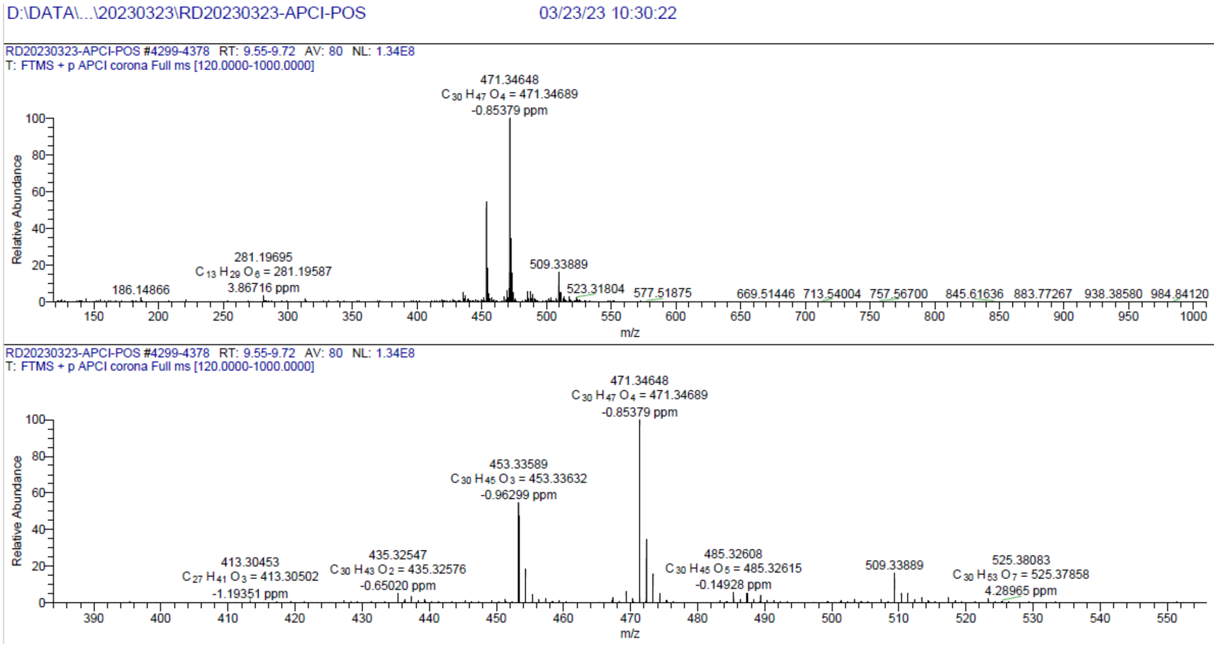

Figure S50. HR-APCI-MS spectrum of compound **7**

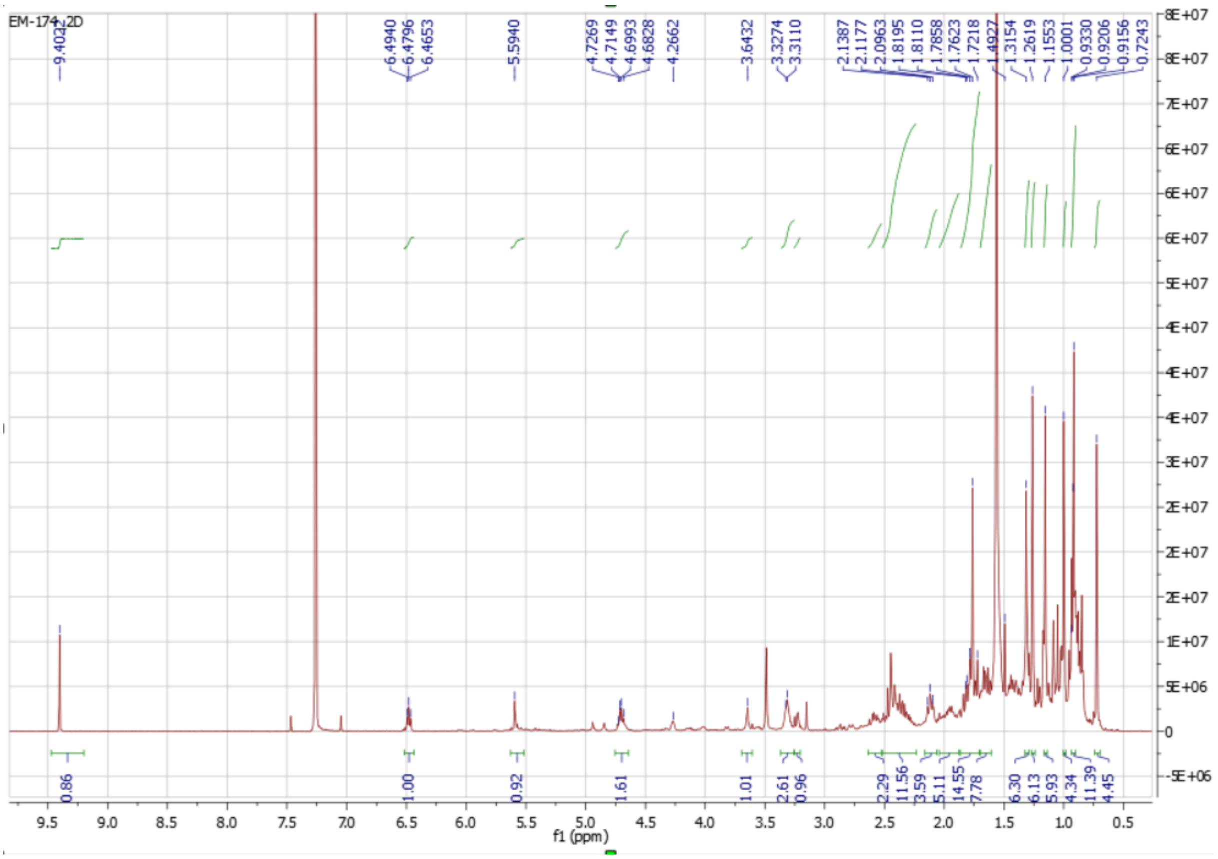

Figure S51. ^{1}H NMR spectrum of compound **7** (500 MHz, $CDCl_3$)

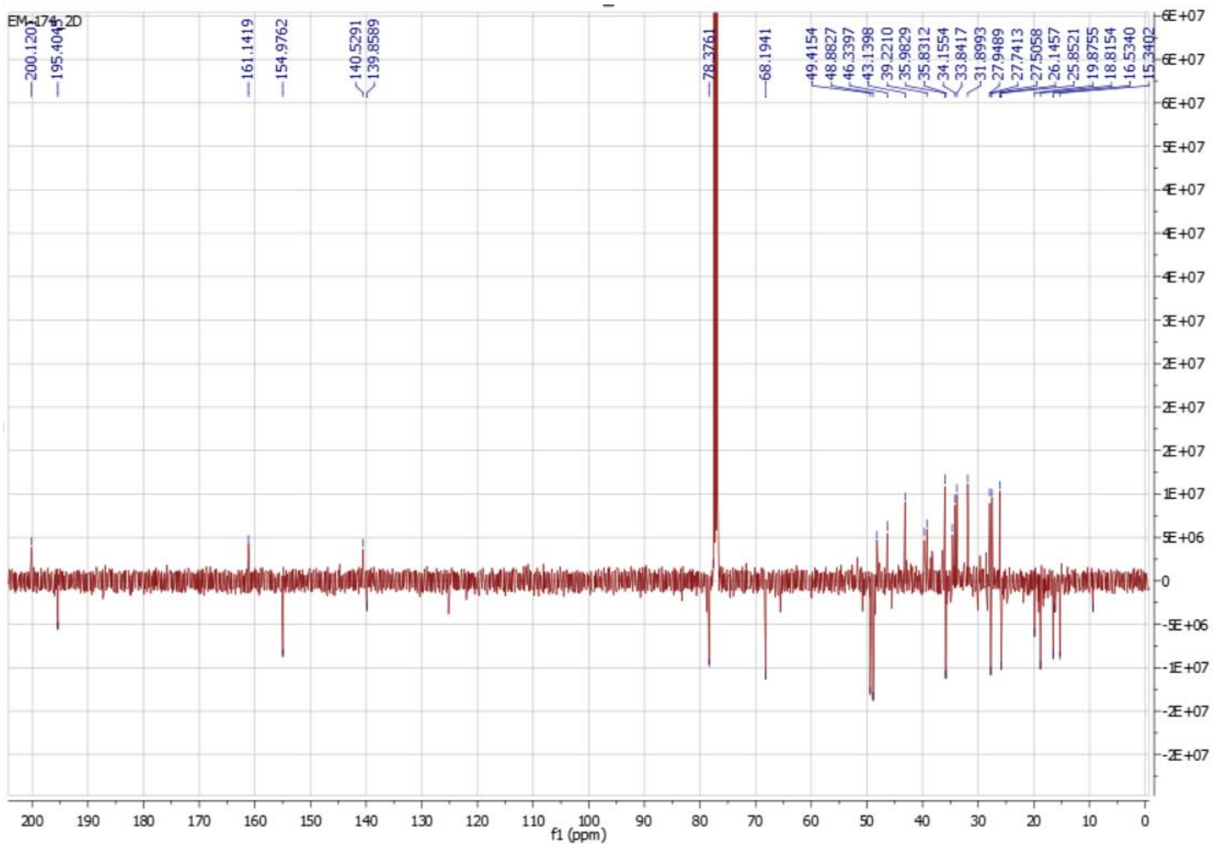

Figure S52. ^{13}C NMR JMOD spectrum of compound **7** (125 MHz, $CDCl_3$)

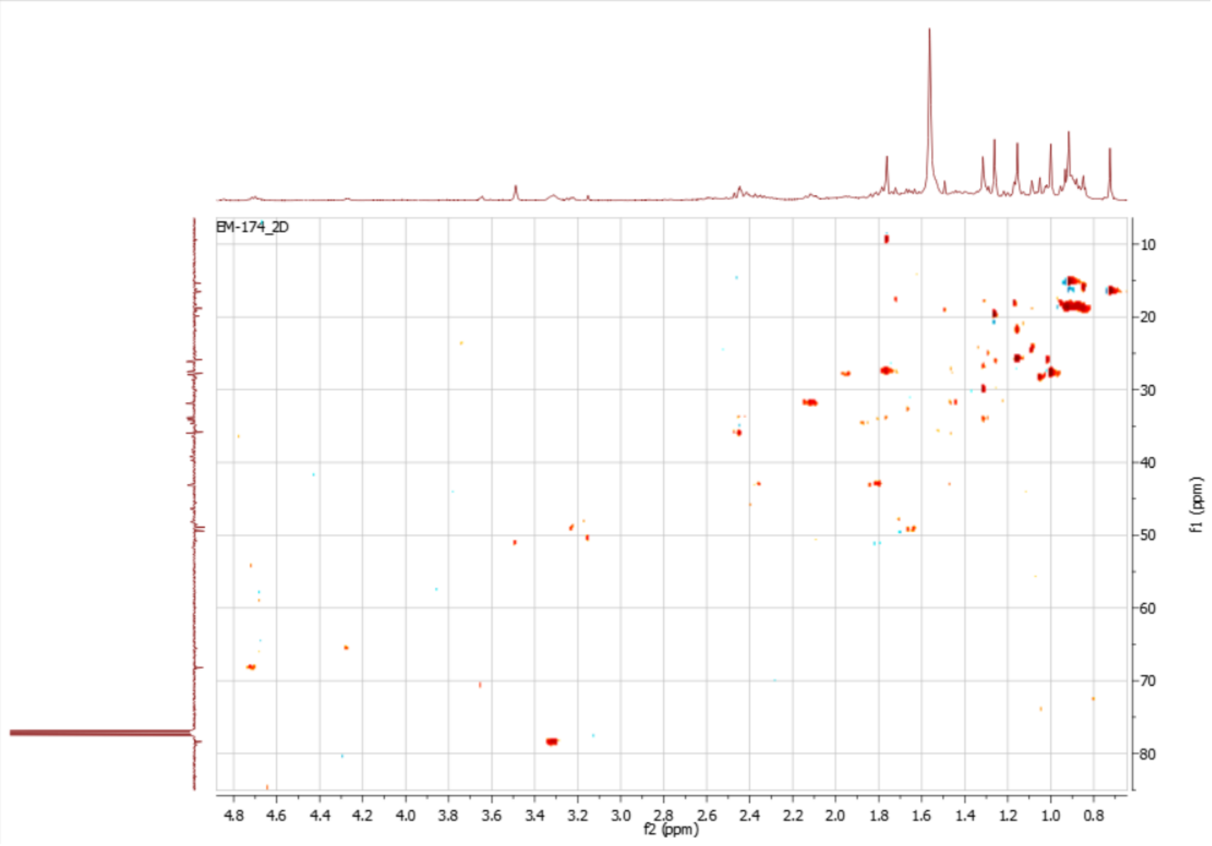

Figure S53. HSQC spectrum of compound **7**

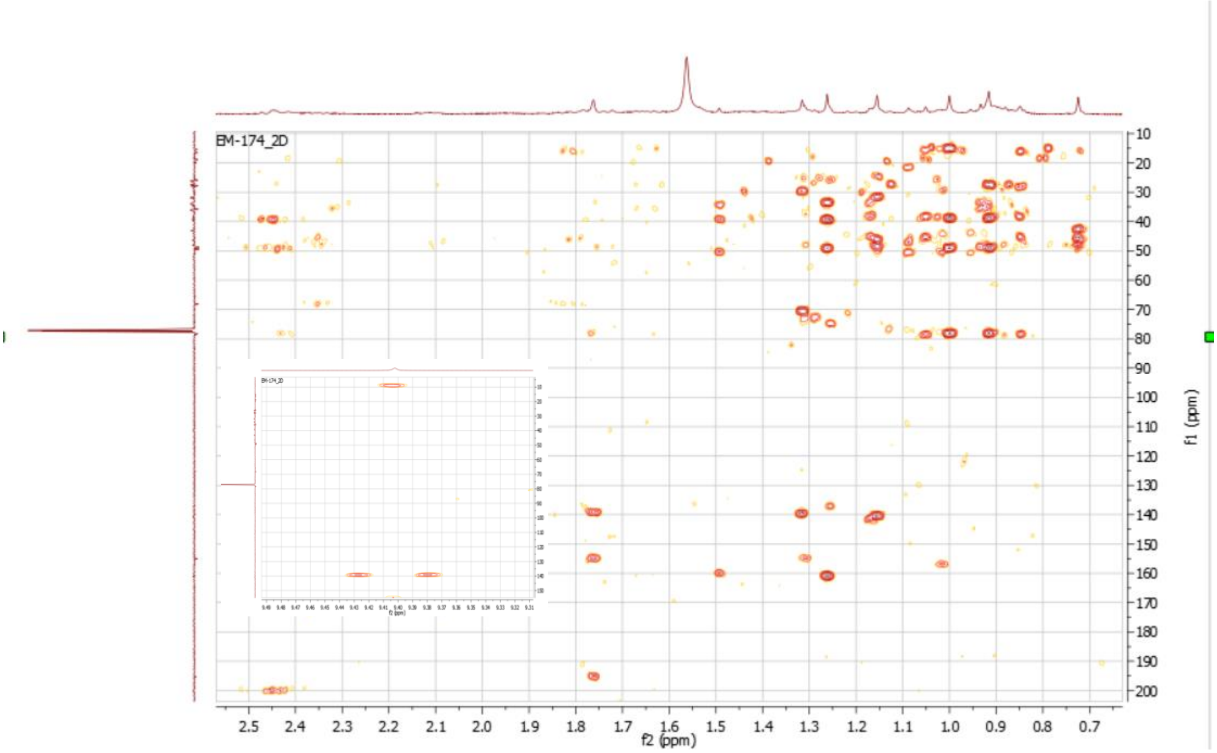

Figure S54. HMBC spectrum of compound **7**

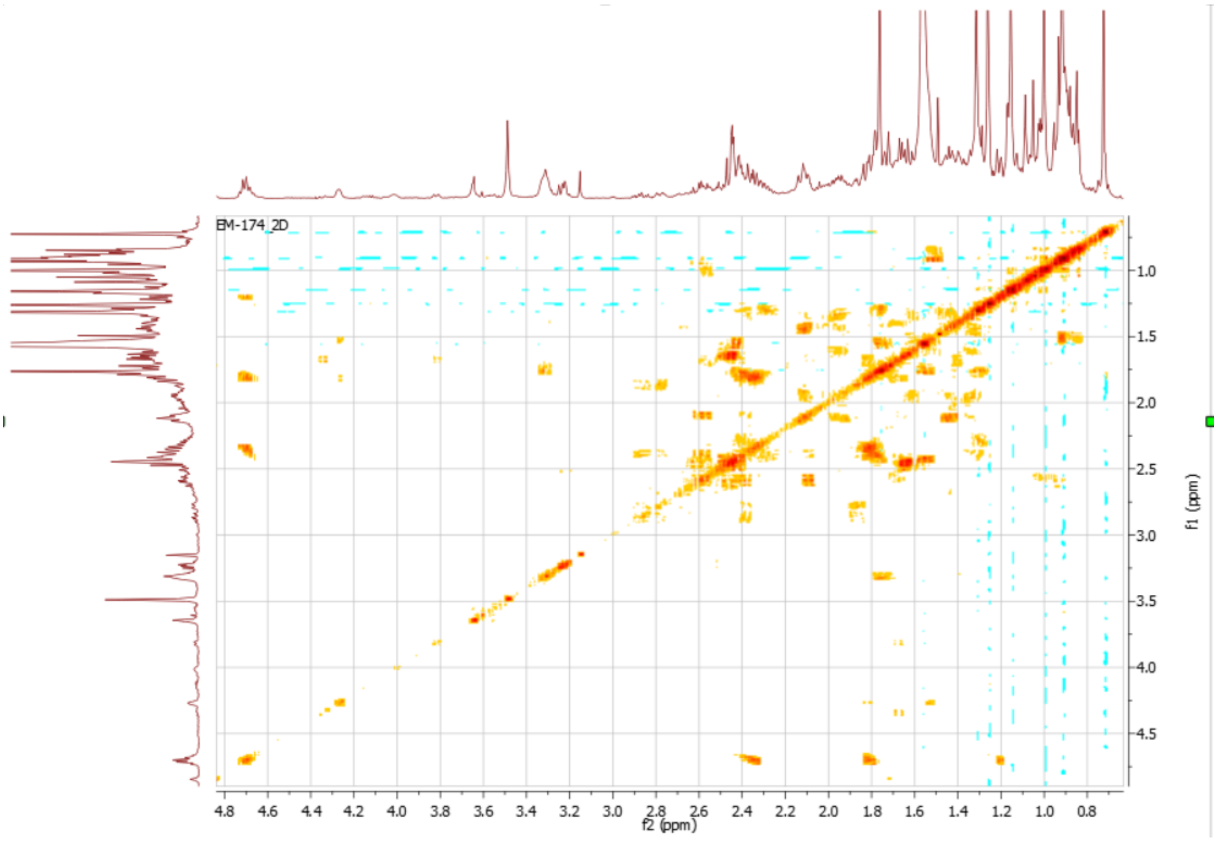

Figure S55. ^{1}H-^{1}H COSY spectrum of compound **7**

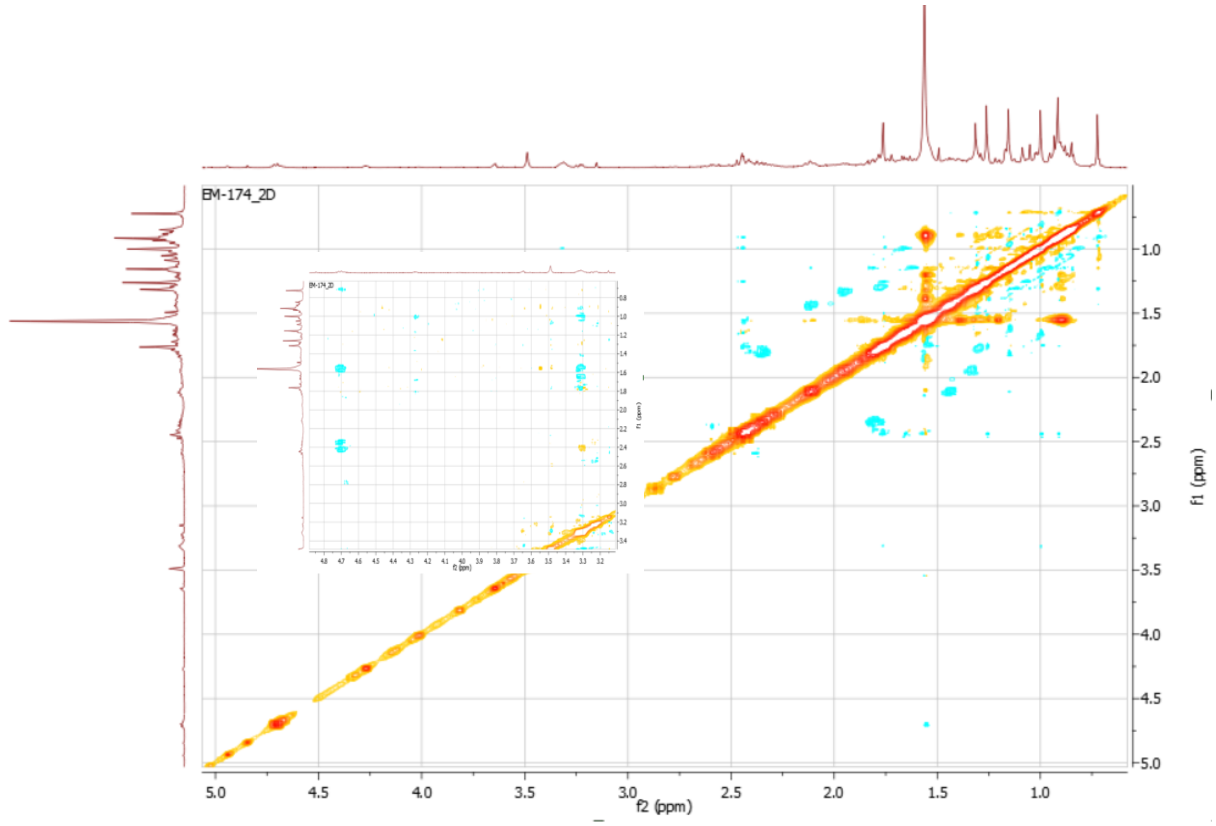

Figure S56. NOESY spectrum of compound **7**

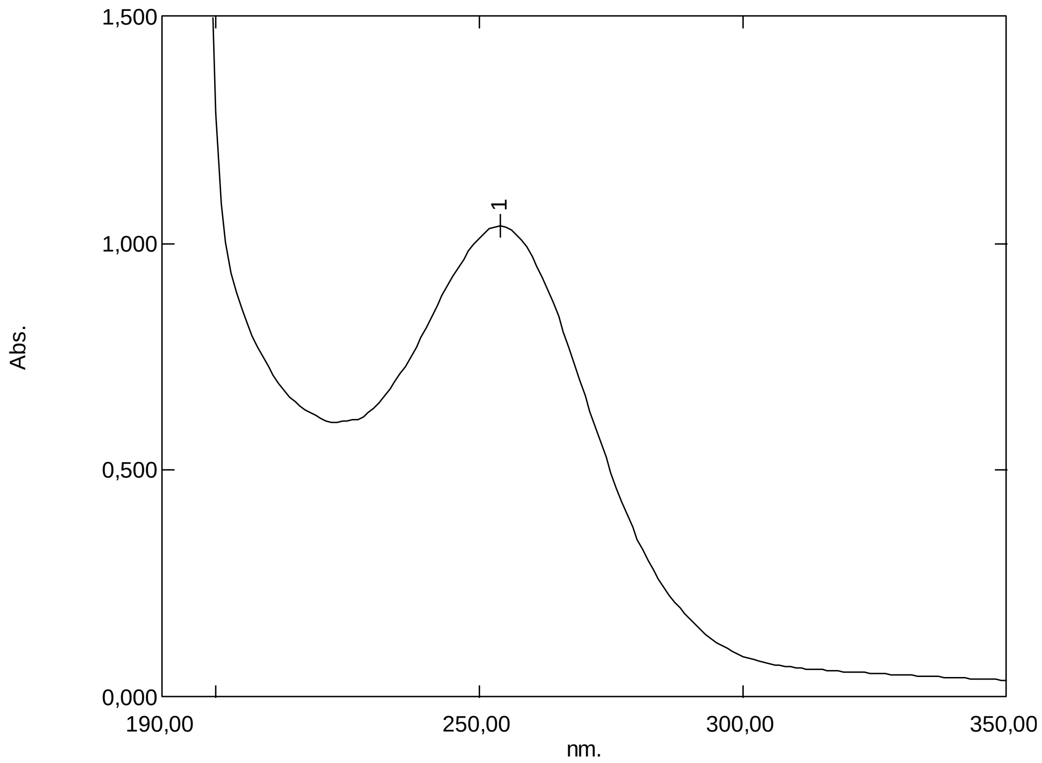

Figure S57. UV spectrum of compound **8**

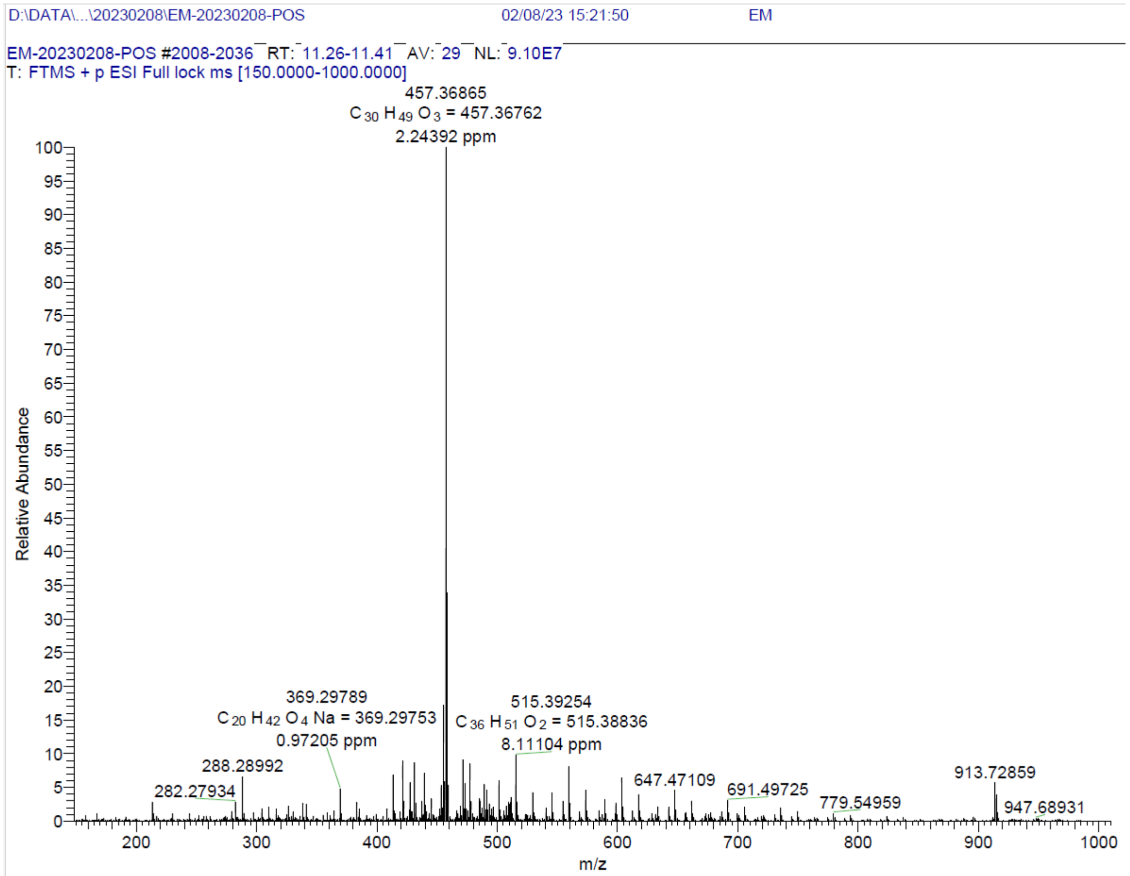

Figure S58. HRESIMS spectrum of compound **8**

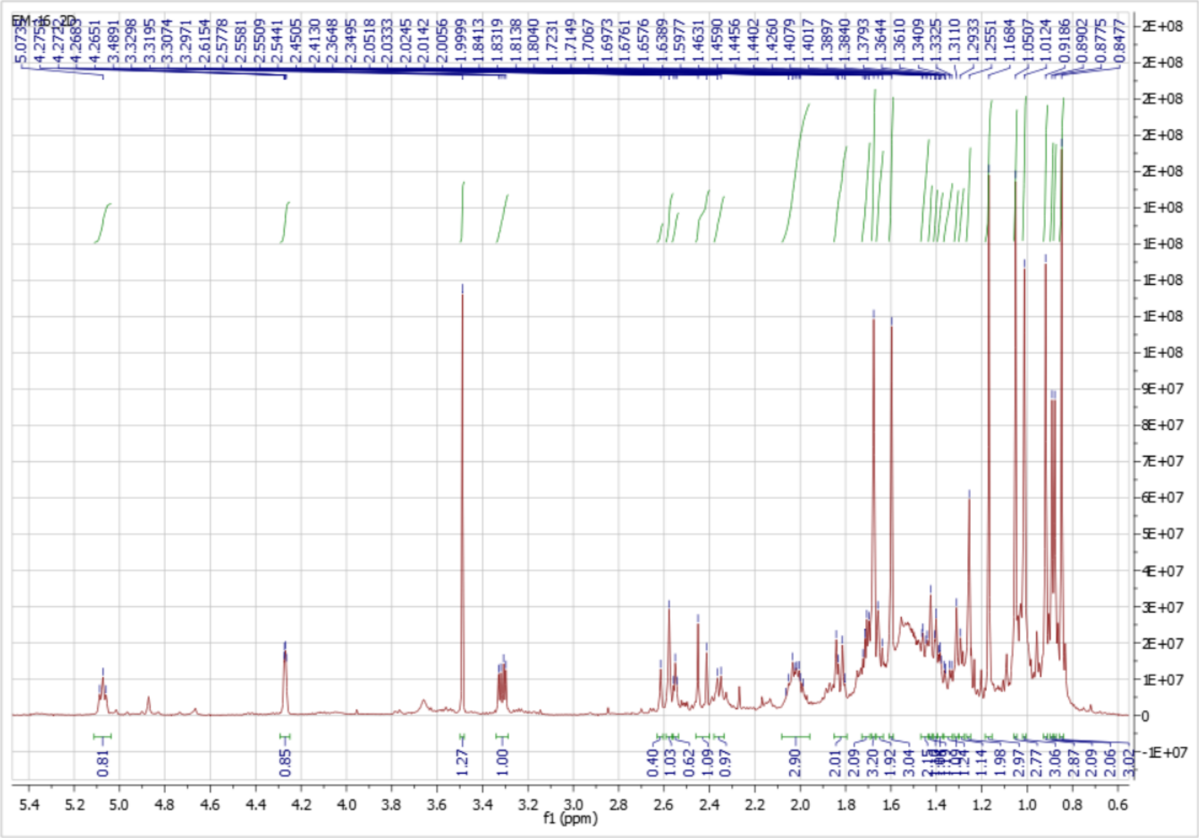

Figure S59. ^{1}H NMR spectrum of compound **8** (500 MHz, $CDCl_3$)

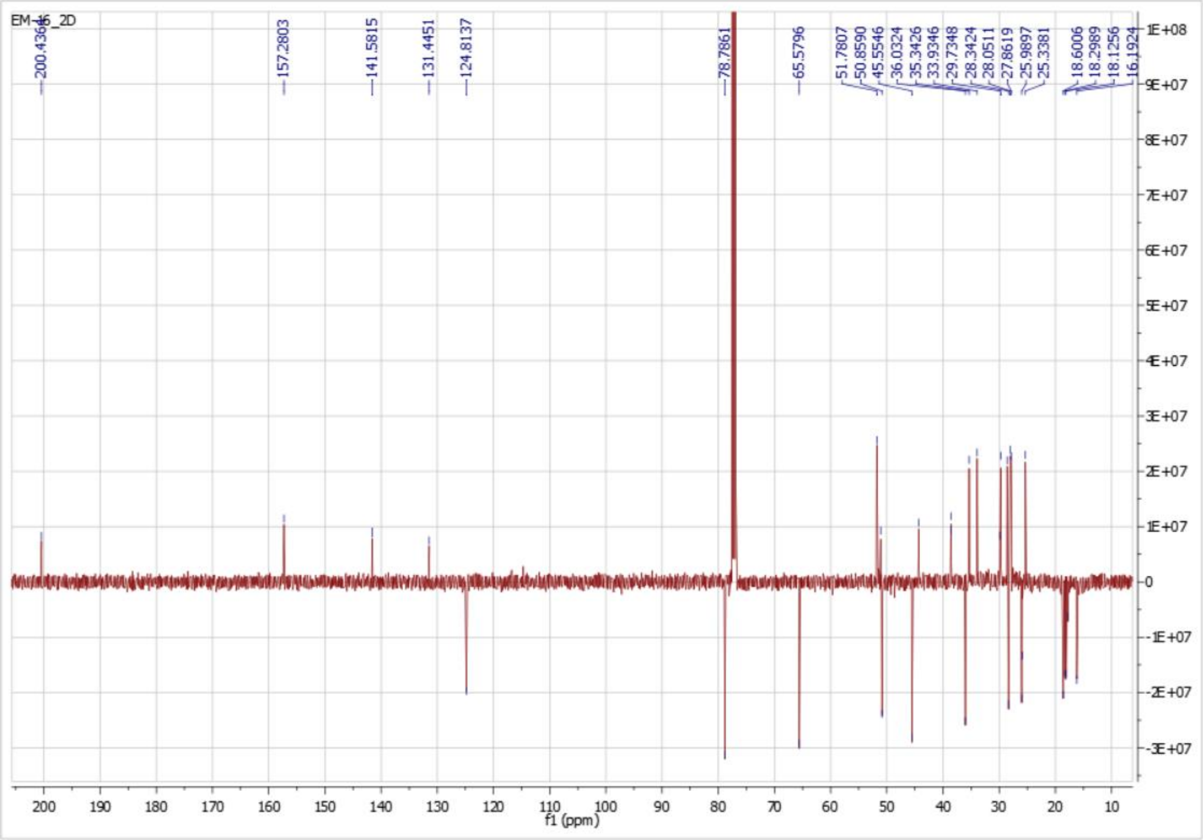

Figure S60. ^{13}C NMR JMOD spectrum of compound **8** (125 MHz, $CDCl_3$)

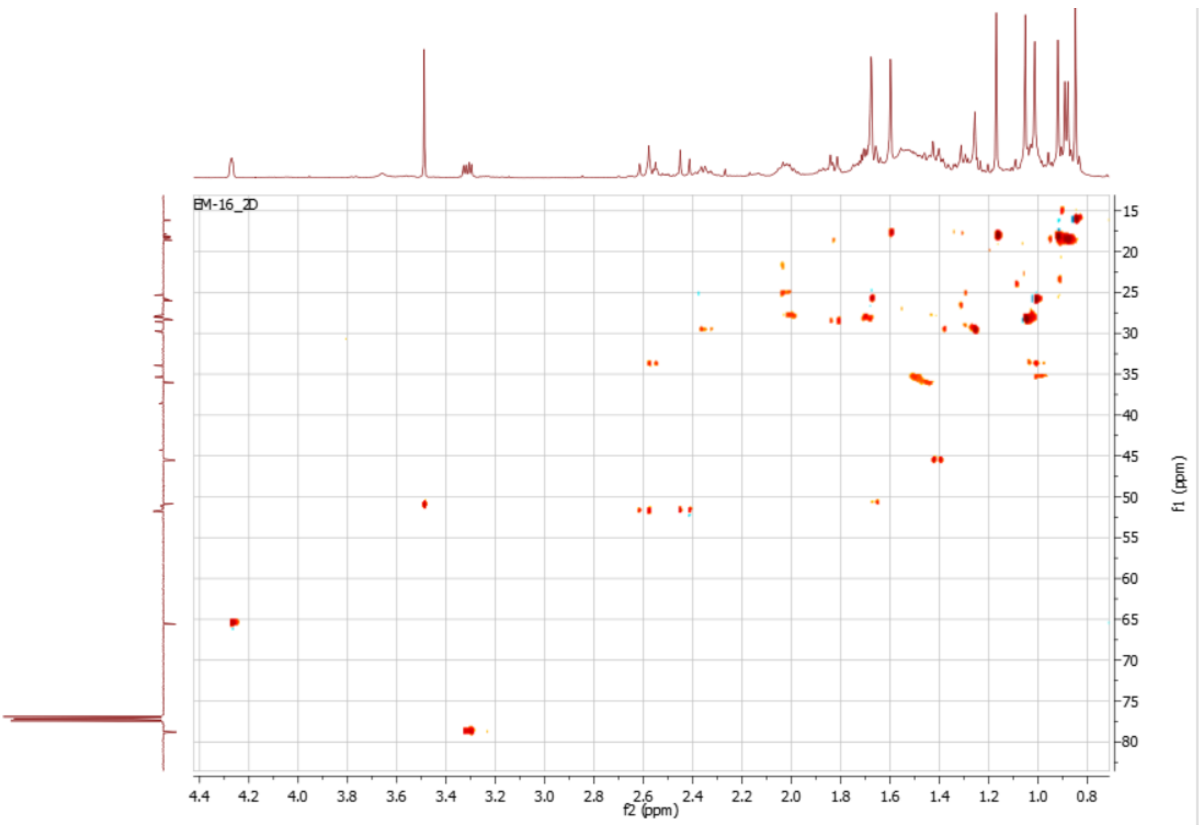

Figure S61. HSQC spectrum of compound **8**

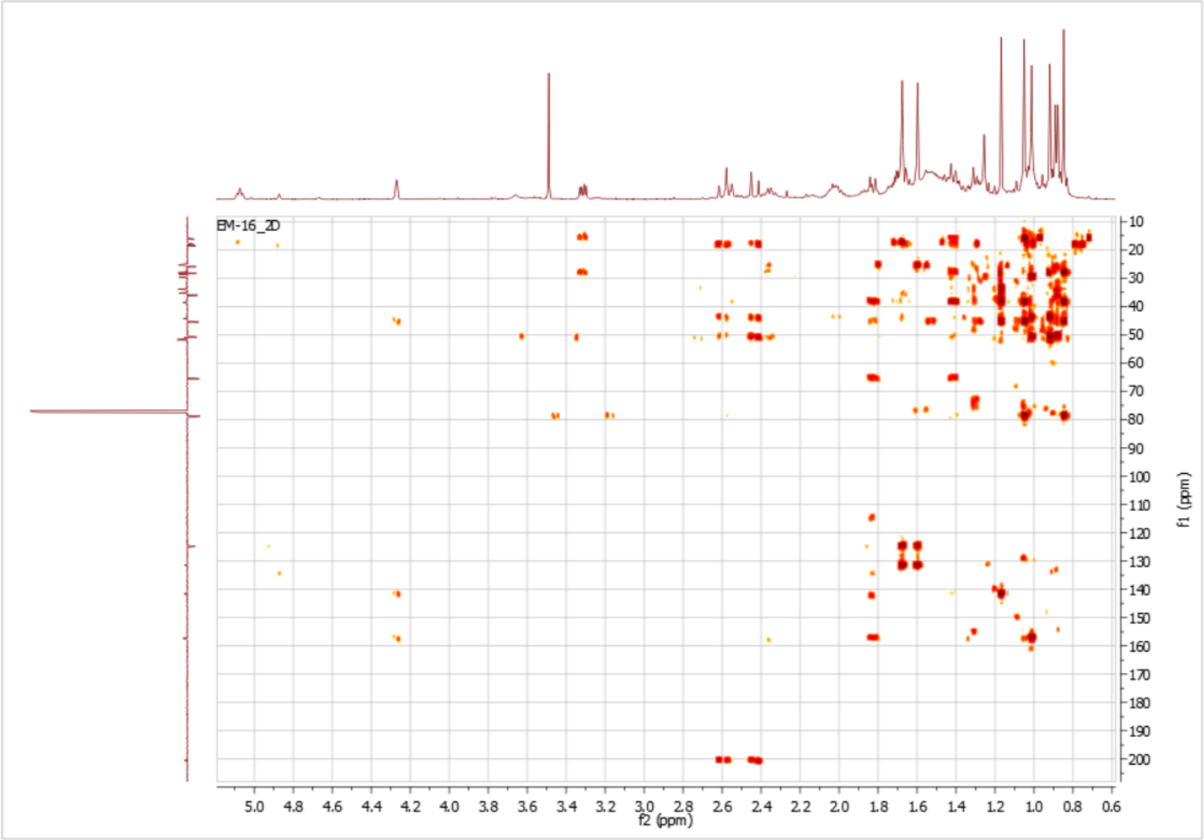

Figure S62. HMBC spectrum of compound **9**

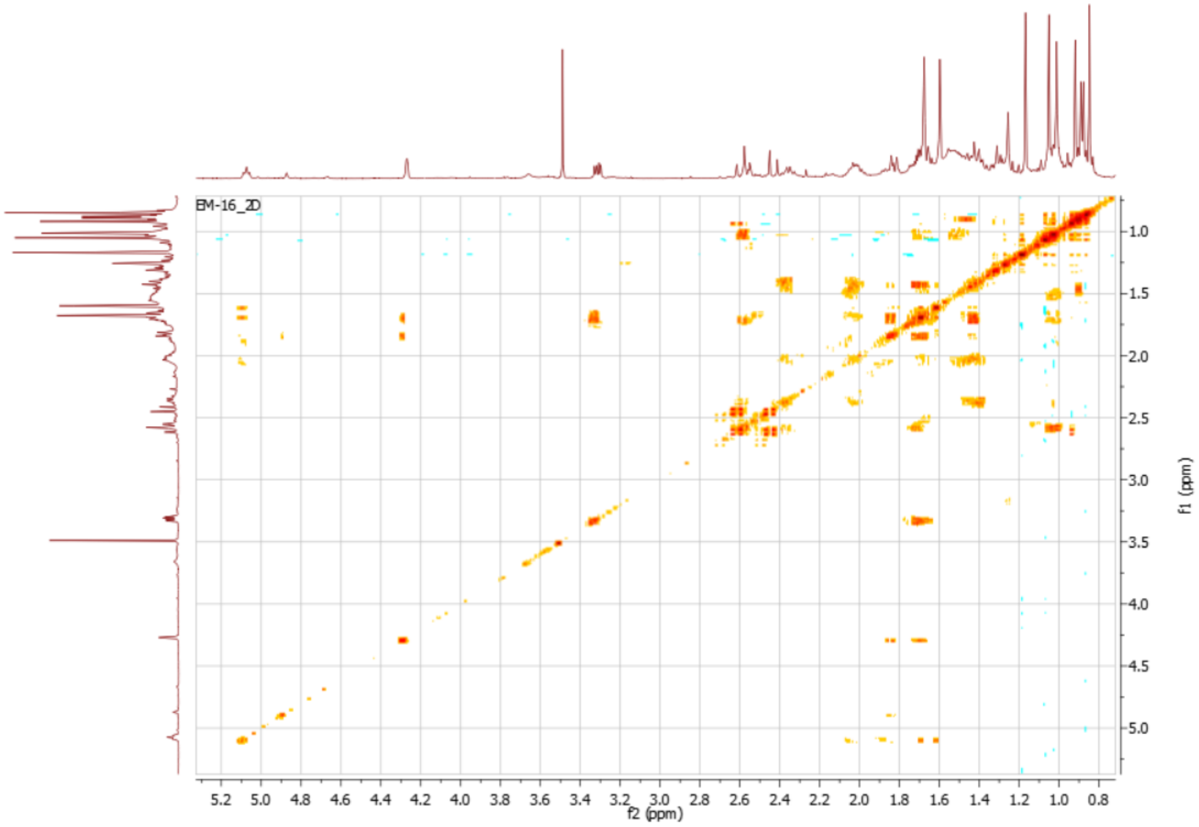

Figure S63. ^{1}H-^{1}H COSY spectrum of compound **8**

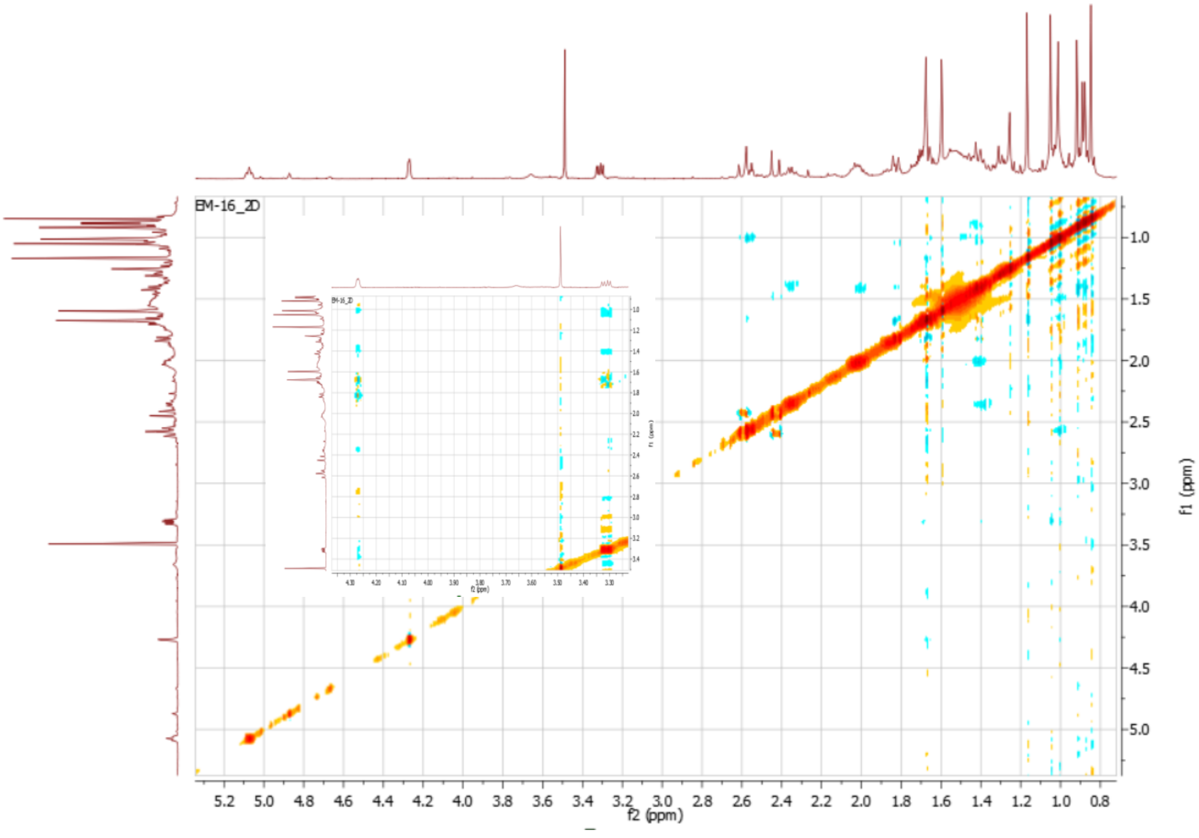

Figure S64. NOESY spectrum of compound **8**

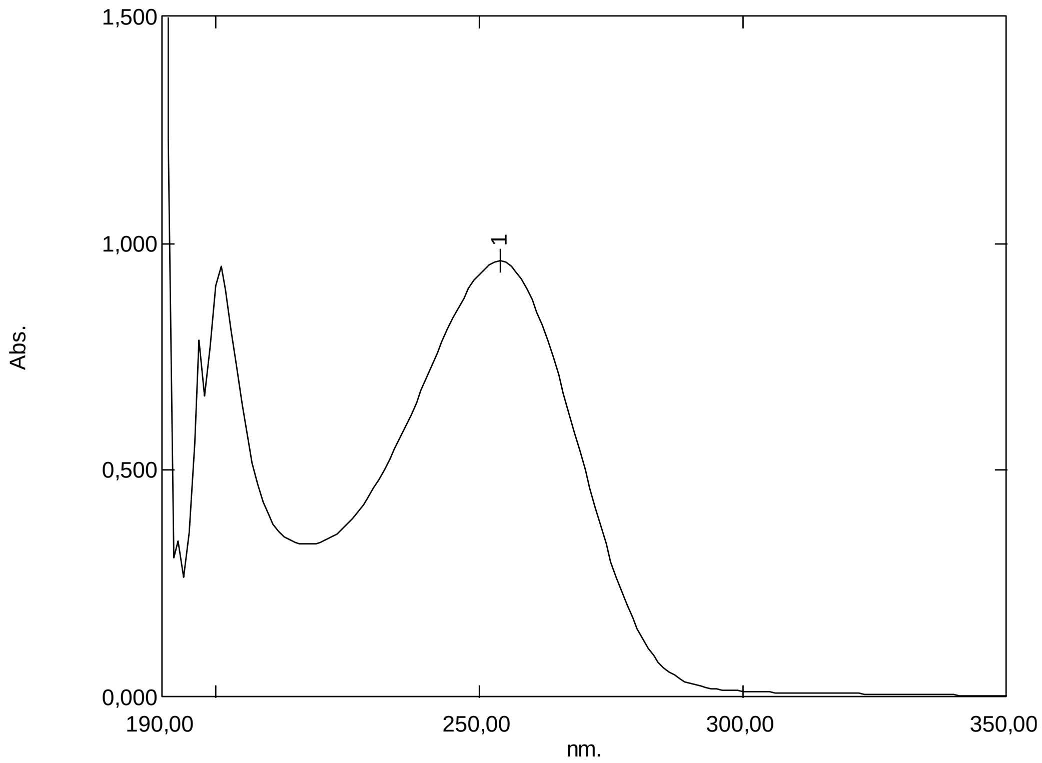

Figure S65. UV spectrum of compound **9**

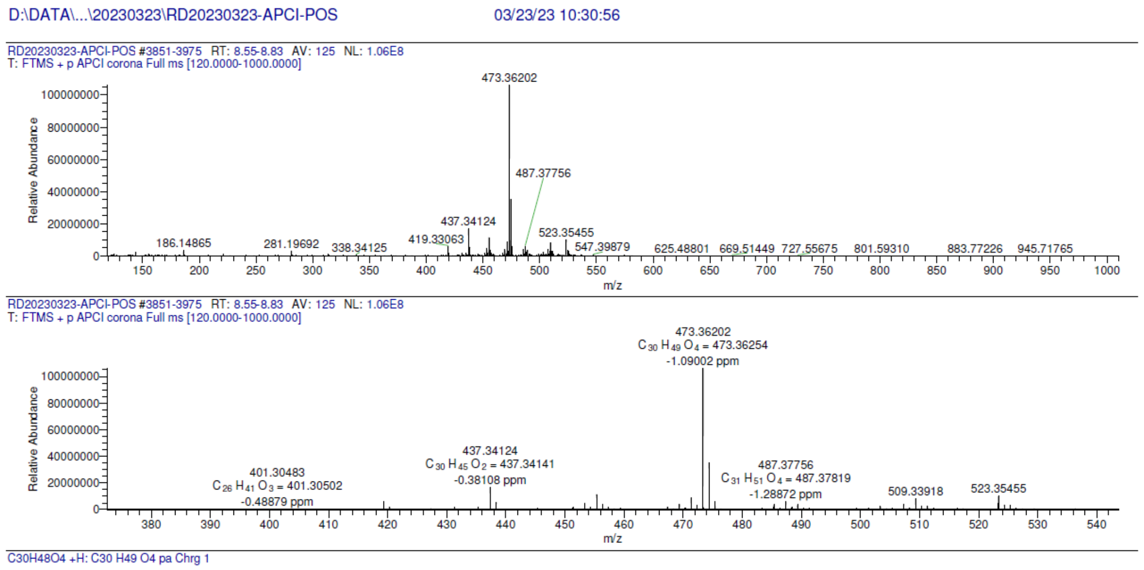

Figure S66. HRESIMS spectrum of compound **9**

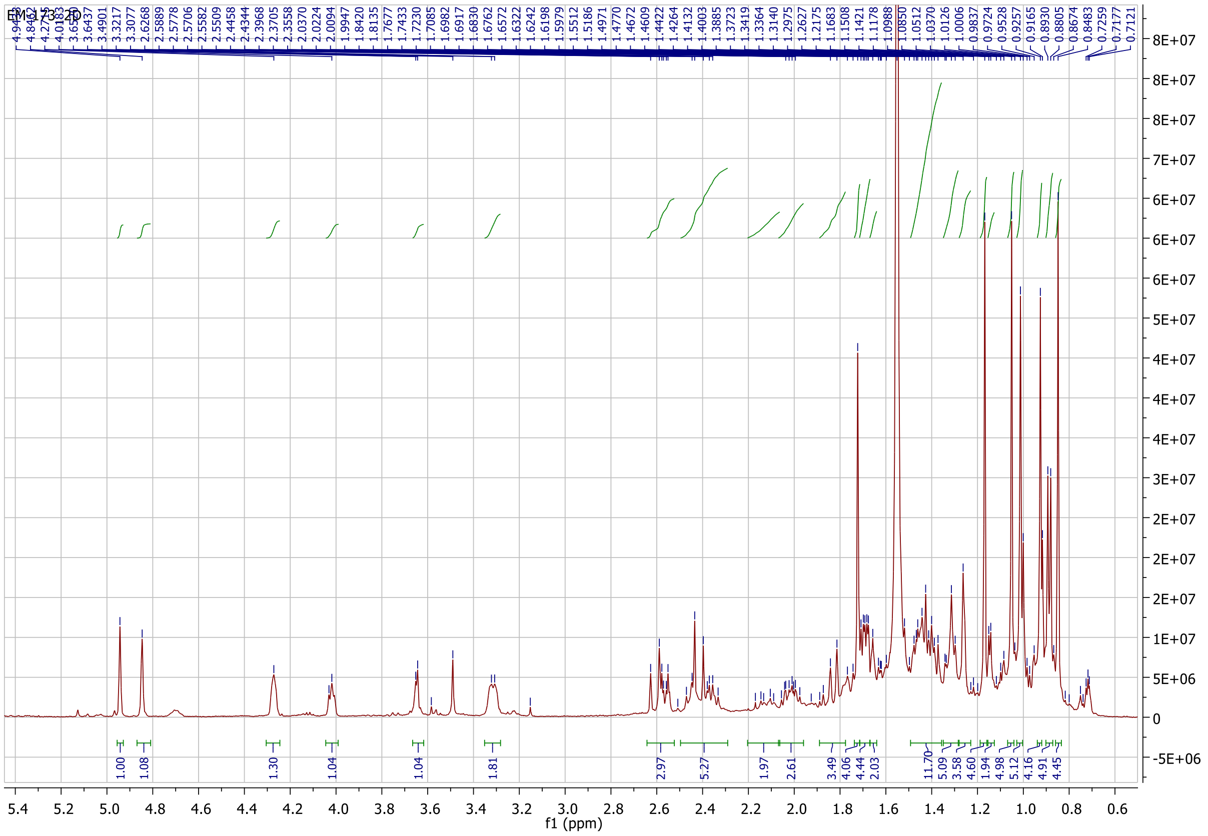

Figure S67. ^{1}H NMR spectrum of compound **9** (500 MHz, $CDCl_3$)

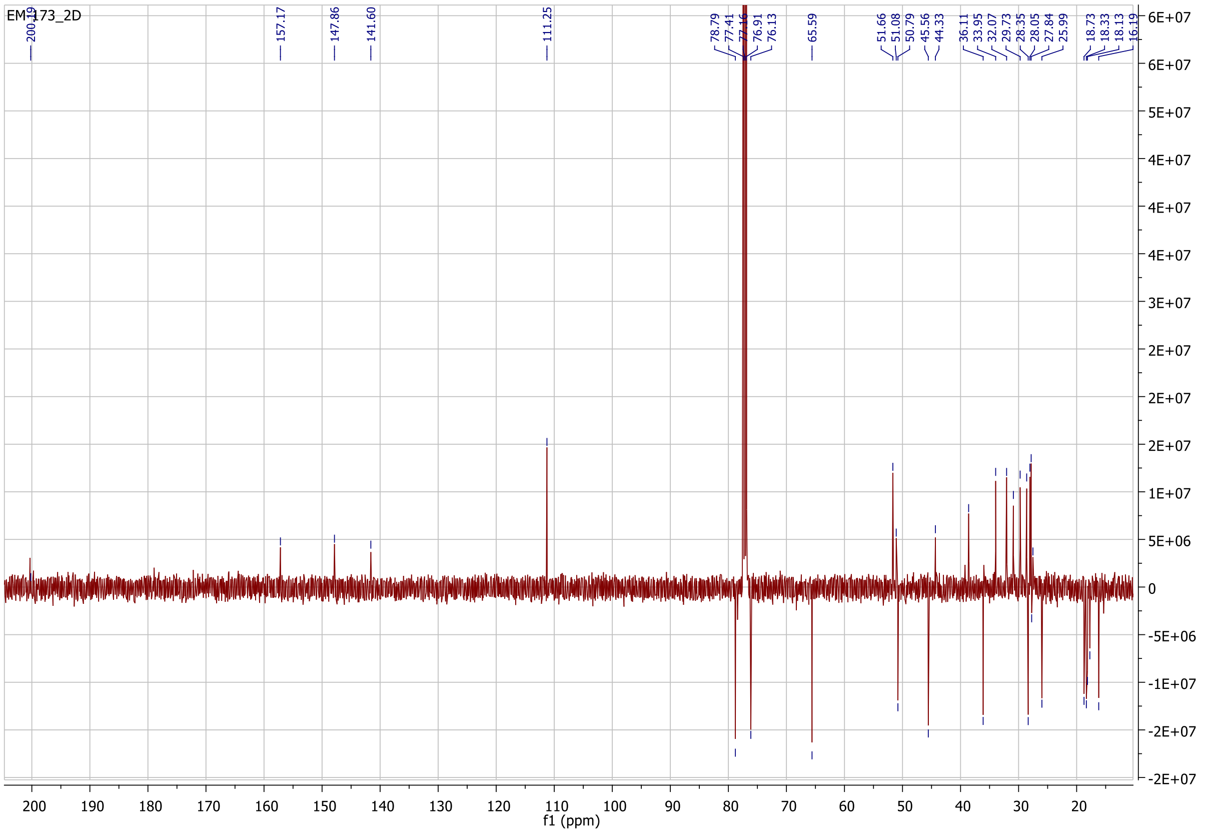

Figure S68. ^{13}C NMR JMOD spectrum of compound **9** (125 MHz, $CDCl_3$)

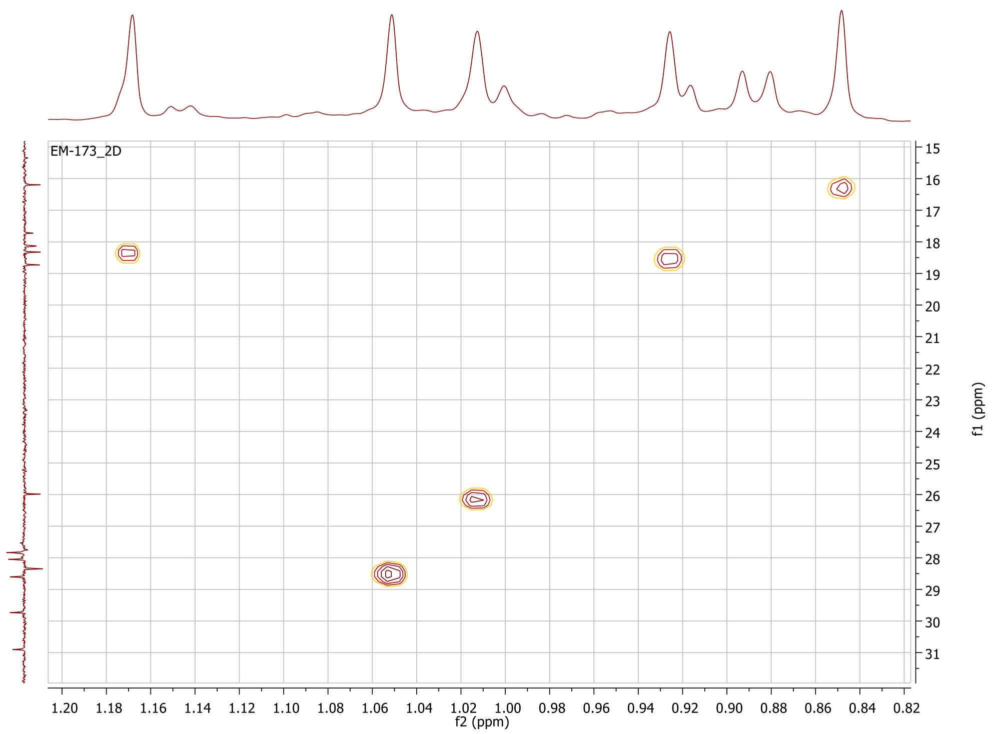

Figure S69. HSQC spectrum of compound **9**

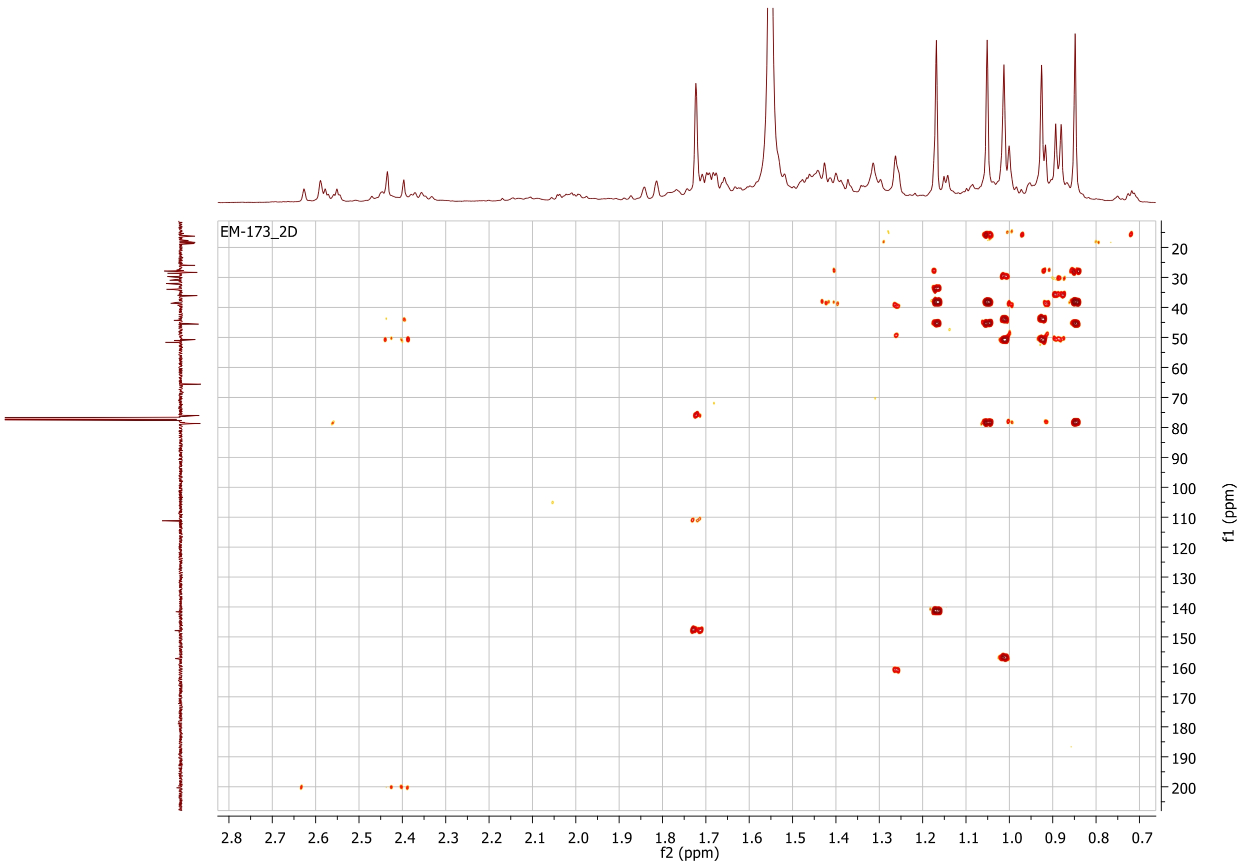

Figure S70. HMBC spectrum of compound **9**

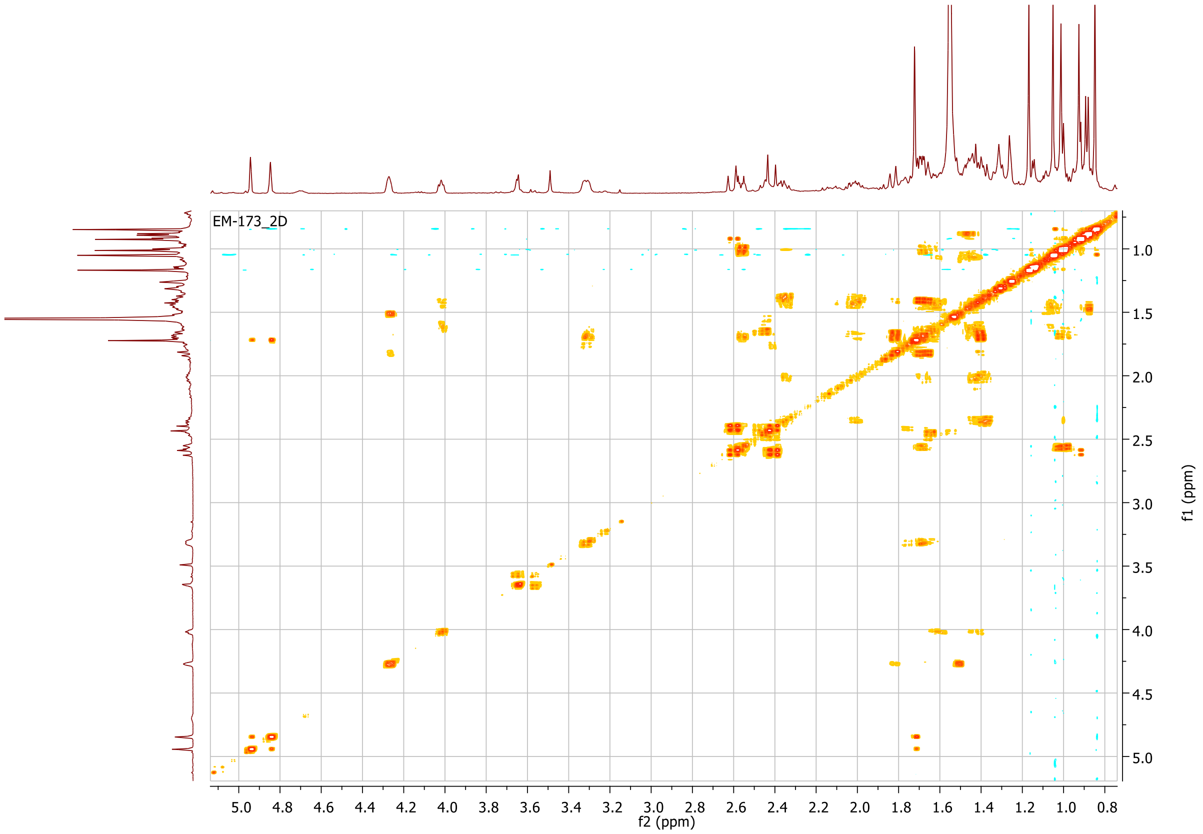

Figure S71. ^{1}H-^{1}H COSY spectrum of compound **9**

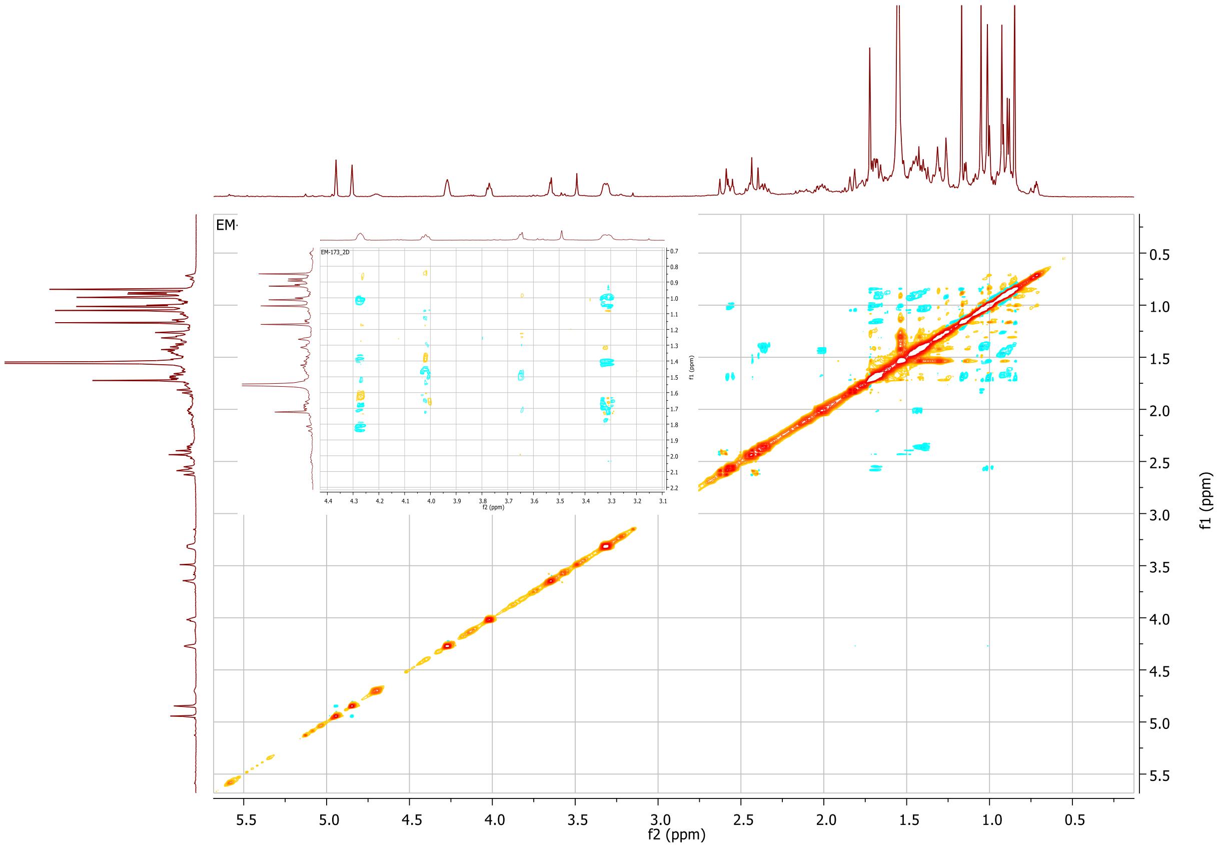

Figure S72. NOESY spectrum of compound **9**

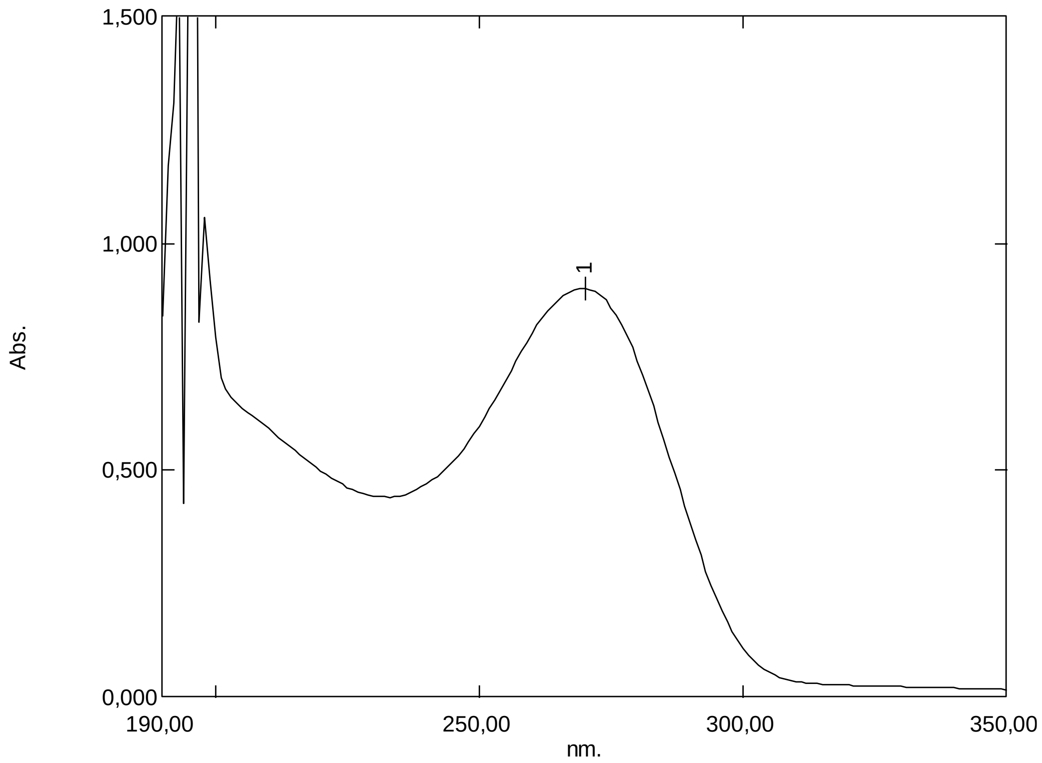

Figure S73. UV spectrum of compound **10**

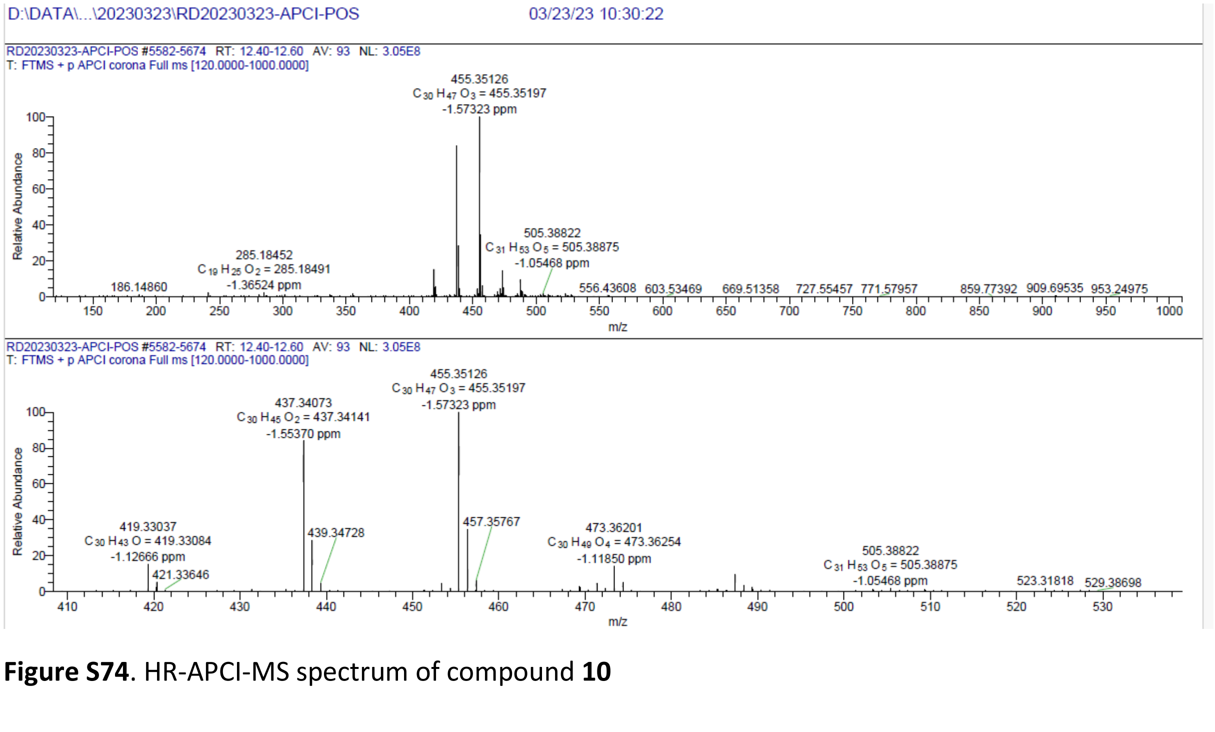

Figure S74. HR-APCI-MS spectrum of compound **10**

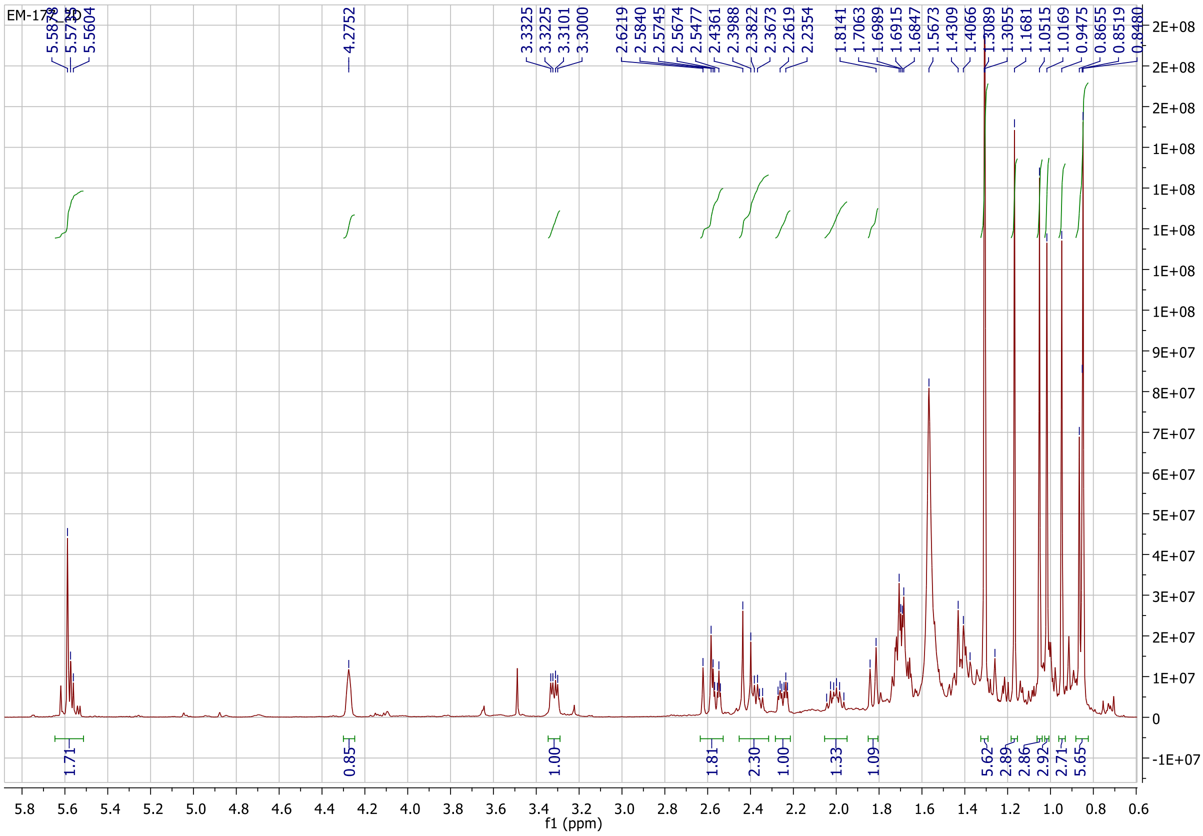

Figure S75. ^{1}H NMR spectrum of compound **10** (500 MHz, $CDCl_3$)

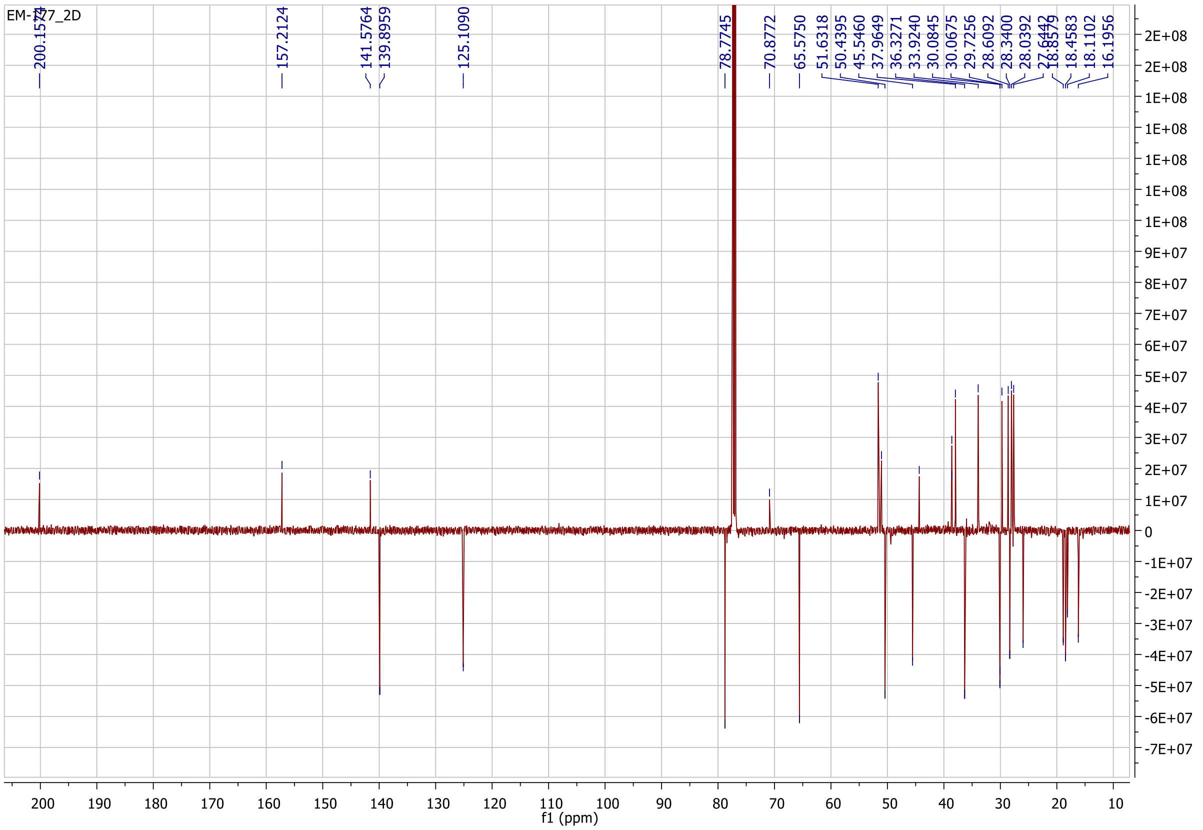

Figure S76. ^{13}C NMR JMOD spectrum of compound **10** (125 MHz, $CDCl_3$)

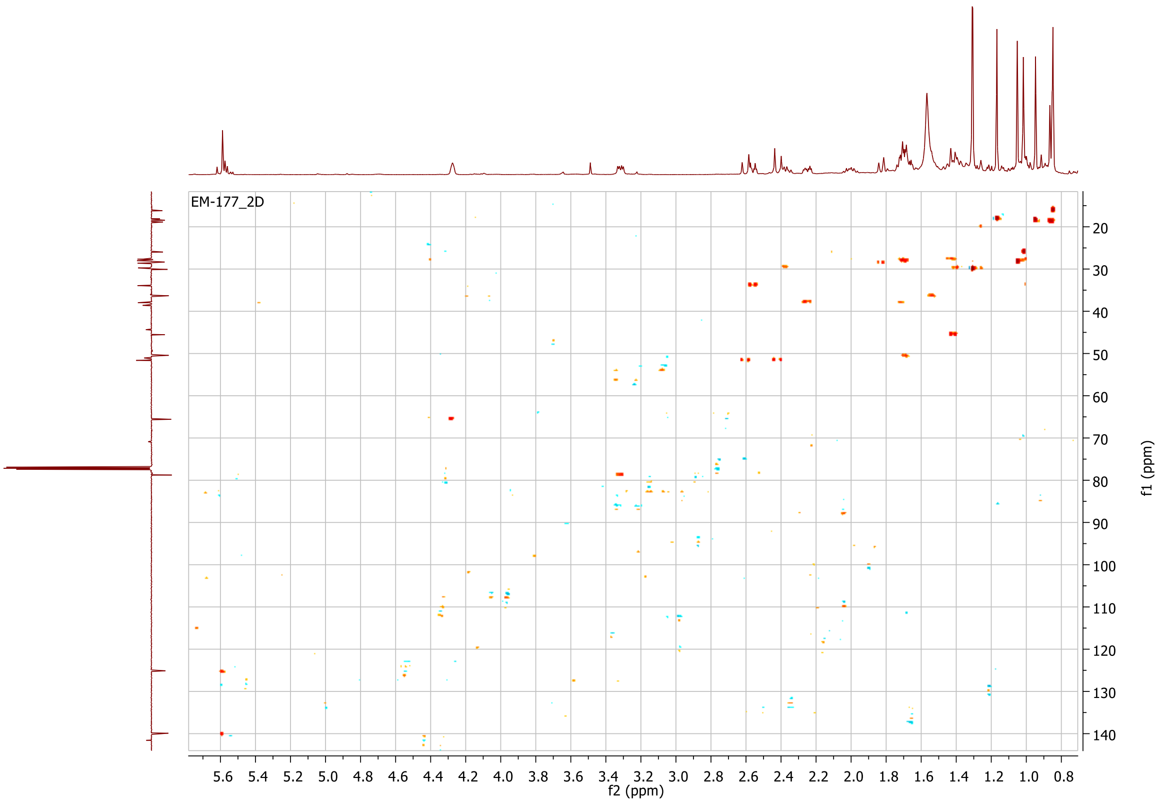

Figure S77. HSQC spectrum of compound **10**

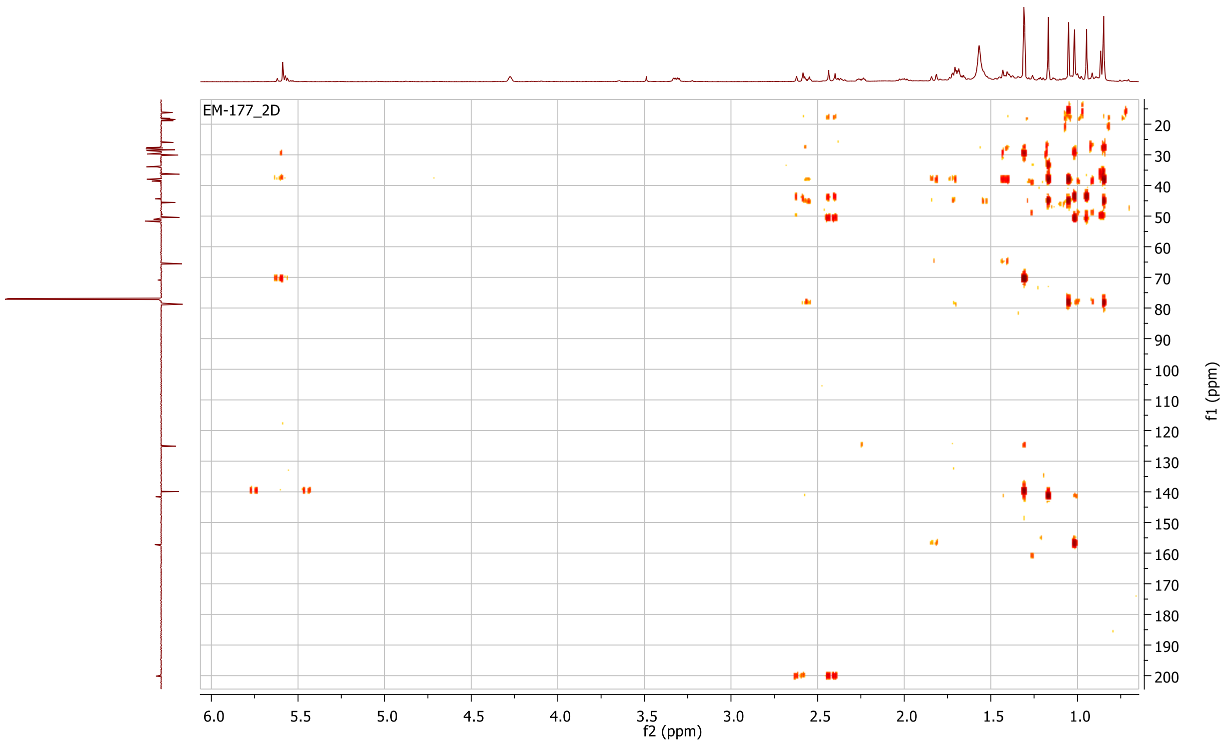

Figure S78. HMBC spectrum of compound **10**

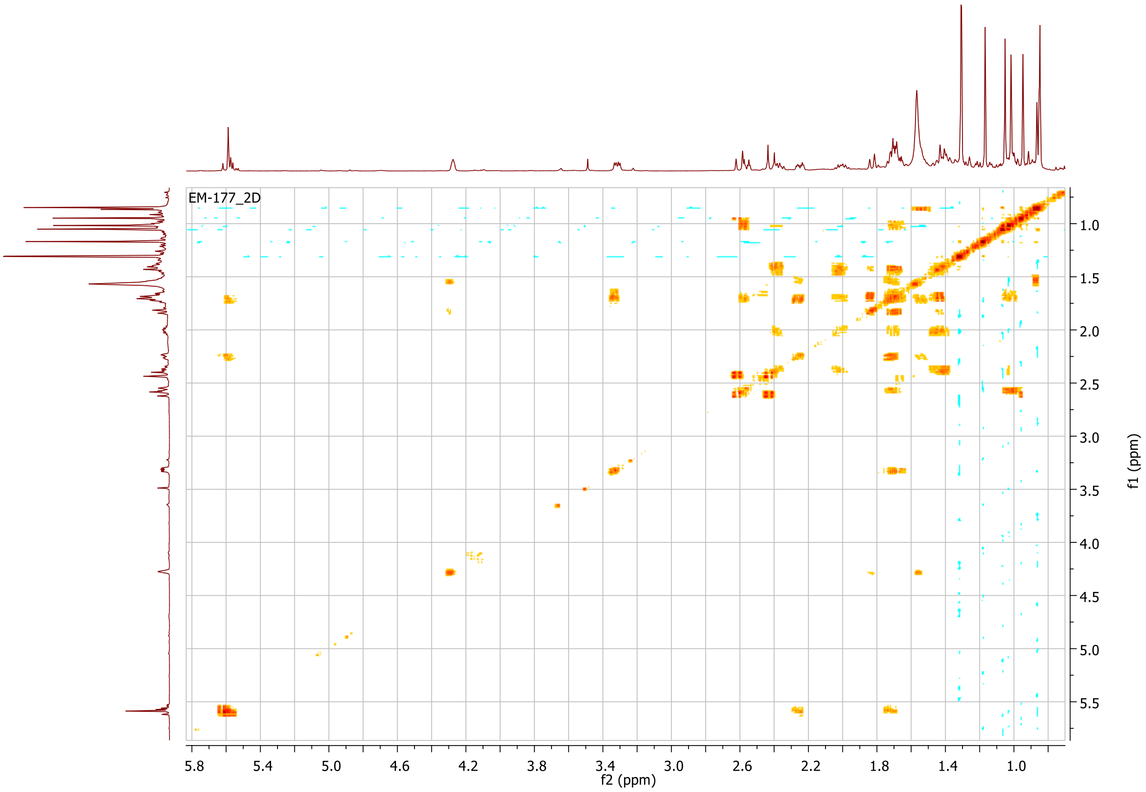

Figure S79. ^{1}H-^{1}H COSY spectrum of compound **10**

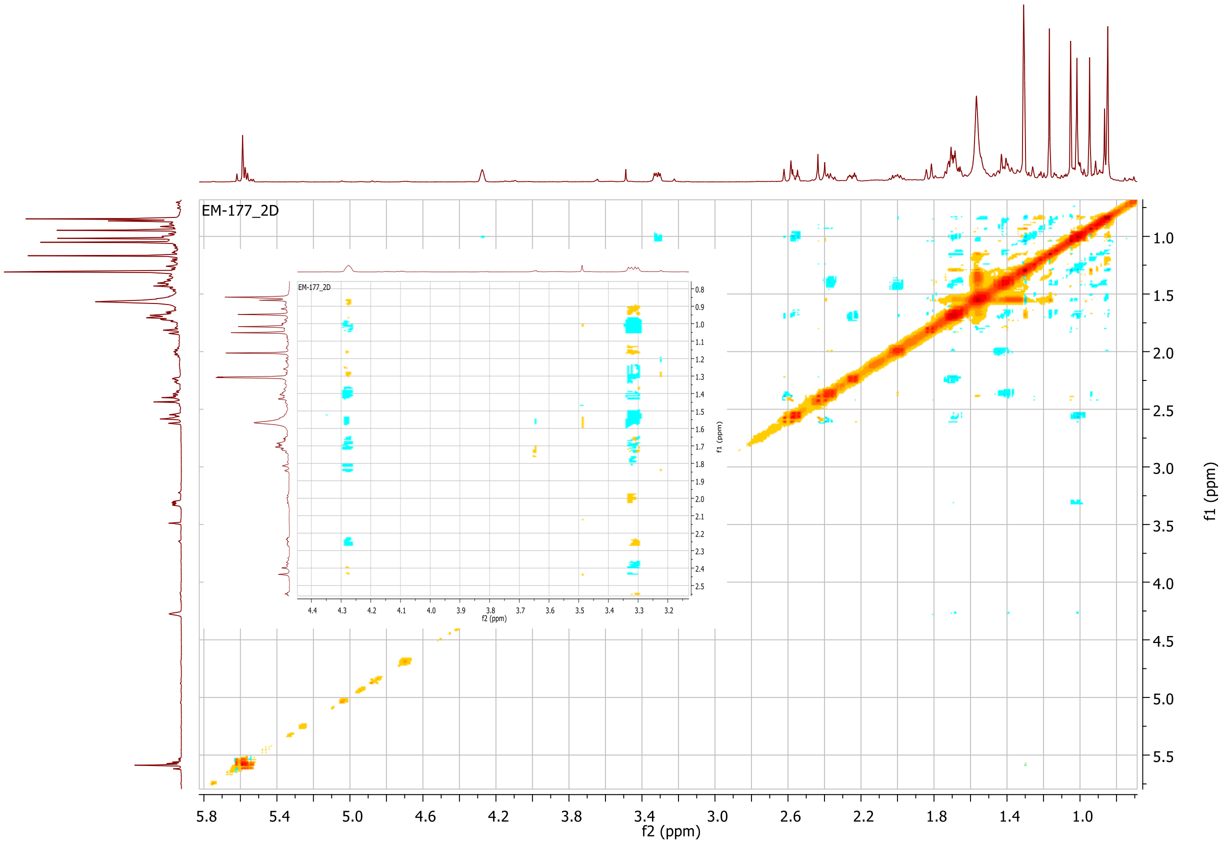

Figure S80. NOESY spectrum of compound **10**

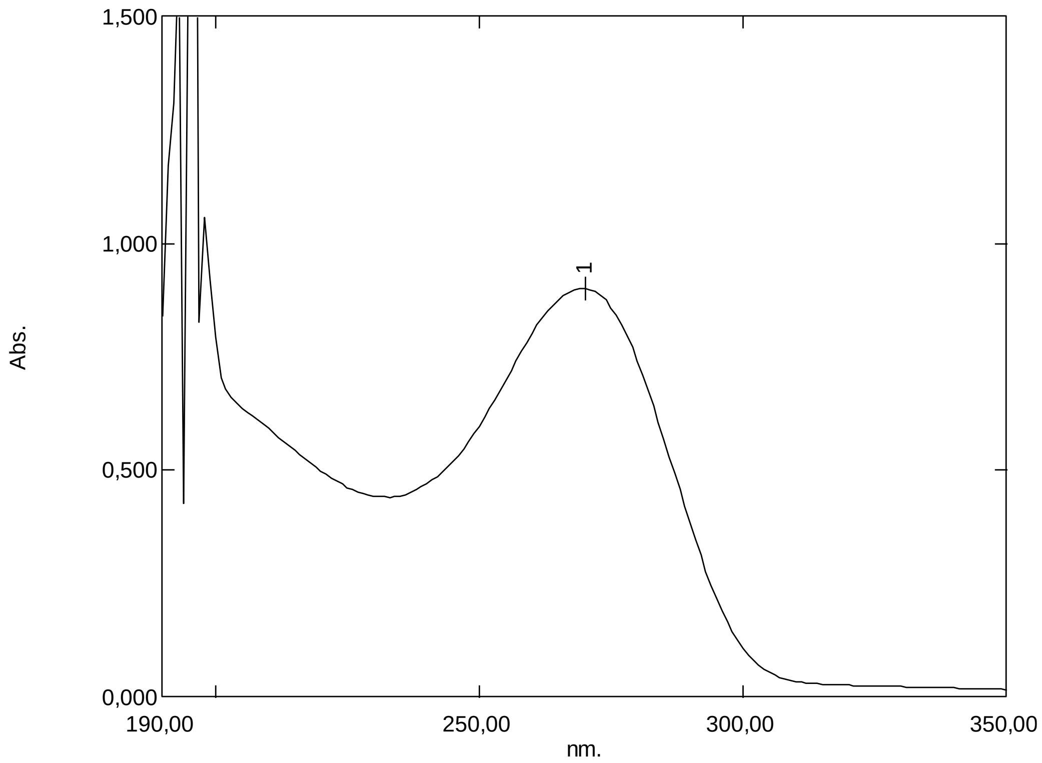

Figure S81. UV spectrum of compound **11**

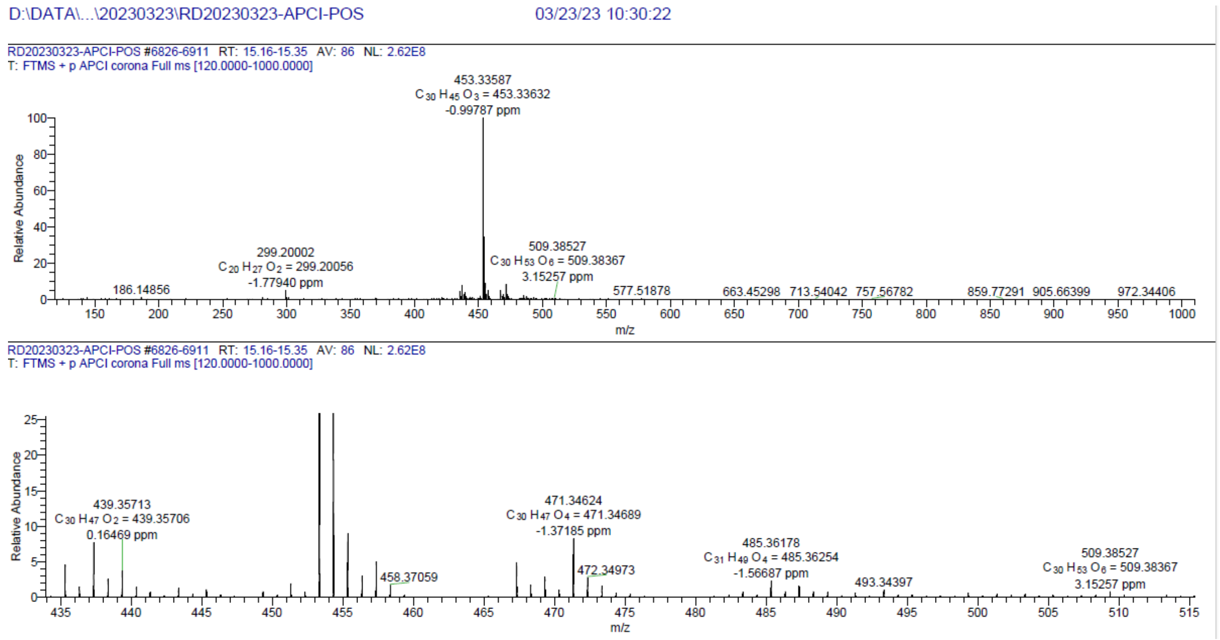

Figure S82. HR-APCI-MS spectrum of compound **11**

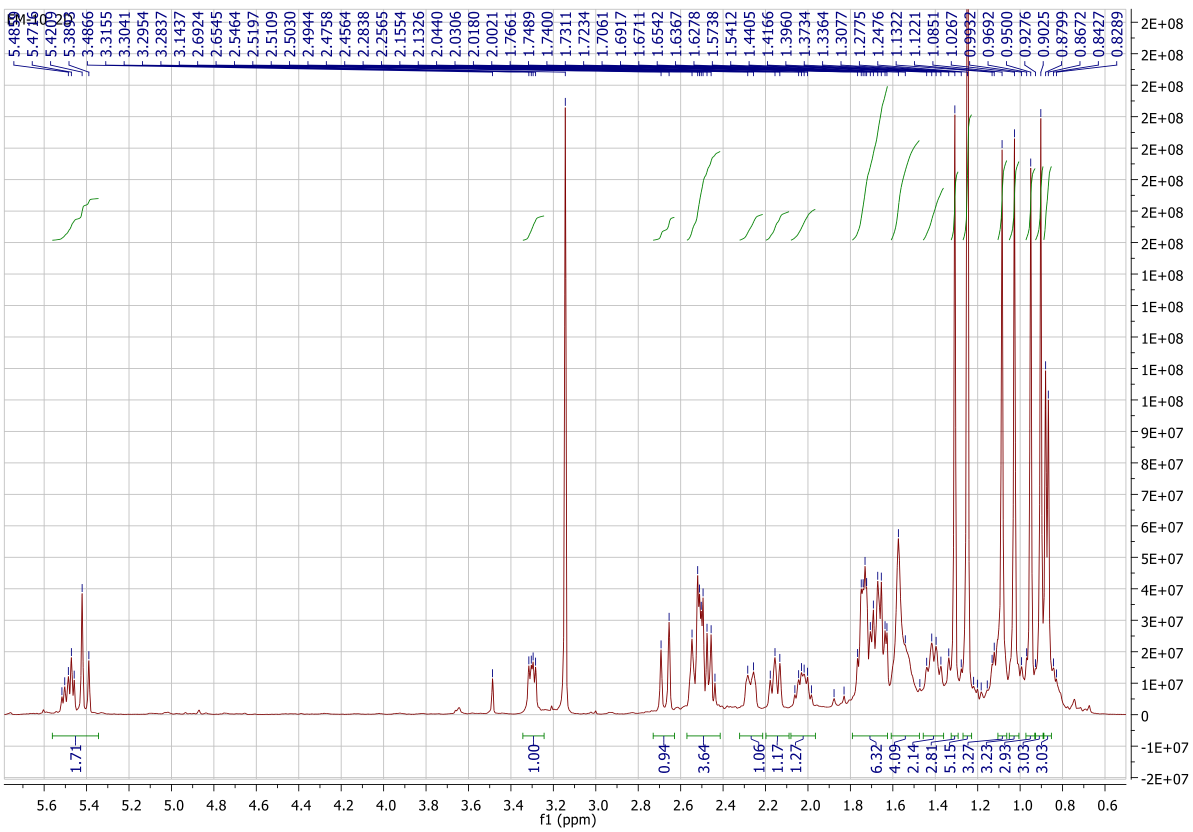

Figure S83. ^{1}H NMR spectrum of compound **11** (500 MHz, $CDCl_3$)

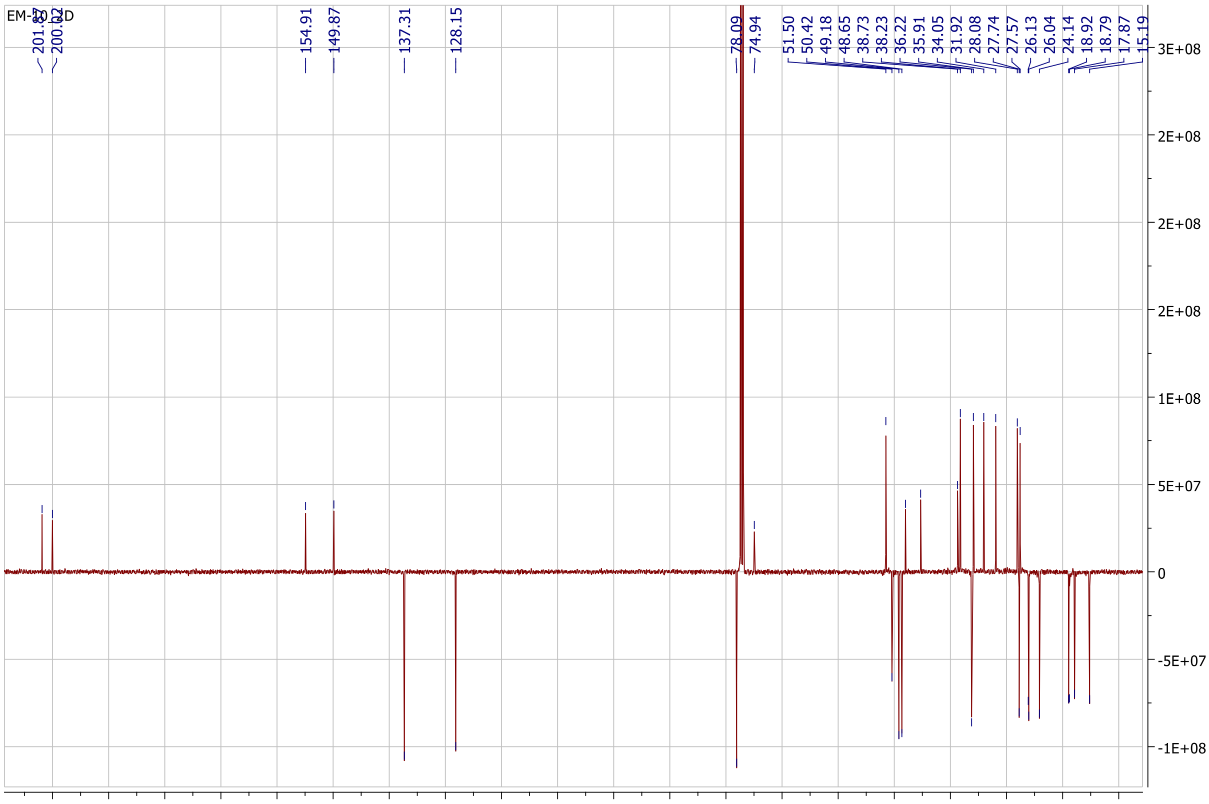

Figure S84. ^{13}C NMR JMOD spectrum of compound **11** (125 MHz, $CDCl_3$)

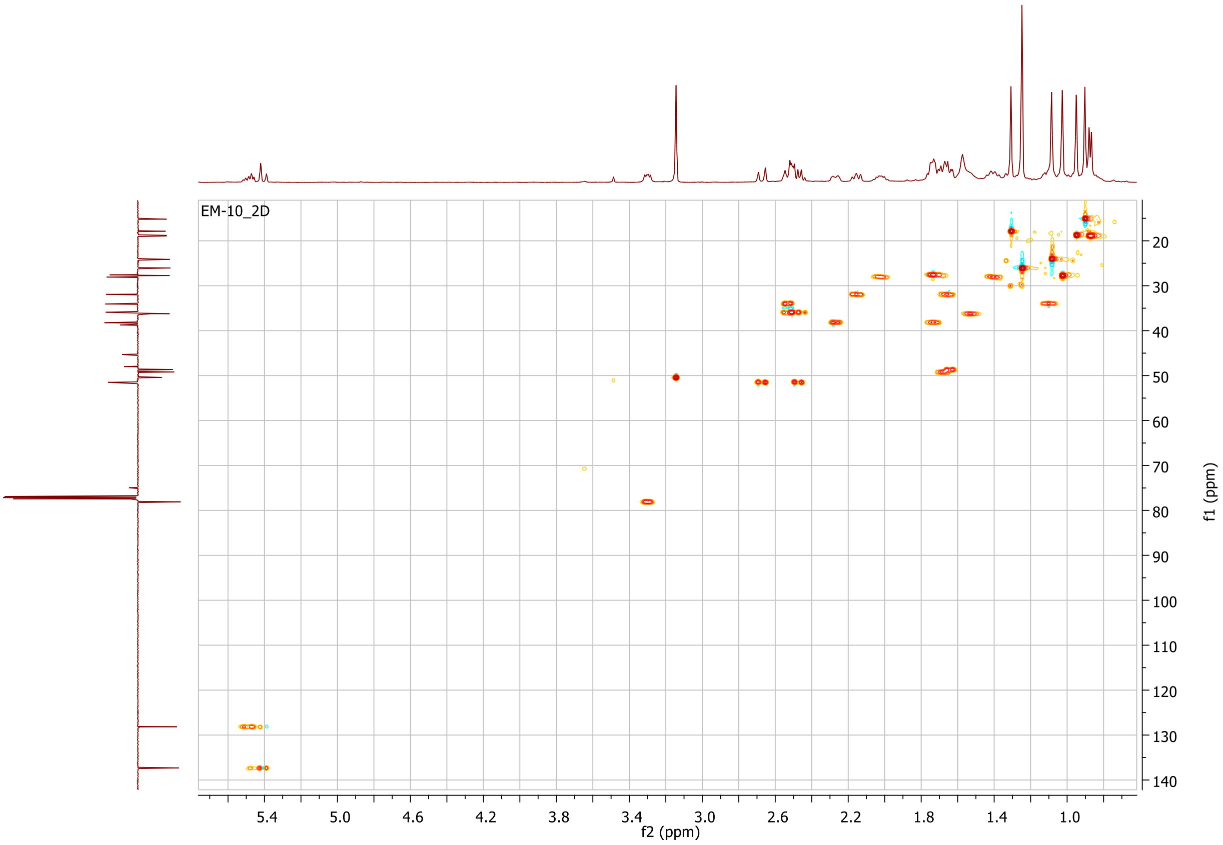

Figure S85. HSQC spectrum of compound **11**

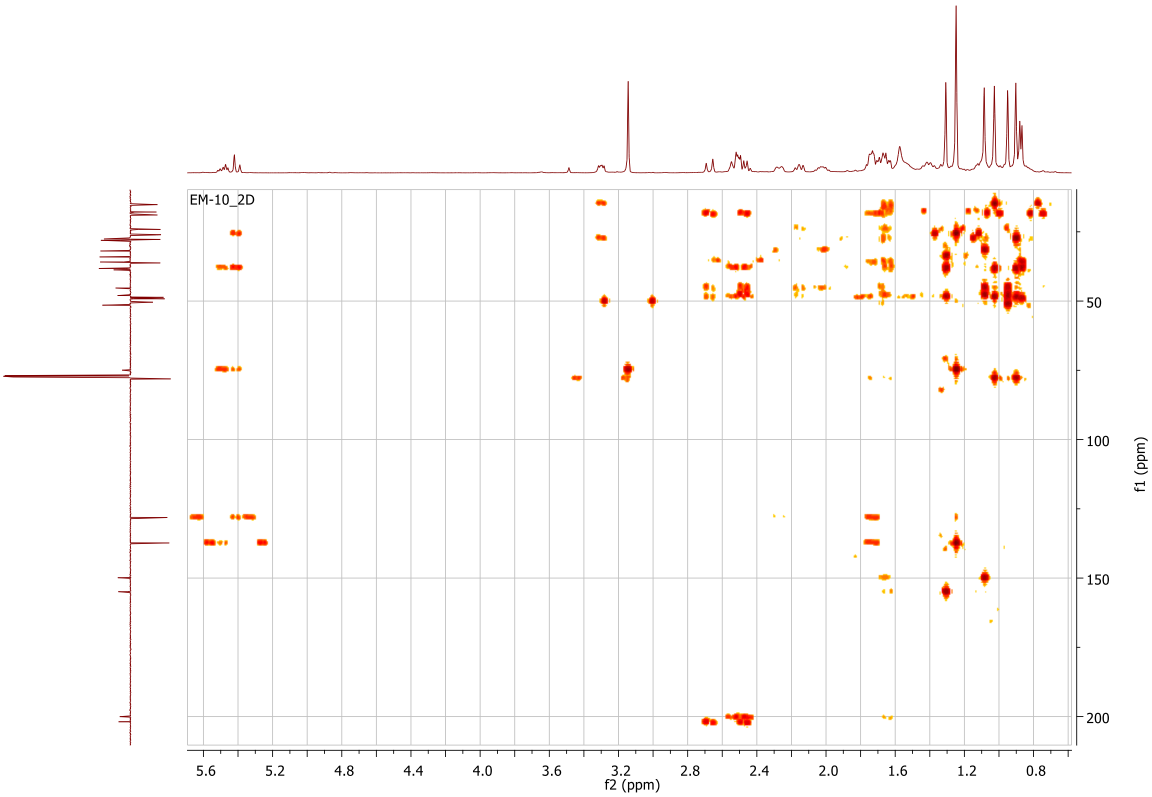

Figure S86. HMBC spectrum of compound **11**

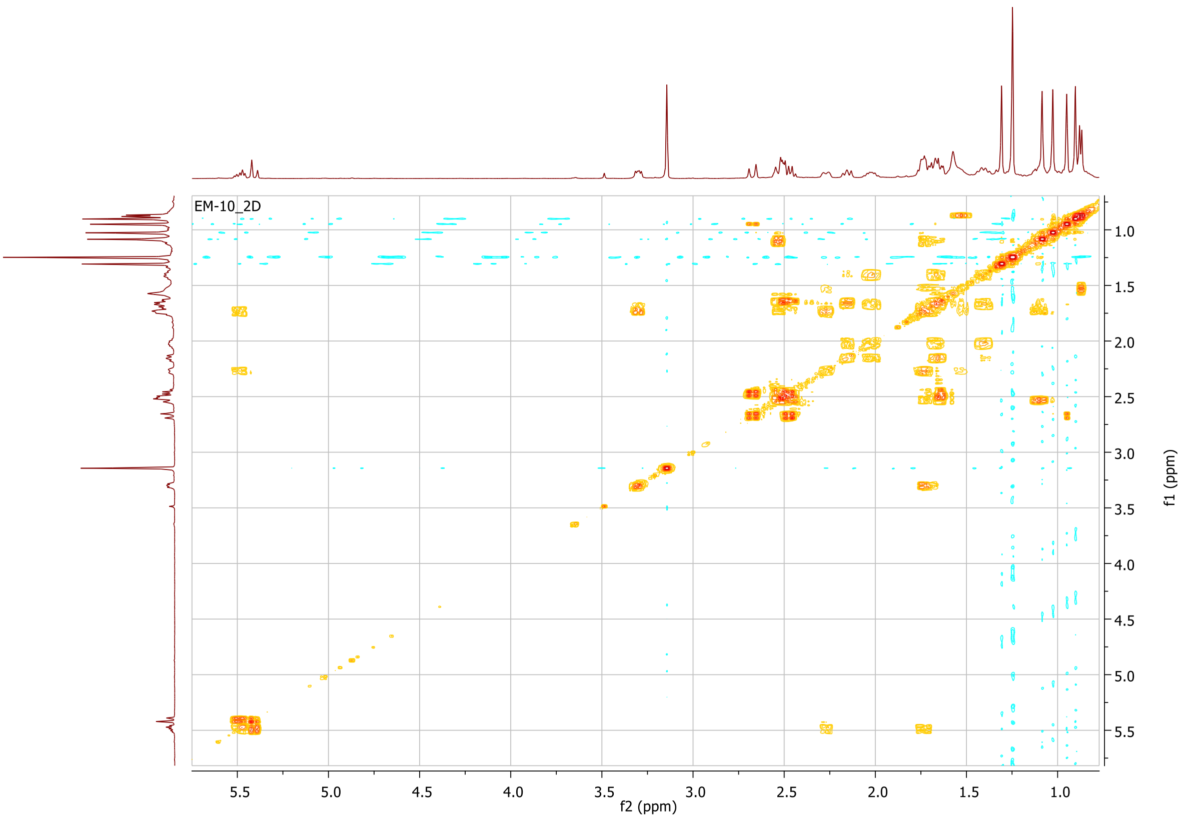

Figure S87. ^{1}H-^{1}H COSY spectrum of compound **11**

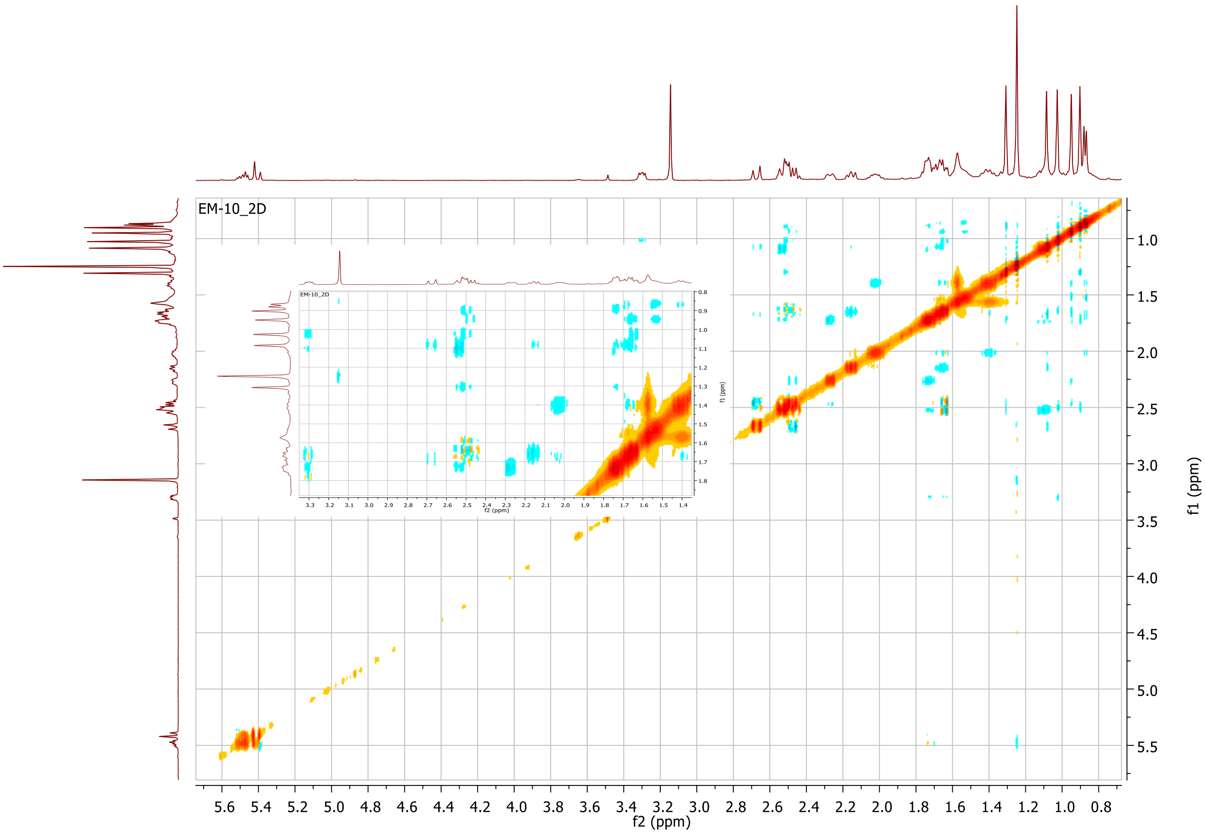

Figure S88. NOESY spectrum of compound **11**

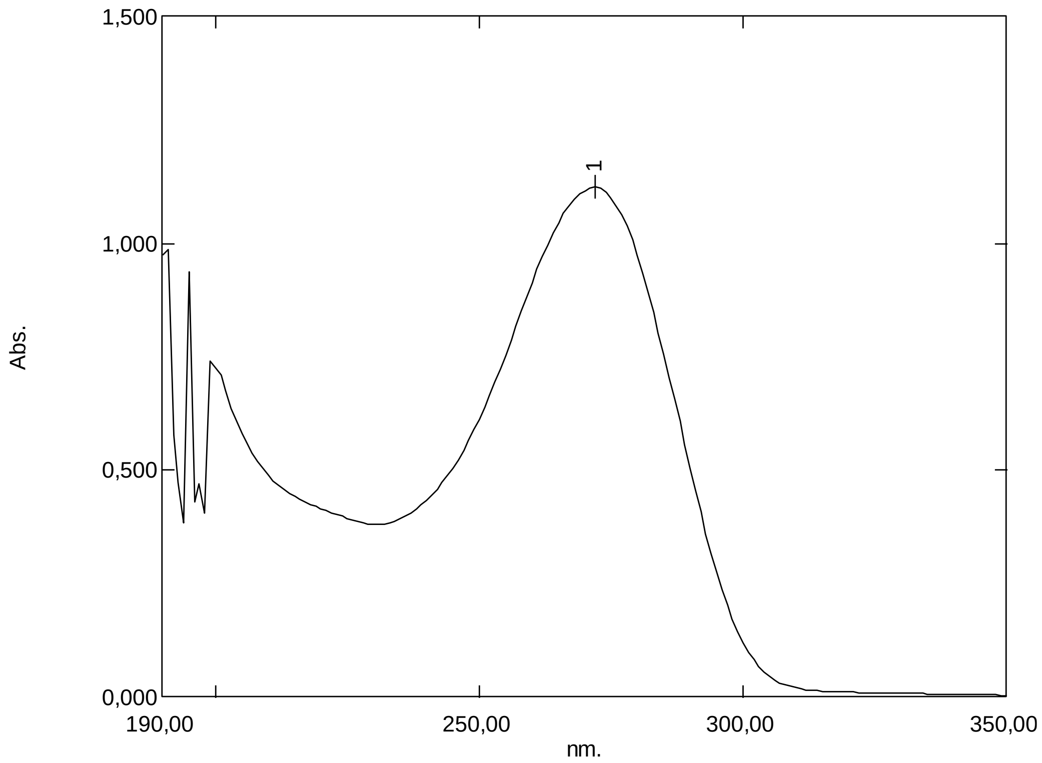

Figure S89. UV spectrum of compound **12**

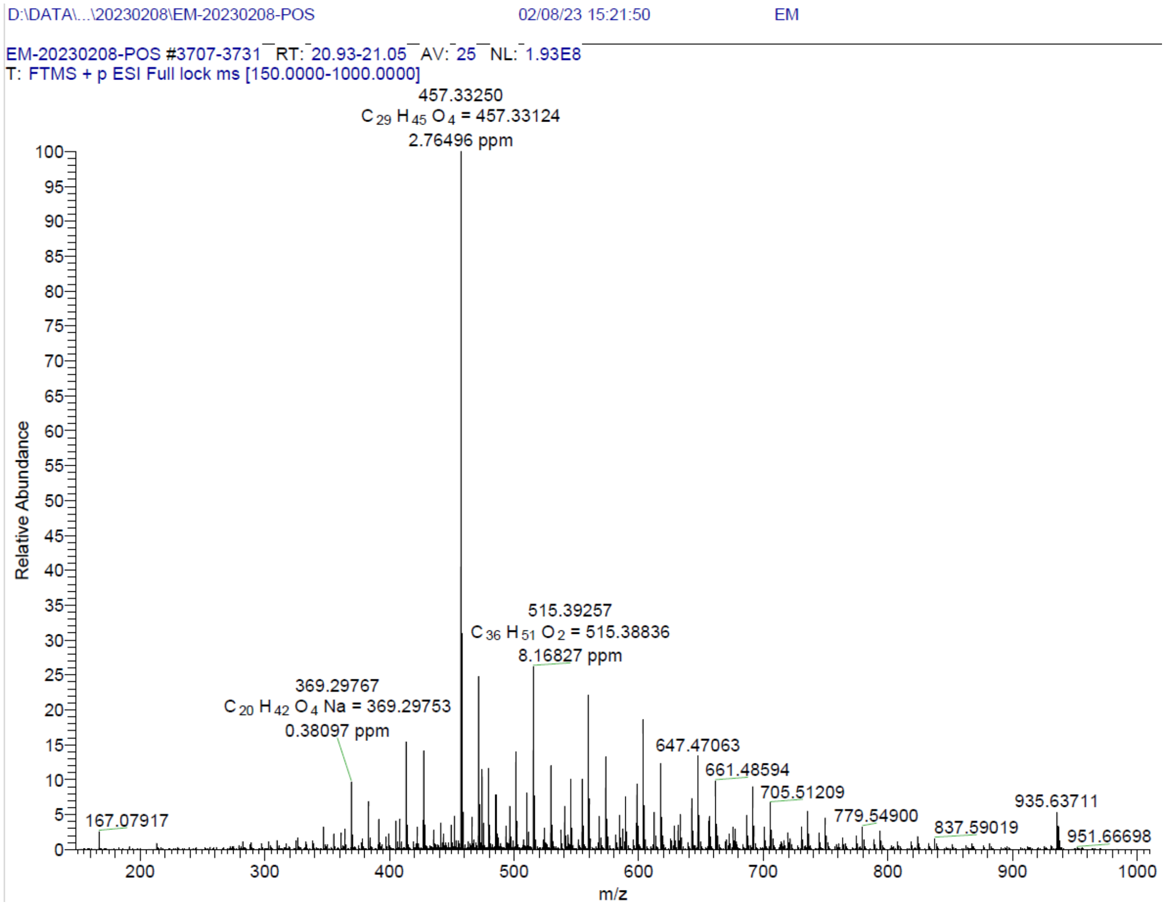

Figure S90. HRESIMS of compound **12**

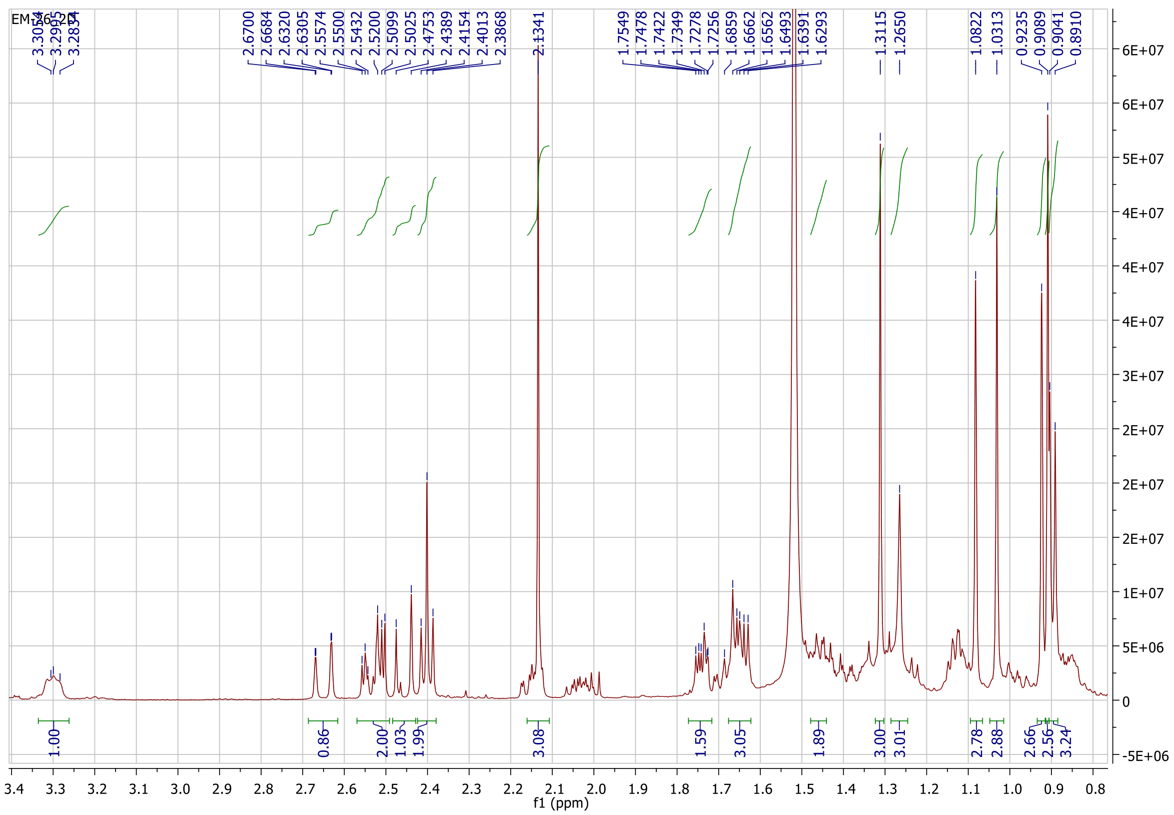

Figure S91. ^{1}H NMR spectrum of compound **12** (500 MHz, $CDCl_3$)

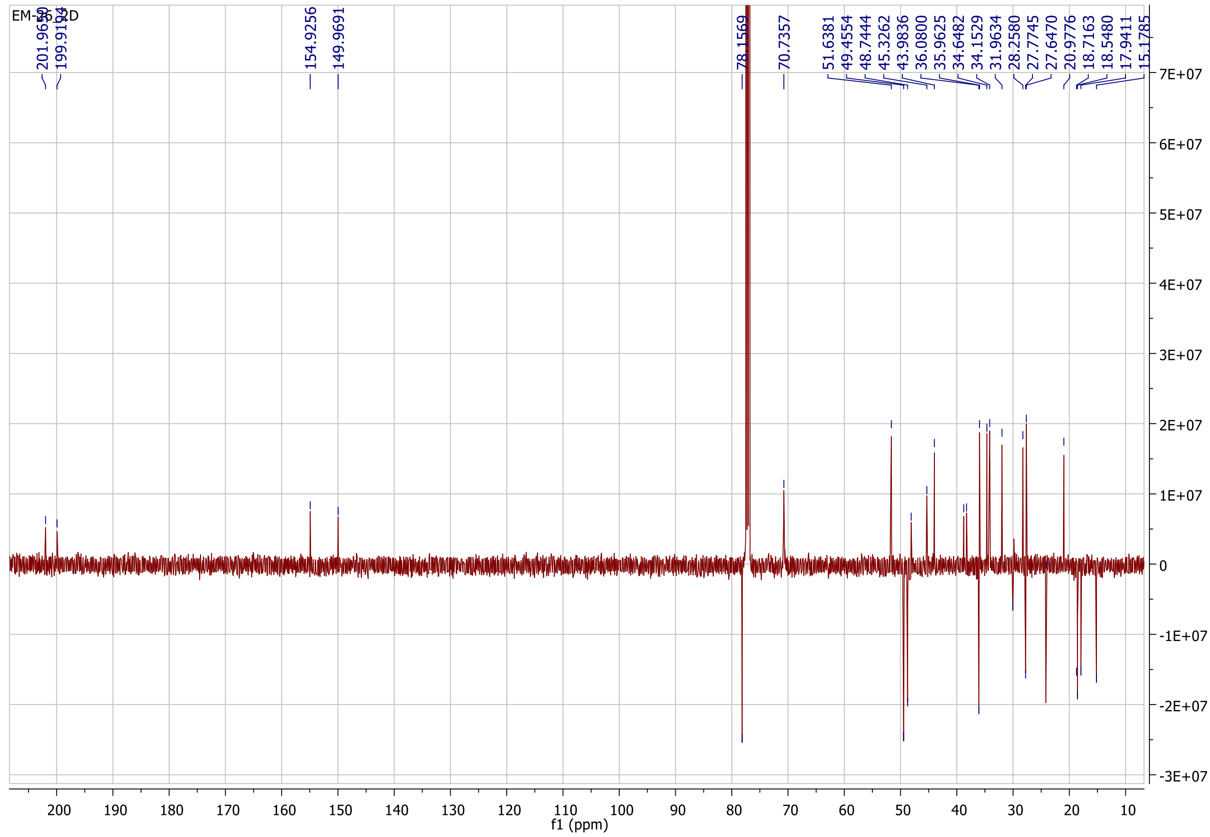

Figure S92. ^{13}C NMR JMOD spectrum of compound **12** (125 MHz, $CDCl_3$)

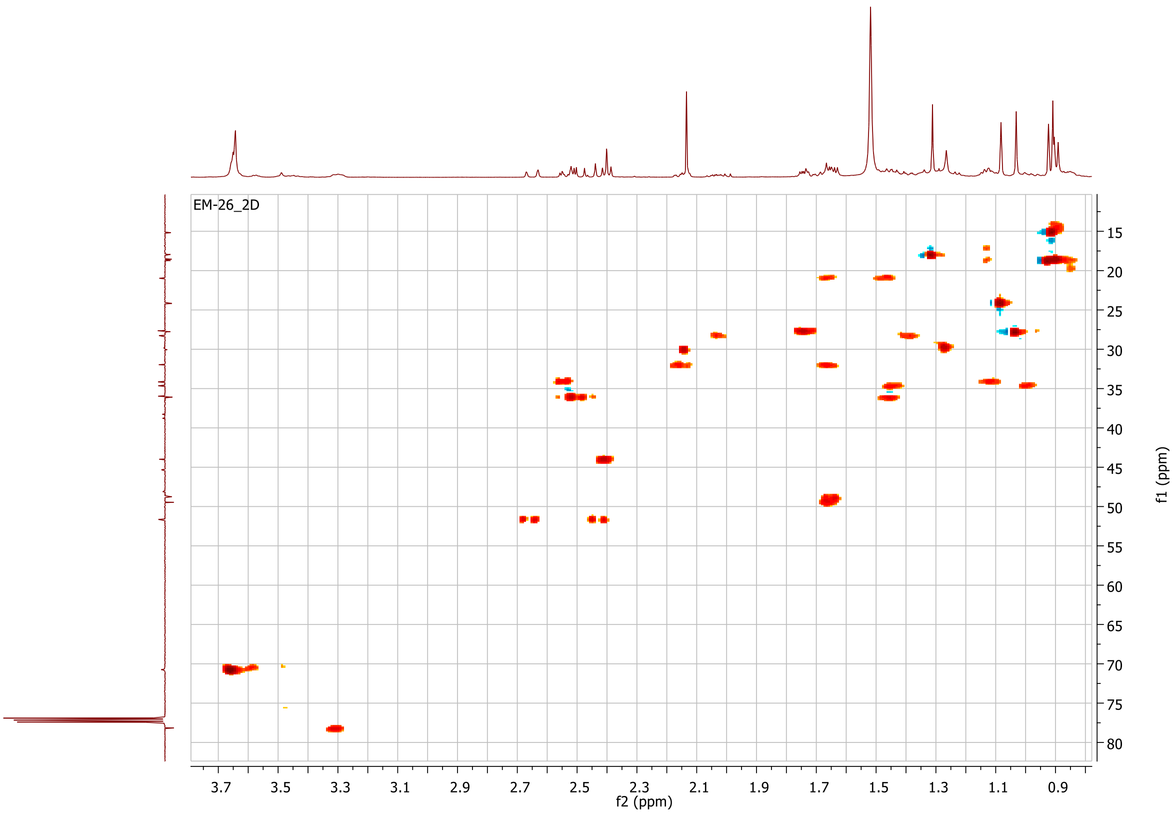

Figure S93. HSQC spectrum of compound **12**

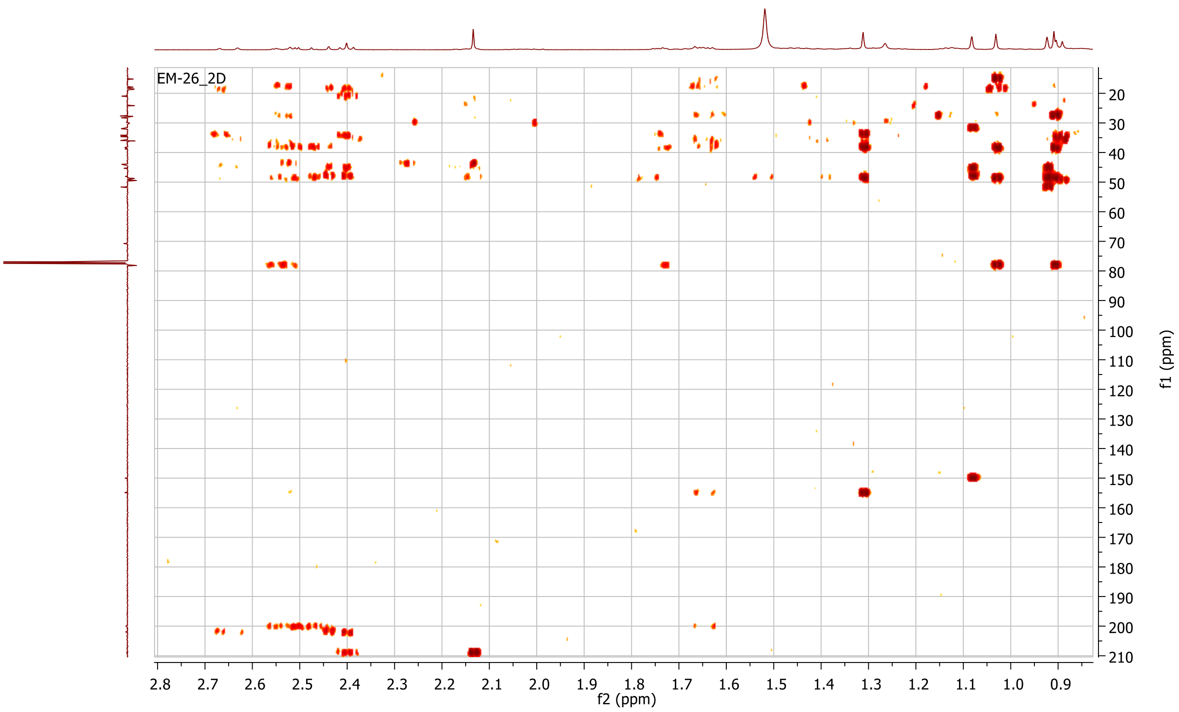

Figure S94. HMBC spectrum of compound **12**

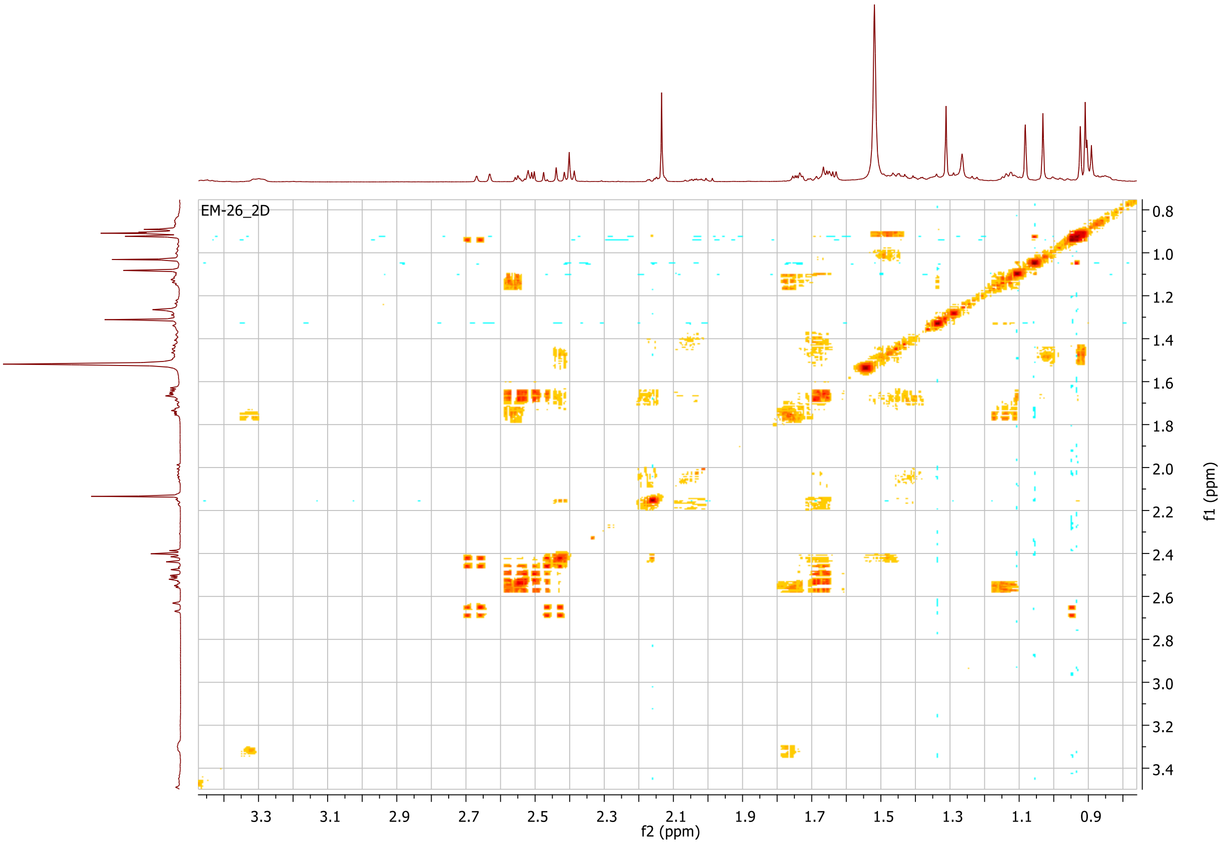

Figure S95. ^{1}H-^{1}H COSY spectrum of compound **12**

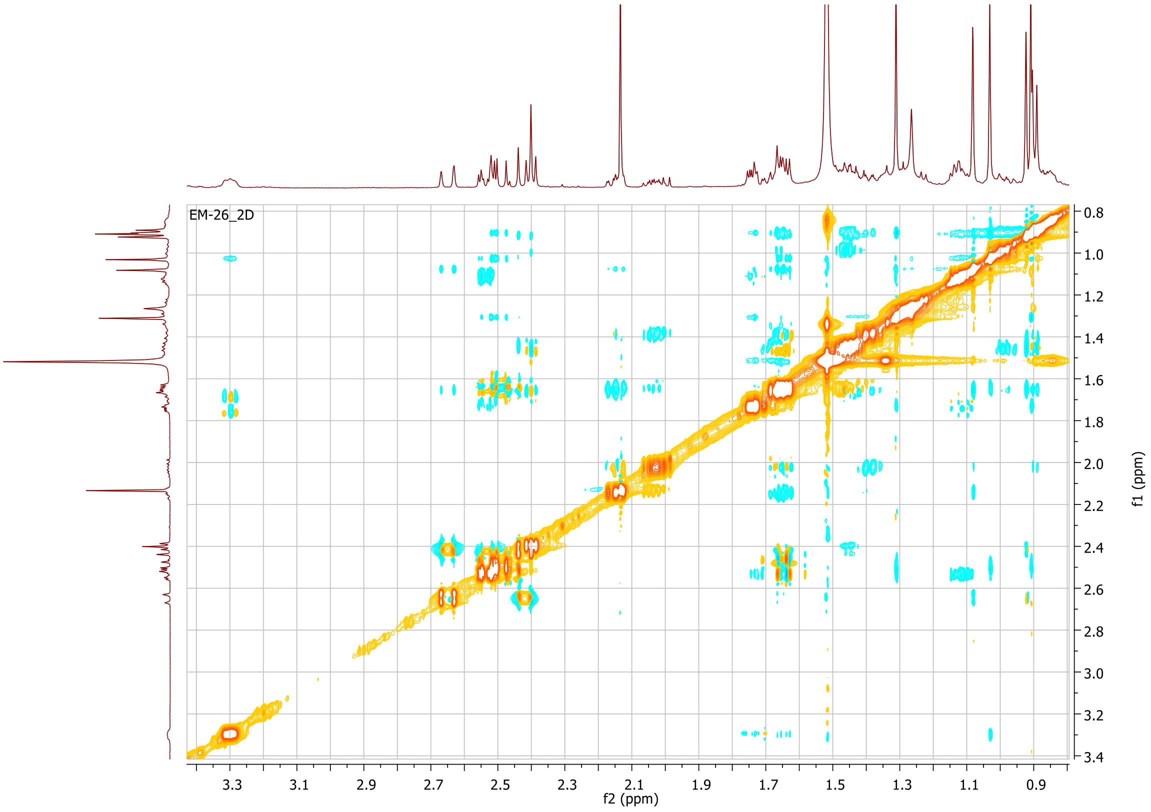

Figure S96. NOESY spectrum of compound **12**

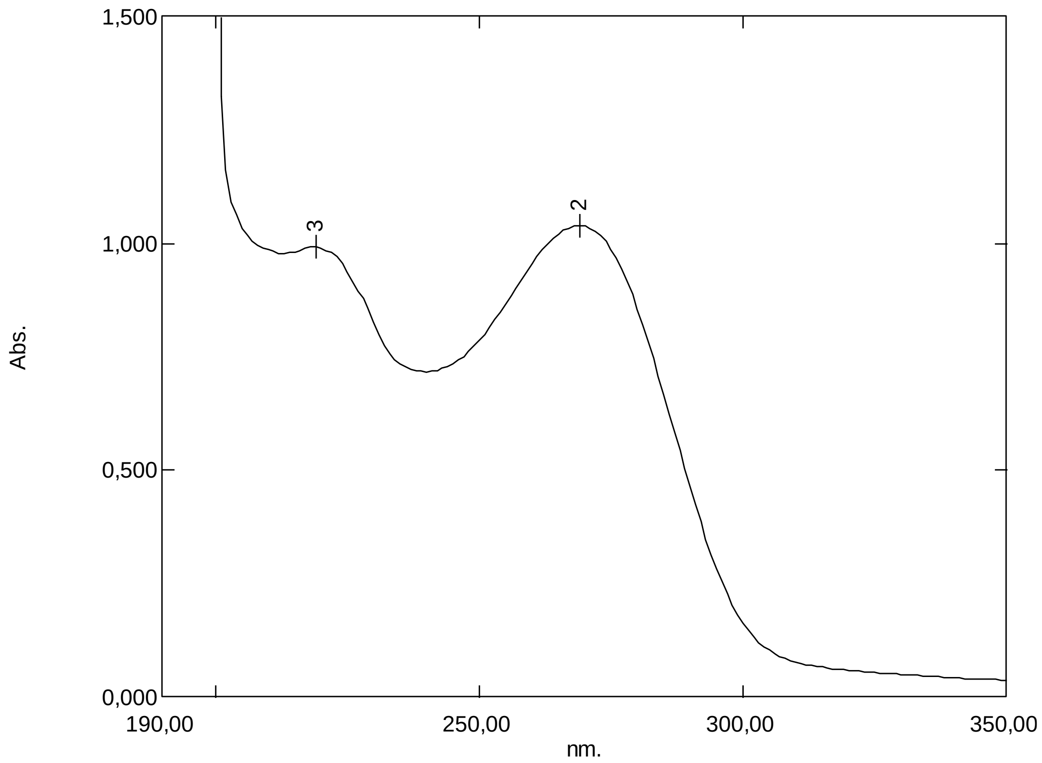

Figure S97. UV spectrum of compound **13**

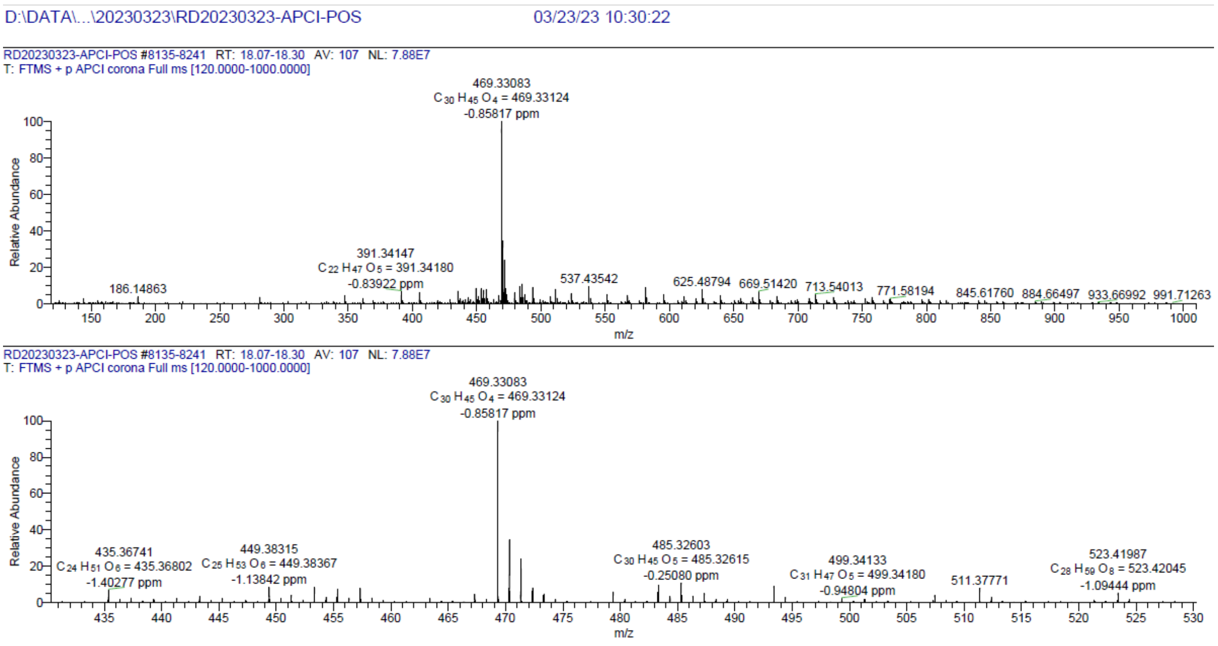

Figure S98. HRESIMS spectrum of compound **13**

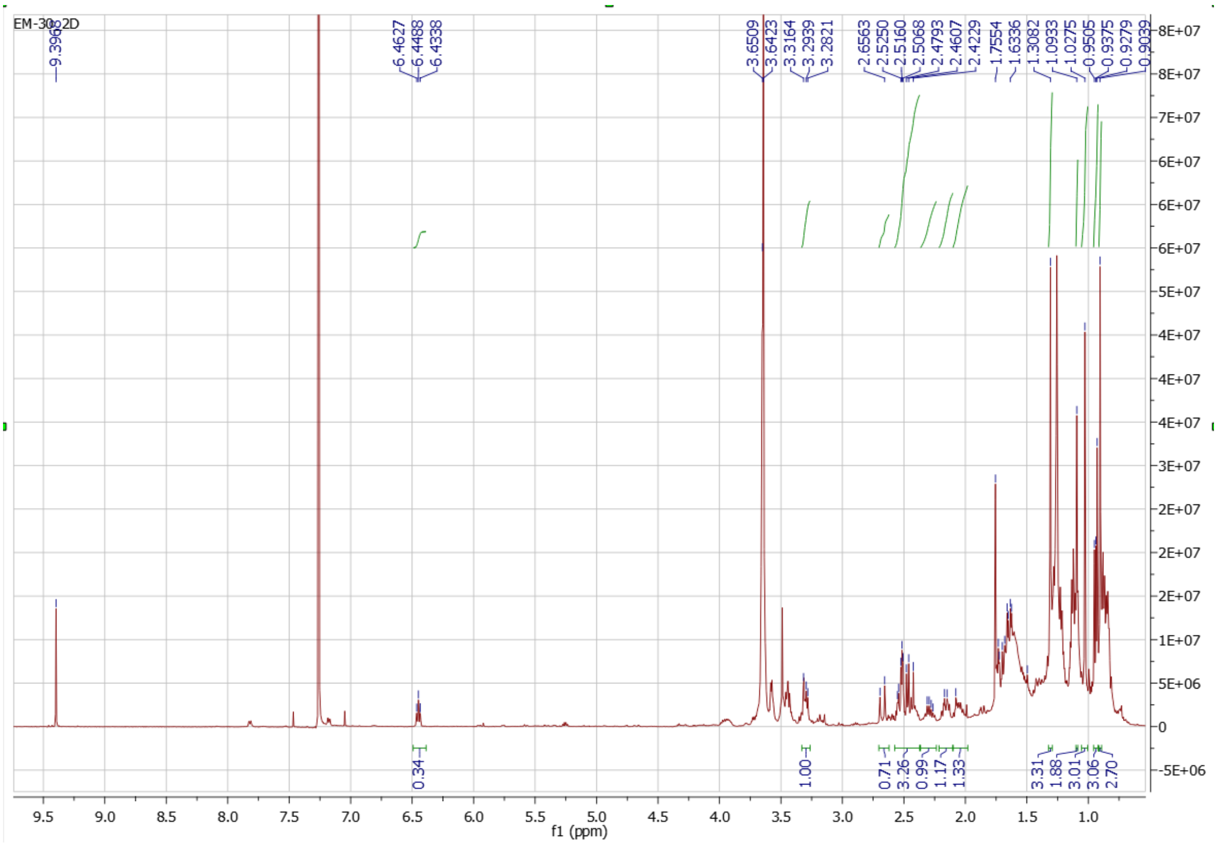

Figure S99. ^{1}H NMR spectrum of compound **13** (500 MHz, $CDCl_3$)

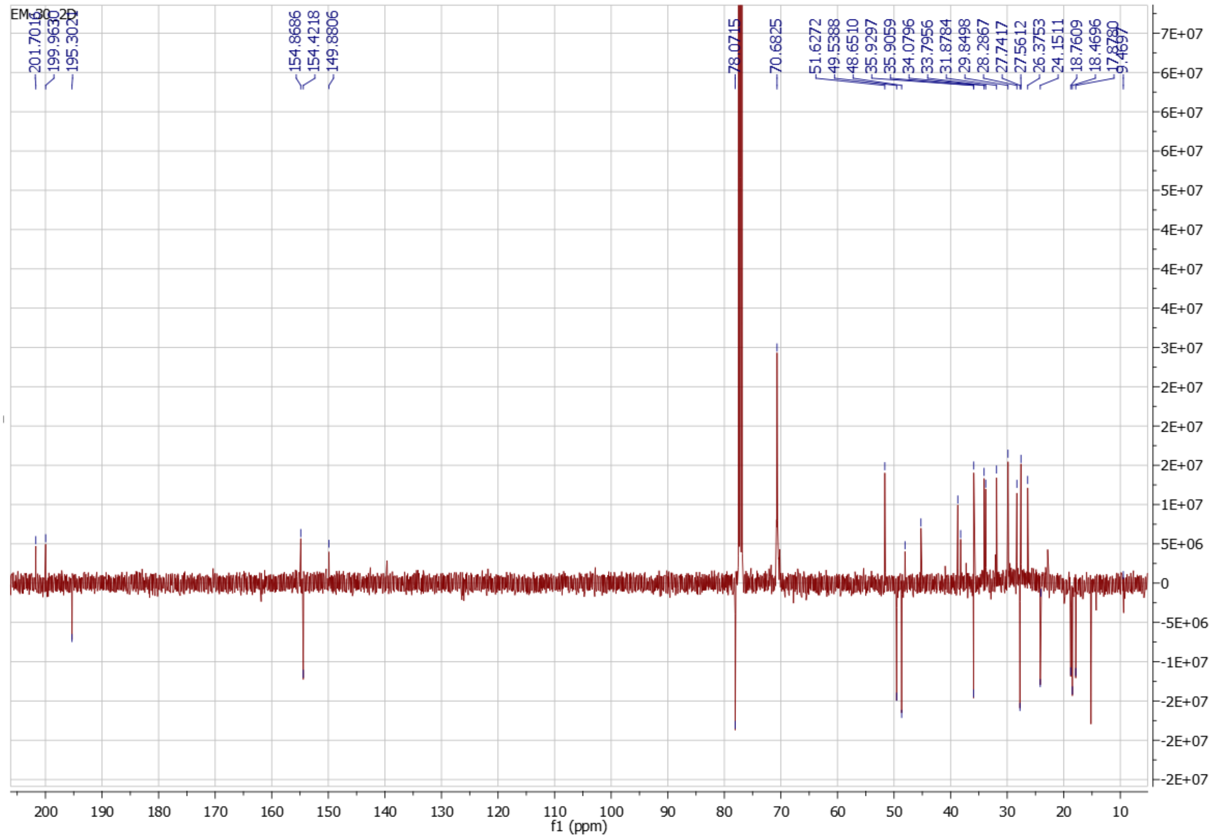

Figure S100. ^{13}C NMR JMOD spectrum of compound **13** (125 MHz, $CDCl_3$)

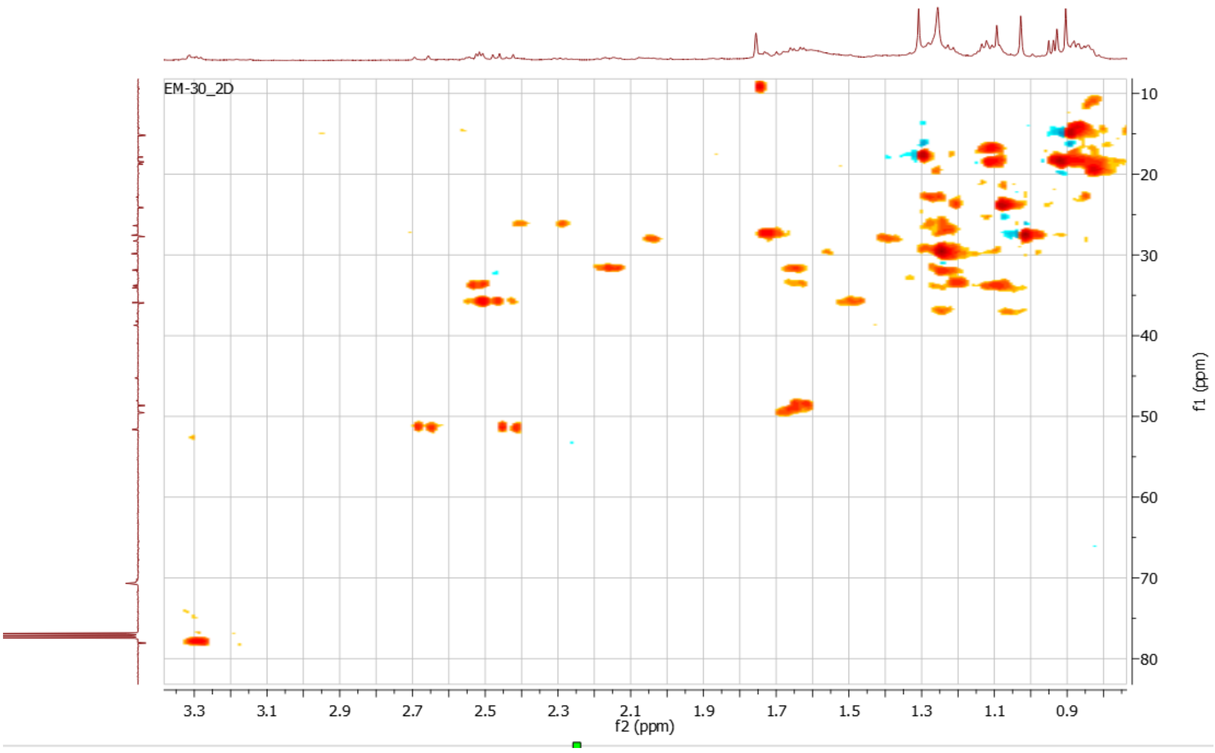

Figure S101. HSQC spectrum of compound **13**

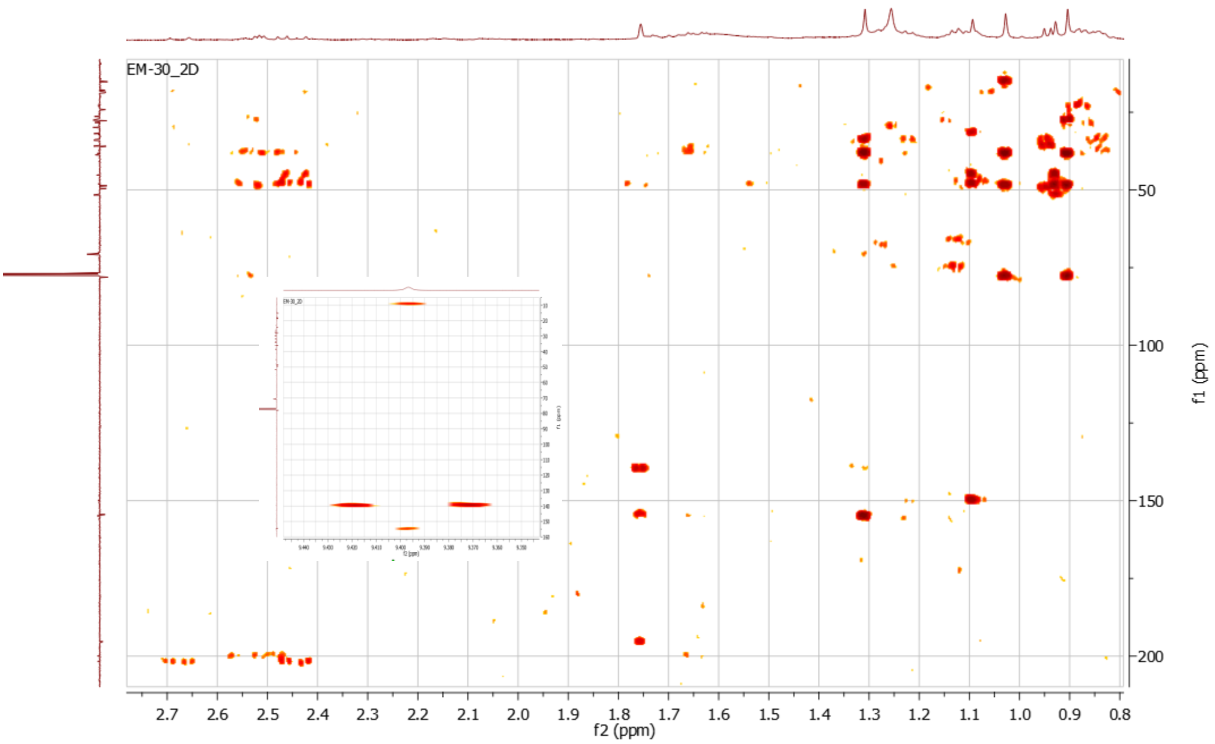

Figure S102. HMBC spectrum of compound **13**

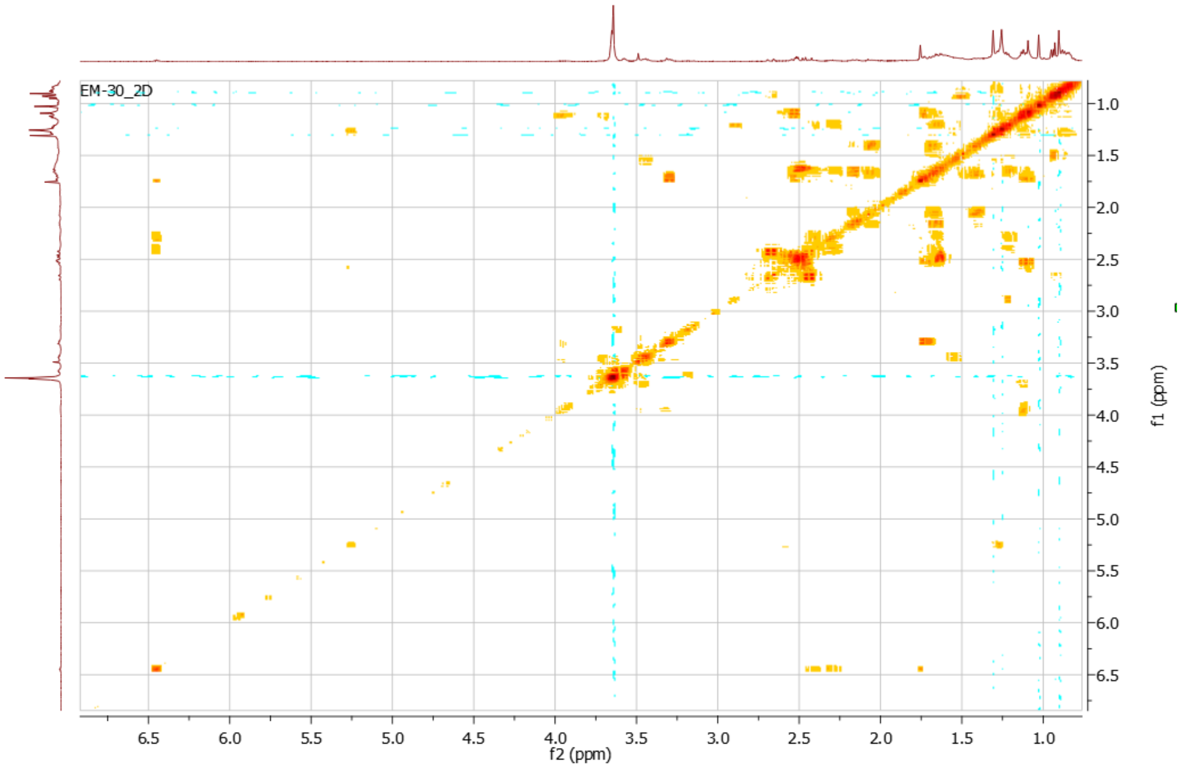

Figure S103. ^{1}H-^{1}H COSY spectrum of compound **13**

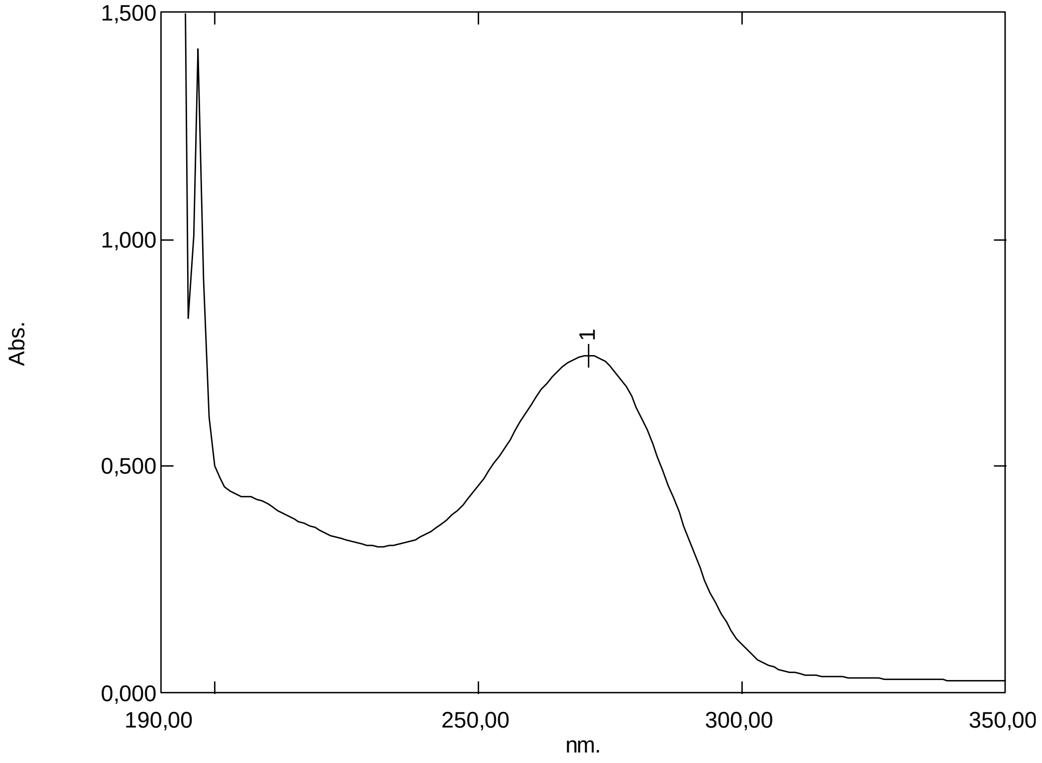

Figure S104. UV spectrum of compound **14 +15**

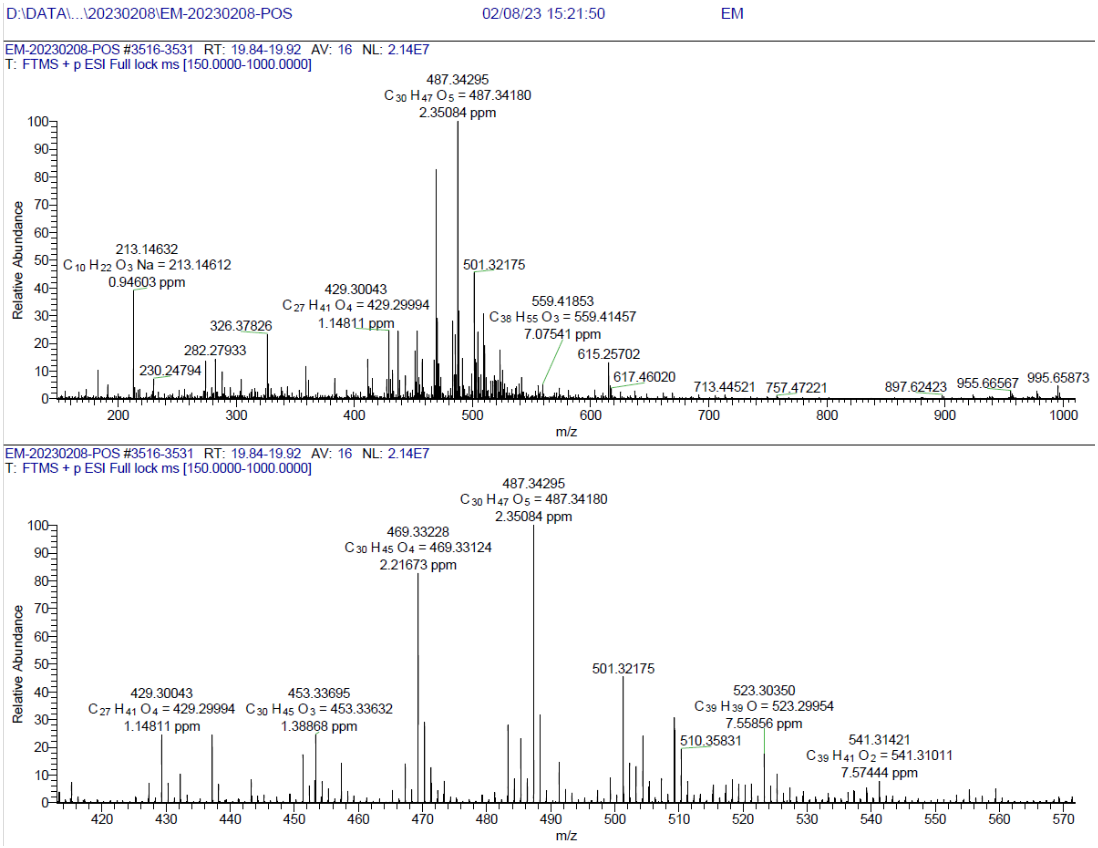

Figure S105. HRESIMS spectrum of compound **14** + **15**

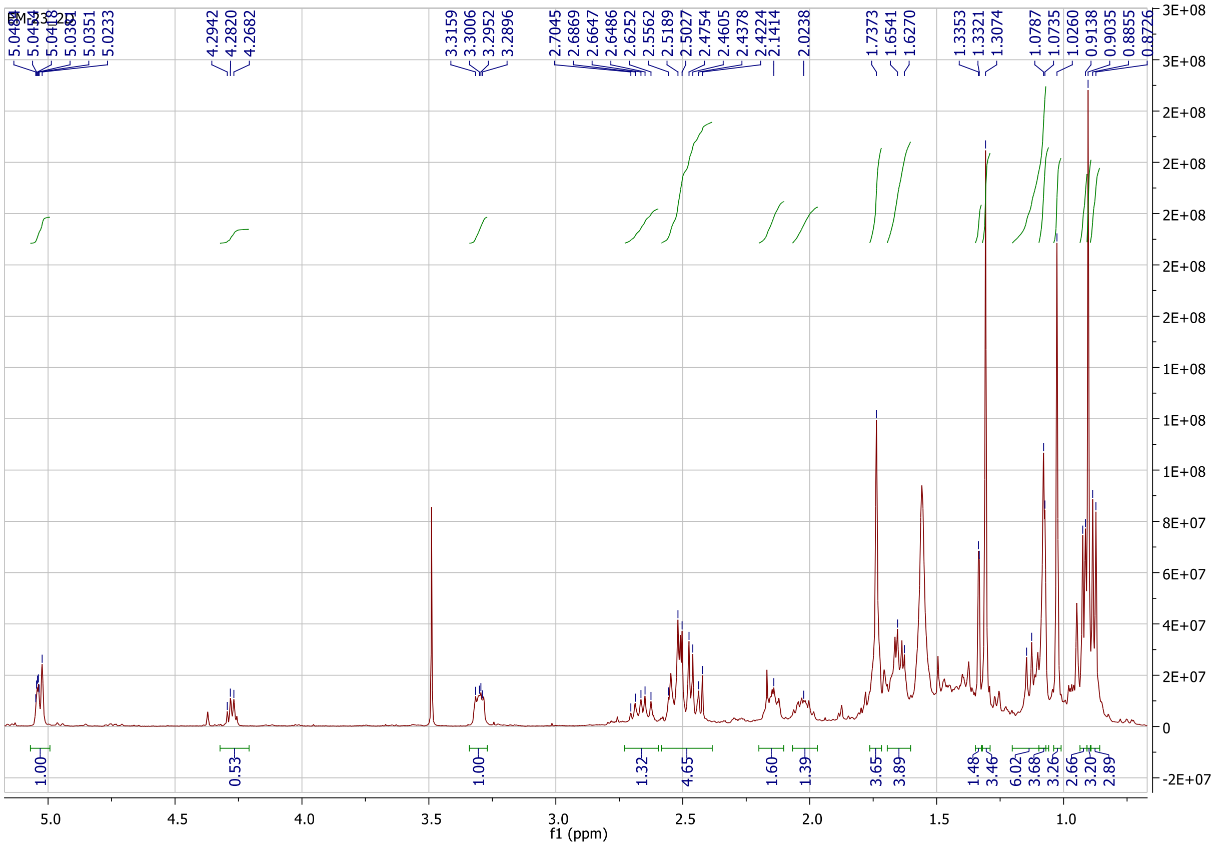

Figure S106. ^{1}H NMR spectrum of compound **14** + **15** (500 MHz, $CDCl_3$)

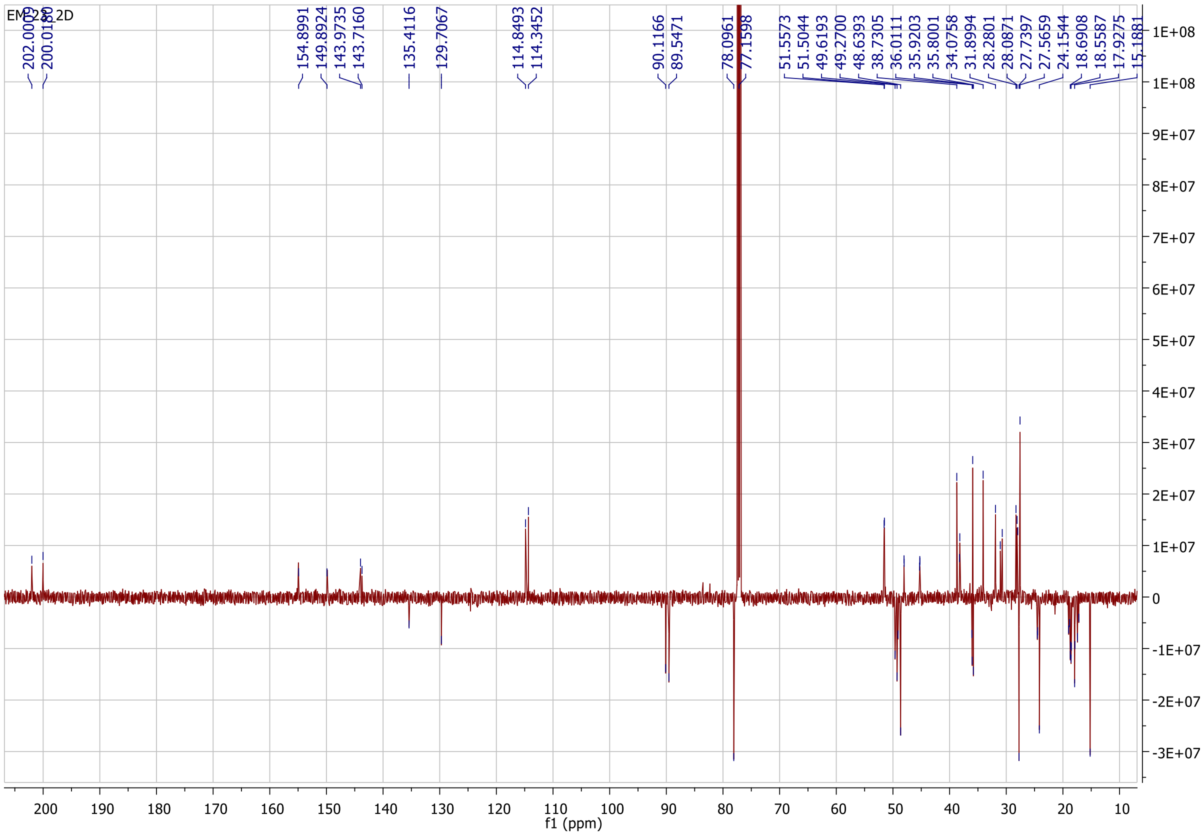

Figure S107. ^{13}C NMR JMOD spectrum of compound **14** + **15** (125 MHz, $CDCl_3$)

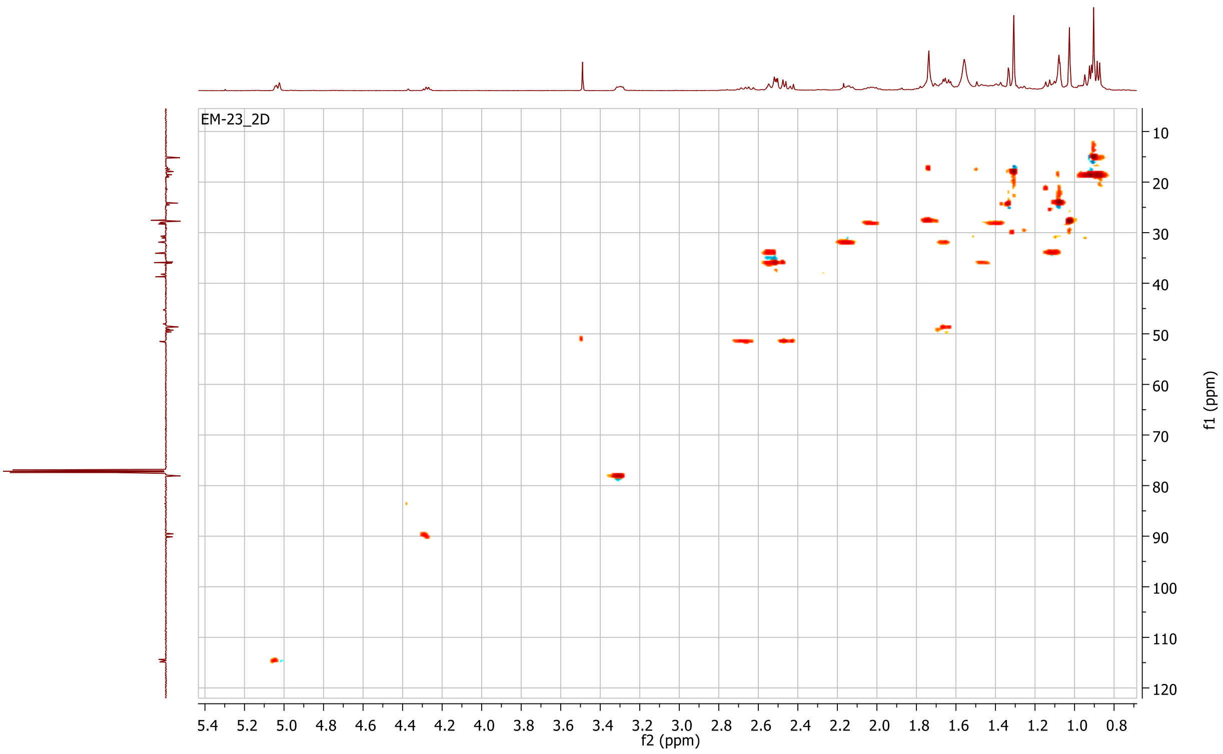

Figure S108. HSQC spectrum of compound **14** + **15**

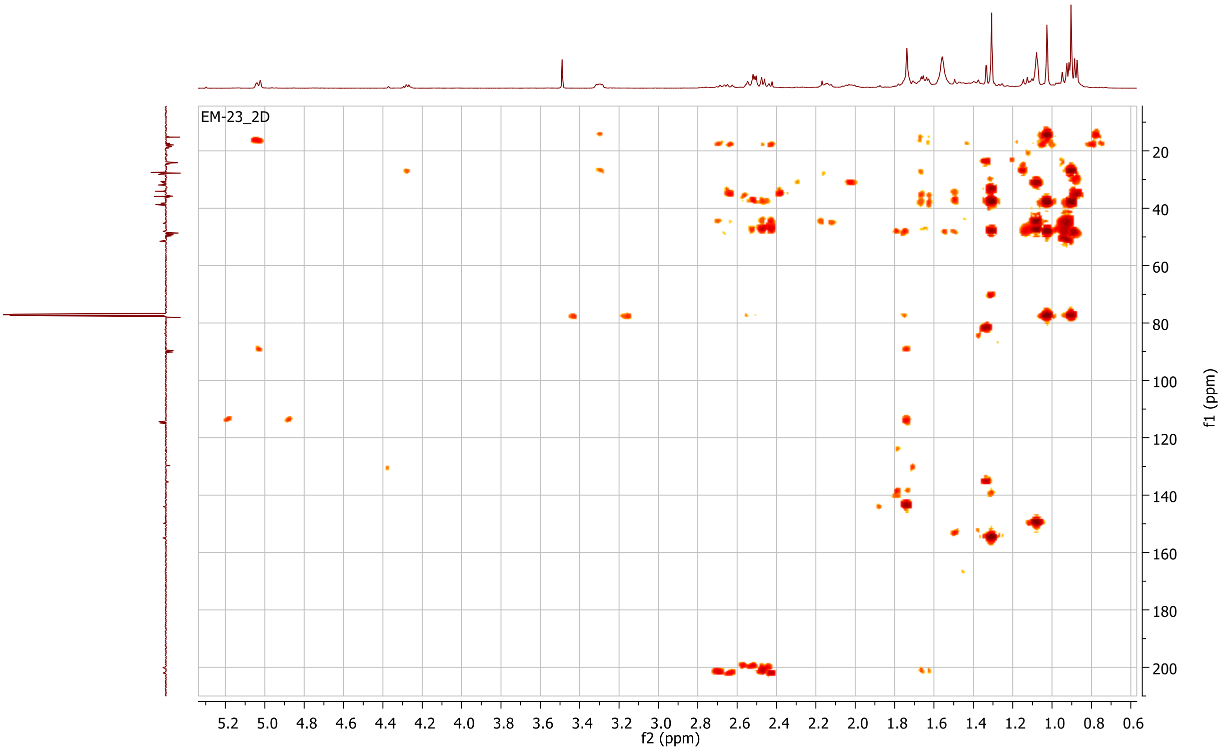

Figure S109. HMBC spectrum of compound **14** + **15**

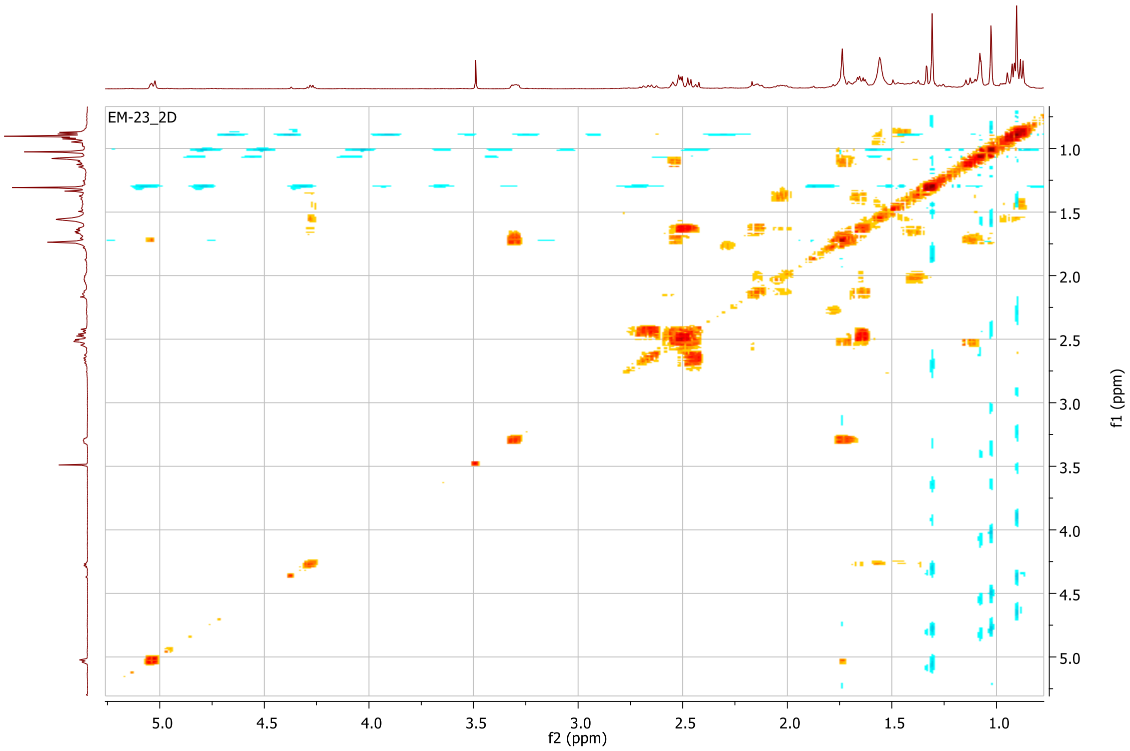

Figure S110. ^{1}H-^{1}H COSY spectrum of compound **14** + **15**

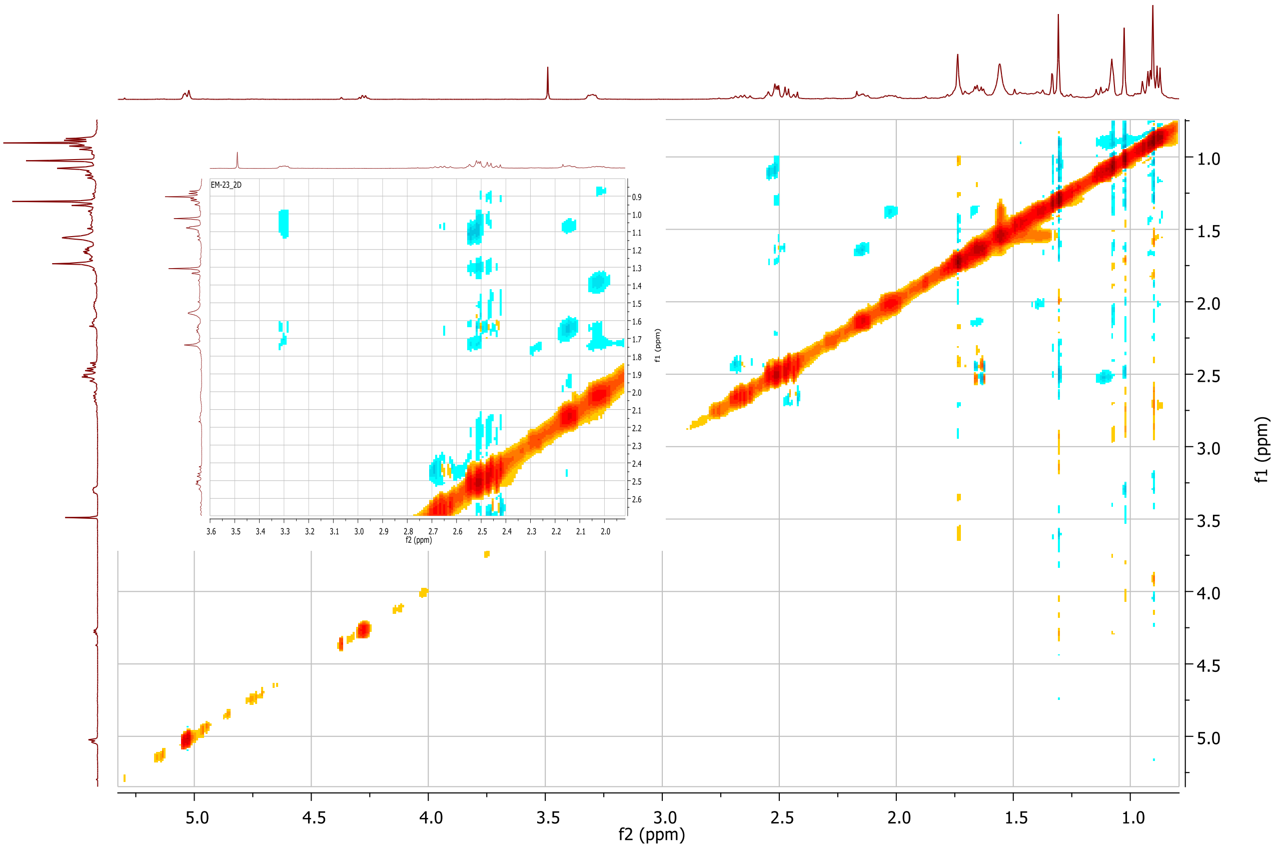

Figure S111. NOESY spectrum of compound **14** + **15**

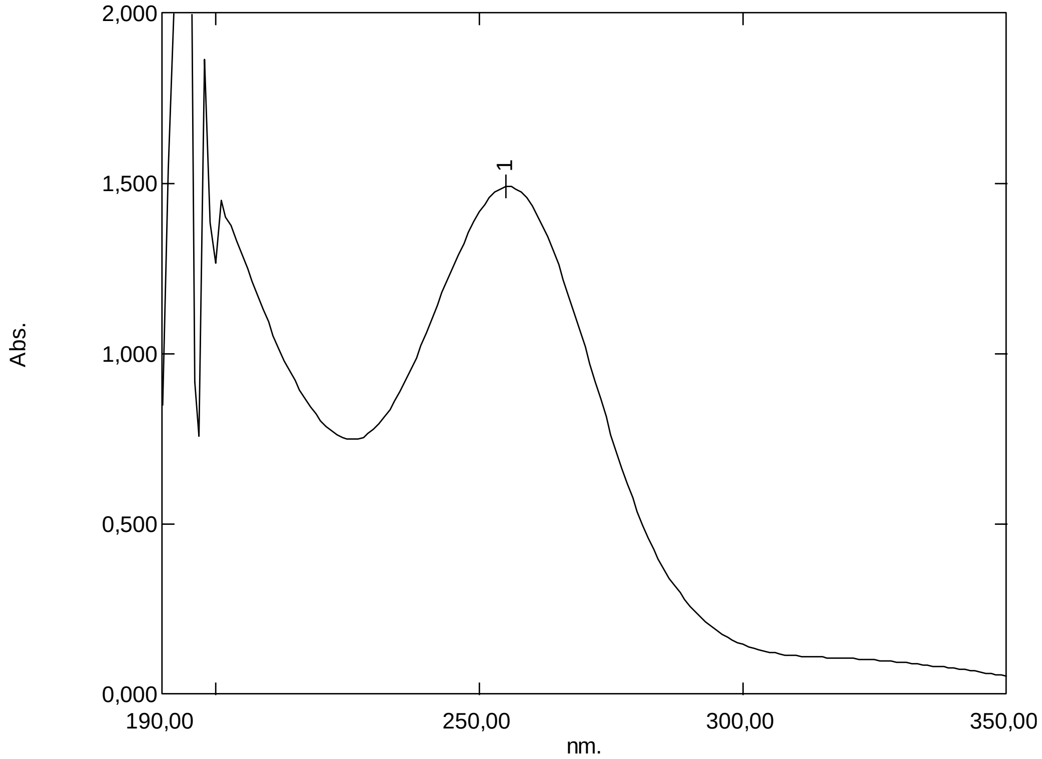

Figure S112. UV spectrum of compound **16**

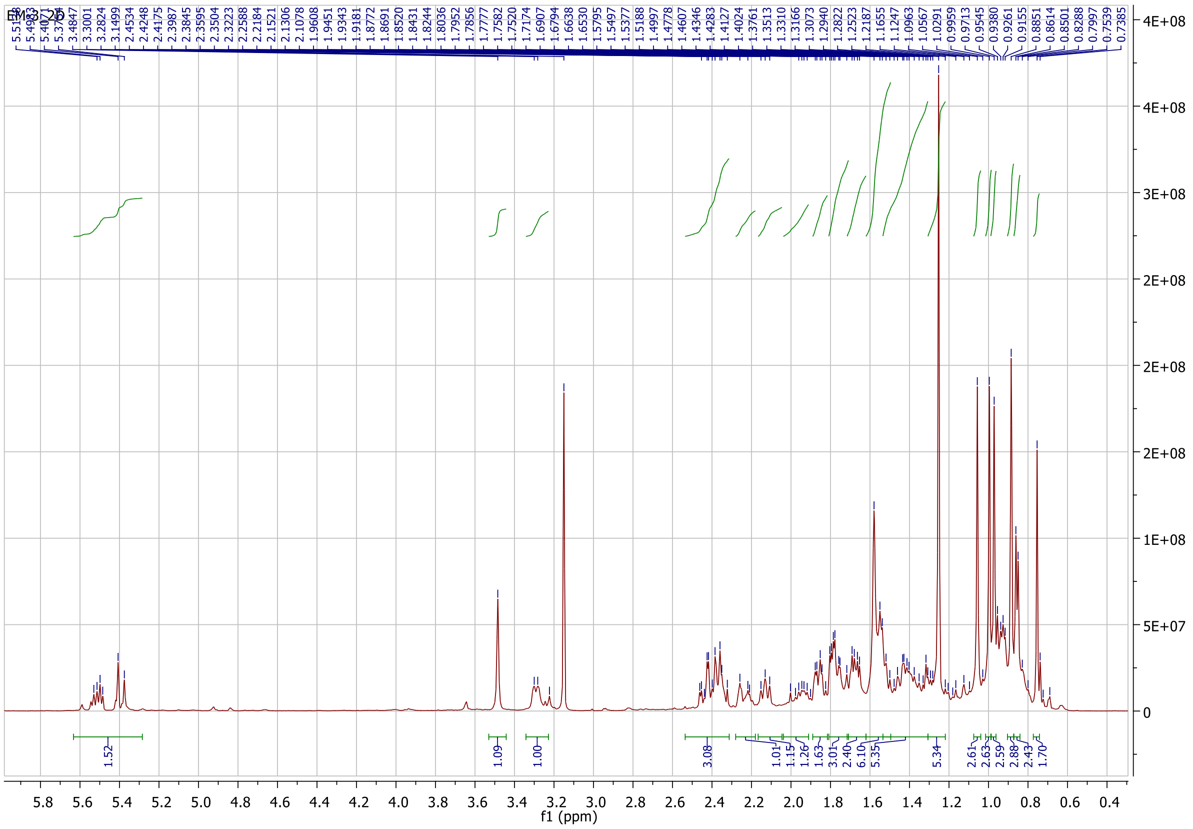

Figure S113. ^{1}H NMR spectrum compound **16** (500 MHz, $CDCl_3$)

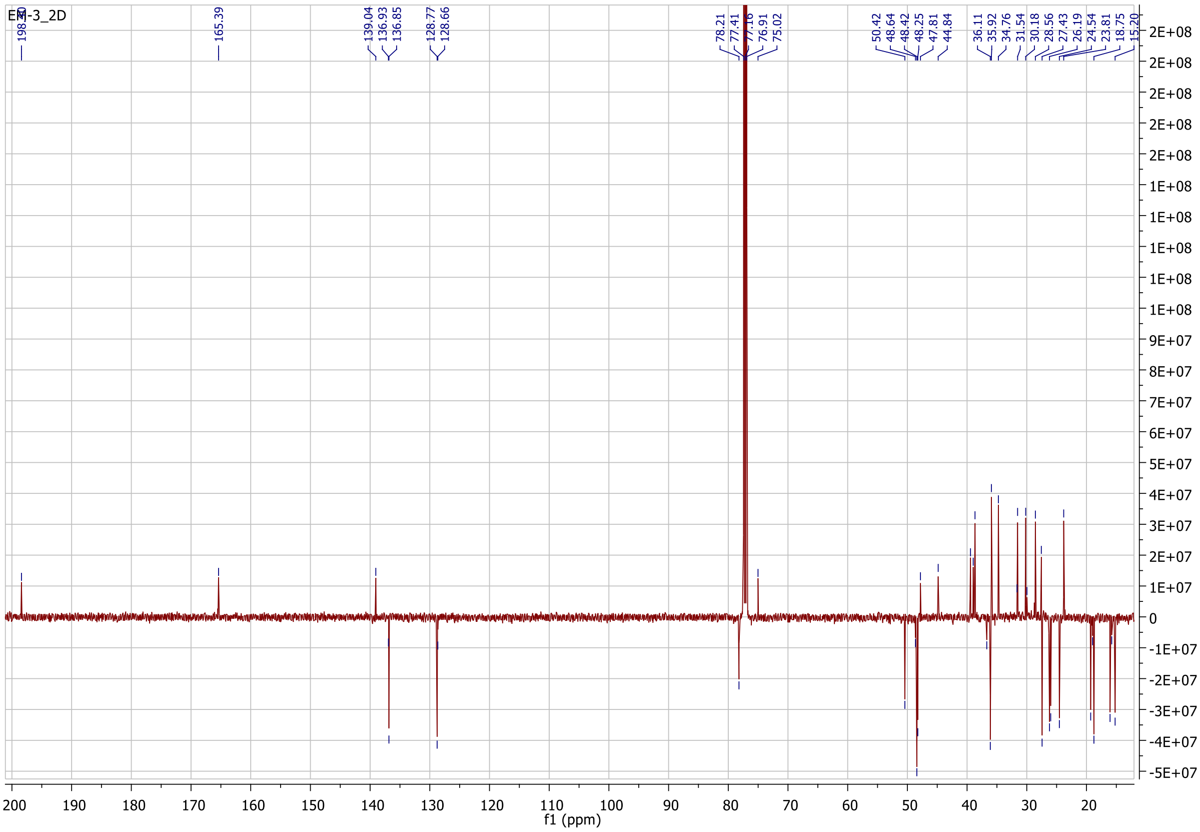

Figure S114. ^{13}C NMR JMOD spectrum compound **16** (125 MHz, $CDCl_3$)

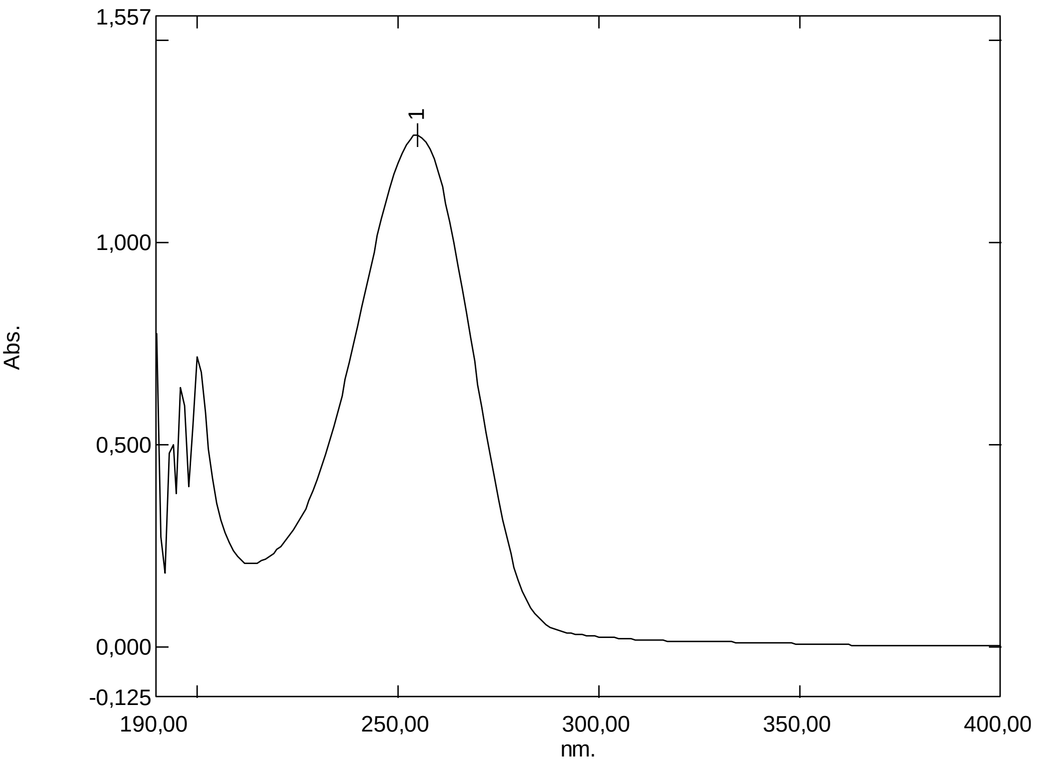

Figure S115. [1]UV spectrum of compound **17**

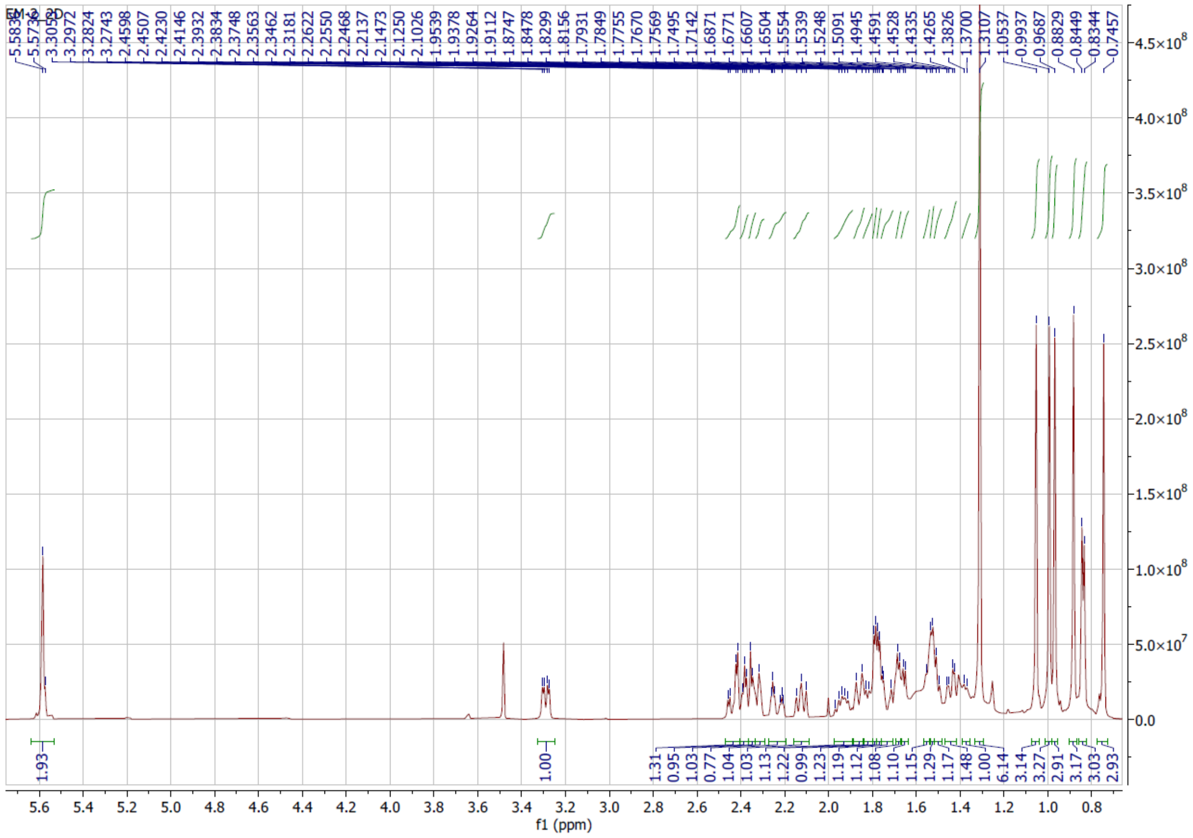

Figure S116. ^{1}H NMR spectrum of compound **17** (500 MHz, $CDCl_3$)

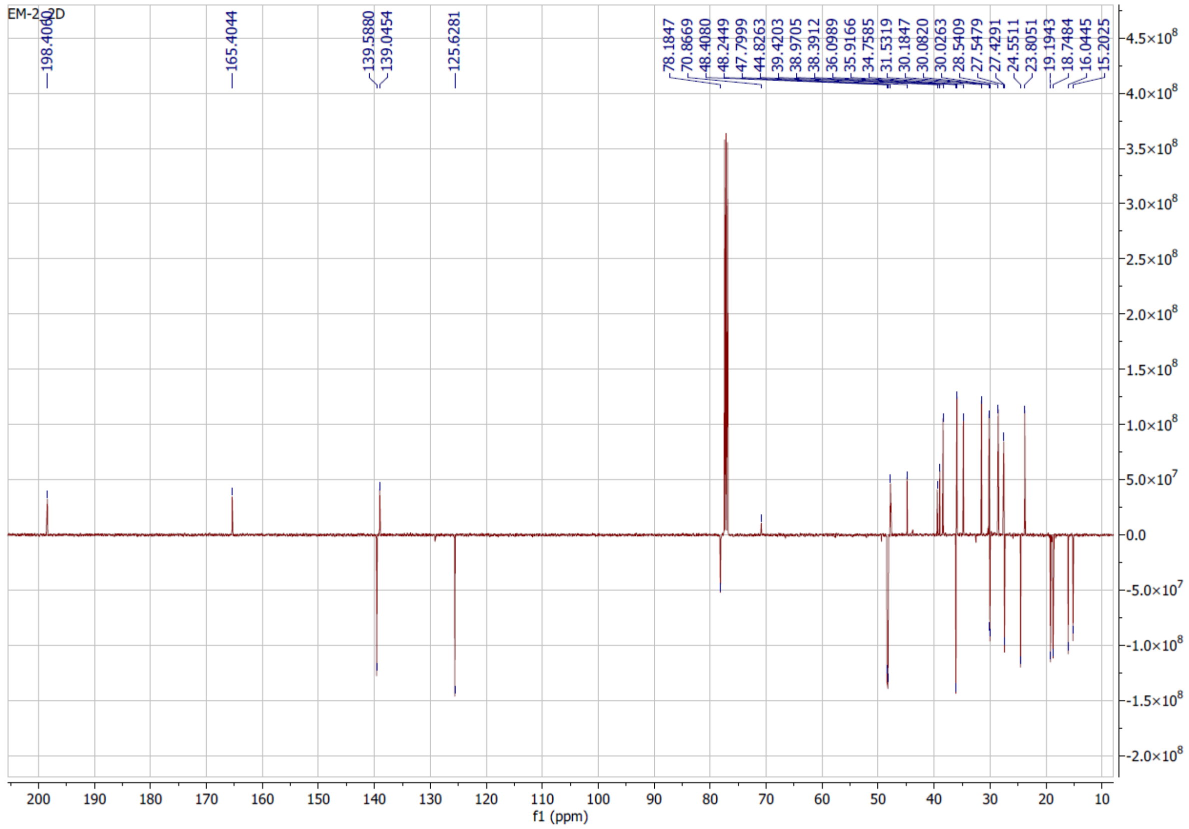

Figure S117. ^{13}C NMR JMOD spectrum of compound **17** (125 MHz, $CDCl_3$)

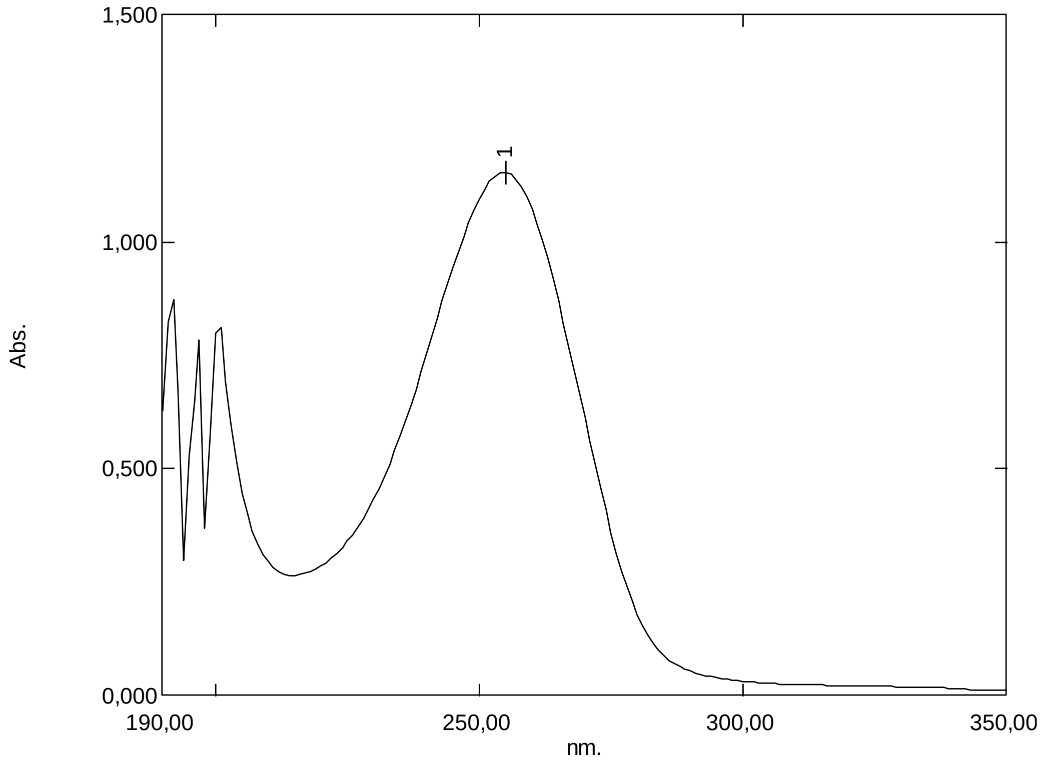

Figure S118. UV spectrum of compound **18**

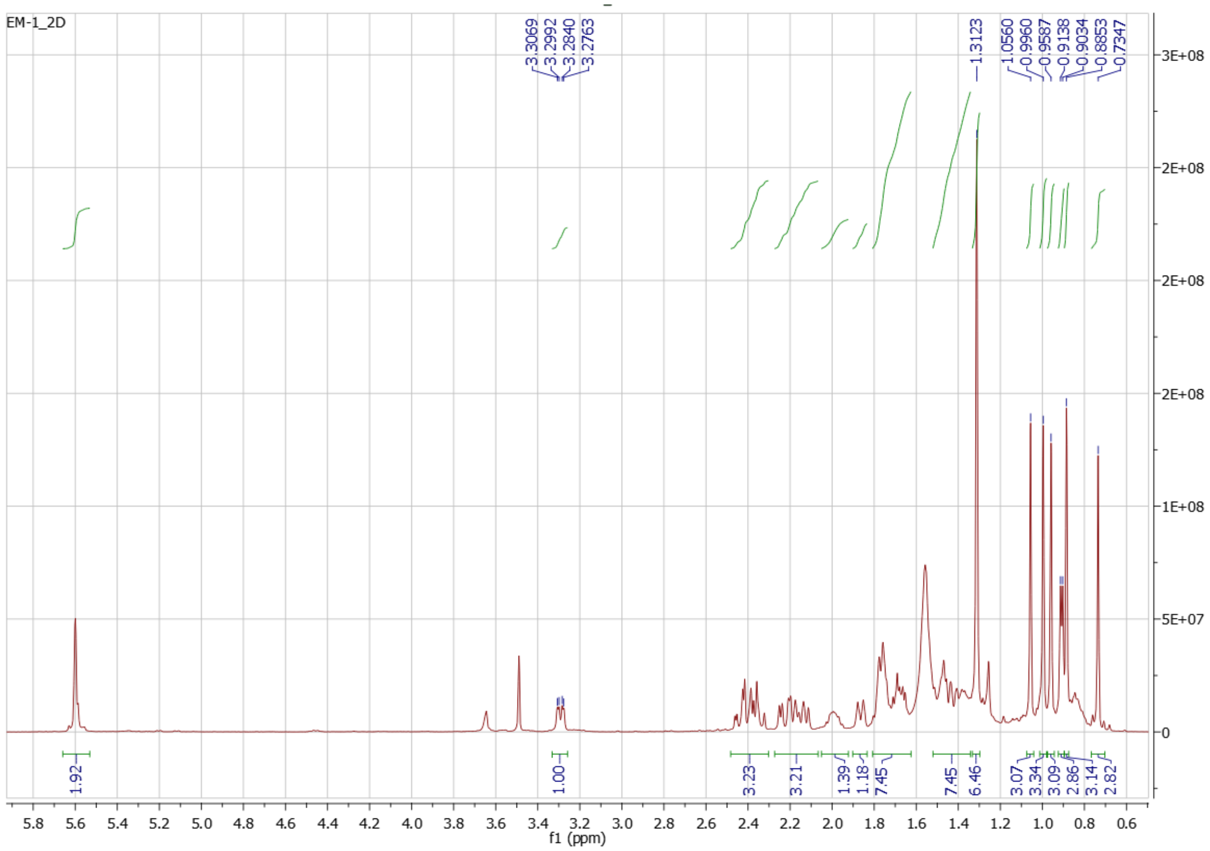

Figure S119. ^{1}H NMR spectrum of compound **18** (500 MHz, $CDCl_3$)

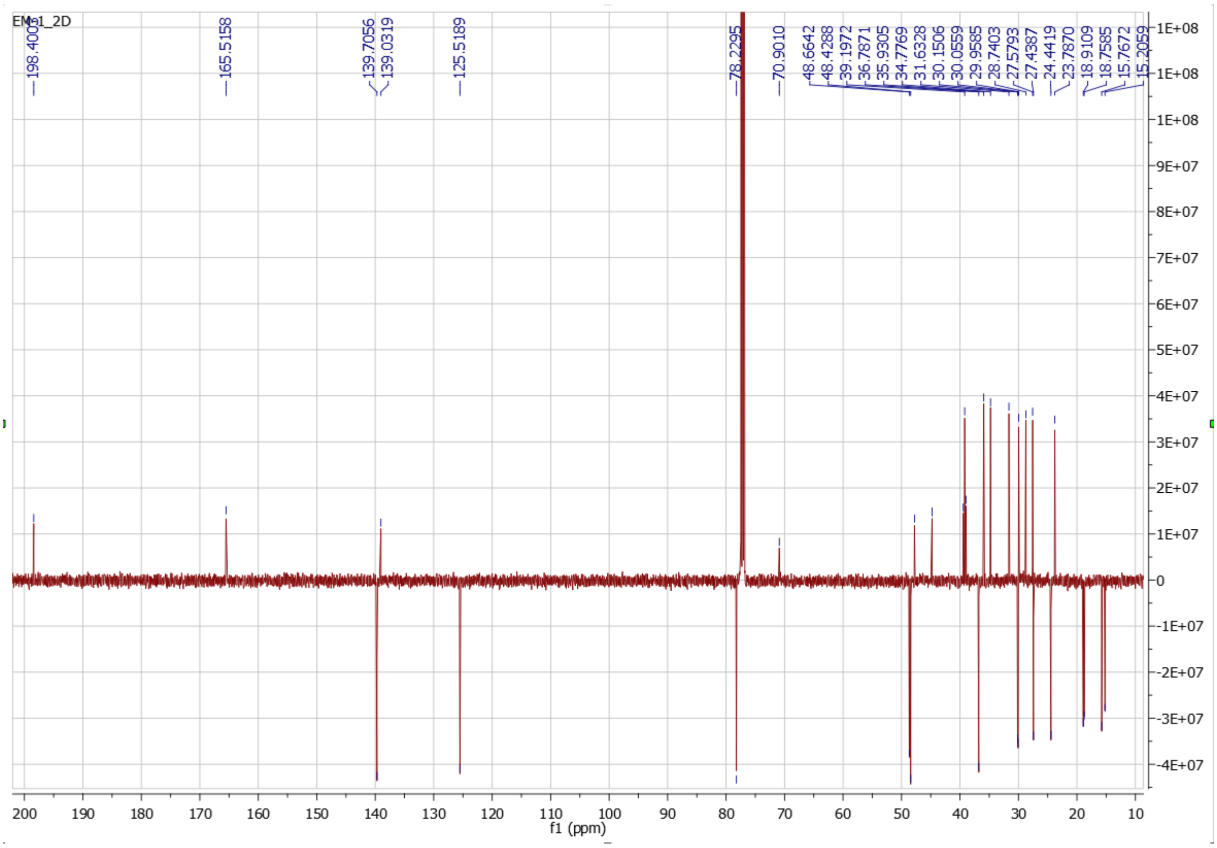

Figure S120. ^{13}C NMR JMOD spectrum of compound **18** (125 MHz, $CDCl_3$)

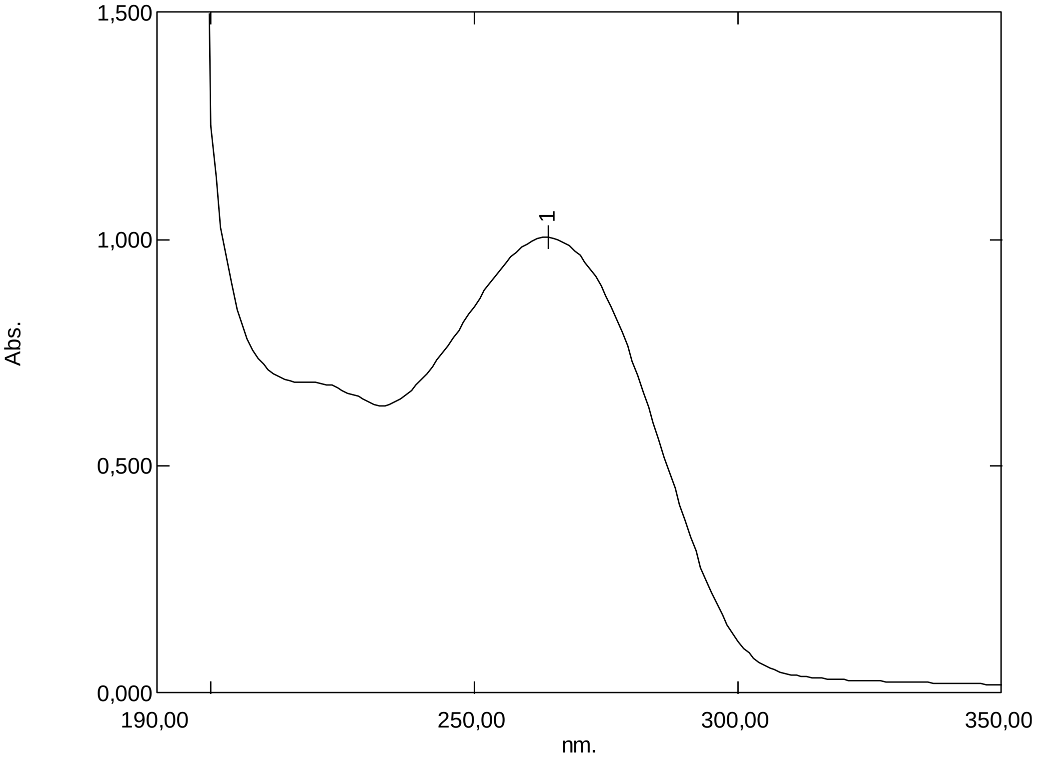

Figure S121. ^{1}H NMR spectrum of compound **19** (500 MHz, $CDCl_3$)

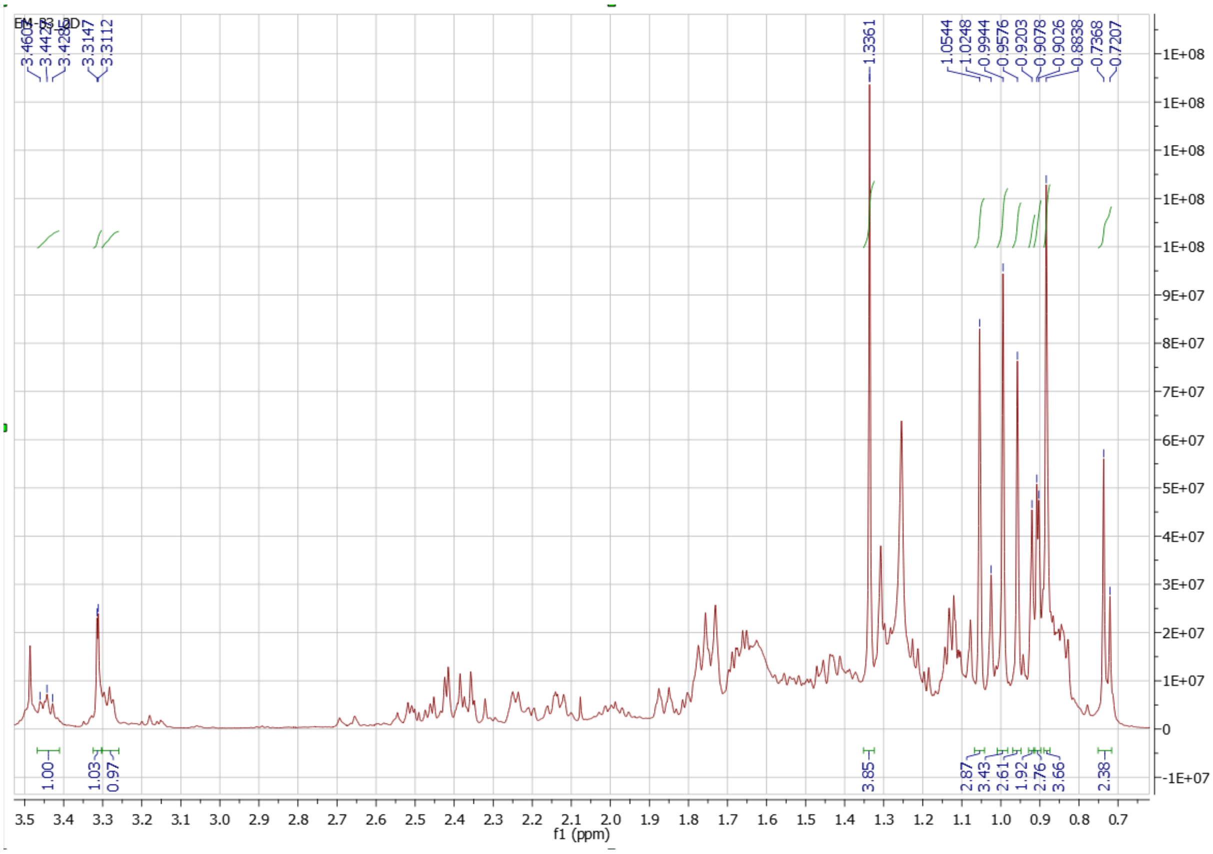

Figure S122. ^{1}H NMR spectrum of compound **19** (500 MHz, $CDCl_3$)

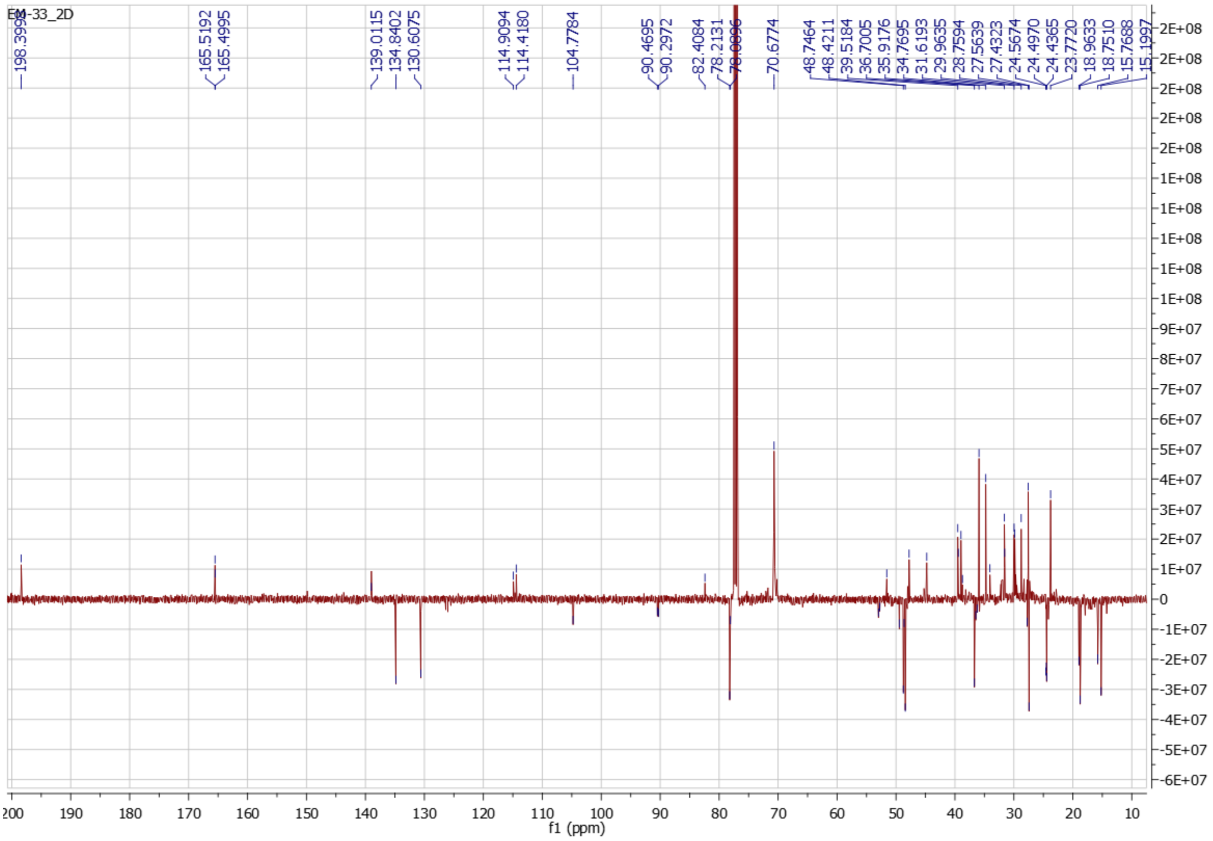

Figure S123. ^{13}C NMR JMOD spectrum of compound **19** (125 MHz, $CDCl_3$)

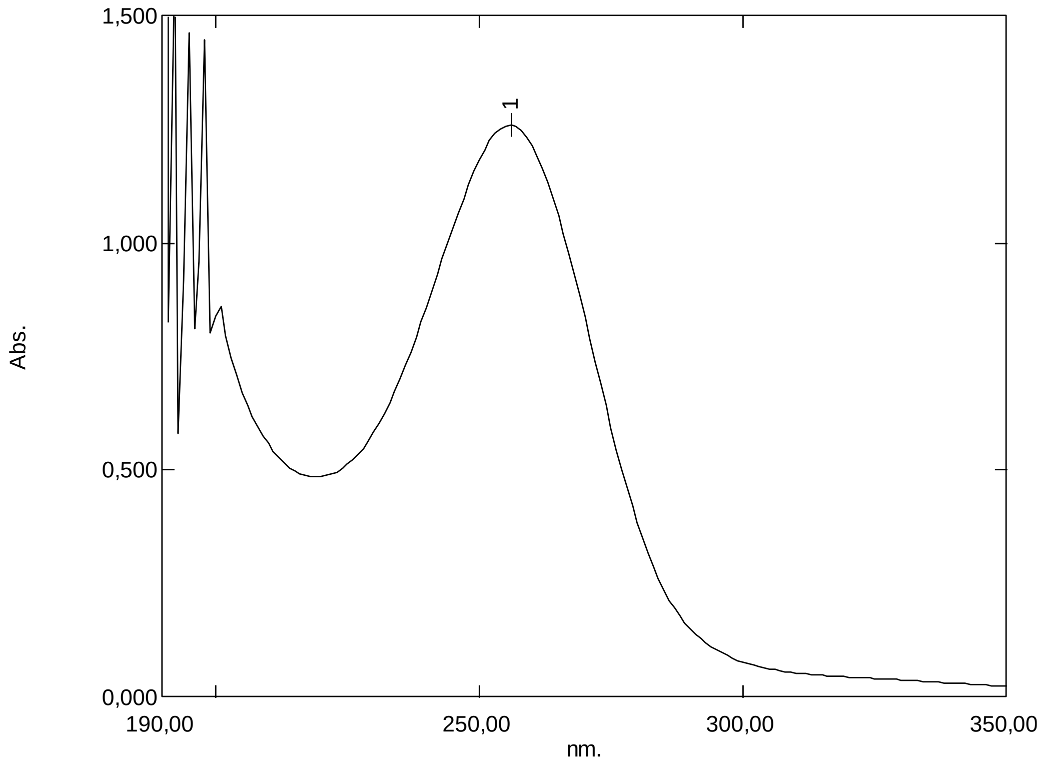

Figure S124. UV spectrum of compound **20** + **21**

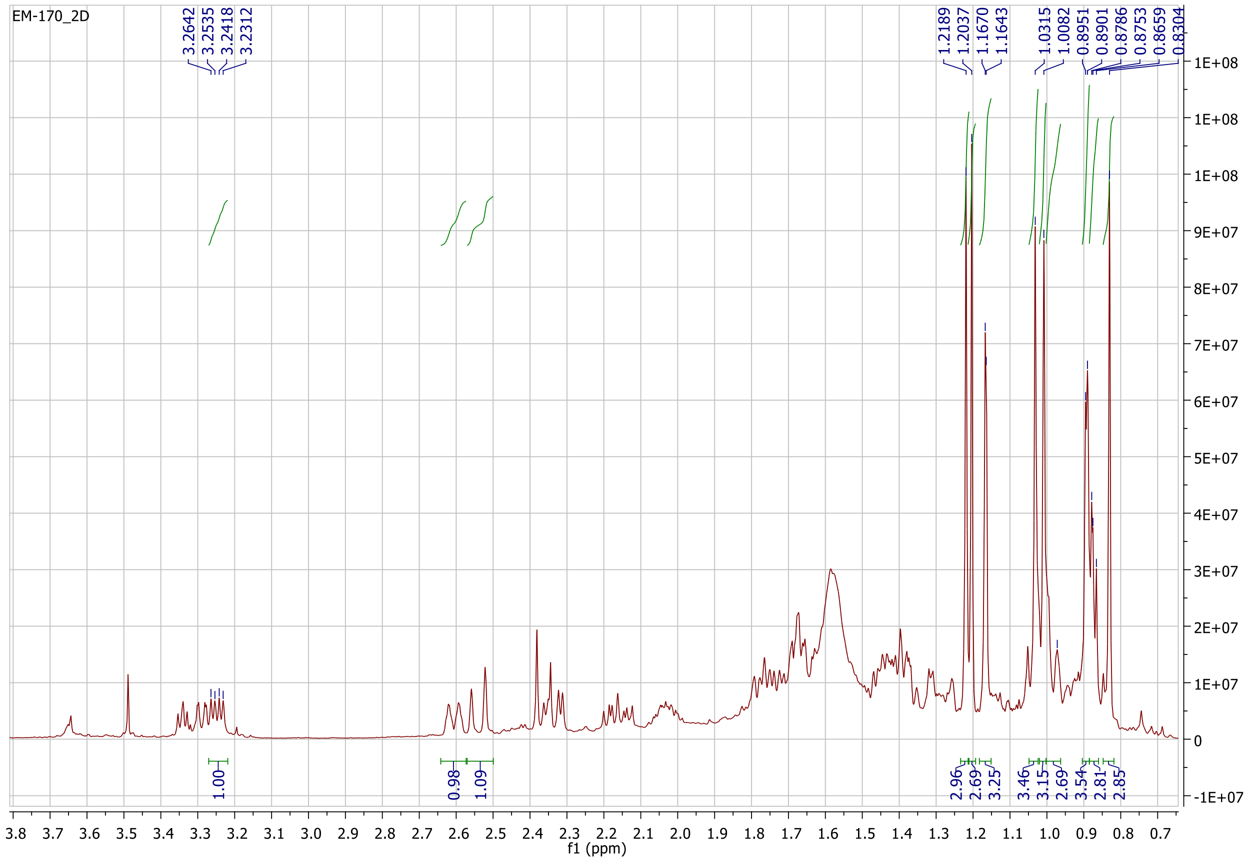

Figure S125. ^{1}H NMR spectrum of compound **20** + **21** (500 MHz, $CDCl_3$)

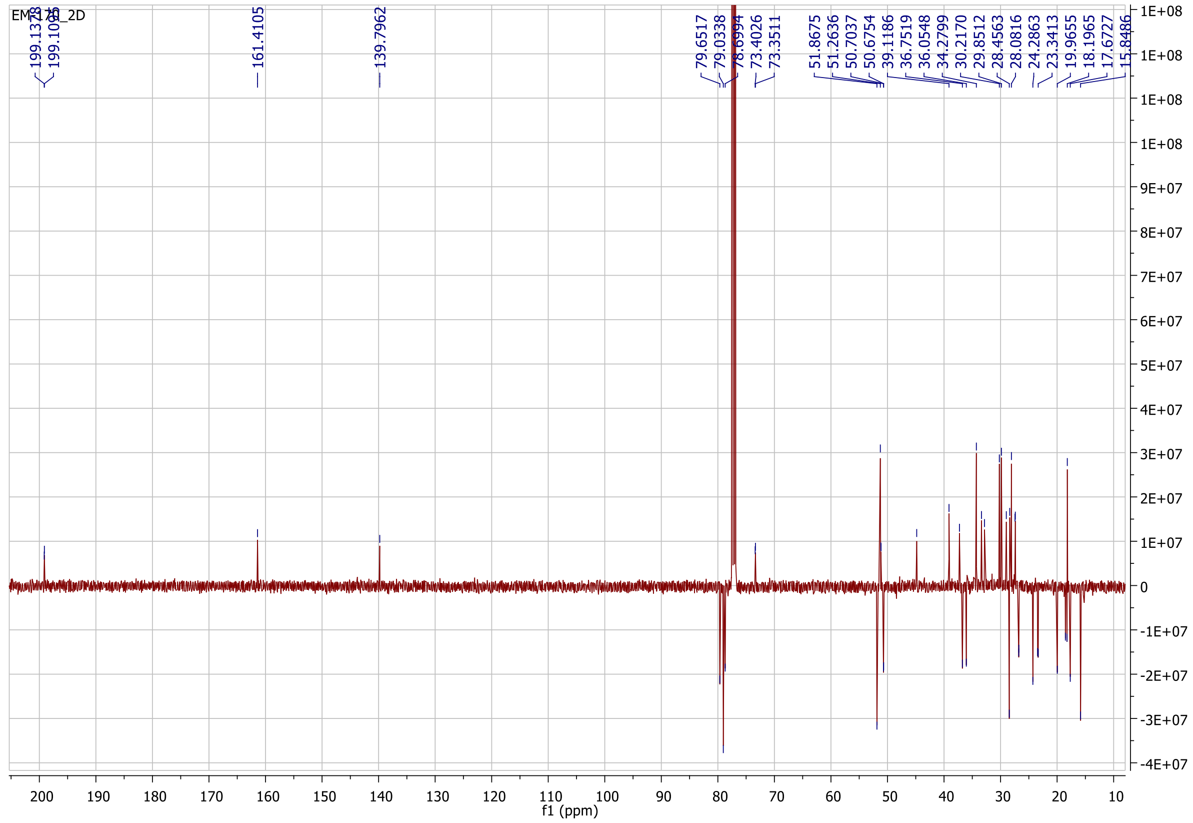

Figure S126. ^{13}C NMR JMOD spectrum of compound **20** + **21** (125 MHz, $CDCl_3$)

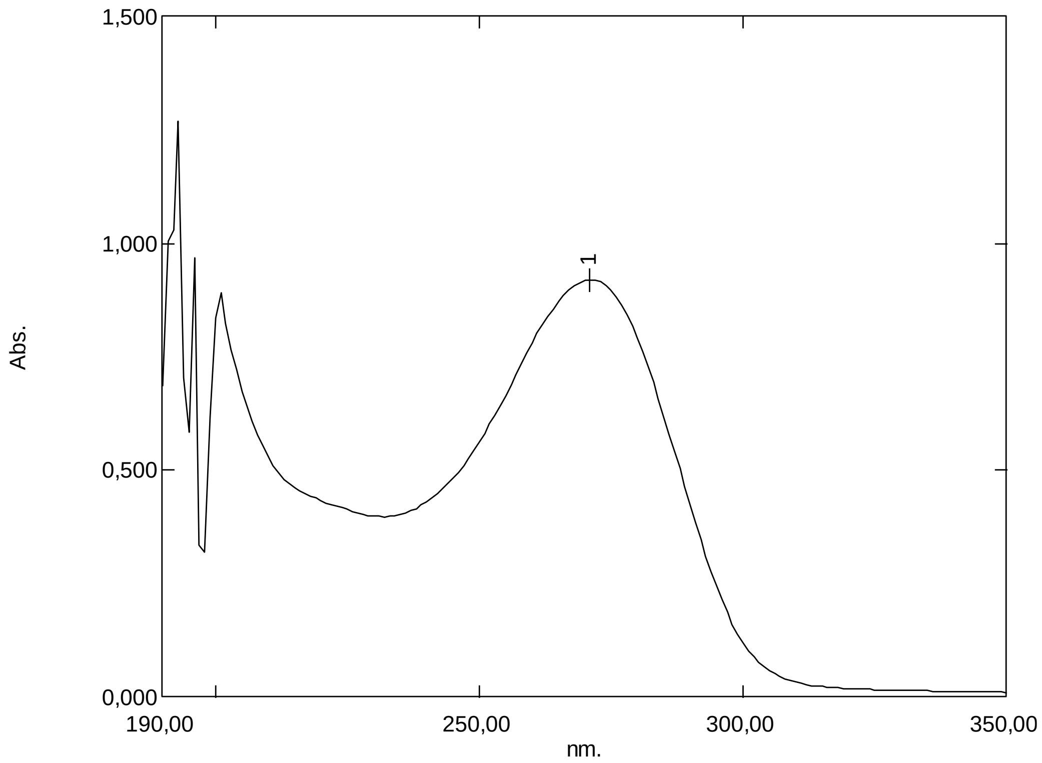

Figure S127. ^{1}H NMR spectrum of compound **22**

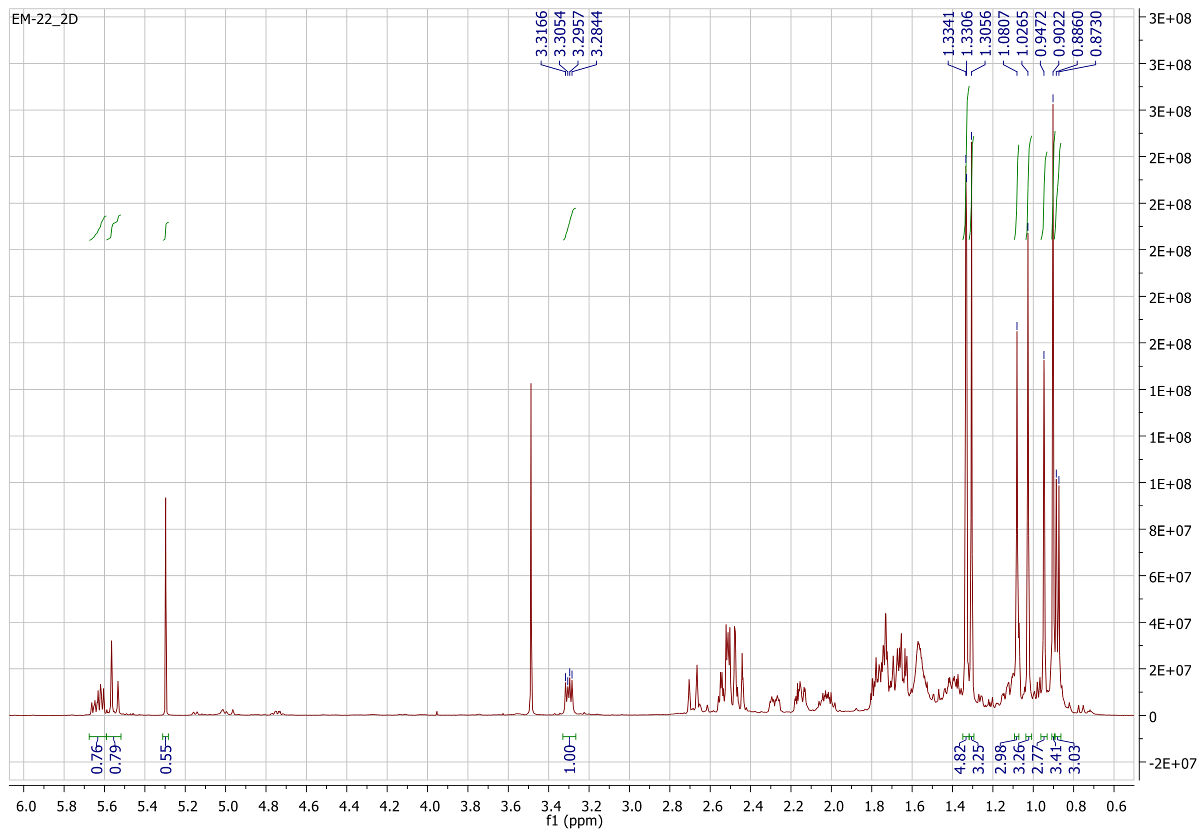

Figure S128. ^{1}H NMR spectrum of compound **22** (500 MHz, $CDCl_3$)

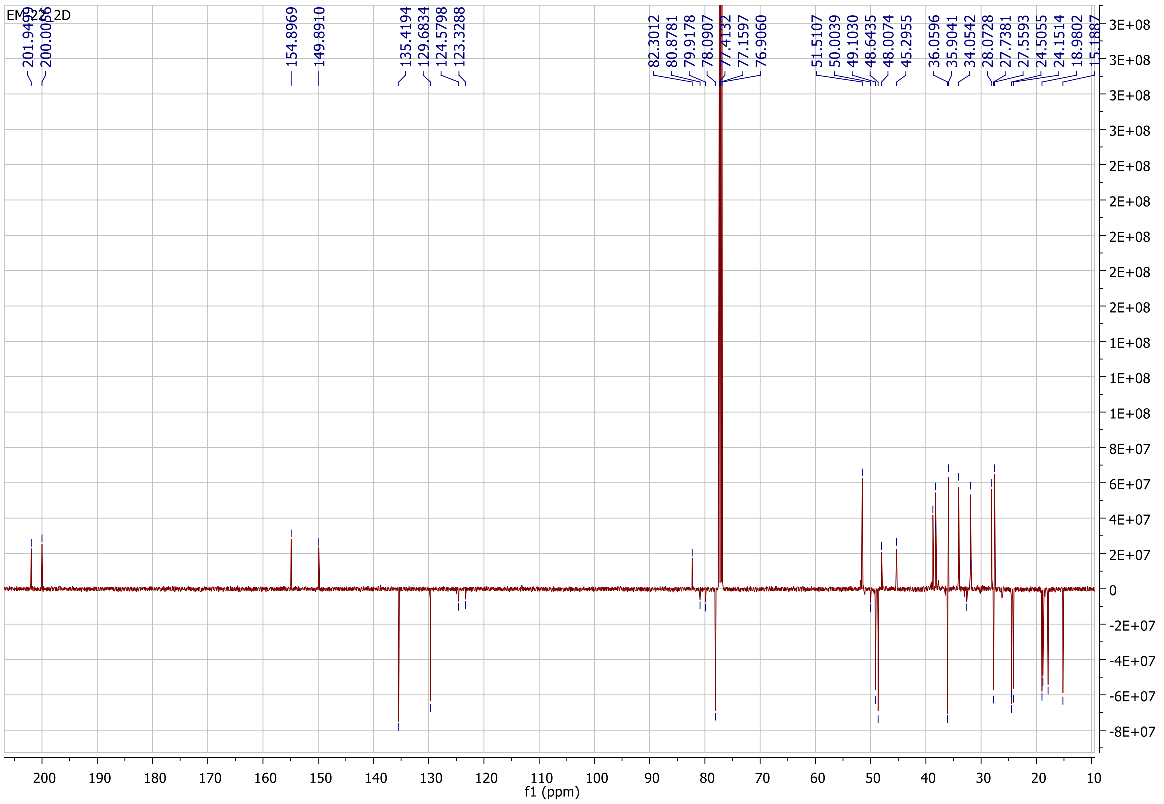

Figure S129. ^{13}C NMR JMOD spectrum of compound **22** (125 MHz, $CDCl_3$)

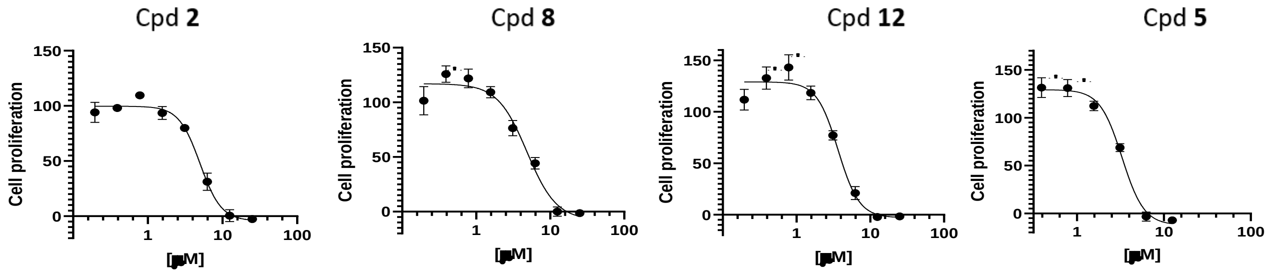

Figure S130. Compounds **2**, **5**, **8**, and **12** induced a significant increase in *T. cruzi* epimastigoge proliferation. Experiments show mean values ± SD of three independent experiments each performed in triplicates.
